# The burden of cancers and their variations across the states of India: the Global Burden of Disease Study 1990–2016

**DOI:** 10.1016/S1470-2045(18)30447-9

**Published:** 2018-10

**Authors:** India State-Level Disease Burden Initiative Cancer Collaborators, Preet K Dhillon, Prashant Mathur, A Nandakumar, Christina Fitzmaurice, G Anil Kumar, Ravi Mehrotra, D K Shukla, G K Rath, Prakash C Gupta, Rajaraman Swaminathan, J S Thakur, Subhojit Dey, Christine Allen, R A Badwe, Rajesh Dikshit, R S Dhaliwal, Tanvir Kaur, Amal C Kataki, Rudrapatna N Visweswara, P Gangadharan, Eliza Dutta, Melissa Furtado, Chris M Varghese, Deeksha Bhardwaj, Pallavi Muraleedharan, Christopher M Odell, Scott Glenn, Manjit S Bal, P P Bapsy, James Bennett, Vijay K Bodal, J K Chakma, Sekhar Chakravarty, Meesha Chaturvedi, Priyanka Das, Vinay Deshmane, Nitin Gangane, James Harvey, P Jayalekshmi, Kaling Jerang, Sarah C Johnson, P K Julka, Debnath Kaushik, Vinotsole Khamo, Shravani Koyande, Michael Kutz, W B Langstieh, K B Lingegowda, R C Mahajan, Jagadish Mahanta, Gautam Majumdar, N Manoharan, Aleyamma Mathew, B M Nene, Sanghamitra Pati, P K Pradhan, Vinod Raina, Ranganathan Rama, C Ramesh, K Sathishkumar, Kathryn Schelonka, Paul Sebastian, Katya Shackelford, Janmesh Shah, V Shanta, Jagannath D Sharma, Atul Shrivastava, Sopai Tawsik, Brij B Tyagi, K Vaitheeswaran, Elizabeth Vallikad, Yogesh Verma, Eric Zomawia, Stephen S Lim, Theo Vos, Rakhi Dandona, K Srinath Reddy, Mohsen Naghavi, Christopher J L Murray, Soumya Swaminathan, Lalit Dandona

## Abstract

**Background:**

Previous efforts to report estimates of cancer incidence and mortality in India and its different parts include the National Cancer Registry Programme Reports, Sample Registration System cause of death findings, Cancer Incidence in Five Continents Series, and GLOBOCAN. We present a comprehensive picture of the patterns and time trends of the burden of total cancer and specific cancer types in each state of India estimated as part of the Global Burden of Diseases, Injuries, and Risk Factors Study (GBD) 2016 because such a systematic compilation is not readily available.

**Methods:**

We used all accessible data from multiple sources, including 42 population-based cancer registries and the nationwide Sample Registration System of India, to estimate the incidence of 28 types of cancer in every state of India from 1990 to 2016 and the deaths and disability-adjusted life-years (DALYs) caused by them, as part of GBD 2016. We present incidence, DALYs, and death rates for all cancers together, and the trends of all types of cancers, highlighting the heterogeneity in the burden of specific types of cancers across the states of India. We also present the contribution of major risk factors to cancer DALYs in India.

**Findings:**

8·3% (95% uncertainty interval [UI] 7·9–8·6) of the total deaths and 5·0% (4·6–5·5) of the total DALYs in India in 2016 were due to cancer, which was double the contribution of cancer in 1990. However, the age-standardised incidence rate of cancer did not change substantially during this period. The age-standardised cancer DALY rate had a 2·6 times variation across the states of India in 2016. The ten cancers responsible for the highest proportion of cancer DALYs in India in 2016 were stomach (9·0% of the total cancer DALYs), breast (8·2%), lung (7·5%), lip and oral cavity (7·2%), pharynx other than nasopharynx (6·8%), colon and rectum (5·8%), leukaemia (5·2%), cervical (5·2%), oesophageal (4·3%), and brain and nervous system (3·5%) cancer. Among these cancers, the age-standardised incidence rate of breast cancer increased significantly by 40·7% (95% UI 7·0–85·6) from 1990 to 2016, whereas it decreased for stomach (39·7%; 34·3–44·0), lip and oral cavity (6·4%; 0·4–18·6), cervical (39·7%; 26·5–57·3), and oesophageal cancer (31·2%; 27·9–34·9), and leukaemia (16·1%; 4·3–24·2). We found substantial inter-state heterogeneity in the age-standardised incidence rate of the different types of cancers in 2016, with a 3·3 times to 11·6 times variation for the four most frequent cancers (lip and oral, breast, lung, and stomach). Tobacco use was the leading risk factor for cancers in India to which the highest proportion (10·9%) of cancer DALYs could be attributed in 2016.

**Interpretation:**

The substantial heterogeneity in the state-level incidence rate and health loss trends of the different types of cancer in India over this 26-year period should be taken into account to strengthen infrastructure and human resources for cancer prevention and control at both the national and state levels. These efforts should focus on the ten cancers contributing the highest DALYs in India, including cancers of the stomach, lung, pharynx other than nasopharynx, colon and rectum, leukaemia, oesophageal, and brain and nervous system, in addition to breast, lip and oral cavity, and cervical cancer, which are currently the focus of screening and early detection programmes.

**Funding:**

Bill & Melinda Gates Foundation; and Indian Council of Medical Research, Department of Health Research, Ministry of Health and Family Welfare, Government of India.

## Introduction

Cancer is the second leading cause of death globally after cardiovascular diseases.^1^ Patients with cancer generally have a poorer prognosis in low-income and middle-income countries, including India, because of relatively low cancer awareness, late diagnosis, and the lack of or inequitable access to affordable curative services compared with patients in high-income countries.^2^, ^3^ India has a population of 1·3 billion spread across 29 states and seven union territories, and many of the states are as large as other countries, with varying degrees of development, population genetics, environments and lifestyles, leading to a heterogeneous distribution of disease burden and health loss.^4^ There have been previous attempts to describe national-level patterns of cancer burden and epidemiology in different parts of India as well as areas of importance for cancer control,^5^, ^6^, ^7^, ^8^, ^9^, ^10^, ^11^, ^12^, ^13^, ^14^, ^15^, ^16^, ^17^, ^18^, ^19^, ^20^ but a systematic and comprehensive understanding of the magnitude and time trends of all types of cancers in each state of India is not readily available. This is needed to inform action for cancer control that is commensurate with the need in each state, since delivery of health care is a state subject in India.

**Research in context**

**Evidence before this study**

We searched PubMed and publicly available reports for estimates of cancer burden across the states of India using the search terms “burden”, “cancer”, “cause of death”, “death”, “DALY”, “epidemiology”, “incidence”, “India”, “morbidity”, “mortality”, “neoplasm”, “prevalence”, and “trends” on March 26, 2018, without language or publication date restrictions. We found a wide variety of valuable data for cancer distribution in India and several states, but no studies that comprehensively described incidence, prevalence, mortality, and disability-adjusted life-years (DALYs) for all cancer types in every state of India over a long period of time.

**Added value of this study**

To our knowledge, this is the first report to produce comprehensive estimates of incidence, prevalence, mortality, and DALYs for 28 types of cancers in every state of India over 26 years from 1990 to 2016. This analysis was part of the Global Burden of Diseases, Injuries, and Risk Factors Study 2016 that assessed all causes of disease burden, using data from all accessible sources. This study estimates that while the age-standardised incidence rate of all cancers considered together has not changed substantially in India during this 26-year period, the proportion of total disease burden caused by cancers has doubled. This study reports the substantial heterogeneity in the incidence, mortality, and DALYs of different cancers across the states of India and documents that tobacco use is the highest contributing risk to cancer burden in India.

**Implications of all the available evidence**

This systematic and comprehensive description of the variations in the distribution and trends of cancer types in different parts of the country can serve as a useful reference for further planning of prevention and management of cancer across India. Additionally, cancer registry coverage should increase in rural areas and some large states of India that do not currently have registries. More large-scale collaborative research is needed to understand the reasons behind the changing trends of the different types of cancers in India.

The United Nations Sustainable Development Goals target the reduction of premature mortality from non-communicable diseases, which includes cancer, by one-third by 2030 through prevention and treatment.^21^ The National Cancer Registry Programme in India was established in 1981 to generate data on the magnitude and patterns of cancer through population-based registries.^22^, ^23^ The number of registries has grown under this programme, and other population-based registries have also been started in recent years.^22^ However, many populous states have no cancer registries yet, and most registries in India are in urban areas, leading to difficulties in assessing population-level cancer burden trends in all parts of the country.

The India State-Level Disease Burden Initiative is a collaboration with the Global Burden of Diseases, Injuries, and Risk Factors Study (GBD) to produce subnational disease burden estimates for India. This initiative recently reported the variable health transition across the states of India from 1990 to 2016 based on analysis done as part of GBD 2016.^4^, ^24^ Here, we report detailed trends of the incidence and health loss due to each type of cancer in every state of India from 1990 to 2016.

## Methods

### Overview

The India State-Level Disease Burden Initiative recently reported the overall trends of diseases, injuries, and risk factors from 1990 to 2016 for every state of India.^4^, ^24^ This analysis was done as part of GBD 2016, which estimated disease burden due to 333 diseases and injuries and 84 risk factors in all age groups using all accessible data from multiple sources. The India State-Level Disease Burden Initiative was supported by the efforts of several expert groups and a vast network of collaborators to identify and access all available data sources, assess their scope and quality for inclusion, and participate in the analysis and interpretation of the findings. The Health Ministry Screening Committee at the Indian Council of Medical Research and the ethics committee of the Public Health Foundation of India approved the work of this initiative.

### Estimation of cancer burden

A detailed description of methods to estimate cancer mortality, incidence, prevalence, and disability-adjusted life-years (DALYs), and the analytical approaches used in GBD 2016 have been reported elsewhere, and are summarised in the appendix (pp 3–16).^1^, ^25^, ^26^, ^27^, ^28^, ^29^ Briefly, the major data inputs to determine cancer mortality in India included the nationwide Sample Registration System (SRS) cause of death data, the Medically Certified Cause of Death data, and 42 population-based cancer registries (appendix pp 6–8). SRS verbal autopsy cause of death data on 455 460 deaths covering the rural and urban populations of every state of India from 2004 to 2013 were included.^4^ For states with at least one population-based cancer registry, the incidence data were transformed to mortality by multiplying incidence data with an independently modelled urban or rural mortality-incidence (MI) ratio for the respective states. Cancer registry data were used as the gold-standard against which other data sources (SRS or Medically Certified Cause of Death data) were compared. If the other data sources differed substantially from the registry data, they were excluded.^1^, ^30^ Because of limitations associated with the Medically Certified Cause of Death mortality data, these were used only when the cancer type was not captured by the SRS cause of death data. The combined data for cancer mortality were used in a modelling approach (CODEm), where an ensemble of plausible models is selected. The CoDCorrect algorithm was used to adjust cancer subtypes to the parent cause and to adjust the sum of predicted deaths from these models for each type of cancers in an age–sex–state–year group to be consistent with the results from all-cause mortality estimation.

The estimation of cancer incidence was driven by registry data from India. The mortality estimates that were derived from transformation of incidence data using the MI ratios, as noted above, were transformed back to incidence after the CODEm and CoDCorrect model adjustments.^1^ 10-year cancer prevalence was estimated by modelling survival using the MI ratio as a surrogate for access to cancer care. Incidence cohorts were scaled between a theoretical best and worst case survival using the MI ratio scaling factor. Lifetime prevalence was only estimated for 10 years post incidence^1^ and for long-term sequelae from procedures (mastectomy, laryngectomy, stoma, incontinence cystectomy, and prostatectomy). Disability for each cancer was estimated by splitting the prevalence into four sequelae: diagnosis and primary treatment, controlled phase, metastatic phase, and terminal phase. Each prevalence sequela was multiplied with specific disability weights to determine years lived with disability (YLDs). We computed years of life lost (YLLs) from the age-specific mortality estimates and a reference life expectancy for that age group. DALYs, a summary measure of total health loss, were computed by adding YLLs and YLDs for each cancer type for location, year, age and sex.^1^, ^28^ The appendix provides a list of data inputs used for these estimations (pp 17–29).

A description of estimation of risk factor exposure and its contribution to disease burden in GBD is available elsewhere.^29^ Briefly, this includes determination of risk exposure and disease outcome pairs based on available evidence and inclusion criteria, assessment of risk exposure from all accessible data sources, and estimation of disease burden attributable to risks based on published relative risks. Estimates of DALYs for specific types of cancers that were attributable to each risk factor were produced by location, age, sex, and year.

GBD uses covariates, which are explanatory variables that have a known association with the outcome of interest, to arrive at the best possible estimate of the outcome of interest when data for the outcome are scarce but data for the covariates are available.^25^, ^26^, ^27^, ^28^, ^29^ This approach was part of the estimation process for the findings presented in this report.

### Analysis presented in this paper

The findings are reported for 31 geographical units in India: 29 states, Union Territory of Delhi, and the union territories other than Delhi (combining the six smaller union territories of Andaman and Nicobar Islands, Chandigarh, Dadra and Nagar Haveli, Daman and Diu, Lakshadweep, and Puducherry). The states of Chhattisgarh, Uttarakhand, and Jharkhand were created from existing larger states in 2000, and the state of Telangana was created in 2014. For trends from 1990 onwards, the data for these four new states were disaggregated from their parent states on the basis of data from the districts that now constitute these states. The findings are also presented for four groups of states based on epidemiological transition level (ETL) as described previously.^4^ Briefly, the ETL state groups were defined on the basis of the ratio of DALYs from communicable, maternal, neonatal and nutritional diseases to those from non-communicable diseases and injuries combined in 2016, with a relatively lower ratio indicating higher ETL: low level ETL state group (ratio 0·56–0·75), lower-middle ETL state group (0·41–0·55), higher-middle ETL state group (0·31–0·40), and high ETL state group (less than 0·31). We have reported previously that epidemiological transition ratios of the states of India have a significant inverse relation with the Socio-demographic Index calculated by the GBD study and based on income, education, and fertility levels, which indicates broad correspondence of ETL groups with sociodemographic development levels.^4^

We present trends of incidence (new cases in a year), mortality, and DALYs due to all cancers together and different types of cancers from 1990 to 2016 for every state of India. We regarded DALYs as the main metric for disease burden because it includes both mortality and morbidity and is recommended by the National Health Policy of India for tracking disease burden.^31^ First, we present the 1990 to 2016 trends for the overall burden from all cancers together in terms of DALYs, followed by incidence and deaths. We describe the trends of ten cancer types that are responsible for the highest proportion of cancer DALYs in India in 2016. We also describe briefly another six cancer types that are among the ten leading incident cancers in females or males in India in 2016, which are not included in the previous ten cancers causing the highest DALYs. We present the age distribution of DALYs due to types of cancers. We also highlight relevant differences between females and males in the distribution of cancers. We present briefly findings related to the major risk factors contributing to cancer DALYs in India as estimated by GBD.

The estimates were produced initially for each state. We then computed the population-weighted mean of these state estimates as the estimate for India. We present both crude and age-standardised rates, since crude rates provide the actual situation in each state that is preferred by policy makers, and age-standardised rates allow comparisons over time and between states after adjusting for the differences in the age structure of the population. We based age-standardised rates on the GBD global reference population.^25^ We report estimates with 95% uncertainty intervals (UIs) where relevant. These were based on 1000 runs of the models for each quantity of interest, with the mean considered as the point estimate and the 2·5th and 97·5th percentiles considered as the 95% UI (appendix p 15).^25^, ^26^, ^27^, ^28^

### Role of the funding source

Some staff of the Indian Council of Medical Research are co-authors on this paper as they contributed to various aspects of the study and analysis. The other funder of the study had no role in the study design, data collection, data analysis, data interpretation, or writing of this paper. The corresponding author had full access to all of the data in the study and had final responsibility for the decision to submit for publication.

## Results

All cancers together contributed 5·0% (95% UI 4·6–5·5) of the total DALYs and 8·3% (7·9–8·6) of the total deaths in India in 2016, an increase of 90·9% and 112·8% respectively from 1990. The crude cancer DALY rate in India increased by 25·3% (16·8–34·2) from 1990 to 2016, but the age-standardised cancer DALY rate did not change substantially during the same period.^32^ The age-standardised cancer DALY rate had a 2·6 times variation across the states of India in 2016 (appendix p 30). The highest crude cancer DALY rates in 2016 were in the states of Mizoram, Kerala, Assam, Haryana, and Meghalaya, and the highest age-standardised rates were in the northeast states of Mizoram, Meghalaya, Arunachal Pradesh, and Assam (appendix p 30).

**Figure 1:**
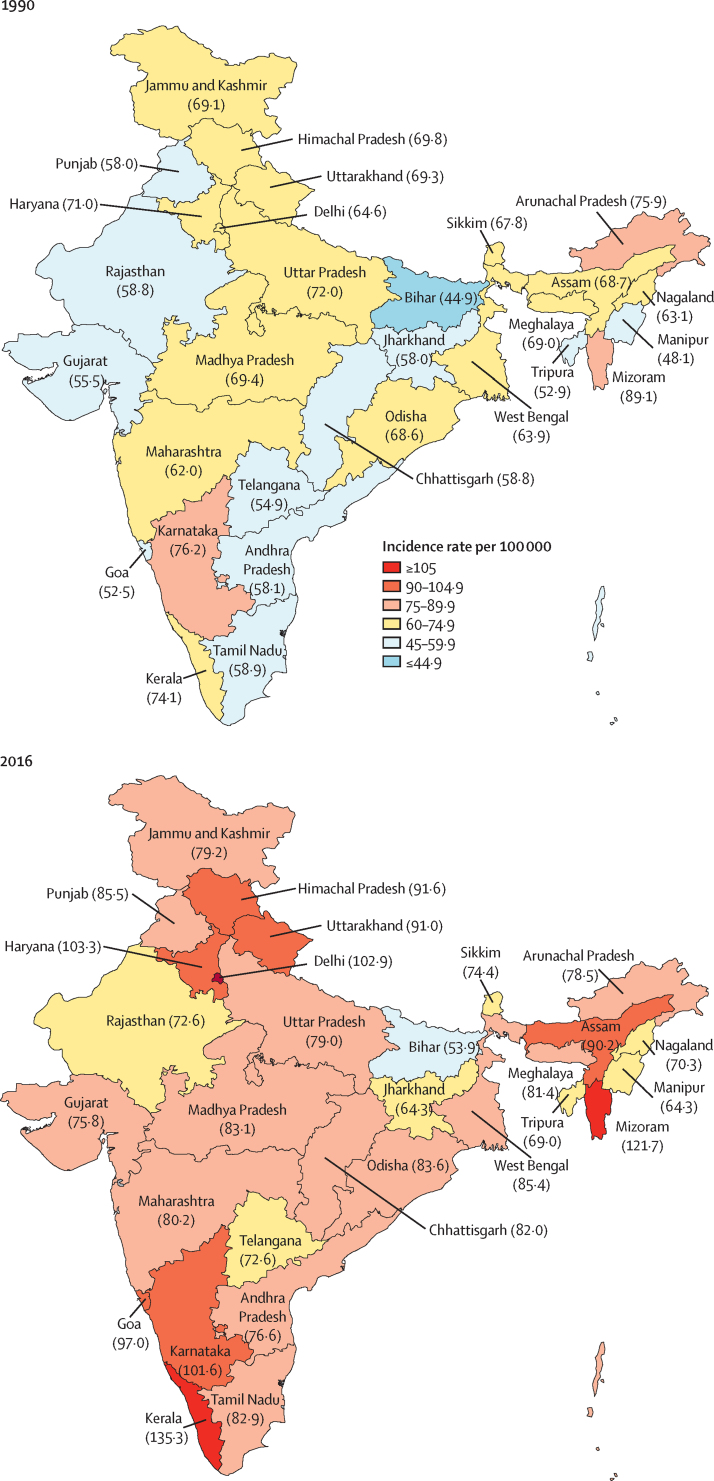
Crude annual incidence rate of all cancers together in the states of India, 1990 and 2016 The states of Chhattisgarh, Jharkhand, Telangana, and Uttarakhand did not exist in 1990, as they were created from existing larger states in 2000 or later. Data for these four new states were disaggregated from their parent states based on their current district composition. These states are shown in the 1990 map for comparison with 2016.

The estimated number of incident cancer cases in India increased from 548 000 (95% UI 520 000–576 000) in 1990 to 1 069 000 (1 043 000–1 101 000) in 2016 (appendix p 31). The crude cancer incidence rate in India increased by 28·2% (95% UI 19·9–35·5) from 63·4 per 100 000 in 1990 to 81·2 per 100 000 in 2016, but there was no change in the age-standardised incidence rate (appendix p 32). Crude cancer incidence rate was highest in Kerala and Mizoram, followed by Haryana, Delhi, Karnataka, Goa, Himachal Pradesh, Uttarakhand, and Assam (figure 1, appendix p 30). Age-standardised incidence rates were highest in the northeast states of Mizoram, Meghalaya, Arunachal Pradesh, and Assam, and in Delhi and Haryana. Crude cancer incidence rate increased substantially from 1990 to 2016 in all ETL state groups, with the highest increase in the high ETL group (appendix p 32).

The number of deaths due to cancer in India increased from 382 000 (95% UI 351 000–412 000) in 1990 to 813 000 (767 000–850 000) in 2016 (appendix p 31). The crude cancer death rate in India in 2016 was 61·8 (95% UI 58·3–64·6) per 100 000, as compared with 44·2 (40·6–47·7) in 1990 (appendix p 32). Male cancer patients had a 12·3% (95% UI 2·9–23·3) increase in age-standardised death rate over the 26-year time period, whereas no substantial changes over time were found in female cancer patients (appendix p 32). There was no significant difference between the ETL state groups for the crude death rate in 2016 (appendix p 32). The crude death rate for both sexes combined was highest in Mizoram, Kerala, and Haryana in 2016, followed by Assam, Karnataka, Odisha, Uttarakhand, Meghalaya, and Himachal Pradesh (appendix pp 30, 33). The age-standardised death rates were highest in the northeast states of Mizoram, Meghalaya, Arunachal Pradesh, and Assam (appendix p 30).

**Figure 2:**
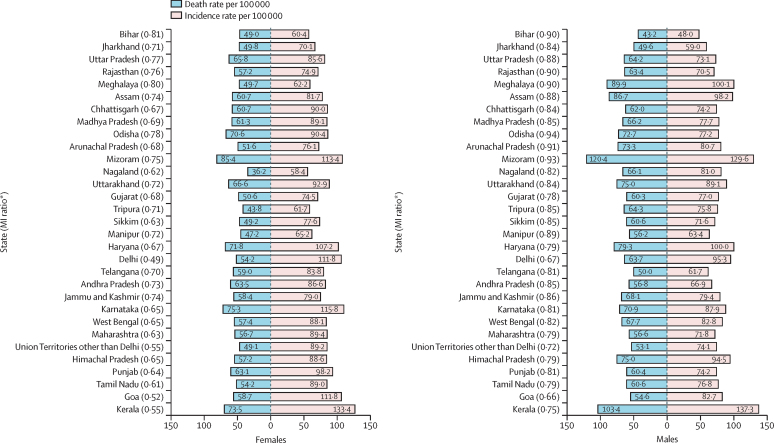
Crude MI ratio of all cancers together in the states of India by sex, 2016 MI=mortality-incidence. *MI ratio is is calculated by dividing crude death rate per 100 000 by the crude incidence rate per 100 000. The sequence of the states is from the lowest to the highest epidemiological transition level in 2016.

The crude cancer MI ratio in India in 2016 was 0·76 (95% UI 0·72–0·79). The MI ratio was higher in low and lower-middle ETL state groups than the high and higher-middle ETL groups in 2016 (figure 2). The overall MI ratio was significantly higher in males (0·83; 95% UI 0·81–0·85) than females (0·69; 0·64–0·71) in 2016. The MI ratio for males was more than 0·80 in all states from the low ETL group and most of the lower-middle ETL states, with a higher ratio in Odisha in low ETL, and Mizoram and Arunachal Pradesh in the lower-middle ETL state group in 2016. The MI ratio was below 0·80 for females in most of the states in India in 2016, except for Bihar and Meghalaya.

**Figure 3:**
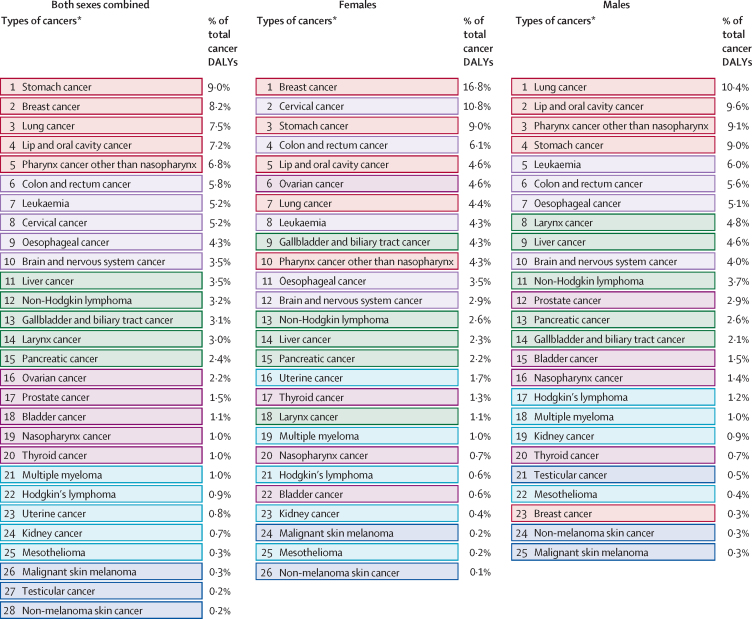
Percentage of total cancer DALYs due to different types of cancers by sex in India, 2016 DALYs=disability-adjusted life-years. *The other neoplasm category was not included in this figure. The types of cancers are colour-coded in groups based on their ranking in both sexes combined.

**Figure 4:**
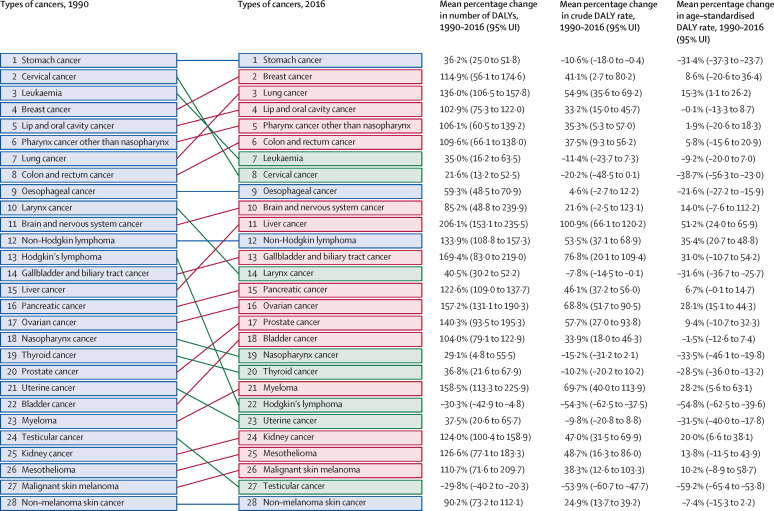
Change in DALYs for different types of cancers in India, 1990–2016 DALYs=disability-adjusted life-years.

The leading types of cancer in India in 2016, those responsible for more than 5% of the total cancer DALYs among both sexes combined, were stomach cancer (9·0%), breast cancer (8·2%), lung cancer (7·5%), lip and oral cavity cancer (7·2%), pharynx cancer other than nasopharynx (6·8%), colon and rectum cancer (5·8%), leukaemia (5·2%), and cervical cancer (5·2%; figure 3). Stomach cancer was responsible for the highest DALYs among all cancers in India in both 1990 and 2016 (figure 4). The age-standardised DALY rate increased significantly in India from 1990 to 2016 for liver cancer (51·2%; 95% UI 24·0–65·9), non-Hodgkin lymphoma (35·4%; 20·7–48·8), ovarian cancer (28·1%; 15·1–44·3) and myeloma (28·2%; 5·6–63·1). The reduction in age-standardised DALY rates from 1990 to 2016 was highest for testicular cancer (59·2%; 53·8–65·4) and Hodgkin's lymphoma (54·8%; 39·6–62·5), followed by cervical (38·7%; 23·0–56·3), nasopharynx (33·5%; 19·8–46·1), larynx (31·6%; 25·7–36·7), uterine (31·5%; 17·8–40·0), and stomach cancer (31·4%; 23·7–37·3). Among females, breast, cervical, and stomach cancer were responsible for the highest DALYs in 2016. The highest cancer DALYs among males in India in 2016 were due to lung cancer, followed by lip and oral cavity cancer, other pharynx cancer, and stomach cancer (figure 3).

**Figure 5:**
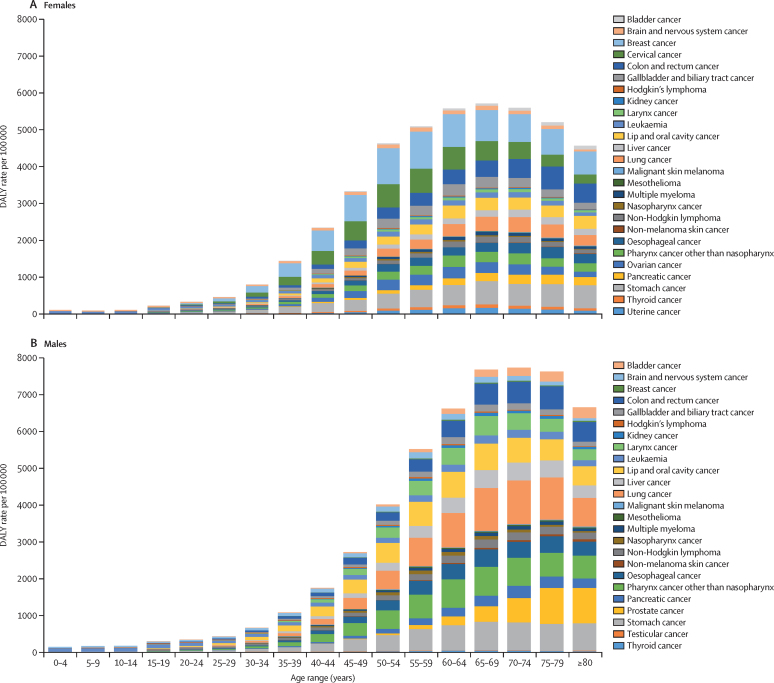
Age-specific DALYs for different types of cancers by sex in India, 2016 DALYs=disability-adjusted life-years.

Among both boys and girls aged 0–14 years, leukaemia was responsible for the highest DALYs in India in 2016, followed by brain and nervous system cancer (figure 5, appendix pp 34–40). Breast and cervical cancer DALY rates in females increased after the age of 30 years and dropped after the age of 60 years in 2016. DALY rates for lung cancer and stomach cancer increased in males after the age of 35 years in 2016, while prostate cancer increased after the age of 50 years.

Tobacco use, alcohol use, and dietary risks were estimated by GBD to contribute the highest cancer DALYs in 2016; they were responsible for 10·9%, 6·6% and 6·0% of the total cancer DALYs, respectively (appendix pp 41–42).

### Stomach cancer

**Figure 6:**
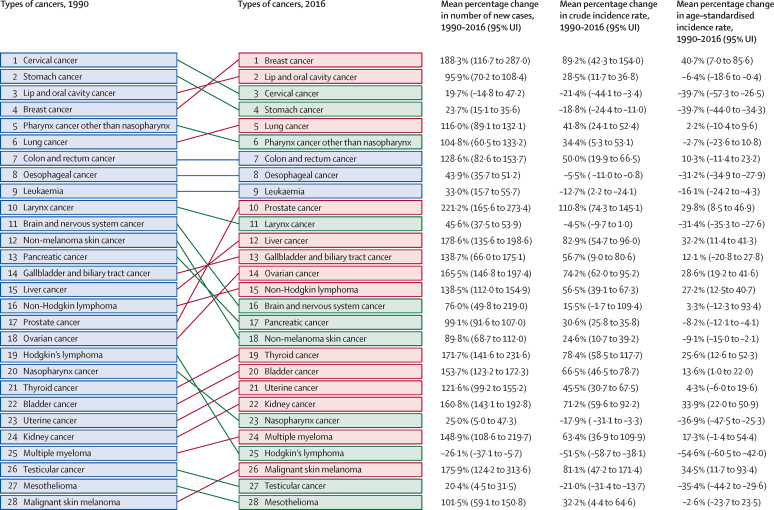
Change in incidence rate of different types of cancers in India, 1990–2016

**Figure 7:**
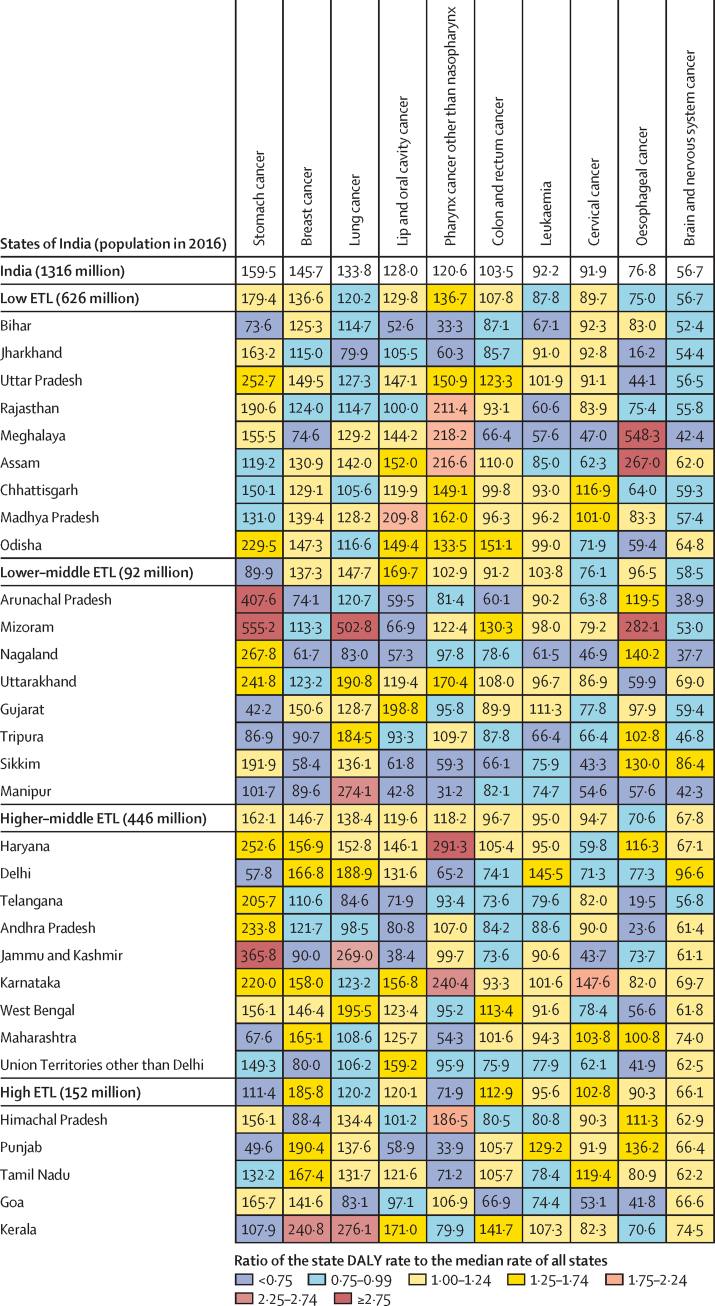
Crude DALY rates in the states of India for the ten cancers responsible for the highest DALYs in India, 2016 DALY is presented as rate per 100 000. DALY=disability-adjusted life-year. ETL=epidemiological transition level.

**Figure 8:**
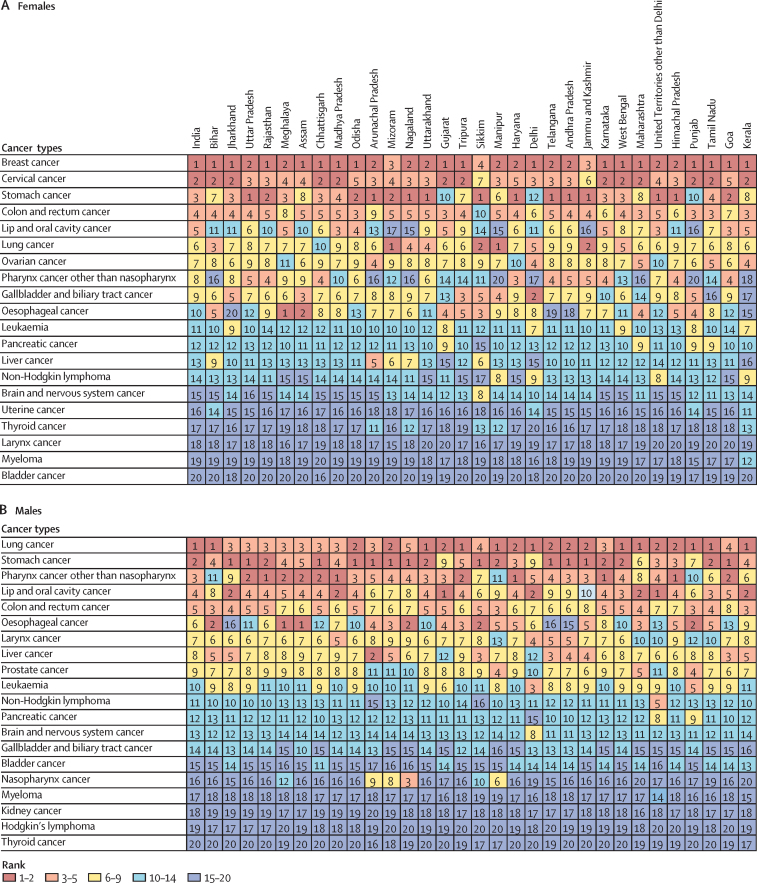
Ranking of crude death rates in each state of India for the 20 cancers causing the highest number of deaths by sex in India, 2016 The sequence of the states is from the lowest to the highest epidemiological transition level in 2016.

The estimated number of incident stomach cancer cases in India in 2016 was 75 000 (95% UI 73 000–78 000) and the prevalent cases were 112 000 (109 000–116 000; appendix p 31). There was a substantial reduction in the age-standardised incidence rate of stomach cancer (39·7%; 95% UI 34·3–44·0) from 1990 to 2016 in India (figure 6). Stomach cancer was the fourth leading incident cancer in females and males in India in 2016 (appendix pp 49, 50). The age-standardised incidence rate for stomach cancer in 2016 varied 11·5 times across the states of India (appendix pp 43–48). Stomach cancer is the only cancer type for which decreasing rates were estimated in all states across the country; a decrease greater than 50% was estimated in Uttarakhand, Sikkim, Delhi, Jammu and Kashmir, and Himachal Pradesh among females, and in Maharashtra among males (appendix pp 51–54). There was a 13·1 times difference between the highest and lowest state-specific DALY rates for stomach cancer (figure 7). The crude DALY rate was highest in Mizoram, Arunachal Pradesh, and Jammu and Kashmir. In 2016, stomach cancer was the first or second highest cause of cancer deaths in 15 states for females and in 18 states for males (figure 8). Only a small proportion of the stomach cancer DALYs in India in 2016 could be attributed to risk factors included in GBD (dietary risks [4·1%] and smoking [3·5%]; appendix pp 41–42).

### Breast cancer

The estimated number of incident breast cancer cases in India in 2016 was 118 000 (95% UI 107 000–130 000), 98·1% of which were in females, and the prevalent cases were 526 000 (474 000–574 000; appendix p 31). Breast cancer is the leading cancer in Indian females, accounting for the largest crude incidence rate and prevalence of any cancer type (appendix pp 49, 50). Over the 26-year period, the age-standardised incidence rate of breast cancer in females increased by 39·1% (95% UI 5·1–85·5) from 1990 to 2016, with increase observed in every state of the country (appendix pp 51). The age-standardised incidence rate of breast cancer varied 3·2 times in females across the states of India in 2016 (appendix pp 43–48). The crude incidence and DALY rates of breast cancer were highest in the high ETL state group (figure 7). There was a 4·1 times difference between the highest and lowest state-specific DALY rates for breast cancer in 2016. The highest crude DALY rates for breast cancer were in Kerala, Punjab, and Tamil Nadu in the high ETL state group, followed by Delhi, Maharashtra, Karnataka, and Haryana in the higher-middle ETL group. Breast cancer was the first or second leading cause of cancer deaths among females in 28 Indian states in 2016 (figure 8). Only a small proportion of breast cancer DALYs in India in 2016 could be attributed to risk factors included in GBD (high fasting plasma glucose [4·9%] and secondhand smoke [2·4%]; appendix pp 41–42).

### Lung cancer

We refer to tracheal, bronchus, and lung cancer as lung cancer in this report for simplicity. The number of incident lung cancer cases in India in 2016 was 67 000 (95% UI 63 000–72 000), 72·2% of which were in males, and the prevalent cases were 74 000 (70 000–80 000; appendix p 31). This cancer was the second most common incident cancer among males in 2016 (appendix pp 49–50). The age-standardised incidence rate of lung cancer varied 8 times in both sexes combined across the states of India in 2016 (appendix pp 43–48). The crude lung cancer incidence rate in males was highest in Kerala and Mizoram, and in females was highest in Mizoram and Manipur (appendix pp 49, 50). There was a 6·3 times difference between the highest and lowest state-specific crude DALY rates for lung cancer in 2016 (figure 7). The crude DALY rate for lung cancer in 2016 was highest in Mizoram, followed by Kerala, Manipur, and Jammu and Kashmir. Lung cancer was the first or second leading cause of cancer deaths in 19 states for males and four states for females in 2016 (figure 8). Tobacco use and air pollution were the leading risk factors in GBD for lung cancer in India in 2016 to which 43·2% and 43·0% of the lung cancer DALYs could be attributed, respectively (appendix pp 41, 42).

### Lip and oral cavity cancer

The number of incident lip and oral cavity cancer cases in India in 2016 was 113 000 (95% UI 106 000–118 000) and the prevalent cases were 397 000 (371 000–412 000; appendix p 31). There was a substantial reduction in the age-standardised incidence rate of lip and oral cavity cancer (6·4%; 95% UI 0·4–18·6) from 1990 to 2016 in India (figure 6). Lip and oral cavity cancer was the most common incident cancer in males in India in 2016 (appendix p 50). The crude incidence rate was substantially higher in males than in females (appendix pp 49, 50). The age-standardised incidence rate for lip and oral cavity cancer varied 5·1 times among both sexes combined across the states of India in 2016 (appendix pp 43–48). The crude incidence and DALY rates of lip and oral cavity cancer were highest in the lower-middle ETL state group (figure 7). There was a 5·5 times difference between the highest and lowest state-specific DALY rates for lip and oral cavity cancer in 2016. The highest DALY rate for lip and oral cavity cancer was in Madhya Pradesh, followed by Gujarat and Kerala. Lip and oral cavity cancer was the first or second leading cause of cancer deaths in seven Indian states for males in 2016 (figure 8). Smokeless tobacco, alcohol use and smoking were the leading risk factors in GBD for lip and oral cavity cancer in India in 2016 to which 33·2%, 29·8%, and 20·9% of the lip and oral cavity cancer DALYs could be attributed, respectively (appendix pp 41, 42).

### Pharynx cancer other than nasopharynx

The number of incident pharynx cancer other than nasopharynx cases in India in 2016 was 65 000 (95% UI 58 000–70 000), 70·2% of which were in males, and the prevalent cases were 152 000 (137 000–163 000; appendix p 31). The age-standardised incidence rate for other pharynx cancer varied 9·8 times for both sexes combined across the states of India in 2016 (appendix pp 43–48). The DALY rate for other pharynx cancer was higher in the low ETL state group than the other ETL groups (figure 7). There was a 9·3 times difference between the highest and lowest state-specific DALY rates for other pharynx cancer in 2016. The DALY rate for other pharynx cancer was highest in Haryana, followed by Karnataka, Meghalaya, Assam, Rajasthan, and Himachal Pradesh. Other pharynx cancer was the first or second leading cause of cancer deaths among males in 11 Indian states in 2016 (figure 8). Alcohol use was the leading risk factor in GBD for other pharynx cancer in India in 2016 to which 30.1% of the other pharynx cancer DALYs could be attributed (appendix pp 41, 42).

### Colon and rectum cancer

The number of incident colon and rectum cancer cases in India in 2016 was 63 000 (95% UI 58 000–66 000) and the prevalent cases were 185 000 (171 000–195 000; appendix p 31). Colon and rectum cancer incidence rate in both sexes was higher in the high ETL as compared with other ETL state groups (appendix pp 49, 50). The age-standardised incidence rate for colon and rectum cancer varied 1·9 times across the states of India in 2016 (appendix pp 43–48). There was a 2·5 times difference between the highest and lowest state-specific DALY rates for colon and rectum cancer in 2016 (figure 7). The highest DALY rate for colon and rectum cancer was in Odisha, followed by Kerala, Mizoram, and Uttar Pradesh. Colon and rectum cancer was the third to fifth leading cause of cancer deaths in 24 states for females and 16 states for males (figure 8). Dietary risks were the leading risk factor in GBD for colon and rectum cancer in India in 2016 to which 43·2% of the colon and rectum cancer DALYs could be attributed (appendix pp 41, 42).

### Leukaemia

The number of incident leukaemia cancer cases in India in 2016 was 34 000 (95% UI 30 000–38 000) and the prevalent cases were 105 000 (96 000–120 000; appendix p 31). The age-standardised incidence rate of leukaemia decreased substantially by 16·1% (95% UI 4·3–24·2) in India from 1990 to 2016 (figure 6). Among children aged 0–14 years, leukaemia was responsible for the highest proportion of the cancer DALYs (34·6%) in India in 2016, which was similar among boys and girls (figure 5, appendix pp 34–40). The age-standardised incidence rate for leukaemia varied 2·2 times across the states of India in 2016 (appendix pp 43–48). The crude DALY rate for leukaemia was similar across the ETL state groups in 2016 (figure 7). The states with the highest DALY rate for leukaemia were Delhi and Punjab. There was a 2·5 times difference between the highest and lowest state-specific DALY rates for leukaemia. In males, leukaemia was the third and fifth leading cause of cancer deaths in Delhi and Punjab respectively in 2016, but lower in other states (figure 8).

### Cervical cancer

Cervical cancer was the second most common cancer in females in India in 2016, with 77 000 (95% UI 68 000–96 000) incident cervical cancer cases in India in 2016 and 288 000 (247 000–342 000) prevalent cases (appendix p 31). The age-standardised incidence rate of cervical cancer decreased substantially by 39·7% (95% UI 26·5–57·3) in India from 1990 to 2016 (figure 6). The age-standardised incidence rate for cervical cancer varied 2·8 times in females across the states of India in 2016 (appendix pp 43–48). There was a 3·4 times difference between the highest and lowest state-specific DALY rates for cervical cancer (figure 7). The DALY rate for cervical cancer in 2016 was highest in Karnataka, followed by Tamil Nadu, Chhattisgarh, Madhya Pradesh, and Maharashtra. Cervical cancer was the second leading cause of cancer deaths for females in 12 Indian states (figure 8). Unsafe sex was estimated to contribute to all of the cervical cancer DALYs in GBD in India in 2016 (appendix pp 41, 42). It is important to note that other unknown risk factors could also be contributing to cervical cancer as the population-attributable fractions of different risks can add up to more than 100%.

### Oesophageal cancer

The number of incident oesophageal cancer cases in India in 2016 was 37 000 (95% UI 36 000–38 000), 60·8% of which were in males, and the prevalent cases were 41 000 (39 000–42 000) (appendix p 31). There was a substantial reduction in the age-standardised incidence rate of oesophageal cancer (31·2%; 95% UI 27·9–34·9) in India from 1990 to 2016 (figure 6). The age-standardised incidence rate for oesophageal cancer varied 34 times across the states of India in 2016 (appendix pp 43–48). There was a 33·8 times difference between the highest and lowest state-specific DALY rates for oesophageal cancer (figure 7). The DALY rate for oesophageal cancer was highest in the north-east states of Meghalaya, Assam, Mizoram, and Nagaland. Oesophageal cancer was the first or second leading cause of cancer deaths in two states for females and six states for males (figure 8). Smokeless tobacco, dietary risks, and smoking were the leading risk factors in GBD for oesophageal cancer in India in 2016 to which 22·6%, 21·5%, and 17·4% of the oesophageal cancer DALYs could be attributed, respectively (appendix pp 41, 42).

### Brain and nervous system cancer

The number of incident brain and nervous system cancer cases in India in 2016 was 23 000 (95% UI 21 000–28 000) and the prevalent cases were 49 000 (44 000–57 000; appendix p 31). The age-standardised incidence rate for brain and nervous system cancer varied 2·1 times across the states of India in 2016 (appendix pp 43–48). Among children aged 0–14 years, brain and nervous system cancer was responsible for the second highest proportion of the cancer DALYs (16%) in India in 2016, which was similar among boys and girls (figure 5, appendix pp 34–40). There was a 2·6 times difference between the highest and lowest state-specific DALY rates for brain and nervous system cancer (figure 7). The DALY rate for brain and nervous system cancer was highest in Delhi followed by Sikkim. The ranking of deaths due to brain and nervous system cancer was relatively low in all states as compared with the other high burden cancers (figure 8).

### Prostate cancer

Prostate cancer had the fifth highest incidence rate among males in India in 2016 (4·8 per 100 000, 95% UI 3·8–5·8), with 33 000 (26 000–40 000) incident cases and 112 000 (87 000–137 000) prevalent cases (appendix pp 31, 50). The age-standardised incidence rate of prostate cancer increased substantially by 29·8% (95% UI 8·5–46·9) from 1990 to 2016 (figure 6). This rate varied 2·4 times across the states of India in 2016 (appendix pp 43–48). The crude incidence rate for prostate cancer was highest in Kerala (appendix p 50).

### Larynx cancer

Larynx cancer had the seventh highest incidence rate among males in India in 2016 (3·8 per 100 000, 95% UI 3·7–4·0). There were 31 000 (95% UI 30 000–33 000) incident cases in India, of which 83·5% were in males, and there were 96 000 (93 000–100 000) prevalent cases (appendix pp 31, 50). The age-standardised incidence rate of larynx cancer in India decreased significantly by 31·4% (95% UI 27·6–35·3) from 1990 to 2016 (figure 6). This rate varied 3·3 times across the states of India in 2016 (appendix pp 43–48). The crude incidence rate for larynx cancer in males was highest in Kerala in 2016, followed by Delhi, Haryana, and Assam (appendix p 50). Smoking and alcohol use were the leading risk factors in GBD for larynx cancer in India in 2016 to which 37·9% and 17·2% of the larynx cancer DALYs could be attributed, respectively (appendix pp 41, 42).

### Liver cancer

Liver cancer had the ninth highest incidence rate among males in 2016 in India (3·1 per 100 000, 95% UI 2·9– 3·2). There were 30 000 (95% UI 29 000–32 000) incident cases in India, of which 68·9% were in males, and 12 000 (11 000–14 000) prevalent cases (appendix pp 31, 50). The age-standardised incidence rate of liver cancer increased substantially by 32·2% (95% UI 11·4–41·3) from 1990 to 2016 (figure 6). This rate varied 7·9 times across the states of India in 2016 (appendix pp 43–48). The crude incidence rate for liver cancer in males in 2016 was highest in Arunachal Pradesh, followed by Kerala, Sikkim, and Mizoram (appendix p 50). In 2016, of the total liver cancer DALYs in India, 11·7% could be attributable to alcohol use in GBD (appendix pp 41, 42).

### Ovarian cancer

Ovarian cancer had the sixth highest incidence rate among females in 2016 in India (4·0 per 100 000, 95% UI 3·7–4·3), with 26 000 (95% UI 24 000–27 000) incident cases and 76 000 (69 000–80 000) prevalent cases (appendix pp 31, 49). The age-standardised incidence rate of ovarian cancer increased substantially by 28·6% (95% UI 19·2–41·6) from 1990 to 2016 (figure 6). This rate varied 3·7 times across the states of India in 2016 (appendix pp 43–48). The crude incidence rate was highest in Kerala, followed by Delhi, Arunachal Pradesh, and Punjab (appendix p 49). Only a small proportion of the ovarian cancer DALYs in India in 2016 could be attributed to risk factors included in GBD (high fasting plasma glucose [5·5%]; appendix pp 41, 42).

### Gallbladder and biliary tract cancer

Gallbladder and biliary tract cancer had the ninth highest incidence rate among females in 2016 in India (2·6 per 100 000, 95% UI 2·3–2·8). There were 26 000 (95% UI 23 000–29 000) incident cases in India, of which 64·4% were in females, and there were 21 000 (18 000–23 000) prevalent cases (appendix pp 31, 49). This rate varied 5·9 times across the states of India in 2016. The crude incidence of gallbladder and biliary tract cancer in females was highest in the states of Assam and Delhi in 2016 (appendix p 49). 9·1% of the gallbladder and biliary tract cancer DALYs in India in 2016 could be attributed to high body-mass index in GBD (appendix pp 41, 42).

### Thyroid cancer

Thyroid cancer had the tenth highest incidence rate among females in 2016 in India (2·5 per 100 000, 95% UI 2·3–2·6). There were 21 000 (95% UI 20 000–23 000) incident cases in India, of which 74·3% were in females, and there were 106 000 (101  000–115 000) prevalent cases (appendix pp 31, 49). The age-standardised incidence rate of thyroid cancer increased substantially by 25·6% (95% UI 12·6–52·3) from 1990 to 2016 (figure 6). This rate varied 6·1 times across the states of India in 2016 (appendix pp 43–48). The crude incidence rate of thyroid cancer was highest in females in Kerala, followed by Sikkim, Nagaland, and Goa in 2016 (appendix p 49). Only a small proportion of the thyroid cancer DALYs in India in 2016 could be attributed to risk factors included in GBD (high body-mass index [5·1%]; appendix pp 41, 42).

## Discussion

The number of new cases and deaths due to cancer doubled in India from 1990 to 2016, as did the proportional contribution of cancers to the total DALYs and deaths in the country. The crude incidence, mortality, and DALYs from cancer increased substantially over the 26-year time period. However, there was no change in the age-standardised rates for both sexes combined, highlighting the contribution of ageing and population growth to the increasing cancer burden of the country. The age-standardised death rate for cancer increased for males during this period, suggesting differences by sex. Males had higher MI ratios than females in every state of the country.

The trends observed in sex-specific and cancer type-specific incidence rates over time in India are likely due to a variety of factors, such as population ageing, changes in cancer literacy, detection, health-care access, and a variety of risk factors. We highlight some of the key risk factors that are associated with the highest burden of cancers in India. The substantial decrease in the age-standardised incidence rate of stomach cancer across the country might be due to lifestyle changes such as reduced consumption of salt-preserved foods, better availability of refrigeration, and increasing fruit consumption, and to decreases in smoking prevalence.^32^, ^33^, ^34^ The prevalence of *Helicobacter pylori* remains persistently high in Indians,^35^ and hence this is an unlikely factor in the decreasing incidence of stomach cancer. For breast cancer, a substantial increase in age-standardised incidence rate is consistent with changes in some risk factors over time in India, such as later age at first birth, lower parity, and increase in overweight and obesity.^32^, ^36^, ^37^ The substantial decrease in the age-standardised incidence rate of oesophageal cancer might be partly due to the decrease in smoking prevalence over the 26-year period and in smokeless tobacco use over the past 10 years.^32^, ^34^ The absence of change in the age-standardised incidence rate of lung cancer in India might be related to the mixed trends of its major risk factors, which include decrease in smoking and household air pollution but an increase in ambient air pollution, but also due to the patterns of other unknown risk factors.^32^, ^34^ The small decrease in the age-standardised incidence rate of lip and oral cavity cancer in India could be related to the reduction in use of smokeless tobacco in India during the past decade.^32^ The decreasing age-standardised incidence rate of cervical cancer could be inversely related to the reproductive risk factors mentioned for breast cancer increase above.^36^ In the absence of systematic human papilloma virus (HPV) testing in India, there is no evidence of changing seroprevalence of HPV and its subtypes over time in India. Leukaemia has been associated with genetic, infectious, and environmental risks,^38^ but the reasons for the substantial reduction in its age-standardised incidence rate in India from 1990 to 2016 are unclear.

The interplay of the trends of the two risk factors to which the highest proportion of cancer DALYs in India could be attributed, tobacco and alcohol, is interesting in relation to the trends of some of the leading cancers. Both of these risks contribute to a variable extent to lip and oral cavity, oesophageal, larynx, and liver cancers. The age-standardised incidence rate of the first three of these cancers decreased in India from 1990 to 2016, and that of liver increased. While tobacco use in India has decreased during this period, alcohol use has increased.^32^ The drop in incidence rate of lip and oral cavity, oesophageal, and larynx cancers could be partly related to a larger influence of tobacco than alcohol on these cancers, and the increase in incidence rate of liver cancer could be partly related to a larger influence of alcohol than tobacco on this cancer, in addition to the trends of yet unknown other risk factors contributing to all of these cancers. Collaborative multi-institutional research efforts on cancer risk factors can help address such knowledge gaps, as well as lead to a better understanding of the reasons for the substantial decreases or increases in the incidence of different types of cancers in different parts of India. Detailed decomposition analyses are needed to tease apart the contribution of population structure changes, risk factors, interventions, and other determinants to the trends of leading cancers in India.

The heterogeneity of the incidence rate of different types of cancers across India is vast. Major variations exist even within the same geographical region, such as neighbouring states in the northeast—for example, a 15 times difference in age-standardised incidence rates of nasopharynx cancer between the neighbouring north-eastern states of Nagaland and Tripura. Examples of the heterogeneous distribution of important risk factors and the corresponding distribution of associated cancers are also insightful. The states in the northeast of India generally have high tobacco use as well as a high incidence of lung, oesophageal, nasopharynx and other pharynx cancers that are associated with tobacco use. There are also unique tobacco consumption patterns in these states, such as use of tobacco-infused water in Mizoram.^39^, ^40^ HPV and cervical cancer are both high in Dindigul in Tamil Nadu,^41^ consumption of smoked or preserved meats and stomach cancer are high in Mizoram,^42^ and delayed childbearing and lower parity are high in Kerala as is breast cancer.^43^ The many variations between the states indicate the need for state-specific approaches for cancer control. If the reasons for the heterogeneous distribution of the major cancer types in different parts of India are understood better through large-scale collaborative research, this knowledge could help plan more specific efforts to reduce the cancer burden across the states of India.

The National Cancer Control Programme was initiated by the Government of India in 1975 to equip tertiary care cancer hospitals and institutions to implement systematic, equitable, and evidence-based strategies for prevention, early detection, diagnosis, treatment, and palliation, using available resources.^44^ State cancer institutes and tertiary care cancer centres have been established under this programme that are responsible for improved cancer awareness and management at the state level.^45^ Despite these attempts, access to critical cancer treatment is low in the country. For example, availability of radiotherapy machines is poor, there are delays in treatment, and there is geographic inequity in the distribution of such resources.^10^, ^11^, ^46^ With the launch of the National Programme for Control of Cancer, Diabetes, CVD and Stroke in 2010 in India, the cancer control efforts are now part of this umbrella programme for non-communicable diseases.^47^ The national programme aims to tackle cancer by addressing preventable common risk factors through community-level, cost-effective screening for high-burden cancers, which include clinical breast examination for breast cancer, visual inspection with acetic acid for cervical cancer and visual examination for oral cancers.^14^ However, there are many challenges with these efforts, including difficulties with trained human resources, follow-up of positive tests, timely diagnosis, and well-tracked referral pathways.^48^ Additionally, there are limited population-level screening modalities available for some of the cancers responsible for the highest cancer burden in India, such as stomach and lung cancers. Primary prevention should therefore be promoted for these cancers, which can be guided by the heterogeneity between the states in this report. For secondary prevention, less invasive tests for *H pylori* may offer cost-effective first-line tests for referrals to more invasive endoscopic tests for early detection of stomach cancer.^49^ Faecal occult blood testing as a non-invasive, cost-effective approach to screen for colorectal cancer should also be considered.^50^

Ideally, national and state-level efforts should coordinate to facilitate the development of a prevention-to-palliation system of upward referral for early confirmatory diagnosis and prompt treatment of cancers, and downward referral for adequate follow-up, including palliative care and pain relief. The experience of some states can be useful to develop such strategies. For example, the Tamil Nadu Health Systems Project paved the way for lessons on breast and cervical cancer screening, from which the importance of community awareness, referral pathways, health management information systems, and trained human resources emerged as critical factors for a successful screening programme.^48^, ^51^ Shortage of appropriately educated and trained medical officers for treatment, management, and palliative care for cancer patients, particularly outside metropolitan cities, remains a challenge across the country.^10^ Recent attempts to address this gap include training through the Extension of Community Healthcare Outcomes tele-mentoring model for primary health-care providers and specialists on cancer screening and management by the National Institute of Cancer Prevention and Research.^52^ Attempts are also being made to strengthen tertiary cancer management by developing evidence-based guidelines through the knowledge exchange platform of the National Cancer Grid of India.^12^ Placing the India cancer trends in the global context, the overall age-standardised incidence rate of cancers has been stable in India during the past quarter century, but it has increased in the other BRICS countries (Brazil, Russia, China and South Africa) where the rate is currently about double that in India.^53^ India should avoid the increasing trends observed in these countries by establishing adequate preventive measures that are consistent with the heterogeneity of cancer distribution in different parts of the country.

Cancer patients in India incur heavy out-of-pocket expenditures.^11^, ^54^ The insurance programmes in some states and a previous national insurance scheme have attempted to minimise the impact of this on households.^55^, ^56^, ^57^ The recently announced National Health Protection Mission (Ayushman Bharat), which aims to provide substantial health insurance coverage for 500 million people from low-income households, has the potential to reduce the economic burden of cancer and other non-communicable diseases at the population level across the country.^58^ It would be useful to ensure that all relevant aspects of cancer care are included in this health protection scheme.

This report provides a comprehensive descriptive epidemiology of cancer and its heterogeneity across all states of India from 1990 to 2016. India pioneered the establishment and expansion of the National Cancer Registry Programme (NCRP) under the Indian Council of Medical Research through a network of cancer registries during the past 30 years, which now covers 23 states and union territories.^23^ The GBD methods rely substantially on the NCRP data from India, but they also use data from all accessible data sources, including some registries that are not part of the NCRP, cause of death data from the SRS and other sources, and a variety of covariates to arrive at the best possible estimates for states where registries do not exist. Accordingly, differences are expected between the GBD estimates and the statistics reported by the NCRP, which are based entirely on population-based registries.^23^ The objectives and advantages of the GBD and NCRP approaches are complementary. While the NCRP adheres to a standardised methodology established by WHO's International Agency for Research in Cancer, the GBD methodology provides a standardised approach that also incorporates other sources of data for estimations for all states of India, including those where registries do not exist such as the populous states of Bihar, Chhattisgarh, Jharkhand, and Uttar Pradesh. The GBD methodology is integrated into a larger total disease framework estimation process that allows comparison of the cancer burden to that of other diseases, which has helped to highlight the increasing contribution of cancers to the disease burden in India over the past quarter century. The close collaboration between the NCRP and GBD has made the findings in this report possible. It has also further highlighted the need to establish cancer registries in large states of north India where none exist, as well as adequate coverage of rural populations.

The limitations of the findings in this report include the general limitations of the GBD approach that are described elsewhere.^1^, ^25^, ^26^, ^27^, ^28^ Input data used to generate cancer mortality can be biased in multiple ways.^1^ A high proportion of ill-defined cancer cases in the registry data or ill-defined causes of death in mortality data sources require redistribution of these cases, which can introduce bias. Underreporting of cancers that require advanced diagnostic techniques (eg, leukaemia, brain, pancreatic, and liver cancer) can be an issue in data from areas where access to these technologies is scarce. Conversely, misclassification of metastatic sites as primary cancer can lead to overestimation of cancer sites that are common sites for metastases like the brain or liver. In addition, risk factors for breast and cervical cancer that are related to reproductive history in women, such as age at first birth and parity, are not yet included in the GBD risk factor assessment. A specific limitation for India is an inadequate cause of death reporting as part of the vital registration system, which reports medically certified cause of death only for a small proportion of deaths in India so far, with variable coverage across the states. The SRS provides cause of death data using verbal autopsy for all states in India, which is a reasonable alternative data source when the cause-specific data are not fully available from vital registration systems.^4^ However, this situation highlights the need to improve vital registration and improve training to code cause of death across India. The absence of population-based registries in major populous states of north India such as Bihar and Uttar Pradesh leaves open questions about the magnitude of some cancers such as gallbladder cancer, which are expected by the India cancer community to be high there, but this cannot be substantiated yet because of the paucity of population-level evidence. Even in the existing registries, the mortality data are variable and need to be strengthened in order to arrive at more robust estimates of MI ratios. The very sparse presence of rural cancer registries in India is also a limitation. In cancer data-scarce locations, the estimates for the types of cancer were calculated using covariates, which are explanatory variables with a causal relation to cancer incidence and mortality.^1^ The strengths of the findings presented in this report were the use of standardised GBD methodology and inclusion of all accessible data from multiple sources, and the substantial contribution of a large network of cancer experts from India in the analysis and interpretation of the findings.

In conclusion, the detailed epidemiology of 28 types of cancer in every state of India over a quarter century described in this report highlights the substantial variations between the states for the different types of cancer, and can serve as a useful reference for more targeted planning of cancer control that is commensurate with the trends of different cancers in each state of India. The increasing overall contribution of cancer to disease burden in India should motivate more systematic and large-scale approaches to reduce this burden at the population level across the country. These efforts should include improved human resources and infrastructure for prevention, screening, treatment, and palliative care for cancers, as well as adequate financial protection for cancer care. These approaches should focus at least on the ten cancers that contribute the highest DALYs in India—ie, stomach, breast, lung, lip and oral cavity, pharynx other than nasopharynx, colon and rectum, leukaemia, cervical, oesophageal, and brain and nervous system cancers. Other types of cancer should also be addressed in India as circumstances and resources allow.
