# Correction to *Lancet Oncol* 2018; 19: 1289–306

**DOI:** 10.1016/S1470-2045(18)30748-4

**Published:** 2018-11

*India State-Level Disease Burden Initiative Cancer Collaborators. The burden of cancers and their variations across the states of India: the Global Burden of Disease Study 1990–2016.* Lancet Oncol *2018;* 19: *1289–306*—The appendix of this Article has been updated. The correction has been made to the online version as of Oct 3, 2018.

