# Supplementary material for: The burden of cancers and their variations across the states of India: the Global Burden of Disease Study 1990–2016
Source: Lancet Oncol. 2018 Oct;19(10):1289–306. doi: 10.1016/S1470-2045(18)30447-9 (PMC6167407; doi:10.1016/S1470-2045(18)30447-9)
Supplement: Supplementary appendix [file mmc1.pdf]

THE LANCET
Oncology

Supplementary appendix

This appendix formed part of the original submission and has been peer reviewed. We post it as supplied by the authors.

Supplement to: India State-Level Disease Burden Initiative Cancer Collaborators. The burden of cancers and their variations across the states of India: the Global Burden of Disease Study 1990–2016. *Lancet Oncol* 2018; published online Sept 10. http://dx.doi.org/10.1016/S1470-2045(18)30447-9.

The burden of cancers and their variations across the states of India: the Global Burden of Disease Study 1990-2016

India State-Level Disease Burden Initiative Cancer Collaborators

Web Appendix

Correspondence to: Prof. Lalit Dandona, lalit.dandona@phfi.org

Table of Contents

1. GBD 2016 cancer burden estimation methods ... 3
2. GBD 2016 India data inputs for cancer mortality, morbidity, risk factors, and covariates ... 17
3. Burden of all cancers together in the states of India, 2016 ... 30
4. Incident cases, prevalent cases, and deaths for different types of cancers in India by sex, 2016 ... 31
5. Change in incidence and death rate for all cancers in the states of India grouped by epidemiological transition level, 1990 to 2016 ... 32
6. Crude annual death rate of all cancers together in the states of India, 1990 and 2016 ... 33
7. Age-specific DALY rate for different types of cancers in the states of India by sex, 2016 ... 34
8. Proportion of total DALYs for leading cancers in India that are attributable to GBD risk factors, 2016 ... 41
9. Risk factors contributing to cancer DALYs in India, 2016 ... 42
10. Age-standardised incidence rate for different types of cancers by sex in the states of India, 2016 ... 43
11. Crude incidence rate of the ten leading incident cancers in the states of India by sex, 2016 ... 49
12. Percent change of age-standardised incidence for different types of cancers in the states of India by sex, 1990 to 2016… ... 51

1. GBD 2016 cancer burden estimation methods

Adapted from: GBD 2016 Cancer Collaborators. Global, regional, and national cancer incidence, mortality, years of life lost, years lived with disability, and disability-adjusted life-years for 29 cancer groups, 1990 to 2016: a systematic analysis for the Global Burden of Disease study. JAMA Oncol 2018; Epub 2 June, 2018.

The GBD cause list is organised in a hierarchy. Levels 1 and 2 represent general groupings. The broad group "neoplasms." which includes all cancer causes, is at level 2 under the level 1 group non-communicable diseases. Level 3 includes 29 cancer groups, and level 4 includes 37 groups since in level 4, leukaemia, liver cancer, and non-melanoma skin cancer are further subdivided.

A. List of ICD codes mapped to the GBD cause list

The GBD Study 2016 uses codes from the 9th and 10th revisions of the International Statistical Classification of Diseases and Related Health Problems (ICD) pertaining to cancer (140-209) and ICD10 (C00-C96) except for Kaposi sarcoma (ICD10: C46).

The ICD codes used for cancer incidence data are listed below:

**Cause**	**ICD10**	**ICD9**
Lip and oral cavity cancer	C00-C07, C08-C08.9, Z85.81-Z85.810	140-145.9, V76.42
Nasopharynx cancer	C11-C11.9	147-147.9
Other pharynx cancer	C09-C10.9, C12-C13.9	146-146.9, 148-148.9
Oesophageal cancer	C15-C15.9, Z85.01	150-150.9
Stomach cancer	C16-C16.9, Z12.0, Z85.02-Z85.028	151-151.9, 209.23, V10.04
Colon and rectum cancer	C18-C19.0, C20, C21-C21.8, Z12.1-Z12.13, Z85.03-Z85.048, Z86.010	153-154.9, 209.1-209.17, V10.05-V10.06, V76.41, V76.5-V76.52
Liver cancer	C22-C22.4, C22.7-C22.9, Z85.05	155-155.9, V10.07
Gallbladder and biliary tract cancer	C23, C24-C24.9	156-156.9
Pancreatic cancer	C25-C25.9, Z85.07	157-157.9
Larynx cancer	C32-C32.9, Z85.21	161-161.9, V10.21
Tracheal, bronchus, and lung cancer	C33, C34-C34.92, Z12.2, Z80.1-Z80.2, Z85.1-Z85.20	162-162.9, 209.21, V10.1-V10.20, V16.1-V16.2, V16.4-V16.40
Malignant skin melanoma	C43-C43.9, Z85.82-Z85.828	172-172.9
Non-melanoma skin cancer	C44-C44.99	173-173.99, 216-216.9, 232-232.9
Non-melanoma skin cancer (squamous-cell carcinoma)	C44.02, C44.12-C44.129, C44.22-C44.229, C44.32-C44.329, C44.42, C44.52-C44.529, C44.62-C44.629, C44.72-C44.729, C44.82, C44.92	173.02, 173.12, 173.22, 173.32, 173.42, 173.52, 173.62, 173.72, 173.82, 173.92
Non-melanoma skin cancer (basal-cell carcinoma)	C44.01, C44.11-C44.119, C44.21-C44.219, C44.31-C44.319, C44.41, C44.51-C44.519, C44.61-C44.619, C44.71-C44.719, C44.81, C44.91	173.01, 173.11, 173.21, 173.31, 173.41, 173.51, 173.60-173.61, 173.71, 173.81, 173.91
Breast cancer	C50-C50.629, C50.8-C50.929, Z12.3-Z12.39, Z80.3, Z85.3, Z86.000	174-175.9, V10.3, V16.3
Cervical cancer	C53-C53.9, Z12.4, Z85.41	180-180.9, V10.41, V72.32

Uterine cancer	C54-C54.3, C54.8-C54.9, Z85.42, Z86.001	182-182.9
Ovarian cancer	C56-C56.2, C56.9, Z80.41, Z85.43	183-183.0, 183.8-183.9, V10.43, V16.41
Prostate cancer	C61-C61.9, Z12.5, Z80.42, Z85.46	185-185.9, V10.46, V16.42, V76.44
Testicular cancer	C62-C62.92, Z80.43, Z85.47-Z85.48	186-186.9, V10.47-V10.48, V16.43
Kidney cancer	C64-C64.2, C64.9-C65.9, Z80.51, Z85.52-Z85.54	189-189.1, 189.5-189.6, 209.24
Bladder cancer	C67-C67.9, Z12.6-Z12.79, Z80.52, Z85.51	188-188.9, V10.51, V16.52, V76.3
Brain and nervous system cancer	C70-C70.1, C70.9-C72.9, Z85.841-Z85.848, Z86.011	191-191.9
Thyroid cancer	C73, Z85.850	193-193.9
Mesothelioma	C45-C45.2, C45.7, C45.9	
Hodgkin's lymphoma	C81-C81.49, C81.7-C81.79, C81.9-C81.99, Z85.71-Z85.72	201-201.98, V10.72
Non-Hodgkin lymphoma	C82-C85.29, C85.7-C86.6, C96-C96.9	200-200.9, 202-202.98
Multiple myeloma	C88-C90.32	203-203.9
Leukaemia	C91-C93.7, C93.9-C95.2, C95.7-C95.92, Z80.6, Z85.6	204-208.92, V10.59-V10.69, V16.6
Acute lymphoid leukaemia	C91.0-C91.02	204.0-204.02
Chronic lymphoid leukaemia	C91.1-C91.12	204.1-204.12
Acute myeloid leukaemia	C92.0-C92.02, C92.3-C92.62, C93.0-C93.02, C94.0-C94.02, C94.2-C94.22, C94.4-C94.5	205.0-205.02, 205.3-205.32, 206.0-206.02, 207.0
Chronic myeloid leukaemia	C92.1-C92.12	205.1-205.12, 206.1-206.12, 207.1
Other leukaemia	C91.2-C91.9, C92.2, C92.7-C92.9, C93.1-C93.9, C94.1, C94.3, C94.6-C95.9	204.2-204.9, 205.2, 205.8-205.9, 206.2-207, 207.2-208.9
Other neoplasms	C17-C17.9, C30-C30.1, C31-C31.9, C37-C37.0, C38-C38.8, C40-C41.4, C41.8-C41.9, C47-C4A, C51-C52, C57-C57.8, C58-C58.0, C60-C60.9, C63-C63.8, C66-C66.9, C68.0-C68.8, C69-C69.92, C74-C75.5, C75.8	152-152.9, 158-158.9, 160-160.9, 163-164.9, 170-171.9, 181-181.9, 183.2-183.5, 184-184.9, 187-187.9, 189.2-189.4, 189.8-190.9, 192-192.9, 194-194.8, 209-209.03, 209.22, 209.25-209.27, 209.31-209.36

The ICD codes used for cancer mortality data are listed below:

Cause	ICD10	ICD9
Lip and oral cavity cancer	C00-C08.9, D10.0-D10.5, D11-D11.9	140-145.9, 210.0-210.6, 235.0
Nasopharynx cancer	C11-C11.9, D10.6	147-147.9, 210.7-210.9
Other pharynx cancer	C09-C10.9, C12-C13.9, D10.7	146-146.9, 148-148.9
Oesophageal cancer	C15-C15.9, D00.1, D13.0	150-150.9, 211.0, 230.1
Stomach cancer	C16-C16.9, D00.2, D13.1, D37.1	151-151.9, 211.1, 230.2
Colon and rectum cancer	C18-C21.9, D01.0-D01.3, D12-D12.9, D37.3-D37.5	153-154.9, 209.1, 209.5, 211.3-211.4, 230.3-230.6
Liver cancer	C22-C22.9, D13.4	155-155.9, 211.5
Gallbladder and biliary tract cancer	C23-C24.9, D13.5	156-156.9
Pancreatic cancer	C25-C25.9, D13.6-D13.7	157-157.9, 211.6-211.7
Larynx cancer	C32-C32.9, D02.0, D14.1, D38.0	161-161.9, 212.1, 231.0, 235.6
Tracheal, bronchus, and lung cancer	C33-C34.9, D02.1-D02.3, D14.2-D14.3, D38.1	162-162.9, 212.2-212.3, 231.1-231.2, 235.7
Malignant skin melanoma	C43-C43.9, D03-D03.9, D22-D23.9, D48.5	172-172.9
Non-melanoma skin cancer	C44-C44.9, D04-D04.9, D49.2	173-173.9, 222.4, 232-232.9, 238.2
Non-melanoma skin cancer (squamous-cell carcinoma)	C44-C44.9, D04-D04.9, D49.2	173-173.9, 222.4, 232-232.9, 238.2
Breast cancer	C50-C50.9, D05-D05.9, D24-D24.9, D48.6, D49.3, N60-N60.9	174-175.9, 217-217.8, 233.0, 238.3, 239.3, 610-610.9
Cervical cancer	C53-C53.9, D06-D06.9, D26.0	180-180.9, 219.0, 233.1
Uterine cancer	C54-C54.9, D07.0-D07.2, N87-N87.9	182-182.8, 233.2
Ovarian cancer	C56-C56.9, D27-D27.9, D39.1	183-183.0, 220-220.9, 236.2
Prostate cancer	C61-C61.9, D07.5, D29.1, D40.0	185-185.9, 222.2, 236.5
Testicular cancer	C62-C62.9, D29.2-D29.8, D40.1-D40.8	186-186.9, 222.0, 222.3, 236.4
Kidney cancer	C64-C65.9, D30.0-D30.1, D41.0-D41.1	189.0-189.1, 189.5-189.6, 223.0-223.1
Bladder cancer	C67-C67.9, D09.0, D30.3, D41.4-D41.8, D49.4	188-188.9, 223.3, 233.7, 236.7, 239.4
Brain and nervous system cancer	C70-C72.9	191-192.9
Thyroid cancer	C73-C73.9, D09.3, D09.8, D34-D34.9, D44.0	193-193.9, 226-226.9
Mesothelioma	C45-C45.9	
Hodgkin’s lymphoma	C81-C81.9	201-201.9
Non-Hodgkin lymphoma	C82-C86.6, C96-C96.9	200-200.9, 202-202.9
Multiple myeloma	C88-C90.9	203-203.9
Leukaemia	C91-C95.9	204-208.9
Acute lymphoid leukaemia	C91.0	204
Chronic lymphoid leukaemia	C91.1	204.1
Acute myeloid leukaemia	C92.0, C92.3-C92.6, C93.0, C94.0, C94.2, C94.4-C94.5	205.0, 205.3, 206.0, 207.0
Chronic myeloid leukaemia	C92.1	205.1, 206.1, 207.1

Other leukaemia	C91.2-C91.9, C92.2, C92.7-C92.9, C93.1-C93.9, C94.1, C94.3, C94.6-C95.9	204.2-204.9, 205.2, 205.8-205.9, 206.2-207, 207.2-208.9
Other neoplasms	C17-C17.9, C30-C31.9, C37-C38.8, C40-C41.9, C47-C4A, C51-C52.9, C57-C57.8, C58-C58.0, C60-C60.9, C63-C63.8, C66-C66.9, C68.0-C68.8, C69-C69.9, C74-C75.8, D07.4, D09.2, D13.2-D13.3, D14.0, D15-D16.9, D28.0-D28.1, D28.7, D29.0, D30.2, D30.4-D30.8, D31-D33.9, D35-D36, D36.1-D36.7, D37.2, D38.2-D38.5, D39.2, D39.8, D41.2-D41.3, D42-D43.9, D44.1-D44.8, D45-D47.0, D47.2-D47.9, D48.0-D48.4, D49.6, K31.7, K62.0-K62.1, K63.5, N84.0-N84.1	152-152.9, 158-158.9, 160-160.9, 163-164.9, 170-171.9, 181-181.9, 182.9, 183.2-183.8, 184.0-184.4, 184.8, 187.1-187.8, 189.2-189.4, 189.8, 190-190.9, 194-194.8, 209.0, 209.4, 211.2, 211.8, 212.0, 212.4-212.8, 213-213.9, 221.0-221.8, 222.1, 222.8, 223.2, 223.8, 224-225.9, 227-228.9, 229.0, 229.8, 230.7-230.8, 233.4-233.5, 234.0-234.8, 235.4, 235.8, 236.1, 237-237.3, 237.5-237.9, 238.0-238.1, 238.4-238.9, 239.2, 239.6, 569.0

ICD codes specifying benign neoplasms in the raw mortality data from vital registration systems were mapped to the respective malignant cause.

B. Data sources

Cancer incidence data sources

For India, cancer incidence was sought from individual cancer registries and from Cancer Incidence In Five Continents (CI5).[1–10] Data were excluded if they were not representative of the population (e.g., hospital-based registries), if they did not cover all malignant neoplasms as defined in ICD9 (140-208) or ICD10 (C00-C96) (e.g., specialty cancer registry), if they did not include data for both sexes and all age groups, if the data were limited to years prior to 1980, or if the source did not provide details on the population covered. A list of the cancer registries included in our analysis and the years covered can be found below. Additional metadata for each source are available in the online GBD citation tool, http://ghdx.healthdata.org/gbd-2016/data-input-sources.

Sources for cancer incidence by year and registry

Registry	Years available from registry	Number of years used for incidence
Ahmedabad	1983-2005	12
Ahmedabad rural	2006-2010	5
Ahmedabad urban	2006-2013	7
Aizawl	2005-2014	5
Aurangabad	2005-2014	9
Bangalore	1982-2012	24
Barshi expanded	2009-2012	2
Barshi rural	1988-2014	20
Bhopal	2004-2013	11
Cachar	2007-2014	7
Chandigarh union territory	2013	1
Chennai	1982-2013	34
Delhi	1993-2012	21
Dibrugarh	2005-2014	11
Dindigul Ambilikkai	2003-2013	7
Imphal	2005-2014	5
Kamrup urban	2005-2014	11

Karunagappally	1991-2007	17
Kolkata	2005-2012	6
Kollam	2006-2014	8
Manipur	2006-2010	5
Manipur excluding Imphal west	2009-2014	5
Mansa district	2013	1
Meghalaya	2010-2014	5
Mizoram	2003-2010	10
Mizoram excluding Aizawl	2005-2014	5
Mumbai	1964-2012	50
Nagaland	2010-2014	4
Nagpur	1980-2013	20
Naharlagun excluding Papum Pare	2012-2014	3
Papum Pare	2012-2014	3
Pasighat	2012-2014	3
Patiala district	2012-2014	3
Pune	1973-2013	32
S.A.S Nagar district	2013	1
Sangrur district	2013	1

Cancer mortality data sources

A detailed description of the data sources and processing steps for the cause of death database can be found in the appendix to the GBD 2016 cause of death paper.[11] The major data input to determine cancer mortality in India was the Sample Registration System (SRS) cause of death data, in addition to which data from the Medically Certified Cause of Death (MCCD) system and population-based cancer registries were also used as relevant.

SRS is operated by the Office of the Registrar General of India working under the Ministry of Home Affairs, Government of India. Cause of death data from SRS verbal autopsy included 455,460 deaths from the rural and urban populations of every state of India from 2004 to 2013 in which physicians assigned the cause of death based on the information provided in the verbal autopsy interview of a person close to each deceased person. Using the 2001 census, 7597 geographic units, 4433 (58·4%) of which were rural, were sampled for the 2004–13 SRS to represent the population of each state and union territory of India, ultimately with a sample of 6·7 million people that was equivalent to 0·7% of India's population. The SRS cause of death data for 2004–06, 2007–09, and 2010–13 were provided for each state and union territory by the Office of the Registrar General of India for use in the state-level disease burden estimation. We used 2005, 2008, and 2012 as midpoint years for these three time periods. The inclusion of SRS 2004–13 data in this analysis offers a comprehensive picture of causes of death in India. In the absence of a fully functional vital registration system, verbal autopsy can provide reasonable population level cause of death distribution.[12]

The MCCD system under the Office of the Registrar General of India has data mostly for the urban parts of the states and union territories beginning in 1980. MCCD covered only 22% of the deaths in India in 2015, with the coverage less than 20% in 15 states, 20–50% in ten states and union territories, and more than 50% in some states and union territories. Deaths reported in this data source are medically certified and are considered vital registration data.[12]

Most cancer registries only report cancer incidence. However, if a cancer registry also reported cancer mortality, mortality data were also extracted from the source to be used in the mortality to incidence estimation.

Bias in the input data

Bias in the input data included for the cause of death database is described elsewhere.[11] Cancer registry data can be biased in multiple ways. A high proportion of ill-defined cancer cases in the registry data requires redistribution of these cases to other cancers, which introduces a potential for bias. Changes between coding systems can lead to artificial differences in disease estimates; however, we adjust for this bias by mapping the different coding systems to the GBD

causes. Underreporting of cancers that require advanced diagnostic techniques (e.g., leukaemia, brain, pancreatic, and liver cancer) can be an issue in cancer registries from areas where access to these technologies is lacking. On the other hand, misclassification of metastatic sites as primary cancer can lead to overestimation of cancer sites that are common sites for metastases like brain or liver. Since many cancer registries are located in urban areas, the representativeness of the registry for the general population can also be problematic.

C. Cancer mortality and morbidity estimation

The approach to cause of death estimation for cancers is shown in the following flowchart:

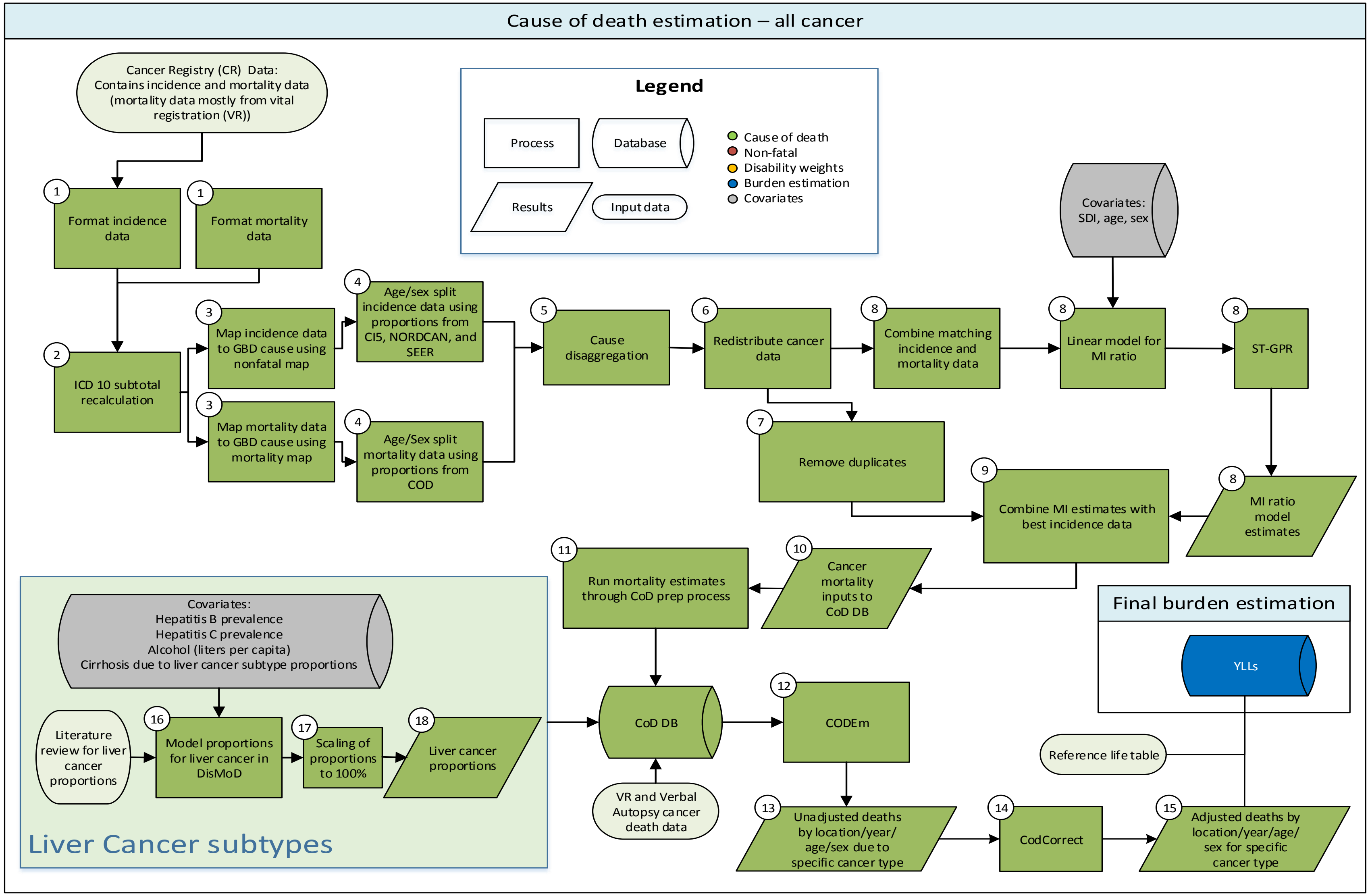

Cancer registry data went through multiple processing steps before integration with the cause of death database. First, the original data were transformed into standardised files, which included standardisation of format, categorization, and registry names (#1 in the flowchart below).

Second, some cancer registries report individual codes as well as aggregated totals (e.g., C18, C19, and C20 are reported individually but the aggregated group of C18–C20 (colorectal cancer) is also reported in the registry data). The data processing step "subtotal recalculation" (#2 in the flowchart) verifies these totals and subtracts the values of any individual codes from the aggregates.

In the third step (#3 in the flowchart), cancer registry incidence data and cancer registry mortality data are mapped to GBD causes. A different map is used for incidence and for mortality data because of the assumption that there are no deaths for certain cancers. One example is basal cell carcinoma of the skin. In the cancer registry incidence data, basal cell carcinoma is mapped to non-melanoma skin cancer (basal cell carcinoma). However, if basal cell skin cancer is recorded in the cancer registry mortality data, the deaths are instead mapped to non-melanoma skin cancer (squamous cell carcinoma) under the assumption that they were indeed misclassified squamous cell skin cancers. Other examples are benign or in situ neoplasms. Benign or in situ neoplasms found in the cancer registry incidence dataset were simply dropped from that dataset. The same neoplasms reported in a cancer registry mortality dataset were mapped to the respective invasive cancer (e.g., melanoma in situ in the cancer registry incidence dataset was dropped from the dataset; melanoma in situ in the cancer registry mortality dataset was mapped to melanoma). Mapping for incidence and mortality data can be found in the ICD code tables above.

In the fourth data processing step (#4 in the flowchart), cancer registry data were standardised to the GBD age groups. Age-specific incidence rates were generated using CI5, SEER, and NORDCAN data, while age-specific mortality rates were generated from the cause of death data, as applicable.[11] Age-specific weights were then generated by applying the age-specific rates to a given registry population that required age-splitting to produce the expected number of cases/deaths for that registry by age. The expected number of cases/deaths for each sex, age, and cancer were then normalized to 1, creating final, age-specific proportions. These proportions were then applied to the total number of cases/deaths by sex and cancer to get the age-specific number of cases/deaths.

In the rare case that the cancer registry only contained data for both sexes combined, the age-specific cases/deaths were split and re-assigned to separate sexes using the same weights that are used for the age-splitting process. Starting from the expected number of deaths, proportions were generated by sex for each age (e.g., if for ages 15-19 years old there are 6 expected deaths for males and 4 expected deaths for females, then 60% of the combined-sex deaths for ages 15-19 years would be assigned to males and the remaining 40% would be assigned to females).

In the fifth step (#5 in the flowchart), data for cause entries that are aggregates of GBD causes were redistributed. Examples of these aggregated causes include some registries reporting ICD10 codes C00-C14 together as, "lip, oral cavity, and pharyngeal cancer." These groups were broken down into subcauses that could be mapped to single GBD causes. In this example, those include lip and oral cavity cancer (C00-C08), nasopharyngeal cancer (C11), cancer of other parts of the pharynx (C09-C10, C12-C13), and "Malignant neoplasm of other and ill-defined sites in the lip, oral cavity, and pharynx" (C14). To redistribute the data, weights were created using the same method employed in age-sex splitting (see step four above). For the undefined code (C14 in the example) an "average all cancer" weight was used, which was generated by adding all cases from SEER/NORDCAN/CI5 and dividing those by the combined population. Then, proportions were generated by subcause for each aggregate cause as in the sex splitting example above (see step four). The total number of cases from the aggregated group (C00-C14) was recalculated for each subgroup and the undefined code (C14). C14 was then redistributed as a "garbage code" in step six. Distinct proportions were used for C46 (Kaposi sarcoma). C46 entries were redistributed as "other cancer" and HIV.

In the sixth step (#6 in the flowchart), unspecified codes ("garbage code") were redistributed. Redistribution of cancer registry incidence and mortality data mirrored the process of the redistribution used in the cause of death database.[11]

In the seventh step (#7 in the flowchart), duplicate or redundant sources were removed from the processed cancer registry dataset. Duplicate sources were present if, for example, the cancer registry was part of the CI5 database but we also had data from the registry directly. Redundancies occurred and were removed as described in "Inclusion and Exclusion Criteria," where more detailed data were available, or when national registry data could replace regionally representative data. From here, two parallel selection processes were run to generate input data for the MI (mortality-incidence) models and to generate incidence for final mortality estimation. Higher priority was given to registry data from the most standardised source when creating the final incidence input, whereas for the MI model input, only sources that reported incidence and mortality were used.

In the eighth step (#8 in the flowchart), the processed incidence and mortality data from cancer registries were matched by cancer, age, sex, year, and location to generate MI ratios. These MI ratios were used as input for a three-step modelling approach using the general GBD spatiotemporal Gaussian process regression (ST-GPR) approach with socio-demographic index as a covariate in the linear step mixed effects model using a logit link function. Predictions were made without the random effects. The ST-GPR model has three main hyper-parameters that control for smoothing across time, age, and geography. The time adjustment parameter (λ) was set to 2, which aims to borrow strength from neighboring time points (i.e., the exposure in this year is highly correlated with exposure in the previous year but less so further back in time). The age adjustment parameter ω was set to 0.5, which borrows strength from data in neighboring age groups. The space adjustment parameter ξ was set to 0.95 in locations with data and to 0.5 in locations without data (the higher ξ was applied when at least one age-sex group in the country of estimation had at least five unique data points. The lower ξ was applied when estimating data-scarce countries). Zeta aims to borrow strength across the hierarchy of geographical locations.[11] For the amplitude parameter in the Gaussian process regression we used 2 and for the scale we used a value of 15.

Since MI ratios can be above 1, especially in older age groups and cancers with low cure rates, we used the 95th percentile of the cleaned dataset that only included MIR that were based on 50 or more cases to cap the MIR input data. This "upper cap" was used to allow MIR over 1 but to constrain the MIR to a maximum level. To run the logit model, the input data were divided by the upper caps and model predictions after ST-GPR was rescaled by multiplying them by the upper caps. To constrain the model at the lower end, we used the 5th percentile of the cancer-specific cleaned MIR input data to replace all model predictions with this lower cap.

Final MI ratios were matched with the cancer registry incidence dataset in the ninth step (#9 in the flowchart) to generate mortality estimates (Incidence * Mortality/Incidence = Mortality) (#10 in the flowchart). The final mortality estimates were then uploaded into the cause of death database (#11 in the flowchart). Cancer-specific mortality modelling then followed the general cause of death ensemble modelling (CODEm) process.

Formatting of data sources for the cause of death database has been described in detail elsewhere (#11 in the flowchart).[11]

CODEm models

Mortality estimates for each cancer were generated using CODEm (#12 in the flowchart). Methods describing the CODEm approach have been described elsewhere.[11,13] In brief, the CODEm modelling approach is based on the principles that all types of available data should be used even if data quality varies; that individual models but also ensemble models should be tested for their predictive validity; and that the best model or sets of models should be chosen based on the out of sample predictive validity. Models were run separately for countries with extensive and complete vital registration data and countries with less VR data to prevent an inflation in the uncertainty around the estimates in "data-rich" countries. Covariates were selected based on a possible predictive relationship between the covariate and the specific cancer mortality. Level 1 covariates have a proven strong relationship with the outcome such as etiological or biological roles. Level 2 covariates have a strong relationship but not a direct biological link. Covariates that are more distal in the causal chain or are mediated through Level 1 or 2 covariates are categorised as Level 3.[13]

Liver cancer etiology split models

To find the proportion of liver cancer cases due to the four etiology groups included in GBD (1. Liver cancer due to hepatitis B, 2. Liver cancer due to hepatitis C, 3. Liver cancer due to alcohol, 4. Liver cancer due to other causes), a systematic literature search was performed in PubMed using the search string *("liver neoplasms"[All Fields] OR "HCC"[All Fields] OR "liver cancer"[All Fields] OR "Carcinoma, Hepatocellular"[Mesh]) AND (("hepatitis B"[All Fields] OR "Hepatitis B"[Mesh] OR "Hepatitis B virus"[Mesh] OR "Hepatitis B Antibodies"[Mesh] OR "Hepatitis B Antigens"[Mesh]) OR ("hepatitis C"[All Fields] OR "Hepatitis C"[Mesh] OR "hepatitis C antibodies"[MESH] OR "Hepatitis C Antigens"[Mesh] OR "Hepacivirus"[Mesh]) OR ("alcohol"[All Fields] OR "Alcohol Drinking"[Mesh] OR "Alcohol-Related Disorders"[Mesh] OR "Alcoholism"[Mesh] OR "Alcohol-Induced Disorders"[Mesh])) AND ("2015"[PDAT] : "2016"[PDAT]) NOT (animals[MeSH] NOT humans[MeSH])*. Studies were included if the study population was representative of the liver cancer population for the respective location. For each study the proportions of liver cancer due to the three specific risk factors were calculated. Remaining risk factors were included under a combined "other" group. Cryptogenic cases were only included if other etiologies like viral hepatitis or alcoholic cirrhosis had been excluded. If multiple risk factors were reported for an individual patient these were apportioned proportionally to the individual risk factors. The proportion data found through the systematic literature review were used as input for four separate DisMod-MR 2.1 models to determine the proportion of liver cancers due to the four subgroups for all locations, both sexes, and all age groups (step #16 in the flowchart). A study covariate was used for

publications that only assessed liver cancer in a cirrhotic population. The reference, or “gold standard,” that was used for crosswalking was the compilation of all studies that assessed the etiology of liver cancer in a general population. A study covariate was also used for studies that only assessed hepatocellular carcinoma (HCC) as opposed to all primary liver cancers. The reference or “gold standard” that was used for crosswalking was the compilation of all studies that assessed the etiology of liver cancer for the population with all primary liver cancers.

For liver cancer due to hepatitis C and hepatitis B, a prior value of 0 was set between age 0 and 0.01. For liver cancer due to alcohol a prior value of 0 was set for ages 0 to 5 years. For liver cancer due to hepatitis C, hepatitis C (IgG) seroprevalence was used as a covariate as well as a covariate for alcohol (liters per capita) and hepatitis B prevalence (HBsAg seroprevalence), forcing a negative relationship between the alcohol and hepatitis B covariate and the outcome of liver cancer due to hepatitis C proportion. For liver cancer due to hepatitis B, seroprevalence of HBsAg was used as a covariate as well as a covariate for alcohol and hepatitis C IgG seroprevalence, forcing a negative relationship between the alcohol and hepatitis C covariate and the outcome of liver cancer due to hepatitis B proportion. For liver cancer due to alcohol, alcohol (liters per capita) was used as a covariate as well as a covariate for proportion of alcohol abstainers, hepatitis B and hepatitis C seroprevalence, forcing a negative relationship between the proportion of alcohol abstainers, hepatitis B and hepatitis C covariates and the outcome of liver cancer due to alcohol proportion. All covariates used were modelled independently. To ensure consistency between cirrhosis and liver cancer estimates and to take advantage of the data for the respective other related cause (e.g., liver cancer due to hepatitis C and the related cause cirrhosis due to hepatitis C), we generated covariates from the liver cancer proportion models that we used in the cirrhosis etiology proportion models. We then created covariates from the cirrhosis etiology proportion models and used those in the liver cancer etiology models.

Since the proportion models are run independently of each other, the final proportion models were scaled to sum to 100% within each age, sex, year, and location, by dividing each proportion by the sum of the four (step # 17). For the liver cancer subtype mortality estimates, we multiplied the parent cause “liver cancer” by the corresponding scaled proportions (step # 18). Single cause estimates were adjusted to fit into the separately modelled all-cause mortality in the process CodCorrect.

CodCorrect

CODEm models estimate the individual cause-level mortality without taking into account the all-cause mortality (#13 in the flowchart). To ensure that all single causes add up to the all-cause mortality and that all child-causes add up to the parent cause, an algorithm called “CodCorrect” is used (#14 and #15 in the flowchart). Details regarding the CodCorrect algorithm can be found elsewhere.[11]

The approach to non-fatal cancer burden or morbidity estimation for cancers is shown in the following flowchart:

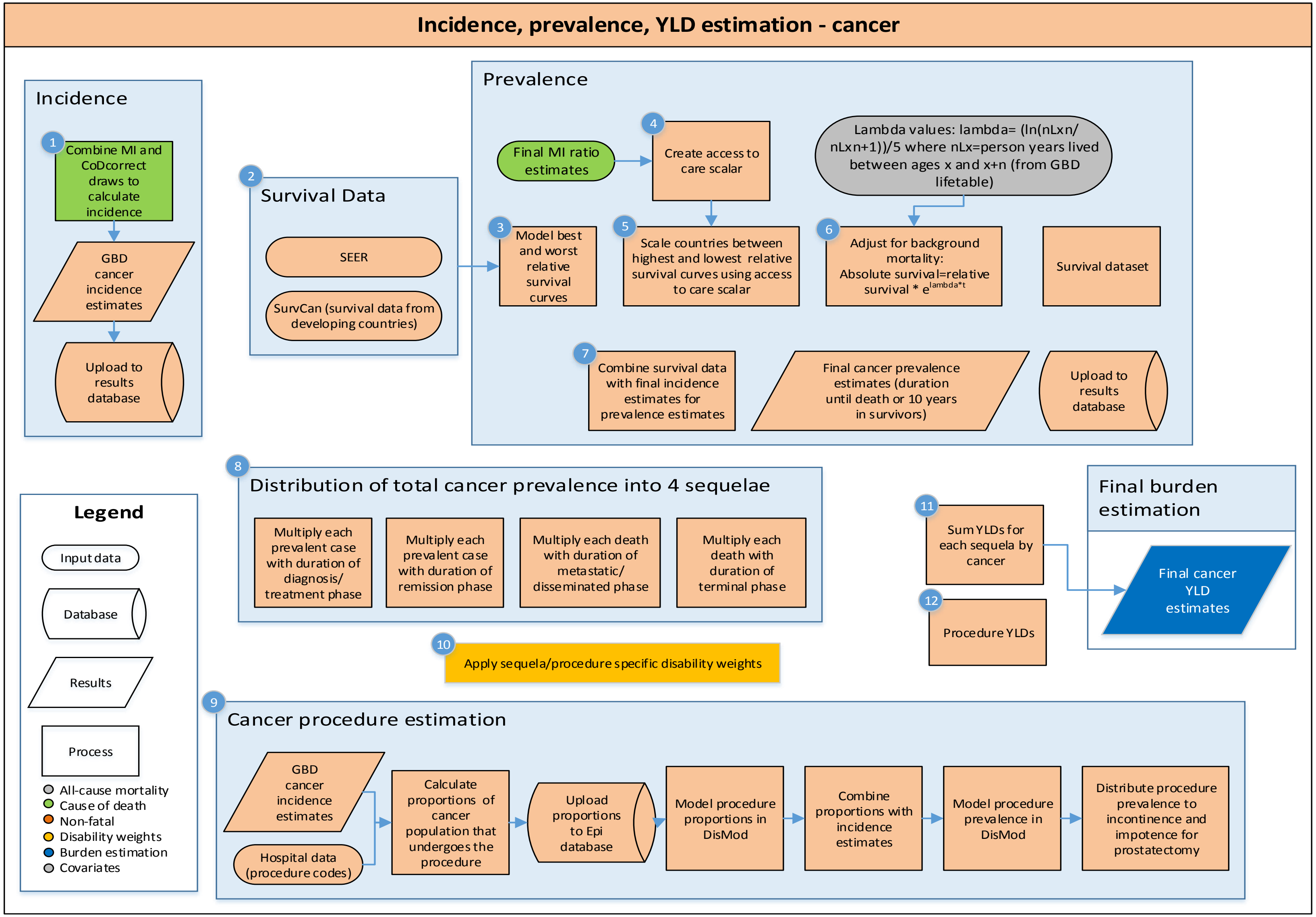

GBD cancer incidence estimates were generated by dividing final mortality estimates (after CodCorrect adjustment) by the MI ratio for the specific cancer (#1 in flowchart below). To propagate uncertainty from the MI ratios and the mortality estimates to incidence, this process was done at the 1,000-draw level. It was assumed that uncertainty in the MI ratio is independent of uncertainty in the estimated age-specific death rates.

Prevalence is estimated as 10-year prevalence for all cancers.[14] To estimate cancer prevalence, relative cancer survival was estimated by scaling cancer-specific survival between a "best case" and "worst case" survival. The methods and input data used to generate the best and worst case survival as well as to scale countries between these boundaries remained the same (# 2, 3, and 5 in the flowchart).[14] For the cause "other leukaemia" , survival data were updated using SEER 1973 survival data for "other leukaemia" as the worst-case scenario and SEER 2010 survival data as the best-case survival. To transform relative to absolute survival (adjusting for background mortality) GBD 2016 lifetables were used (# 6 and 7 in the flowchart).[15] The access to cancer care variable to scale countries between the best and worst case survival was estimated (# 4 in the flowchart):[14]

$$Access\ to\ care = 1 - \frac{Age\ standardised\ MI\ ratio_{cys} - Age\ standardised\ MI\ ratio_{min}}{Age\ standardised\ MI\ ratio_{max} - Age\ standardised\ MI\ ratio_{min}}$$

c=country; y=year; s=sex; Age-standardised MI $ratio_{min}$=lowest MI ratio for all countries and years; Age-standardised MI $ratio_{max}$=highest MI ratio for all countries and years

Duration of the treatment phases (1. diagnosis and primary therapy; 2. Controlled phases; 3. Metastatic phase; 4. Terminal phase) remained the same, with the exception of the "other leukaemia" cause, which was added for GBD 2016. Total prevalence time was divided into phases 1, 3, and 4 for the population that died within 10 years, and the remaining prevalence was attributed to the controlled phase. For the population that survived beyond 10 years, prevalence person time was attributed to phase 1 and phase 2 (#8 in the flowchart). YLDs were calculated by multiplying each phase with the respective disability weight. To generate the total YLDs for each cancer (with the exception of cancers where additional disability is added due to procedures – see next paragraph) the YLDs for each cancer sequela were added.

Additional disability was estimated for breast cancer (disability due to mastectomy), larynx cancer (disability due to laryngectomy), colon and rectum cancer (disability due to stoma), bladder cancer (disability due to incontinence), and prostatectomy (disability due to incontinence and impotence) (#10). Hospital data were used to estimate the number of cancer patients undergoing mastectomy, laryngectomy, stoma, prostatectomy, and cystectomy. These proportions were used as input for proportion models that were run in DisMod-MR 2.0 (#9).[14,16] The procedure proportions (proportion of cancer population that undergoes procedures) from hospital data was used as input for a proportion model in DisMod-MR 2.0 in order to estimate the proportions for all locations, by age, and by sex.

Since colostomy or ileostomy procedures are done for reasons other than cancer, a literature review was done to determine the proportion of ostomies due to colorectal cancer. The "all cause" colostomy proportions were multiplied by 0.58 based on the results of the literature review showing that on average 58% of ostomies are done for colorectal cancer. [17–19]

The final procedure proportions were applied to the incidence cases of the respective cancers and multiplied with the proportion of the incidence population surviving for 10 years to determine the incident cases of the cancer population that underwent procedures. These incident cases were used again as an input for DisMod-MR 2.1, with a remission specification of zero and an excess mortality rate prior of 0 to 0.1. Mortality for the population undergoing disease-specific procedures (e.g., mastectomy for breast cancer) is likely closer to the general population after they have survived for a period of time compared to the cause-specific mortality of the underlying disease (e.g., breast cancer).

Since disability associated with prostatectomy comes from impotence and incontinence and not from the prostatectomy itself, 18% of the prostatectomy prevalence was assumed to be incontinent and 55% was assumed to be impotent based on a literature review.[20–27]

Since all sequelae for a cause need to be mutually exclusive, the controlled phase for the cancers with additional procedure-related disability was adjusted to only include the population without procedure-related disability (= controlled phases prevalence of the total population – controlled phase prevalence of the proportion that experienced

procedure related disability) (#11). The disability weight for the prevalence of the population that experiences additional disability was adjusted to reflect the combined disability of the controlled phase as well as the procedure.

Lastly, the procedure sequelae prevalence and general sequelae prevalence were multiplied with disability weights for the procedures to obtain the number of YLDs (#10). The sum of these YLDs are the final YLD estimate associated with each cancer.

D. Uncertainty intervals

Point estimates for each quantity of interest were derived from the mean of the draws, while 95% uncertainty intervals (UIs) were derived from the 2.5th and 97.5th percentiles of the 1000 draw level values. Uncertainty in the estimation is attributable to sample size variability within data sources, different availability of data by age, sex, year, or location, and cause specific model specifications. We determined UIs for components of cause-specific estimation based on 1000 draws from the posterior distribution of cause specific mortality by age, sex, and location for each year included in the GBD 2016 analysis. Similarly, for non-fatal estimates if there was a change in disease estimates between locations or over time that was in the same direction in more than 950 of the 1000 samples we report it as significant. With this approach, uncertainty could be quantified and propagated into the final quantities of interest.

References

1 Doll R, Payne P, Waterhouse J. Cancer incidence in five continents I. Geneva: Union Internationale Contre le Cancer, 1966.

2 Doll R, Muir C, Waterhouse J. Cancer incidence in five continents II. Geneva: Union Internationale Contre le Cancer, Geneva, 1970.

3 Waterhouse J, Muir C, Correa P, Powell J. Cancer incidence in five continents III. Lyon: IARC, 1976.

4 Waterhouse J, Muir C, Shanmugaratnam K, Powell J. Cancer incidence in five continents IV. Lyon: IARC, 1982.

5 Muir C, Mack T, Powell J, Whelan S. Cancer incidence in five continents V. Lyon: IARC, 1987.

6 Parkin D, Muir C, Whelan S, Gao Y, Ferlay J, Powell J. Cancer incidence in five continents VI. Lyon: IARC, 1992.

7 Parkin D, Whelan S, Ferlay J, Raymond L, Young J. Cancer incidence in five continents VII. Lyon: IARC, 1997.

8 Parkin D, Whelan S, Ferlay J, Teppo L, Thomas D. Cancer incidence in five continents VIII. Lyon: IARC, 2002.

9 Curado M, Edwards B, Shin H, *et al.* Cancer incidence in five continents IX. Lyon: IARC, 2007 http://www.iarc.fr/en/publications/pdfs-online/epi/sp160/CI5vol9-A.pdf.

10 Forman D, Bray F, Brewster D, *et al.* Cancer incidence in five continents X. 2013. http://ci5.iarc.fr.

11 GBD 2016 Causes of Death Collaborators. Global, regional, and national age-sex specific mortality for 264 causes of death, 1980–2016: a systematic analysis for the Global Burden of Disease Study 2016. *Lancet* 2017; **390**: 1151–210.

12 India State-Level Disease Burden Initiative Collaborators. Nations within a nation: variations in epidemiological transition across the states of India, 1990–2016 in the Global Burden of Disease Study. Lancet 2017; 390: 2437–60.

13 Foreman KJ, Lozano R, Lopez AD, Murray CJ. Modeling causes of death: an integrated approach using CODEm. *Popul Health Metr* 2012; **10**: 1.

14 Global Burden of Disease Cancer Collaboration. The global burden of cancer 2013. *JAMA Oncol* 2015; **1**: 505-527.

15 GBD 2015 Mortality and Causes of Death Collaborators. Global, regional, and national life expectancy, all-cause and cause specific mortality for 249 causes of death, 1980–2015: a systematic analysis for the Global Burden of Disease Study 2015. *Lancet* 2016; **388**: 1459–544.

16 Flaxman AD, Vos T, Murray C. An integrative metaregression framework for descriptive epidemiology. University of Washington Press, 2015.

17 Canova C, Giorato E, Roveron G, Turrini P, Zanotti R. Validation of a stoma-specific quality of life questionnaire in a sample of patients with colostomy or ileostomy. *Colorectal Dis Off J Assoc Coloproctology G B Irel* 2013; **15**: e692-698.

18 Caricato M, Ausania F, Ripetti V, Bartolozzi F, Campoli G, Coppola R. Retrospective analysis of long-term defunctioning stoma complications after colorectal surgery. *Colorectal Dis Off J Assoc Coloproctology G B Irel* 2007; **9**: 559–61.

19 Erwin-Toth P, Thompson SJ, Davis JS. Factors impacting the quality of life of people with an ostomy in North America: results from the Dialogue Study. *J Wound Ostomy Cont Nurs Off Publ Wound Ostomy Cont Nurses Soc WOCN* 2012; **39**: 417–22; quiz 423–4.

20 Catalona WJ, Carvalhal GF, Mager DE, Smith DS. Potency, continence and complication rates in 1,870 consecutive radical retropubic prostatectomies. *J Urol* 1999; **162**: 433–8.

21 Donnellan SM, Duncan HJ, MacGregor RJ, Russell JM. Prospective assessment of incontinence after radical retropubic prostatectomy: objective and subjective analysis. *Urology* 1997; **49**: 225–30.

22 Eastham JA, Kattan MW, Rogers E, *et al.* Risk factors for urinary incontinence after radical prostatectomy. *J Urol* 1996; **156**: 1707–13.

23 Kundu SD, Roehl KA, Eggener SE, Antenor JAV, Han M, Catalona WJ. Potency, continence and complications in 3,477 consecutive radical retropubic prostatectomies. *J Urol* 2004; **172**: 2227–31.

24 Potosky AL, Davis WW, Hoffman RM, *et al.* Five-year outcomes after prostatectomy or radiotherapy for prostate cancer: the prostate cancer outcomes study. *JNCI J Natl Cancer Inst* 2004; **96**: 1358–67.

25 Sacco E, Prayer-Galetti T, Pinto F, *et al.* Urinary incontinence after radical prostatectomy: incidence by definition, risk factors and temporal trend in a large series with a long-term follow-up. *BJU Int* 2006; **97**: 1234–41.

26 Stanford JL, Feng Z, Hamilton AS, *et al.* Urinary and sexual function after radical prostatectomy for clinically localized prostate cancer: the prostate cancer outcomes study. *JAMA* 2000; **283**: 354–60.

27 Walsh PC, Marschke P, Ricker D, Burnett AL. Patient-reported urinary continence and sexual function after anatomic radical prostatectomy. *Urology* 2000; **55**: 58–61.

2. GBD 2016 India data inputs for cancer mortality, morbidity, risk factors, and covariates

Action Aid International, Regional Medical Research Centre - Port Blair, Indian Council of Medical Research, National Institute of Mental Health and Neurosciences. Report on alcohol consumption prevalence and patterns in Andaman and Nicobar Islands 2007. Available from: http://nimhans.ac.in/cam/sites/default/files/Publications/10.pdf
Agarwal N, Naik S, Aggarwal R, Singh H, Somani SK, Kini D, Pandey R, Choudhuri G, Saraswat VA, Naik SR. Occult hepatitis B virus infection as a cause of cirrhosis of liver in a region with intermediate endemicity. Indian J Gastroenterol. 2003; 22(4): 127-31.
Amarapurkar DN, Dharod M, Gautam S, Patel N. Risk of development of hepatocellular carcinoma in patients with NASH-related cirrhosis. Trop Gastroenterol. 2013; 34(3): 159-63.
Asim M, Sarma MP, Kar P. Etiological and molecular profile of hepatocellular cancer from India. Int J Cancer. 2013; 133(2): 437-45.
Bhagyalaxmi A, Atul T, Shikha J. Prevalence of risk factors of non-communicable diseases in a District of Gujarat, India. J Health Popul Nutr. 2013; 31(1): 78-85.
Cancer Institute (Women's India Association), International Agency for Research on Cancer. Dindigul Ambilikkai Cancer Registry 2012-2013. [Data shared for this analysis]
Cancer Institute (Women's India Association), Ministry of Health and Family Welfare, Government of Tamil Nadu. Tamil Nadu Cancer Registry Project (TNCRP) 2012-2013. [Data shared for this analysis]
Center for Addiction Medicine, National Institute of Mental Health and Neurosciences, Government of India, World Health Organization Collaborative Programme. Report on Patterns and Consequences of Alcohol Misuse in India: An Epidemiological Survey 2011-2012. Available from: http://nimhans.ac.in/cam/sites/default/files/Publications/WHO_ALCOHOL%20IMPACT_REPORT-FINAL21082012.pdf
Christian Medical College - Vellore. India Prevalence of Risk Factors for Non-Communicable Diseases in Rural and Urban Tamil Nadu 2010-2012. [Data shared for this analysis]
Curado MP, Edwards B, Shin HR, Storm H, Ferlay J, Heanue M, Boyle P, eds. Cancer Incidence in Five Continents, Vol. IX. International Agency for Research on Cancer (IARC) Scientific Publications, No. 160, Lyon, France: IARC; 2007.
Danawala SA, Arora M, Stigler MH. Analysis of motivating factors for smokeless tobacco use in two Indian states. Asian Pac J Cancer Prev. 2014; 15(16): 6553-8.
Das M, Sharma SK, Sekhon GS, Saikia BJ, Mahanta J, Phukan RK. Promoter methylation of MGMT gene in serum of patients with esophageal squamous cell carcinoma in North East India. Asian Pac J Cancer Prev. 2014; 15(22): 9955-60.
Department of Health and Family Welfare, Government of Punjab, Postgraduate Institute of Medical Education & Research Chandigarh, University of Michigan. India - Punjab Noncommunicable Disease Risk Factors Survey 2014-2015. [Data shared for this analysis]
Department of Women and Child Development, Ministry of Human Resource Development, Government of India, United Nations Children's Fund (UNICEF) - India Country Office. India Summary Report on the Multiple Indicator Cluster Survey 2000. New Delhi, India: UNICEF India Country Office; 2000.
Department of Women and Child Development, Ministry of Human Resource Development, Government of India. India Nutrition Profile Survey 1995-1996. New Delhi, India: Ministry of Human Resource Development.
Desai, Sonalde, Reeve Vanneman, National Council of Applied Economic Research, University of Michigan. India Human Development Survey 2005. Ann Arbor, Michigan: Inter-University Consortium for Political and Social Research.
Dikshit RP, Kanhere S. Tobacco habits and risk of lung, oropharyngeal and oral cavity cancer: a population-based case-control study in Bhopal, India. Int J Epidemiol. 2000; 29(4): 609-14.
Directorate of Economics & Statistics and Office of Chief Registrar (Births & Deaths), Government of National Capital Territory of Delhi. Report on Medical Certification of Cause of Deaths in Delhi-2011. New Delhi, India: Directorate of Economics & Statistics and Office of Chief Registrar (Births & Deaths); 2012.
Directorate of Economics & Statistics and Office of Chief Registrar (Births & Deaths), Government of National Capital Territory of Delhi. Report on Medical Certification of Cause of Deaths in Delhi-2012. New Delhi, India: Directorate of Economics & Statistics and Office of Chief Registrar (Births & Deaths); 2014.
Directorate of Economics & Statistics and Office of Chief Registrar (Births & Deaths), Government of National Capital Territory of Delhi. Report on Medical Certification of Cause of Deaths in Delhi-2013. New Delhi, India: Directorate of Economics & Statistics and Office of Chief Registrar (Births & Deaths); 2014.
Doll R, Muir CS, Waterhouse JAH, eds. Cancer Incidence in Five Continents, Vol. II. Geneva, Switzerland: Union Internationale Contre le Cancer; 1970.
Embassy of the United States - New Delhi, United States Environmental Protection Agency. United States Mission India NowCast Air Quality Data 2014. New Delhi, India: Embassy of the United States.

Euromonitor International. Euromonitor Passport - Cigarette Statistics 1999-2016. London, United Kingdom: Euromonitor International.
Food and Agriculture Organization of the United Nations (FAO). FAO Supply Utilization Accounts 1961-2013. Personal Correspondence with Dr. Josef Schmidhuber, 2016. [Data shared for this analysis]
Food and Agriculture Organization of the United Nations (FAO). FAOSTAT Commodity Balances - Crops Primary Equivalent. Rome, Italy: FAO.
Food and Agriculture Organization of the United Nations (FAO). FAOSTAT Food Balance Sheets, October 2014. Rome, Italy: FAO.
Forman D, Bray F, Brewster DH, Gombe Mbalawa C, Kohler B, Piñeros M, Steliarova-Foucher E, Swaminathan R, Ferlay J, eds. Cancer Incidence in Five Continents, Vol. X. International Agency for Research on Cancer (IARC) Scientific Publications, No. 164. Lyon, France: IARC; 2014.
Ganju SA, Goel A. Sero-surveillance of HIV, HBV and HCV infections in antenatal and STD clinic attendees. J Commun Dis. 2004; 36(1): 60-2.
Garg R, Kaur S, Aseri R, Aggarwal S, Singh JP, Mann S, Kumar S, Kaur S. Hepatitis B & C among farmers - a seroprevalence study. J Clin Diagn Res. 2014; 8.0(11): MC07-9.
Garg S, Mathur DR, Garg DK. Comparison of seropositivity of HIV, HBV, HCV and syphilis in replacement and voluntary blood donors in western India. Indian J Pathol Microbiol. 2001; 44(4): 409-12.
Ghosh I, Ghosh P, Bharti AC, Mandal R, Biswas J, Basu P. Prevalence of human papillomavirus and co-existent sexually transmitted infections among female sex workers, men having sex with men and injectable drug abusers from eastern India. Asian Pac J Cancer Prev. 2012; 13(3): 799–802.
Global Burden of Disease Health Financing Collaborator Network, Institute for Health Metrics and Evaluation (IHME). Global Development Assistance for Health, Government, Prepaid Private, and Out-of-Pocket Health Spending 1995-2014. Seattle, United States: IHME; 2017.
Hashibe M, Sankaranarayanan R, Thomas G, Kuruvilla B, Mathew B, Somanathan T, Parkin DM, Zhang ZF. Alcohol drinking, body mass index and the risk of oral leukoplakia in an Indian population. Int J Cancer. 2000; 88(1): 129-34.
Healis-Sekhsaria Institute for Public Health, Madhya Pradesh Voluntary Health Association, Cancer Foundation of India, School of Preventive Oncology, University of Waterloo. International Tobacco Control Policy Evaluation Project: India Tobacco Control Survey 2010-2011. Navi Mumbai, India and Waterloo, Canada: Healis-Sekhsaria Institute for Public Health and University of Waterloo.
Healis-Sekhsaria Institute for Public Health, Madhya Pradesh Voluntary Health Association, Cancer Foundation of India, School of Preventive Oncology, University of Waterloo. International Tobacco Control Policy Evaluation Project: India Tobacco Control Survey 2010-2011. Navi Mumbai, India and Waterloo, Canada: Healis-Sekhsaria Institute for Public Health and University of Waterloo.
Health Department - Chandigarh Administration, Postgraduate Institute of Medical Education & Research (PGIMER) Chandigarh, Tata Memorial Center. Cancer Incidence and Mortality in Chandigarh Union Territory 2013. Chandigarh, India: PGIMER Chandigarh.
Indian Council of Medical Research (ICMR), Madras Diabetes Research Foundation. Indian Council of Medical Research India Diabetes Study (ICMR-INDIAB) - North East 2012-2015. [Data shared for this analysis]
Indian Council of Medical Research (ICMR), Madras Diabetes Research Foundation. Indian Council of Medical Research India Diabetes Study (ICMR-INDIAB) 2008-2010. [Data shared for this analysis]
Indian Council of Medical Research (ICMR), Madras Diabetes Research Foundation. Indian Council of Medical Research India Diabetes Study (ICMR-INDIAB) 2012-2013. [Data shared for this analysis]
Indian Council of Medical Research (ICMR). India Study on Causes of Death by Verbal Autopsy 2003. New Delhi, India: ICMR. [Data shared for this analysis]
Indian Council of Medical Research, World Health Organization. India STEPS Noncommunicable Disease Risk Factors Survey 2003-2005. [Data shared for this analysis]
Indian Institute of Health Management Research, International Institute of Population Sciences, Ministry of Health and Family Welfare, Government of India, Performance Monitoring and Accountability 2020 (PMA2020) Project, Bill & Melinda Gates Institute for Population and Reproductive Health, Johns Hopkins Bloomberg School of Public Health. PMA 2016 Rajasthan Round 1 Survey Data as part of PMA2020 Project. Baltimore, United States: PMA2020.
Institute of Health Systems, World Health Organization (WHO). WHO Multi-country Survey Study Report on Health and Health System Responsiveness, Andhra Pradesh 2000-2001.
International Institute for Population Sciences (IIPS), Ministry of Health and Family Welfare, Government of India, Harvard T. H. Chan School of Public Health, RAND Corporation, Monash University, University of California Los Angeles, Indian Academy of Geriatrics, National AIDS Research Institute, Columbia University. Longitudinal Aging Study in India, Pilot Data 2010. Mumbai, India: IIPS.

International Institute for Population Sciences (IIPS), Ministry of Health and Family Welfare, Government of India, Macro International. India National Family Health Survey Data (NFHS-1) 1992-1993. Mumbai, India: IIPS.

International Institute for Population Sciences (IIPS), Ministry of Health and Family Welfare, Government of India, Macro International. India National Family Health Survey (NFHS-1) 1992-1993: National Report. Mumbai, India: IIPS.

International Institute for Population Sciences (IIPS), Ministry of Health and Family Welfare, Government of India, ORC Macro. India National Family Health Survey Data (NFHS-2) 1998-1999. Mumbai, India: IIPS.

International Institute for Population Sciences (IIPS), Ministry of Health and Family Welfare, Government of India, ORC Macro. India National Family Health Survey (NFHS-2) 1998-1999: National and State Reports. Mumbai, India: IIPS.

International Institute for Population Sciences (IIPS), Ministry of Health and Family Welfare, Government of India, Macro International. India National Family Health Survey Data (NFHS-3) 2005-2006. Mumbai, India: IIPS.

International Institute for Population Sciences (IIPS), Ministry of Health and Family Welfare, Government of India, Macro International. India National Family Health Survey (NFHS-3) 2005-2006: National and State Reports. Mumbai, India: IIPS.

International Institute for Population Sciences (IIPS), Ministry of Health and Family Welfare, Government of India, ICF. India National Family Health Survey (NFHS-4) 2015-2016: National and State-level Factsheets. Mumbai, India: IIPS.

International Institute for Population Sciences (IIPS), Ministry of Health and Family Welfare, Government of India. India District Level Household Survey Data (DLHS-1) 1998-1999. Mumbai, India: IIPS.

International Institute for Population Sciences (IIPS), Ministry of Health and Family Welfare, Government of India. India District Level Household Survey (DLHS-1) 1998-1999: National Report. Mumbai, India: IIPS.

International Institute for Population Sciences (IIPS), Ministry of Health and Family Welfare, Government of India. India District Level Household Survey Data (DLHS-2) 2002-2004. Mumbai, India: IIPS.

International Institute for Population Sciences (IIPS), Ministry of Health and Family Welfare, Government of India. India District Level Household Survey (DLHS-2) 2002-2004: National and State Reports. Mumbai, India: IIPS.

International Institute for Population Sciences (IIPS), Ministry of Health and Family Welfare, Government of India. India District Level Household and Facility Survey Data (DLHS-3) 2007-2008. Mumbai, India: IIPS.

International Institute for Population Sciences (IIPS), Ministry of Health and Family Welfare, Government of India. India District Level Household and Facility Survey (DLHS-3) 2007-2008: National and State Reports. Mumbai, India: IIPS.

International Institute for Population Sciences, Ministry of Health and Family Welfare, Government of India. India District Level Household and Facility Survey Data (DLHS-4) 2012-2013. Mumbai, India: IIPS.

International Institute for Population Sciences (IIPS), Ministry of Health and Family Welfare, Government of India. India District Level Household and Facility Survey Data (DLHS-4) 2012-2013: State Reports. Mumbai, India: IIPS.

International Institute for Population Sciences, World Health Organization (WHO). India WHO Study on Global Ageing and Adult Health 2007-2008.

International Institute for Population Sciences, World Health Organization. India World Health Survey 2003.

International Labour Organization (ILO). International Labour Statistics Database (ILOSTAT) - Employment to Population Ratio. Geneva, Switzerland: ILO.

Jayalekshmi PA, Gangadharan P, Akiba S, Koriyama C, Nair RR. Oral cavity cancer risk in relation to tobacco chewing and bidi smoking among men in Karunagappally, Kerala, India: Karunagappally cohort study. Cancer Sci. 2011; 102(2.0): 460-7.

Jayalekshmi PA, Gangadharan P, Akiba S, Nair RR, Tsuji M, Rajan B. Tobacco chewing and female oral cavity cancer risk in Karunagappally cohort, India. Br J Cancer. 2009; 100(5.0): 848-52.

Joint United Nations Program on HIV/AIDS (UNAIDS). UNAIDS Spectrum - National HIV Estimates 2016. Geneva, Switzerland: UNAIDS.

Jussawalla DJ, Deshpande VA. Evaluation of cancer risk in tobacco chewers and smokers: an epidemiologic assessment. Cancer. 1971; 28(1): 244-52.

Kothari A, Ramachandran VG, Gupta P, Singh B, Talwar V. Seroprevalence of cytomegalovirus among voluntary blood donors in Delhi, India. J Health Popul Nutr. 2002; 20(4): 348-51.

Kumar M, Kumar R, Hissar SS, Saraswat MK, Sharma BC, Sakhuja P, Sarin SK. Risk factors analysis for hepatocellular carcinoma in patients with and without cirrhosis: a case-control study of 213 hepatocellular carcinoma patients from India. J Gastroenterol Hepatol. 2007; 22(7): 1104-11.

Kumar R, Saraswat MK, Sharma BC, Sakhuja P, Sarin SK. Characteristics of hepatocellular carcinoma in India: a retrospective analysis of 191 cases. QJM. 2008; 101(6): 479-85.
Max Planck Institute for Demographic Research, Vienna Institute of Demography. Human Fertility Collection - Total Fertility Rate and Mean Age at Birth. Rostock, Germany: Max Planck Institute for Demographic Research.
Ministry of Health and Family Welfare, Government of India, International Institute for Population Sciences, Centers for Disease Control and Prevention (CDC), Johns Hopkins Bloomberg School of Public Health, Research Triangle Institute, Inc., World Health Organization. India Global Adult Tobacco Survey 2009-2010. Atlanta, United States: CDC.
Ministry of Health and Family Welfare, Government of India, United Nations Children's Fund. India Coverage Evaluation Survey 2005.
Ministry of Health and Family Welfare, Government of India, United Nations Children's Fund. India Coverage Evaluation Survey 2007.
Ministry of Health and Family Welfare, Government of India, United Nations Children's Fund. India Coverage Evaluation Survey Data 2009-2010. [Data shared for this analysis]
Ministry of Health and Family Welfare, Government of India, World Health Organization - Regional Office for South-East Asia and Country Office for India, Centers for Disease Control and Prevention. India-Arunachal Pradesh Global Youth Tobacco Survey 2000. New Delhi, India: Ministry of Health and Family Welfare.
Ministry of Health and Family Welfare, Government of India, World Health Organization - Regional Office for South-East Asia and Country Office for India, Centers for Disease Control and Prevention. India-Assam Global Youth Tobacco Survey 2000. New Delhi, India: Ministry of Health and Family Welfare.
Ministry of Health and Family Welfare, Government of India, World Health Organization - Regional Office for South-East Asia and Country Office for India, Centers for Disease Control and Prevention. India-Bihar Global Youth Tobacco Survey 2000. New Delhi, India: Ministry of Health and Family Welfare.
Ministry of Health and Family Welfare, Government of India, World Health Organization - Regional Office for South-East Asia and Country Office for India, Centers for Disease Control and Prevention. India-Goa Global Youth Tobacco Survey 2000. New Delhi, India: Ministry of Health and Family Welfare.
Ministry of Health and Family Welfare, Government of India, World Health Organization - Regional Office for South-East Asia and Country Office for India, Centers for Disease Control and Prevention. India-Maharashtra Global Youth Tobacco Survey 2000. New Delhi, India: Ministry of Health and Family Welfare.
Ministry of Health and Family Welfare, Government of India, World Health Organization - Regional Office for South-East Asia and Country Office for India, Centers for Disease Control and Prevention. India-Manipur Global Youth Tobacco Survey 2000. New Delhi, India: Ministry of Health and Family Welfare.
Ministry of Health and Family Welfare, Government of India, World Health Organization - Regional Office for South-East Asia and Country Office for India, Centers for Disease Control and Prevention. India-Meghalaya Global Youth Tobacco Survey 2000. New Delhi, India: Ministry of Health and Family Welfare.
Ministry of Health and Family Welfare, Government of India, World Health Organization - Regional Office for South-East Asia and Country Office for India, Centers for Disease Control and Prevention. India-Mizoram Global Youth Tobacco Survey 2000. New Delhi, India: Ministry of Health and Family Welfare.
Ministry of Health and Family Welfare, Government of India, World Health Organization - Regional Office for South-East Asia and Country Office for India, Centers for Disease Control and Prevention. India-Nagaland Global Youth Tobacco Survey 2000. New Delhi, India: Ministry of Health and Family Welfare.
Ministry of Health and Family Welfare, Government of India, World Health Organization - Regional Office for South-East Asia and Country Office for India, Centers for Disease Control and Prevention. India-Sikkim Global Youth Tobacco Survey 2000. New Delhi, India: Ministry of Health and Family Welfare.
Ministry of Health and Family Welfare, Government of India, World Health Organization - Regional Office for South-East Asia and Country Office for India, Centers for Disease Control and Prevention. India-Tamil Nadu Global Youth Tobacco Survey 2000. New Delhi, India: Ministry of Health and Family Welfare.
Ministry of Health and Family Welfare, Government of India, World Health Organization - Regional Office for South-East Asia and Country Office for India, Centers for Disease Control and Prevention. India-Tripura Global Youth Tobacco Survey 2000. New Delhi, India: Ministry of Health and Family Welfare.
Ministry of Health and Family Welfare, Government of India, World Health Organization - Regional Office for South-East Asia and Country Office for India, Centers for Disease Control and Prevention. India-West Bengal Global Youth Tobacco Survey 2000. New Delhi, India: Ministry of Health and Family Welfare.
Ministry of Health and Family Welfare, Government of India, World Health Organization - Regional Office for South-East Asia and Country Office for India, Centers for Disease Control and Prevention. India-Uttar Pradesh Global Youth Tobacco Survey 2002. New Delhi, India: Ministry of Health and Family Welfare.

Ministry of Health and Family Welfare, Government of India, World Health Organization - Regional Office for South-East Asia and Country Office for India, Centers for Disease Control and Prevention. India-Uttaranchal Global Youth Tobacco Survey 2002. New Delhi, India: Ministry of Health and Family Welfare.

Ministry of Health and Family Welfare, Government of India, World Health Organization - Regional Office for South-East Asia and Country Office for India, Centers for Disease Control and Prevention. India-Karnataka Global Youth Tobacco Survey 2003. New Delhi, India: Ministry of Health and Family Welfare.

Ministry of Health and Family Welfare, Government of India, World Health Organization - Regional Office for South-East Asia and Country Office for India, Centers for Disease Control and Prevention. India-Rajasthan Global Youth Tobacco Survey 2003. New Delhi, India: Ministry of Health and Family Welfare.

Ministry of Health and Family Welfare, Government of India, World Health Organization - Regional Office for South-East Asia and Country Office for India, Centers for Disease Control and Prevention. India-Gujarat Global Youth Tobacco Survey 2004. New Delhi, India: Ministry of Health and Family Welfare.

Ministry of Health and Family Welfare, Government of India, World Health Organization - Regional Office for South-East Asia and Country Office for India, Centers for Disease Control and Prevention. India-Gujarat with Ahmedabad Global Youth Tobacco Survey 2004. New Delhi, India: Ministry of Health and Family Welfare.

Ministry of Health and Family Welfare, Government of India, World Health Organization - Regional Office for South-East Asia and Country Office for India, Centers for Disease Control and Prevention. India-Haryana Global Youth Tobacco Survey 2004. New Delhi, India: Ministry of Health and Family Welfare.

Ministry of Health and Family Welfare, Government of India, World Health Organization - Regional Office for South-East Asia and Country Office for India, Centers for Disease Control and Prevention. India-Jammu and Kashmir Global Youth Tobacco Survey 2004. New Delhi, India: Ministry of Health and Family Welfare.

Ministry of Health and Family Welfare, Government of India, World Health Organization - Regional Office for South-East Asia and Country Office for India, Centers for Disease Control and Prevention. India- Kerala Global Youth Tobacco Survey 2004. New Delhi, India: Ministry of Health and Family Welfare.

Ministry of Health and Family Welfare, Government of India, World Health Organization - Regional Office for South-East Asia and Country Office for India, Centers for Disease Control and Prevention. India-Madhya Pradesh Global Youth Tobacco Survey 2004. New Delhi, India: Ministry of Health and Family Welfare.

Ministry of Health and Family Welfare, Government of India, World Health Organization - Regional Office for South-East Asia and Country Office for India, Centers for Disease Control and Prevention. India-Punjab Global Youth Tobacco Survey 2004. New Delhi, India: Ministry of Health and Family Welfare.

Ministry of Health and Family Welfare, Government of India, World Health Organization - Regional Office for South-East Asia and Country Office for India, Centers for Disease Control and Prevention. India Global Youth Tobacco Survey 2006. New Delhi, India: Ministry of Health and Family Welfare.

Ministry of Health and Family Welfare, Government of India, World Health Organization - Regional Office for South-East Asia and Country Office for India, Centers for Disease Control and Prevention. India Global Youth Tobacco Survey 2009. New Delhi, India: Ministry of Health and Family Welfare.

Ministry of Statistics and Programme Implementation, Government of India. Employment and Unemployment Survey, National Sample Survey Round 38, January-December 1983. New Delhi, India: Ministry of Statistics and Programme Implementation.

Ministry of Statistics and Programme Implementation, Government of India. Survey on Participation in Education, National Sample Survey Round 42, July 1986- June 1987. New Delhi, India: Ministry of Statistics and Programme Implementation.

Ministry of Statistics and Programme Implementation, Government of India. Consumer Expenditure Survey, National Sample Survey Round 43, July 1987-June 1988. New Delhi, India: Ministry of Statistics and Programme Implementation.

Ministry of Statistics and Programme Implementation, Government of India. Survey on Literacy and Culture, National Sample Survey Round 47, July - December 1991. New Delhi, India: Ministry of Statistics and Programme Implementation.

Ministry of Statistics and Programme Implementation, Government of India. Consumer Expenditure Survey, National Sample Survey Round 49, January-June 1993. New Delhi, India: Ministry of Statistics and Programme Implementation.

Ministry of Statistics and Programme Implementation, Government of India. Employment and Unemployment Survey, National Sample Survey Round 50, July 1993- June 1994. New Delhi, India: Ministry of Statistics and Programme Implementation.

Ministry of Statistics and Programme Implementation, Government of India. Consumer Expenditure Survey, National Sample Survey Round 50, July 1993-June 1994. New Delhi, India: Ministry of Statistics and Programme Implementation.

Ministry of Statistics and Programme Implementation, Government of India. Participation in Education Survey, National Sample Survey Round 52, July 1995-June 1996. New Delhi, India: Ministry of Statistics and Programme Implementation.

Ministry of Statistics and Programme Implementation, Government of India. Common Property Resources, Sanitation & Hygiene Services, National Sample Survey Round 54, January - June 1998. New Delhi, India: Ministry of Statistics and Programme Implementation.

Ministry of Statistics and Programme Implementation, Government of India. Employment and Unemployment Survey, India National Sample Survey Round 55, July 1999-June 2000. New Delhi, India: Ministry of Statistics and Programme Implementation.

Ministry of Statistics and Programme Implementation, Government of India. Survey on Morbidity and Healthcare, National Sample Survey Round 60, January-June 2004. New Delhi, India: Ministry of Statistics and Programme Implementation.

Ministry of Statistics and Programme Implementation, Government of India. Employment and Unemployment Survey, National Sample Survey Round 61, July 2004- June 2005. New Delhi, India: Ministry of Statistics and Programme Implementation.

Ministry of Statistics and Programme Implementation, Government of India. Participation & Expenditure in Education Survey, National Sample Survey Round 64, July 2007- June 2008. New Delhi, India: Ministry of Statistics and Programme Implementation.

Ministry of Statistics and Programme Implementation, Government of India. Consumer Expenditure Survey, National Sample Survey Round 66, July 2009-June 2010. New Delhi, India: Ministry of Statistics and Programme Implementation.

Ministry of Statistics and Programme Implementation, Government of India. Employment and Unemployment Survey, National Sample Survey Round 66, July 2009-June 2010. New Delhi, India: Ministry of Statistics and Programme Implementation.

Ministry of Statistics and Programme Implementation, Government of India. Consumer Expenditure Survey, National Sample Survey Round 68, July 2011- June 2012. New Delhi, India: Ministry of Statistics and Programme Implementation.

Ministry of Statistics and Programme Implementation, Government of India. Employment and Unemployment Survey, National Sample Survey Round 68, July 2011- June 2012. New Delhi, India: Ministry of Statistics and Programme Implementation.

Ministry of Statistics and Programme Implementation, Government of India. Social Consumption Survey on Health and Education, National Sample Survey Round 71, January 2014 - June 2014. New Delhi, India: Ministry of Statistics and Programme Implementation.

Ministry of Statistics and Programme Implementation, Government of India. Household expenditure on services and durable goods survey, National Sample Survey Round 72, July 2014 - June 2015. New Delhi, India: Ministry of Statistics and Programme Implementation.

Ministry of Statistics and Programme Implementation, Government of India. India State Series: Gross State Domestic Product at Factor Cost by Industry of Origin Tables 1980-81 to 1993-94. New Delhi, India: Ministry of Statistics and Programme Implementation.

Ministry of Statistics and Programme Implementation, Government of India. India State Series: Gross State Domestic Product at Factor Cost by Industry of Origin Tables 1993-94 to 2004-05. New Delhi, India: Ministry of Statistics and Programme Implementation.

Ministry of Statistics and Programme Implementation, Government of India. India State Series: Gross State Domestic Product at Factor Cost by Industry of Origin Tables 1999-2000 to 2009-10. New Delhi, India: Ministry of Statistics and Programme Implementation.

Ministry of Statistics and Programme Implementation, Government of India. India State Series: Gross State Domestic Product at Factor Cost by Industry of Origin Tables 2004-05 to 2013-14. New Delhi, India: Ministry of Statistics and Programme Implementation.

Muir CS, Waterhouse J, Mack T, Powell J, Whelan SL, eds. Cancer Incidence in Five Continents, Vol. V. International Agency for Research on Cancer (IARC) Scientific Publications, No. 88. Lyon, France: IARC; 1987.

Murhekar MV, Murhekar KM, Sehgal SC. Age-specific prevalence of hepatitis B infection among the Karen in the Andaman and Nicobar Islands, India. Trop Doct. 2004; 34(2): 117-8.

Murhekar MV, Murhekar KM, Sehgal SC. Seroepidemiology of hepatitis B infection among tribal school children in Andaman and Nicobar Islands, India. Ann Trop Paediatr. 2004; 24(1): 85-8.

Muwonge R, Ramadas K, Sankila R, Thara S, Thomas G, Vinoda J, Sankaranarayanan R. Role of tobacco smoking, chewing and alcohol drinking in the risk of oral cancer in Trivandrum, India: a nested case-control design using incident cancer cases. Oral Oncol. 2008; 44(5): 446-54.

Nandi J, Bhawalkar V, Mody H, Elavia A, Desai PK, Banerjee K. Detection of HIV-1, HBV and HCV antibodies in blood donors from Surat, western India. Vox Sang. 1994; 67(4): 406-7.

National Cancer Registry Programme, National Centre for Disease Informatics and Research, Indian Council of Medical Research (ICMR). India Consolidated Report of Population Based Cancer Registries 2004-2005. New Delhi, India: ICMR; 2008.

National Cancer Registry Programme, National Centre for Disease Informatics and Research, Indian Council of Medical Research (ICMR). India Three-Year Report of Population Based Cancer Registries 2006-2008. New Delhi, India: ICMR; 2010.

National Cancer Registry Programme, National Centre for Disease Informatics and Research, Indian Council of Medical Research (ICMR). India Three-year Report of Population Based Cancer Registries 2009-2011. New Delhi, India: ICMR; 2013.

National Cancer Registry Programme, National Centre for Disease Informatics and Research, Indian Council of Medical Research (ICMR). India Three-year Report of Population Based Cancer Registries 2012-2014. New Delhi, India: ICMR; 2016.
National Cancer Registry Programme, National Centre for Disease Informatics and Research, Indian Council of Medical Research (ICMR). North East Population Based Cancer Registries Report 2005-2006. New Delhi, India: ICMR; 2008.
National Cancer Registry Programme, National Centre for Disease Informatics and Research, Indian Council of Medical Research (ICMR). Population Based Cancer Registry, Ahmedabad Urban Agglomeration Area 2007-2013. New Delhi, India: ICMR; 2016.
National Cancer Registry Programme, National Centre for Disease Informatics and Research, Indian Council of Medical Research (ICMR). Population Based Cancer Registry, Arunachal West 2011-2014. New Delhi, India: ICMR; 2016.
National Cancer Registry Programme, National Centre for Disease Informatics and Research, Indian Council of Medical Research (ICMR). Population Based Cancer Registry, Assam Medical College & Hospital, Dibrugarh 2003-2014. New Delhi, India: ICMR; 2016.
National Cancer Registry Programme, National Centre for Disease Informatics and Research, Indian Council of Medical Research (ICMR). Population Based Cancer Registry, Aurangabad 1982-2014. New Delhi, India: ICMR; 2016.
National Cancer Registry Programme, National Centre for Disease Informatics and Research, Indian Council of Medical Research (ICMR). Population Based Cancer Registry, Bangalore 1982-2012. New Delhi, India: ICMR; 2016.
National Cancer Registry Programme, National Centre for Disease Informatics and Research, Indian Council of Medical Research (ICMR). Barshi Expanded Registry (Osmanabad & Beed District) Data 2007-2012. New Delhi, India: ICMR; 2016.
National Cancer Registry Programme, National Centre for Disease Informatics and Research, Indian Council of Medical Research (ICMR). Population Based Cancer Registry of Bhopal 1986-2013. New Delhi, India: ICMR; 2016.
National Cancer Registry Programme, National Centre for Disease Informatics and Research, Indian Council of Medical Research (ICMR). Population Based Cancer Registry, Cachar District 2007-2014. New Delhi, India: ICMR; 2016.
National Cancer Registry Programme, National Centre for Disease Informatics and Research, Indian Council of Medical Research (ICMR). Population Based Cancer Registry, Chennai 1982-2013. New Delhi, India: ICMR; 2016.
National Cancer Registry Programme, National Centre for Disease Informatics and Research, Indian Council of Medical Research (ICMR). Population Based Cancer Registry, Delhi 1986-2012. New Delhi, India: ICMR; 2016.
National Cancer Registry Programme, National Centre for Disease Informatics and Research, Indian Council of Medical Research (ICMR). Population Based Cancer Registry, Guwahati Kamrup Urban District 2003-2014. New Delhi, India: ICMR; 2016.
National Cancer Registry Programme, National Centre for Disease Informatics and Research, Indian Council of Medical Research (ICMR). Population Based Cancer Registry, Kolkata 2005-2012. New Delhi, India: ICMR; 2016.
National Cancer Registry Programme, National Centre for Disease Informatics and Research, Indian Council of Medical Research (ICMR). District Cancer Registry Kollam, Kerala 1990-2014. New Delhi, India: ICMR; 2016.
National Cancer Registry Programme, National Centre for Disease Informatics and Research, Indian Council of Medical Research (ICMR). Population Based Cancer Registry, Manipur State 2003-2014. New Delhi, India: ICMR; 2016.
National Cancer Registry Programme, National Centre for Disease Informatics and Research, Indian Council of Medical Research (ICMR). Population Based Cancer Registry, Meghalaya 2009-2014. New Delhi, India: ICMR; 2016.
National Cancer Registry Programme, National Centre for Disease Informatics and Research, Indian Council of Medical Research (ICMR). Population Based Cancer Registry, Mizoram State 2003-2014. New Delhi, India: ICMR; 2016.
National Cancer Registry Programme, National Centre for Disease Informatics and Research, Indian Council of Medical Research (ICMR). Population Based Cancer Registry, Mumbai 1982-2012. New Delhi, India: ICMR; 2016.
National Cancer Registry Programme, National Centre for Disease Informatics and Research, Indian Council of Medical Research (ICMR). Population Based Cancer Registry, Nagaland 2009-2014. New Delhi, India: ICMR; 2016.
National Cancer Registry Programme, National Centre for Disease Informatics and Research, Indian Council of Medical Research (ICMR). Population Based Cancer Registry, Nagpur 1982-2013. New Delhi, India: ICMR; 2016.
National Cancer Registry Programme, National Centre for Disease Informatics and Research, Indian Council of Medical Research (ICMR). Population Based Cancer Registry, Naharlagun 2012-2014. New Delhi, India: ICMR; 2016.
National Cancer Registry Programme, National Centre for Disease Informatics and Research, Indian Council of Medical Research (ICMR). Population Based Cancer Registry, Pasighat 2011-2014. New Delhi, India: ICMR; 2016.
National Cancer Registry Programme, National Centre for Disease Informatics and Research, Indian Council of Medical Research (ICMR). Population Based Cancer Registry, Patiala District 2011-2014. New Delhi, India: ICMR; 2016.

National Cancer Registry Programme, National Centre for Disease Informatics and Research, Indian Council of Medical Research (ICMR). Population Based Cancer Registry, Pune 1982-2013. New Delhi, India: ICMR; 2016.

National Cancer Registry Programme, National Centre for Disease Informatics and Research, Indian Council of Medical Research (ICMR). Population Based Cancer Registry, Sikkim State 2003-2014. New Delhi, India: ICMR; 2016.

National Cancer Registry Programme, National Centre for Disease Informatics and Research, Indian Council of Medical Research (ICMR). Population Based Cancer Registry, Thiruvananthapuram 2006-2014. New Delhi, India: ICMR; 2016.

National Cancer Registry Programme, National Centre for Disease Informatics and Research, Indian Council of Medical Research (ICMR). Population Based Cancer Registry, Tripura 2009-2014. New Delhi, India: ICMR; 2016.

National Cancer Registry Programme, National Centre for Disease Informatics and Research, Indian Council of Medical Research (ICMR). Population Based Cancer Registry, Wardha 2010-2014. New Delhi, India: ICMR; 2016.

National Cancer Registry Programme, National Centre for Disease Informatics and Research, Indian Council of Medical Research (ICMR). Population Based Rural Cancer Registry, Barshi (Barshi, Paranda and Bhum) 1987-2014. New Delhi, India: ICMR; 2016.

National Cancer Registry Programme, National Centre for Disease Informatics and Research, Indian Council of Medical Research (ICMR). Rural Cancer Registry, Ahmedabad District 2004-2014. New Delhi, India: ICMR; 2016.

National Institute of Medical Statistics, Indian Council of Medical Research (ICMR), Integrated Disease Surveillance Programme. Non-Communicable Disease Risk Factors Survey Data 2007-2008. New Delhi, India: ICMR. [Data shared for this analysis]

National Institute of Medical Statistics, Indian Council of Medical Research (ICMR), Integrated Disease Surveillance Programme. Non-Communicable Disease Risk Factors Survey Report 2007-2008. New Delhi, India: ICMR.

National Institute of Mental Health and Neurosciences, World Health Organization (WHO). Report on WHO Collaborative Project on Unrecorded Consumption of Alcohol in Karnataka, India 2001-2002. Available from: http://nimhans.ac.in/cam/sites/default/files/Publications/25.pdf

National Nutrition Monitoring Bureau, National Institute of Nutrition (NIN), Indian Council of Medical Research. India Rural First Repeat Survey of Diet and Nutritional Status Data 1988-1990. [Data shared for this analysis]

National Nutrition Monitoring Bureau, National Institute of Nutrition (NIN), Indian Council of Medical Research. India Rural First Repeat Survey of Diet and Nutritional Status Report 1988-1990. Hyderabad, India: NIN.

National Nutrition Monitoring Bureau, National Institute of Nutrition (NIN), Indian Council of Medical Research. India Rural Second Repeat Survey of Diet and Nutritional Status Data 1996-1997. [Data shared for this analysis]

National Nutrition Monitoring Bureau, National Institute of Nutrition (NIN), Indian Council of Medical Research. India Rural Second Repeat Survey of Diet and Nutritional Status Report 1996-1997. Hyderabad, India: NIN.

National Nutrition Monitoring Bureau, National Institute of Nutrition (NIN), Indian Council of Medical Research. India Rural Survey of Diet and Nutritional Status Data 1994-1995. [Data shared for this analysis]

National Nutrition Monitoring Bureau, National Institute of Nutrition (NIN), Indian Council of Medical Research. India Rural Survey of Diet and Nutritional Status Report 1994-1995. Hyderabad, India: NIN.

National Nutrition Monitoring Bureau, National Institute of Nutrition (NIN), Indian Council of Medical Research. India Rural Survey of Diet and Nutritional Status Data 2000-2001. [Data shared for this analysis]

National Nutrition Monitoring Bureau, National Institute of Nutrition (NIN), Indian Council of Medical Research. India Rural Survey of Diet and Nutritional Status Report 2000-2001. Hyderabad, India: NIN.

National Nutrition Monitoring Bureau, National Institute of Nutrition (NIN), Indian Council of Medical Research. India Rural Survey of Diet and Nutritional Status Data 2004-2006. [Data shared for this analysis]

National Nutrition Monitoring Bureau, National Institute of Nutrition (NIN), Indian Council of Medical Research. India Rural Survey of Diet and Nutritional Status Report 2004-2006. Hyderabad, India: NIN.

National Nutrition Monitoring Bureau, National Institute of Nutrition (NIN), Indian Council of Medical Research. India Rural Third Repeat Survey of Diet and Nutritional Status Data 2011-2012. [Data shared for this analysis]

National Nutrition Monitoring Bureau, National Institute of Nutrition (NIN), Indian Council of Medical Research. India Rural Third Repeat Survey of Diet and Nutritional Status Report 2011-2012. Hyderabad, India: NIN.

National Nutrition Monitoring Bureau, National Institute of Nutrition (NIN), Indian Council of Medical Research. India Survey of Diet and Nutritional Status Data 1990-1992. [Data shared for this analysis]

National Nutrition Monitoring Bureau, National Institute of Nutrition (NIN), Indian Council of Medical Research. India Survey of Diet and Nutritional Status Report 1990-1992. Hyderabad, India: NIN.

National Nutrition Monitoring Bureau, National Institute of Nutrition (NIN), Indian Council of Medical Research. India Tribal First Repeat Survey of Diet and Nutritional Status Data 1998-1999. [Data shared for this analysis]

National Nutrition Monitoring Bureau, National Institute of Nutrition (NIN), Indian Council of Medical Research. India Tribal First Repeat Survey of Diet and Nutritional Status Report 1998-1999. Hyderabad, India: NIN.

National Nutrition Monitoring Bureau, National Institute of Nutrition (NIN), Indian Council of Medical Research. India Tribal Second Repeat Survey of Diet and Nutritional Status Data 2007-2008. [Data shared for this analysis]

National Nutrition Monitoring Bureau, National Institute of Nutrition (NIN), Indian Council of Medical Research. India Tribal Second Repeat Survey of Diet and Nutritional Status Report 2007-2008. Hyderabad, India: NIN.

National Nutrition Monitoring Bureau, National Institute of Nutrition (NIN), Indian Council of Medical Research. India Urban Slums Survey of Diet and Nutritional Status Data 1993-1994. [Data shared for this analysis]

National Nutrition Monitoring Bureau, National Institute of Nutrition (NIN), Indian Council of Medical Research. India Urban Slums Survey of Diet and Nutritional Status Report 1993-1994. Hyderabad, India: NIN.

National Nutrition Monitoring Bureau, National Institute of Nutrition, Indian Council of Medical Research. India National Nutrition Monitoring Bureau Eight States Pooled Data 1991-1992. [Data shared for this analysis]

Office of the Registrar General & Census Commissioner, Centre for Global Health Research - University of Toronto. India SRS Special Fertility and Mortality Survey 1998. New Delhi, India: Office of the Registrar General & Census Commissioner; 2005.

Office of the Registrar General & Census Commissioner, Ministry of Home Affairs, Government of India. India Annual Health Survey Data 2010-2011. New Delhi, India: Office of the Registrar General & Census Commissioner.

Office of the Registrar General & Census Commissioner, Ministry of Home Affairs, Government of India. India Annual Health Survey Report 2010-2011. New Delhi, India: Office of the Registrar General & Census Commissioner.

Office of the Registrar General & Census Commissioner, Ministry of Home Affairs, Government of India. India Annual Health Survey Data 2010-2013. New Delhi, India: Office of the Registrar General & Census Commissioner.

Office of the Registrar General & Census Commissioner, Ministry of Home Affairs, Government of India. India Annual Health Survey Report 2010-2013. New Delhi, India: Office of the Registrar General & Census Commissioner.

Office of the Registrar General & Census Commissioner, Ministry of Home Affairs, Government of India. India Annual Health Survey Data 2011-2012. New Delhi, India: Office of the Registrar General & Census Commissioner.

Office of the Registrar General & Census Commissioner, Ministry of Home Affairs, Government of India. India Annual Health Survey Report 2011-2012. New Delhi, India: Office of the Registrar General & Census Commissioner.

Office of the Registrar General & Census Commissioner, Ministry of Home Affairs, Government of India. India Annual Health Survey Data 2012-2013. New Delhi, India: Office of the Registrar General & Census Commissioner.

Office of the Registrar General & Census Commissioner, Ministry of Home Affairs, Government of India. India Annual Health Survey Report 2012-2013. New Delhi, India: Office of the Registrar General & Census Commissioner.

Office of the Registrar General & Census Commissioner, Ministry of Home Affairs, Government of India. India Medical Certification of Cause of Death Report 1997. New Delhi, India: Office of the Registrar General & Census Commissioner.

Office of the Registrar General & Census Commissioner, Ministry of Home Affairs, Government of India. India Medical Certification of Cause of Death Report 2005. New Delhi, India: Office of the Registrar General & Census Commissioner.

Office of the Registrar General & Census Commissioner, Ministry of Home Affairs, Government of India. India Medical Certification of Cause of Death Report 2006. New Delhi, India: Office of the Registrar General & Census Commissioner.

Office of the Registrar General & Census Commissioner, Ministry of Home Affairs, Government of India. India Medical Certification of Cause of Death Report 2008. New Delhi, India: Office of the Registrar General & Census Commissioner.

Office of the Registrar General & Census Commissioner, Ministry of Home Affairs, Government of India. India Medical Certification of Cause of Death Report 2009. New Delhi, India: Office of the Registrar General & Census Commissioner; 2014.

Office of the Registrar General & Census Commissioner, Ministry of Home Affairs, Government of India. India Medical Certification of Cause of Death Report 2010. New Delhi, India: Office of the Registrar General & Census Commissioner; 2014.

Office of the Registrar General & Census Commissioner, Ministry of Home Affairs, Government of India. India Medical Certification of Cause of Death State-Level Tabulations 1990. New Delhi, India: Office of the Registrar General & Census Commissioner.

Office of the Registrar General & Census Commissioner, Ministry of Home Affairs, Government of India. India Medical Certification of Cause of Death State-Level Tabulations 1991. New Delhi, India: Office of the Registrar General & Census Commissioner.

Office of the Registrar General & Census Commissioner, Ministry of Home Affairs, Government of India. India Medical Certification of Cause of Death State-Level Tabulations 1992. New Delhi, India: Office of the Registrar General & Census Commissioner.
Office of the Registrar General & Census Commissioner, Ministry of Home Affairs, Government of India. India Medical Certification of Cause of Death State-Level Tabulations 1993. New Delhi, India: Office of the Registrar General & Census Commissioner.
Office of the Registrar General & Census Commissioner, Ministry of Home Affairs, Government of India. India Medical Certification of Cause of Death State-Level Tabulations 1994. New Delhi, India: Office of the Registrar General & Census Commissioner.
Office of the Registrar General & Census Commissioner, Ministry of Home Affairs, Government of India. India Medical Certification of Cause of Death State-Level Tabulations 1995. New Delhi, India: Office of the Registrar General & Census Commissioner.
Office of the Registrar General & Census Commissioner, Ministry of Home Affairs, Government of India. India Medical Certification of Cause of Death State-Level Tabulations 1996. New Delhi, India: Office of the Registrar General & Census Commissioner.
Office of the Registrar General & Census Commissioner, Ministry of Home Affairs, Government of India. India Medical Certification of Cause of Death State-Level Tabulations 1998. New Delhi, India: Office of the Registrar General & Census Commissioner.
Office of the Registrar General & Census Commissioner, Ministry of Home Affairs, Government of India. India Medical Certification of Cause of Death State-Level Tabulations 1999. New Delhi, India: Office of the Registrar General & Census Commissioner.
Office of the Registrar General & Census Commissioner, Ministry of Home Affairs, Government of India. India Medical Certification of Cause of Death State-Level Tabulations 2000. New Delhi, India: Office of the Registrar General & Census Commissioner.
Office of the Registrar General & Census Commissioner, Ministry of Home Affairs, Government of India. India Medical Certification of Cause of Death State-Level Tabulations 2001. New Delhi, India: Office of the Registrar General & Census Commissioner.
Office of the Registrar General & Census Commissioner, Ministry of Home Affairs, Government of India. India Medical Certification of Cause of Death State-Level Tabulations 2002. New Delhi, India: Office of the Registrar General & Census Commissioner.
Office of the Registrar General & Census Commissioner, Ministry of Home Affairs, Government of India. India Medical Certification of Cause of Death State-Level Tabulations 2003. New Delhi, India: Office of the Registrar General & Census Commissioner.
Office of the Registrar General & Census Commissioner, Ministry of Home Affairs, Government of India. India Medical Certification of Cause of Death State-Level Tabulations 2004. New Delhi, India: Office of the Registrar General & Census Commissioner.
Office of the Registrar General & Census Commissioner, Ministry of Home Affairs, Government of India. India Population and Housing Census 1981. New Delhi, India: Office of the Registrar General & Census Commissioner.
Office of the Registrar General & Census Commissioner, Ministry of Home Affairs, Government of India. India Population and Housing Census 1991. New Delhi, India: Office of the Registrar General & Census Commissioner.
Office of the Registrar General & Census Commissioner, Ministry of Home Affairs, Government of India. India Population and Housing Census 2001. New Delhi, India: Office of the Registrar General & Census Commissioner.
Office of the Registrar General & Census Commissioner, Ministry of Home Affairs, Government of India. India Population and Housing Census 2011. New Delhi, India: Office of the Registrar General & Census Commissioner; 2012.
Office of the Registrar General & Census Commissioner, Ministry of Home Affairs, Government of India. India Sample Registration System Baseline Survey 2014. New Delhi, India: Office of the Registrar General & Census Commissioner.
Office of the Registrar General & Census Commissioner, Ministry of Home Affairs, Government of India. India SRS Statistical Report 1999. New Delhi, India: Office of the Registrar General & Census Commissioner.
Office of the Registrar General & Census Commissioner, Ministry of Home Affairs, Government of India. India SRS Statistical Report 2000. New Delhi, India: Office of the Registrar General & Census Commissioner.
Office of the Registrar General & Census Commissioner, Ministry of Home Affairs, Government of India. India SRS Statistical Report 2001. New Delhi, India: Office of the Registrar General & Census Commissioner; 2005.
Office of the Registrar General & Census Commissioner, Ministry of Home Affairs, Government of India. India SRS Statistical Report 2002. New Delhi, India: Office of the Registrar General & Census Commissioner; 2005.
Office of the Registrar General & Census Commissioner, Ministry of Home Affairs, Government of India. India SRS Statistical Report 2003. New Delhi, India: Office of the Registrar General and Census Commissioner; 2005.
Office of the Registrar General & Census Commissioner, Ministry of Home Affairs, Government of India. India SRS Statistical Report 2004. New Delhi, India: Office of the Registrar General and Census Commissioner; 2006.
Office of the Registrar General & Census Commissioner, Ministry of Home Affairs, Government of India. India SRS Statistical Report 2005. New Delhi, India: Office of the Registrar General and Census Commissioner; 2006.

Office of the Registrar General & Census Commissioner, Ministry of Home Affairs, Government of India. India SRS Statistical Report 2006. New Delhi, India: Office of the Registrar General and Census Commissioner; 2008.
Office of the Registrar General & Census Commissioner, Ministry of Home Affairs, Government of India. India SRS Statistical Report 2007. New Delhi, India: Office of the Registrar General and Census Commissioner; 2008.
Office of the Registrar General & Census Commissioner, Ministry of Home Affairs, Government of India. India SRS Statistical Report 2008. New Delhi, India: Office of the Registrar General and Census Commissioner; 2010.
Office of the Registrar General & Census Commissioner, Ministry of Home Affairs, Government of India. India SRS Statistical Report 2009. New Delhi, India: Office of the Registrar General and Census Commissioner.
Office of the Registrar General & Census Commissioner, Ministry of Home Affairs, Government of India. India SRS Statistical Report 2010. New Delhi, India: Office of the Registrar General and Census Commissioner; 2012.
Office of the Registrar General & Census Commissioner, Ministry of Home Affairs, Government of India. India SRS Statistical Report 2011. New Delhi, India: Office of the Registrar General and Census Commissioner; 2013.
Office of the Registrar General & Census Commissioner, Ministry of Home Affairs, Government of India. India SRS Statistical Report 2012. New Delhi, India: Office of the Registrar General and Census Commissioner; 2013.
Office of the Registrar General & Census Commissioner, Ministry of Home Affairs, Government of India. India SRS Statistical Report 2013. New Delhi, India: Office of the Registrar General and Census Commissioner; 2014.
Office of the Registrar General & Census Commissioner, Ministry of Home Affairs, Government of India. India SRS Statistical Report 2014. New Delhi, India: Office of the Registrar General and Census Commissioner.
Office of the Registrar General & Census Commissioner, Ministry of Home Affairs, Government of India. India SRS Verbal Autopsy 2004-2006. New Delhi, India: Office of the Registrar General and Census Commissioner. [Data shared for this analysis]
Office of the Registrar General & Census Commissioner, Ministry of Home Affairs, Government of India. India SRS Verbal Autopsy Report 2004-2006. New Delhi, India: Office of the Registrar General and Census Commissioner.
Office of the Registrar General & Census Commissioner, Ministry of Home Affairs, Government of India. India SRS Verbal Autopsy 2007-2009. New Delhi, India: Office of the Registrar General and Census Commissioner. [Data shared for this analysis]
Office of the Registrar General & Census Commissioner, Ministry of Home Affairs, Government of India. India SRS Verbal Autopsy Report 2007-2009. New Delhi, India: Office of the Registrar General and Census Commissioner.
Office of the Registrar General & Census Commissioner, Ministry of Home Affairs, Government of India. India SRS Verbal Autopsy 2010-2013. New Delhi, India: Office of the Registrar General and Census Commissioner. [Data shared for this analysis]
Office of the Registrar General & Census Commissioner, Ministry of Home Affairs, Government of India. India SRS Verbal Autopsy Report 2010-2013. New Delhi, India: Office of the Registrar General and Census Commissioner.
Office of the Registrar General & Census Commissioner, Ministry of Home Affairs, Government of India. India Vital Statistics 1980. New Delhi, India: Office of the Registrar General and Census Commissioner.
Office of the Registrar General & Census Commissioner, Ministry of Home Affairs, Government of India. India Vital Statistics 1981. New Delhi, India: Office of the Registrar General and Census Commissioner.
Office of the Registrar General & Census Commissioner, Ministry of Home Affairs, Government of India. India Vital Statistics 1982. New Delhi, India: Office of the Registrar General and Census Commissioner.
Office of the Registrar General & Census Commissioner, Ministry of Home Affairs, Government of India. India Vital Statistics 1983. New Delhi, India: Office of the Registrar General and Census Commissioner.
Office of the Registrar General & Census Commissioner, Ministry of Home Affairs, Government of India. India Vital Statistics 1984. New Delhi, India: Office of the Registrar General and Census Commissioner.
Office of the Registrar General & Census Commissioner, Ministry of Home Affairs, Government of India. India Vital Statistics 1986. New Delhi, India: Office of the Registrar General and Census Commissioner.
Office of the Registrar General & Census Commissioner, Ministry of Home Affairs, Government of India. India Vital Statistics 1987. New Delhi, India: Office of the Registrar General and Census Commissioner.
Office of the Registrar General & Census Commissioner, Ministry of Home Affairs, Government of India. India Vital Statistics 1989. New Delhi, India: Office of the Registrar General and Census Commissioner.
Office of the Registrar General and Census Commissioner, Ministry of Health and Family Welfare, Government of India, National Institute of Health and Family Welfare, Nutrition Foundation of India, National Institute of Nutrition, Indian Council of Medical Research. India Clinical, Anthropometric and Bio-chemical (CAB) Survey Data 2014 [Biomarker Component of Annual Health Survey]. New Delhi, India: Office of the Registrar General and Census Commissioner.

Office of the Registrar General and Census Commissioner, Ministry of Health and Family Welfare, Government of India, National Institute of Health and Family Welfare, Nutrition Foundation of India, National Institute of Nutrition, Indian Council of Medical Research. India Clinical, Anthropometric and Bio-chemical (CAB) Survey Report 2014 [Biomarker Component of Annual Health Survey]. New Delhi, India: Office of the Registrar General and Census Commissioner.
Oommen AM, Abraham VJ, George K, Jose VJ. Prevalence of coronary heart disease in rural and urban Vellore: A repeat cross-sectional survey. Indian Heart J. 2016; 68(4): 473-9.
Parkin D, Whelan S, Ferlay J, Raymond L, Young J. Cancer Incidence in Five Continents VII. Lyon: IARC, 1997.
Parkin D, Whelan S, Ferlay J, Teppo L, Thomas D. Cancer Incidence in Five Continents VIII. Lyon: IARC, 2002.
Parkin DM, Muir CS, Whelan SL, Gao YT, Ferlay J, Powell J, eds. Cancer Incidence in Five Continents, Vol. VI. International Agency for Research on Cancer (IARC) Scientific Publications, No. 120. Lyon, France: IARC; 1992.
Paul SB, Chalamalasetty SB, Vishnubhatla S, Madan K, Gamanagatti SR, Batra Y, Gupta SD, Panda SK, Acharya SK. Clinical Profile, Etiology and Therapeutic Outcome in 324 Hepatocellular Carcinoma Patients at a Tertiary Care Center in India. Oncology. 2009; 77(3-4): 162-71.
Postgraduate Institute of Medical Education & Research (PGIMER) Chandigarh, Tata Memorial Center. Cancer Incidence and Mortality in S.A.S. Nagar District 2013. Chandigarh, India: PGIMER Chandigarh.
Postgraduate Institute of Medical Education & Research (PGIMER), Tata Memorial Center, Civil Hospital-Mansa District. Cancer Incidence and Mortality in Mansa District. Chandigarh, India: PGIMER; 2013.
Postgraduate Institute of Medical Education & Research (PGIMER), Tata Memorial Center, Civil Hospital-Sangur District. Cancer Incidence and Mortality in Sangrur District. Chandigarh, India: PGIMER; 2013.
Public Health Foundation of India. India Cause of Death Estimation Study in Bihar 2011-2014. New Delhi, India: Public Health Foundation of India. [Data shared for this analysis]
Sarin SK, Thakur V, Guptan RC, Saigal S, Malhotra V, Thyagarajan SP, Das BC. Profile of hepatocellular carcinoma in India: an insight into the possible etiologic associations. J Gastroenterol Hepatol. 2001; 16(6): 666-73.
State Bureau of Health Intelligence & Vital Statistics, Directorate of Health Services, Department of Health & Family Welfare, Government of Odisha. India - Odisha Medical Certification of Cause of Death Data 2009. Odisha, India: Department of Health & Family Welfare, Government of Odisha.
State Bureau of Health Intelligence & Vital Statistics, Directorate of Health Services, Department of Health & Family Welfare, Government of Odisha. India - Odisha Medical Certification of Cause of Death Data 2010. Odisha, India: Department of Health & Family Welfare, Government of Odisha
State Bureau of Health Intelligence & Vital Statistics, Directorate of Health Services, Department of Health & Family Welfare, Government of Odisha. India - Odisha Medical Certification of Cause of Death Data 2011. Odisha, India: Department of Health & Family Welfare, Government of Odisha.
State Bureau of Health Intelligence & Vital Statistics, Directorate of Health Services, Department of Health & Family Welfare, Government of Odisha. India - Odisha Medical Certification of Cause of Death Data 2012. Odisha, India: Department of Health & Family Welfare, Government of Odisha.
State Bureau of Health Intelligence & Vital Statistics, Directorate of Health Services, Department of Health & Family Welfare, Government of Odisha. India - Odisha Medical Certification of Cause of Death Data 2013. Odisha, India: Department of Health & Family Welfare, Government of Odisha.
Thakur TS, Goyal A, Sharma V, Gupta ML, Singh S. Incidence of australia antigen (HBs Ag) in Himachal Pradesh. J Commun Dis. 1990; 22(3): 173-7.
Thakur TS, Sharma V, Goyal A, Gupta ML. Seroprevalence of HIV antibodies, Australia antigen and VDRL reactivity in Himachal Pradesh. Indian J Med Sci. 1991; 45(12): 332-5.
The INTERSALT Co-operative Research Group. Appendix tables. Centre-specific results by age and sex. J Hum Hypertens 1989; 3(5):331-407.
Thun MJ, Hannan LM, Adams-Campbell LL, Boffetta P, Buring JE, Feskanich D, Flanders WD, Jee SH, Katanoda K, Kolonel LN, Lee IM, Marugame T, Palmer JR, Riboli E, Sobue T, Avila-Tang E, Wilkens LR, Samet JM. Lung cancer occurrence in never-smokers: an analysis of 13 cohorts and 22 cancer registry studies. PLoS Med. 2008; 5(9): e185.
Tiwari RV, Gupta A, Agrawal A, Gandhi A, Gupta M, Das M. Women and Tobacco Use: Discrepancy in the Knowledge, Belief and Behavior towards Tobacco Consumption among Urban and Rural Women in Chhattisgarh, Central India. Asian Pac J Cancer Prev. 2015; 16(15): 6365-73.
Tiwari S, Hopke PK, Pipal AS, Srivastava AK, Bishta DS, Tiwari S, Singh AK, Sonie VK, Attrie SD. Intra-urban variability of particulate matter (PM2.5 and PM10) and its relationship with optical properties of aerosols over Delhi, India. Atmos Res. 2015; 166: 223-32.

Tobacco Sales Data, Personal Correspondence with Christopher Tan, 1997-2012. [Data shared for this analysis]
Tyagi S, Tyagi A. Possible Correlation of Transfusion Transmitted Diseases with Rh type and ABO Blood Group System. J Clin Diagn Res. 2013; 7.0(9): 1930-1.
U.S. Department of Agriculture (USDA). USDA Global Tobacco Database 1960-2005. Washington DC , United States: USDA.
United States Geological Survey (USGS). USGS Mineral Industry Surveys: World Asbestos Consumption 2003-2007. Reston, United States: USGS; 2009.
United States Geological Survey (USGS). USGS Minerals Yearbook 2013. Reston, United States: USGS.
Virta RL, United States Geological Survey (USGS). Worldwide Asbestos Supply and Consumption Trends from 1990 through 2003: USGS Circular 1298. Reston, United States: USGS; 2006.
Wang B-E, Ma W-M, Sulaiman A, Noer S, Sumoharjo S, Sumarsidi D, Tandon BN, Nakao K, Mishiro S, Miyakawa Y, Akahane Y, Suzuki H. Demographic, clinical, and virological characteristics of hepatocellular carcinoma in Asia: survey of 414 patients from four countries. J Med Virol. 2002; 67(3): 394-400.
Waterhouse J, Muir CS, Correa P, Powell J, eds. Cancer Incidence in Five Continents, Vol. III. International Agency for Research on Cancer (IARC) Scientific Publications, No. 15. Lyon, France: IARC; 1976.
Waterhouse J, Muir CS, Shanmugaratnam K, Powell J, eds. Cancer Incidence in Five Continents, Vol. IV. International Agency for Research on Cancer (IARC) Scientific Publications, No. 42. Lyon, France: IARC; 1982.
Werner GT, Frosner GG, Sareen DK. Prevalence of hepatitis A, B and HIV markers in Punjab. J Indian Med Assoc. 1990; 88(10): 293-4.
World Bank. Survey of Living Conditions 1997-1998, Uttar Pradesh and Bihar. Washington D.C., United States: World Bank.
World Health Organization (WHO). WHO Urban Ambient Air Pollution Database Draft 2016. Geneva, Switzerland: WHO.
World Health Organization. Study on Global Ageing and Adult Health (SAGE) Pilot Study 2005 Data from the Data Archive of Social Research on Aging. Los Altos, United States: Sociometrics Corporation. Available from: http://home.socio.com/age2728.php

3. Burden of all cancers together in the states of India, 2016

States of India (population in 2016)	Incidence rate per 100,000 (95% uncertainty interval)		Death rate per 100,000 (95% uncertainty interval)		DALY rate per 100,000 (95% uncertainty interval)	
	Crude	Age-standardised	Crude	Age-standardised	Crude	Age-standardised
**India (1316 million)**	**81.2 (79.3 to 83.7)**	**106.6 (104.2 to 109.7)**	**61.8 (58.3 to 64.6)**	**86.9 (81.5 to 91.0)**	**1773 (1682 to 1851)**	**2140 (2025 to 2234)**
**Low ETL (626 million)**	**74.7 (72.7 to 77.2)**	**109.6 (106.8 to 113.0)**	**61.0 (56 to 65.2)**	**95.8 (87.4 to 102.8)**	**1780 (1651 to 1903)**	**2369 (2190 to 2533)**
Bihar	53.9 (51.5 to 56.5)	84.7 (80.8 to 88.2)	45.9 (39.3 to 53.0)	76.9 (65.8 to 88.6)	1371 (1177 to 1573)	1905 (1633 to 2190)
Jharkhand	64.3 (62.1 to 67.1)	97.3 (94.1 to 101.2)	49.7 (41.7 to 57.5)	80.9 (67.4 to 93.5)	1468 (1250 to 1683)	1996 (1688 to 2293)
Uttar Pradesh	79.0 (75.9 to 82.6)	118.6 (113.9 to 123.8)	64.9 (57.8 to 72.2)	104.0 (92.5 to 115.7)	1900 (1695 to 2111)	2591 (2306 to 2882)
Rajasthan	72.6 (69.9 to 75.7)	106.8 (102.9 to 111.0)	60.5 (52.8 to 68.0)	95.2 (83.0 to 107.2)	1743 (1526 to 1964)	2338 (2045 to 2634)
Meghalaya	81.4 (78.3 to 85.9)	153.3 (147.9 to 160.5)	70.0 (59.8 to 82.4)	145.8 (124.5 to 170.8)	2124 (1814 to 2502)	3576 (3059 to 4202)
Assam	90.2 (86.8 to 94.4)	134.4 (129.6 to 140.1)	74.1 (64.7 to 84.1)	118.5 (103.6 to 133.6)	2212 (1933 to 2511)	2950 (2584 to 3344)
Chhattisgarh	82.0 (79.2 to 86.1)	112.0 (108.3 to 117.2)	61.4 (53.5 to 70.1)	91.2 (79.2 to 103.9)	1797 (1568 to 2059)	2240 (1956 to 2561)
Madhya Pradesh	83.1 (79.7 to 85.7)	117.2 (113.0 to 120.7)	63.8 (56.3 to 71.8)	96.5 (84.5 to 108.5)	1837 (1628 to 2059)	2380 (2104 to 2675)
Odisha	83.6 (80.4 to 90.7)	103.4 (99.6 to 111.2)	71.7 (62.0 to 83.3)	94.0 (81.5 to 109.4)	2021 (1763 to 2352)	2299 (1998 to 2670)
**Lower-middle ETL (92 million)**	**77.3 (74.4 to 81.7)**	**103.1 (99.3 to 10.3.1)**	**57.9 (52.9 to 63.1)**	**83.7 (76.7 to 83.7)**	**1690 (1548 to 1839)**	**2068 (1896 to 2251)**
Arunachal Pradesh	78.5 (71.4 to 81.3)	145.9 (134.4 to 150.7)	62.9 (52.8 to 74.2)	136.7 (115.4 to 159.7)	1926 (1618 to 2278)	3232 (2715 to 3800)
Mizoram	121.7 (105.9 to 125.8)	186.5 (163.5 to 193.0)	103.2 (86.2 to 123.5)	175.4 (146.6 to 209.2)	2924 (2432 to 3520)	4126 (3447 to 4949)
Nagaland	70.3 (67.7 to 75.2)	127.1 (122.9 to 134.3)	51.9 (43.9 to 61.0)	109.0 (93.3 to 126.7)	1542 (1301 to 1827)	2558 (2169 to 3006)
Uttarakhand	91.0 (86.0 to 96.2)	117.5 (111.4 to 124.3)	70.9 (61.4 to 81.0)	98.0 (85.3 to 111.7)	1976 (1703 to 2260)	2383 (2058 to 2718)
Gujarat	75.8 (72.9 to 80.2)	99.2 (95.3 to 105.1)	55.7 (49.7 to 62.1)	78.9 (70.7 to 87.9)	1647 (1475 to 1827)	1971 (1766 to 2187)
Tripura	69.0 (66.4 to 75.4)	94.1 (90.9 to 101.3)	54.3 (45.0 to 64.7)	77.9 (64.8 to 92.3)	1526 (1259 to 1833)	1923 (1590 to 2295)
Sikkim	74.4 (66.7 to 78.8)	123.1 (112.0 to 129.2)	55.3 (47.2 to 65.2)	104.3 (89 to 122.8)	1615 (1380 to 1909)	2449 (2089 to 2880)
Manipur	64.3 (61.7 to 71.9)	92.4 (88.9 to 102.0)	51.7 (44.0 to 62.1)	81.4 (69.4 to 97.0)	1441 (1217 to 1737)	1915 (1627 to 2298)
**Higher-middle ETL (446 million)**	**86.0 (83.7 to 88.7)**	**105.9 (103.2 to 105.9)**	**62.2 (58.3 to 65.8)**	**82.6 (77.3 to 87.4)**	**1774 (1669 to 1877)**	**2022 (1900 to 2141)**
Haryana	103.3 (97.8 to 111.0)	139.1 (131.7 to 149.1)	75.8 (66.3 to 86.0)	110.6 (97.1 to 125.1)	2144 (1881 to 2435)	2668 (2343 to 3024)
Delhi	102.9 (86.5 to 111.1)	148.6 (126.6 to 159.3)	59.4 (47.2 to 68.3)	98.2 (79.7 to 111.7)	1843 (1447 to 2131)	2435 (1940 to 2804)
Telangana	72.6 (69.4 to 77.3)	88.7 (85.1 to 94.0)	54.4 (45.3 to 64.2)	72.7 (60.4 to 85.6)	1530 (1278 to 1796)	1727 (1443 to 2024)
Andhra Pradesh	76.6 (74.2 to 81.9)	93.3 (90.5 to 99.4)	60.1 (51.8 to 69.9)	79.8 (69.2 to 92.1)	1700 (1465 to 1982)	1912 (1651 to 2221)
Jammu and Kashmir	79.2 (76.4 to 83.9)	113.9 (110.0 to 119.9)	63.6 (55.8 to 71.5)	97.3 (85.4 to 109.1)	1813 (1590 to 2041)	2352 (2062 to 2645)
Karnataka	101.6 (97.8 to 106.2)	123.5 (119.0 to 129.0)	73.1 (64.6 to 82.0)	95.4 (84.2 to 106.6)	2064 (1832 to 2311)	2328 (2067 to 2601)
West Bengal	85.4 (82.5 to 88.1)	105.6 (102.3 to 108.6)	62.7 (55.2 to 70.5)	82.9 (73.2 to 93.1)	1830 (1608 to 2058)	2066 (1825 to 2323)
Maharashtra	80.2 (77.5 to 84.6)	94.1 (91.1 to 99.3)	56.6 (50.5 to 63.2)	71.0 (63.5 to 79.0)	1585 (1422 to 1771)	1750 (1571 to 1953)
UTs* other than Delhi	81.1 (77.9 to 84.8)	115.1 (110.8 to 119.8)	51.3 (42.6 to 61.6)	83.1 (70.2 to 98.7)	1530 (1266 to 1849)	1968 (1642 to 2357)
**High ETL (152 million)**	**96.8 (92.2 to 99.8)**	**101.6 (96.8 to 101.6)**	**66.1 (61.5 to 70.5)**	**73.8 (68.4 to 78.6)**	**1788 (1657 to 1906)**	**1791 (1663 to 1908)**
Himachal Pradesh	91.6 (86.8 to 97.7)	103.3 (98.1 to 109.7)	66.3 (58.2 to 74.9)	78.4 (68.7 to 88.6)	1761 (1540 to 2003)	1890 (1660 to 2145)
Punjab	85.5 (79.9 to 88.6)	97.5 (91.8 to 100.9)	61.7 (54.1 to 69.3)	74.5 (65.7 to 83.5)	1709 (1496 to 1925)	1850 (1626 to 2081)
Tamil Nadu	82.9 (79.3 to 89.5)	89.3 (85.7 to 96.3)	57.4 (50.9 to 65.9)	66.7 (59.2 to 76.3)	1575 (1387 to 1818)	1602 (1419 to 1849)
Goa	97.0 (92.5 to 101.9)	103.1 (98.6 to 108.4)	56.6 (51.2 to 65.4)	65.7 (59.8 to 75.2)	1554 (1397 to 1807)	1565 (1414 to 1813)
Kerala	135.3 (109.2 to 141.6)	125.4 (101.8 to 131)	87.9 (75.1 to 96.9)	84.8 (72.6 to 93.4)	2303 (1951 to 2537)	2079 (1759 to 2296)

*Union territories. DALY is disability-adjusted life-year. ETL is epidemiological transition level.

4. Incident cases, prevalent cases, and deaths for different types of cancers in India by sex, 2016

Types of cancers by the rank of DALYs for both sexes combined in 2016	Both sexes combined			Females			Males		
	Number of incident cases in thousands in 2016 (95% uncertainty interval)	Number of prevalent cases in thousands in 2016 (95% uncertainty interval)	Number of deaths in thousands in 2016 (95% uncertainty interval)	Number of incident cases in thousands in 2016 (95% uncertainty interval)	Number of prevalent cases in thousands in 2016 (95% uncertainty interval)	Number of deaths in thousands in 2016 (95% uncertainty interval)	Number of incident cases in thousands in 2016 (95% uncertainty interval)	Number of prevalent cases in thousands in 2016 (95% uncertainty interval)	Number of deaths in thousands in 2016 (95% uncertainty interval)
**Stomach cancer**	75.1 (72.8 to 77.9)	112.2 (108.6 to 116.3)	77.1 (71.4 to 82.4)	35.5 (33.8 to 37.5)	53.5 (50.9 to 56.6)	36.6 (32.5 to 40.5)	39.6 (38.1 to 41.8)	58.8 (56.5 to 62.0)	40.5 (37.2 to 43.6)
**Breast cancer**	118.1 (106.9 to 130.0)	525.6 (474.0 to 573.5)	62.0 (53.2 to 70.4)	115.9 (104.7 to 127.2)	516.5 (465.5 to 565.1)	60.5 (51.7 to 68.8)	2.2 (1.8 to 2.5)	9.0 (7.1 to 9.9)	1.6 (1.3 to 1.8)
**Lung cancer**	67.0 (63.1 to 71.6)	74.3 (69.6 to 79.5)	69.6 (64.1 to 75.1)	18.3 (17.1 to 23.1)	23.1 (21.5 to 29.0)	19.2 (17.2 to 24.0)	48.7 (44.9 to 50.8)	51.2 (47.0 to 54.0)	50.4 (45.6 to 54.1)
**Lip and oral cavity cancer**	113.3 (105.9 to 117.6)	396.7 (371.1 to 412.3)	59.2 (54.1 to 63.0)	45.6 (43.5 to 49.0)	169.2 (161.2 to 181.2)	19.2 (16.8 to 21.4)	67.7 (61.3 to 71.2)	227.6 (205.2 to 239.9)	40.0 (35.8 to 43.0)
**Pharynx cancer other than nasopharynx**	64.8 (58.3 to 69.7)	151.9 (136.5 to 163.3)	57.5 (50.7 to 62.9)	19.4 (18.2 to 20.6)	46.8 (43.9 to 49.6)	17.0 (14.8 to 18.9)	45.5 (39.7 to 49.9)	105.1 (91.4 to 115.2)	40.5 (34.8 to 45.1)
**Colon and rectum cancer**	63.1 (58.3 to 66.4)	185.4 (170.8 to 194.8)	53.7 (48.7 to 57.9)	31.4 (28.9 to 33.6)	91.6 (84.3 to 97.8)	27.2 (23.4 to 30.2)	31.7 (28.7 to 33.4)	93.8 (84.8 to 98.6)	26.5 (23.8 to 28.7)
**Leukaemia**	33.5 (30.3 to 37.8)	105.3 (96.3 to 120.4)	29.9 (27.2 to 34.7)	13.4 (10.5 to 15.9)	44.9 (35.4 to 53.2)	12.2 (9.8 to 14.4)	20.0 (17.9 to 23.6)	60.4 (53.6 to 72.2)	17.7 (16.0 to 21.5)
**Cervical cancer**	77.3 (68.3 to 95.8)	287.8 (246.6 to 342.3)	37.8 (33.4 to 49.5)	77.3 (68.3 to 95.8)	287.8 (246.6 to 342.3)	37.8 (33.4 to 49.5)	-	-	-
**Oesophageal cancer**	37.0 (36.0 to 37.9)	40.6 (39.3 to 41.8)	38.4 (36.2 to 40.3)	14.5 (14.1 to 14.9)	16.5 (16.0 to 17.1)	15.2 (13.8 to 16.4)	22.5 (21.6 to 23.3)	24.1 (23.1 to 25.0)	23.2 (21.7 to 24.5)
**Brain and nervous system cancer**	23.3 (21.4 to 28.3)	48.7 (44.1 to 57.4)	21.0 (18.8 to 26.0)	9.5 (8.1 to 12.1)	19.5 (16.5 to 24.2)	8.6 (7.2 to 11.2)	13.9 (12.1 to 17.4)	29.2 (25.0 to 35.7)	12.4 (10.7 to 15.7)
**Liver cancer**	30.2 (28.6 to 31.7)	12.0 (10.8 to 13.5)	31.5 (29.4 to 33.5)	9.47 (8.0 to 10.48)	3.6 (3.0 to 4.3)	9.9 (8.3 to 11.2)	20.8 (19.9 to 21.8)	8.4 (7.6 to 9.4)	21.6 (20.0 to 23.1)
**Non-Hodgkin lymphoma**	25.0 (20.7 to 26.3)	68.2 (54.0 to 72.3)	22.8 (19.1 to 24.5)	10.0 (8.1 to 10.5)	27.8 (21.5 to 29.1)	9.2 (7.5 to 10.0)	15.0 (11.6 to 16.1)	40.5 (30.9 to 43.7)	13.6 (10.4 to 14.8)
**Gallbladder and biliary tract cancer**	26.1 (22.7 to 29.0)	20.7 (17.9 to 22.7)	26.1 (22.4 to 28.9)	16.8 (14.5 to 18.0)	13.5 (11.2 to 14.4)	16.8 (14.0 to 18.9)	9.3 (6.3 to 11.4)	7.2 (4.9 to 8.8)	9.2 (6.2 to 11.5)
**Larynx cancer**	31.3 (30.3 to 32.5)	96.0 (92.8 to 99.5)	26.5 (24.7 to 28.2)	5.2 (45.0 to 5.4)	16.0 (15.4 to 16.6)	4.3 (3.8 to 4.7)	26.2 (25.1 to 27.3)	80.0 (76.9 to 83.5)	22.2 (20.5 to 23.8)
**Pancreatic cancer**	22.6 (22.0 to 23.2)	16.5 (16.0 to 17.1)	24.1 (22.7 to 25.4)	10.0 (9.6 to 10.5)	7.3 (7.0 to 7.7)	10.9 (9.7 to 11.9)	12.6 (12.3 to 12.9)	9.2 (9.0 to 9.5)	13.2 (12.4 to 14.0)
**Ovarian cancer**	25.7 (23.8 to 27.4)	76.1 (69.2 to 80.4)	17.3 (15.3 to 19.0)	25.7 (23.8 to 27.4)	76.1 (69.2 to 80.4)	17.3 (15.3 to 19.0)	-	-	-
**Prostate cancer**	32.7 (26.0 to 39.7)	112.2 (86.5 to 137.4)	21.0 (16.1 to 25.8)	-	-	-	32.7 (26.0 to 39.7)	112.2 (86.5 to 137.4)	21.0 (16.1 to 25.8)
**Bladder cancer**	19.0 (18.5 to 19.4)	67.7 (65.9 to 69.3)	11.9 (11.2 to 12.5)	5.1 (4.8 to 5.3)	17.5 (16.6 to 18.2)	3.3 (2.9 to 3.6)	13.9 (13.4 to 14.3)	50.2 (48.3 to 51.9)	8.6 (8.0 to 9.2)
**Nasopharynx cancer**	10.0 (9.2 to 10.5)	32.3 (30.0 to 34.3)	7.6 (7.0 to 8.2)	2.6 (2.3 to 2.8)	8.0 (7.1 to 8.8)	2.3 (2.0 to 2.6)	7.4 (6.8 to 8.1)	24.3 (22.5 to 26.7)	5.3 (4.8 to 6.0)
**Thyroid cancer**	21.0 (20.1 to 22.9)	105.6 (101.1 to 114.6)	7.9 (7.3 to 8.8)	15.6 (14.8 to 16.5)	79.3 (75.3 to 83.6)	5.0 (4.5 to 5.6)	5.5 (5.1 to 6.5)	26.3 (24.7 to 31.2)	2.9 (2.6 to 3.5)
**Multiple myeloma**	8.9 (7.1 to 9.7)	14.6 (10.4 to 16.1)	8.7 (7.0 to 9.6)	4.3 (3.5 to 4.9)	7.0 (5.0 to 7.9)	4.3 (3.4 to 5.0)	4.6 (3.1 to 5.1)	7.6 (4.6 to 8.5)	4.4 (3.1 to 5.0)
**Hodgkin's lymphoma**	6.7 (5.7 to 9.2)	20.0 (16.5 to 26.4)	5.2 (4.4 to 7.3)	2.2 (1.7 to 3.1)	6.7 (4.7 to 9.0)	1.8 (1.4 to 2.5)	4.5 (3.7 to 6.6)	13.3 (10.5 to 18.6)	3.4 (2.8 to 5.1)
**Uterine cancer**	14.5 (13.5 to 15.2)	64.8 (60.5 to 68.2)	7.5 (6.7 to 8.2)	14.5 (13.5 to 15.2)	64.8 (60.5 to 68.2)	7.5 (6.7 to 8.2)	-	-	-
**Kidney cancer**	13.2 (12.7 to 13.6)	46.1 (44.6 to 47.4)	5.6 (5.3 to 5.9)	4.6 (4.4 to 5.0)	17.5 (16.7 to 18.5)	1.7 (1.5 to 1.9)	8.5 (8.2 to 8.9)	28.7 (27.3 to 29.9)	3.9 (3.7 to 4.2)
**Mesothelioma**	2.9 (2.4 to 3.4)	4.3 (3.6 to 5.1)	2.4 (2.0 to 2.9)	0.8 (0.6 to 1.1)	1.1 (0.8 to 1.5)	0.6 (0.4 to 0.8)	2.10 (1.7 to 2.6)	3.1 (2.5 to 4.0)	1.8 (1.5 to 2.4)
**Malignant skin melanoma**	3.2 (2.9 to 3.8)	12.5 (11.3 to 14.8)	2.1 (1.8 to 2.5)	1.5 (1.2 to 1.8)	5.9 (4.6 to 7.0)	0.9 (0.7 to 1.1)	1.7 (1.519 to 2.3)	6.6 (5.8 to 8.9)	1.2 (1.0 to 1.6)
**Testicular cancer**	2.9 (2.7 to 3.1)	13.2 (12.0 to 14.1)	1.3 (1.2 to 1.4)	-	-	-	2.9 (2.7 to 3.1)	13.2 (12.0 to 14.1)	1.3 (1.2 to 1.4)
**Non-melanoma skin cancer**	21.7 (14.1 to 34.9)	2.4 (1.6 to 3.7)	2.4 (2.2 to 2.5)	9.2 (5.7 to 15.2)	0.9 (0.6 to 1.5)	0.5 (0.4 to 0.5)	12.5 (8.4 to 19.6)	1.5 (1.0 to 2.2)	1.9 (1.7 to 2.0)
**Other neoplasms**	79.0 (54.5 to 82.8)	220.4 (147.1 to 232.0)	74.6 (51.8 to 80.0)	33.4 (23.3 to 35.2)	95.4 (64.3 to 100.0)	31.5 (22.0 to 34.5)	45.6 (29.6 to 48.9)	125.0 (79.8 to 134.6)	43.1 (28.1 to 47.2)
**All cancers**	1,069.2 (1,043.3 to 1,101.3)	2,036.8 (1,956.8 to 2,117.0)	812.7 (767.1 to 850.0)	551.7 (534.5 to 582.2)	551.7 (534.5 to 582.2)	381.3 (343.7 to 413.6)	517.5 (499.4 to 531.7)	765.5 (721.4 to 797.2)	431.4 (403.3 to 453.8)

DALY is disability-adjusted life-year.

5. Change in incidence and death rate for all cancers in the states of India grouped by epidemiological transition level, 1990 to 2016

Sex	State groups	Incidence rate per 100,000 (95% uncertainty interval)			Death rate per 100,000 (95% uncertainty interval)		
		Crude incidence rate per 100,000 in 2016	Percent change in crude incidence rate from 1990 to 2016	Percent change in age-standardised incidence rate from 1990 to 2016	Crude death rate per 100,000 in 2016	Percent change in crude death rate from 1990 to 2016	Percent change in age-standardised death rate from 1990 to 2016
**Both sexes combined**	**India**	**81.2 (79.3** to **83.7)**	**28.2% (19.9** to **35.5%)**	**-3.5% (-9.6 to 1.4%)**	**61.8 (58.3** to **64.6)**	**39.7% (30.0** to **50.0%)**	**5.0% (-2.4** to **13.1%)**
	Low ETL	74.7 (72.7 to 77.2)	17.2% (8.5 to 24.7%)	-3.2% (-9.9 to 2.1%)	61.0 (56.0 to 65.2)	37.5% (24.6 to 51.0%)	13.1% (2.2 to 24.5%)
	Lower-middle ETL	77.3 (74.4 to 81.7)	33.7% (26.4 to 40.6%)	-2.5% (-8.0 to 2.6%)	57.9 (52.9 to 63.1)	42.5% (27.5 to 58.6%)	4.6% (-6.1 to 17.2%)
	Higher-middle ETL	86.0 (83.7 to 88.7)	34.0% (24.6 to 42.5%)	-5.6% (-12.1 to -0.4%)	62.2 (58.3 to 65.8)	40.4% (28.1 to 53.0%)	-1.0% (-9.5 to 8.7%)
	High ETL	96.8 (92.2 to 99.8)	53.2% (43.5 to 63.3%)	0.9% (-5.4 to 7.7%)	66.1 (61.5 to 70.5)	45.5% (31.9 to 60.1%)	-4.5% (-12.6 to 5.1%)
**Females**	**India**	**87.0 (84.4** to **91.8)**	**23.3% (13.8** to **34.8%)**	**-7.7% (-14.6 to -0.1%)**	**60.1 (54.2** to **65.2)**	**32.5% (20.4** to **47.1%)**	**-1.9% (-11.4 to 9.1%)**
	Low ETL	79.7 (76.4 to 85.1)	10.1% (-4.2 to 21.6%)	-9.1% (-20.3 to -1.2%)	60.1 (52.4 to 67.1)	28.4% (11.8 to 48.6%)	4.7% (-8.4 to 20.4%)
	Lower-middle ETL	75.8 (72.3 to 84.5)	32.9% (23.4 to 45.6%)	-3.9% (-10.7 to 5.4%)	52.1 (45.1 to 60.1)	38.4% (17.7 to 63.1%)	0.1% (-14.5 to 18.4%)
	Higher-middle ETL	94.0 (91.0 to 98.8)	29.8% (18.8 to 43.6%)	-9.4% (-16.8 to -1.0%)	61.5 (55.6 to 66.1)	33.6% (18.6 to 50.8%)	-7.5% (-18.5 to 5.0%)
	High ETL	102.3 (95.4 to 107.0)	53.3% (40.3 to 74.4%)	0.2% (-7.9 to 13.3%)	61.0 (54.6 to 67.1)	42.3% (22.7 to 63.9%)	-8.2% (-20.6 to 6.0%)
**Males**	**India**	**75.9 (73.2** to **78.0)**	**33.9% (25.4** to **39.5%)**	**1.8% (-4.5 to 6.1%)**	**63.3 (59.1** to **66.5)**	**46.7% (35.0** to **58.4%)**	**12.3% (2.9 to 23.3%)**
	Low ETL	70.0 (67.1 to 72.3)	25.6% (17.9 to 31.3%)	3.7% (-2.6 to 8.3%)	66.6 (55.9 to 61.7)	46.8% (31.5 to 63.2%)	21.8% (9.0 to 36.3%)
	Lower-middle ETL	78.7 (75.3 to 82.2)	34.4% (25.7 to 41.0%)	-0.5% (-6.9 to 4.4%)	70.5 (56.8 to 63.2)	45.7% (25.6 to 69.4%)	9.3% (-5.8 to 27.0%)
	Higher-middle ETL	78.5 (75.9 to 80.5)	39.1% (29.3 to 46.0%)	-0.8% (-7.5 to 4.2%)	67.3 (58.2 to 62.8)	47.3% (31.9 to 63.3%)	6.3% (-5.3 to 19.4%)
	High ETL	91.3 (88.0 to 94.3)	53.1% (44.1 to 60.2%)	2.8% (-3.1 to 7.8%)	77.9 (65.5 to 71.2)	48.4% (31.9 to 67.6%)	0.4% (-10.9 to 13.8%)

Incidence rate and death rate per 100,000. ETL is epidemiological transition level.

6. Crude annual death rate of all cancers together in the states of India, 1990 and 2016

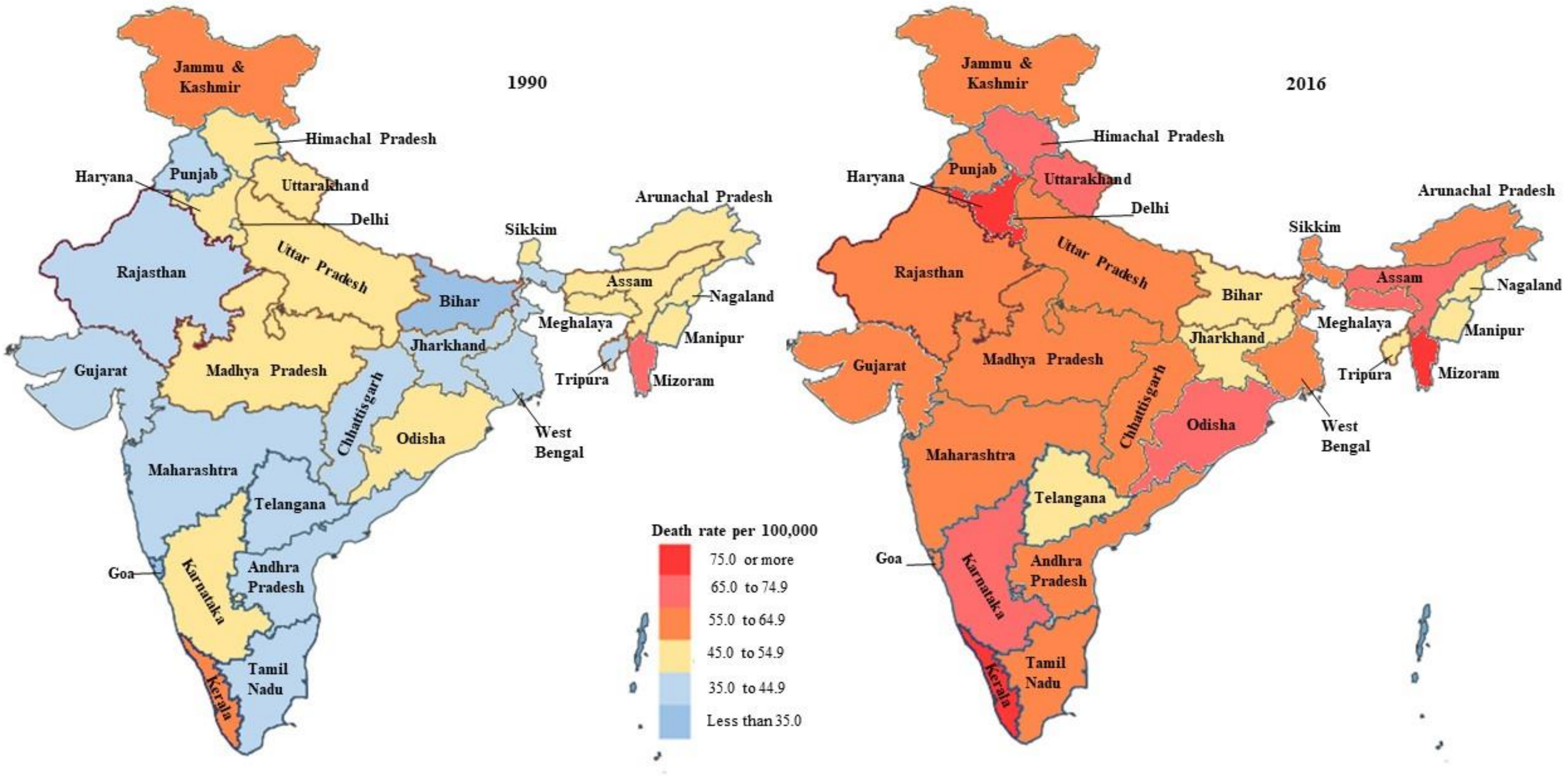

7. **Age-specific DALY rate for different types of cancers in the states of India by sex, 2016**

Both sexes combined

Both sexes combined										
**Age group (in years)**	**Stomach cancer (95% uncertainty interval)**	**Breast cancer (95% uncertainty interval)**	**Tracheal, bronchus, and lung cancer (95% uncertainty interval)**	**Lip and oral cavity cancer (95% uncertainty interval)**	**Pharynx cancer other than nasopharynx (95% uncertainty interval)**	**Colon and rectum cancer (95% uncertainty interval)**	**Leukaemia (95% uncertainty interval)**	**Cervical cancer (95% uncertainty interval)**	**Oesophageal cancer (95% uncertainty interval)**	**Brain and nervous system cancer (95% uncertainty interval)**
**<1**	0.0 (0.0 to 0.0)	0.0 (0.0 to 0.0)	0.0 (0.0 to 0.0)	0.0 (0.0 to 0.0)	0.0 (0.0 to 0.0)	0.0 (0.0 to 0.0)	77.5 (66.3 to 91.7)	0.0 (0.0 to 0.0)	0.0 (0.0 to 0.0)	24.7 (20.6 to 31.4)
**1 to 4**	0.0 (0.0 to 0.0)	0.0 (0.0 to 0.0)	0.0 (0.0 to 0.0)	0.0 (0.0 to 0.0)	0.0 (0.0 to 0.0)	0.0 (0.0 to 0.0)	77.1 (59.8 to 94.3)	0.0 (0.0 to 0.0)	0.0 (0.0 to 0.0)	28.7 (21.8 to 40.1)
**5 to 9**	0.0 (0.0 to 0.0)	0.0 (0.0 to 0.0)	0.0 (0.0 to 0.0)	0.0 (0.0 to 0.0)	0.0 (0.0 to 0.0)	0.0 (0.0 to 0.0)	70.0 (54.0 to 87.7)	0.0 (0.0 to 0.0)	0.0 (0.0 to 0.0)	35.5 (27.0 to 48.2)
**10 to 14**	0.0 (0.0 to 0.0)	0.0 (0.0 to 0.0)	0.0 (0.0 to 0.0)	0.0 (0.0 to 0.0)	0.0 (0.0 to 0.0)	0.0 (0.0 to 0.0)	74.7 (62.1 to 91.6)	0.0 (0.0 to 0.0)	0.0 (0.0 to 0.0)	38.2 (30.9 to 50.3)
**15 to 19**	16.3 (14.6 to 18.2)	6.1 (4.3 to 7.3)	8.8 (7.6 to 10.2)	9.3 (8.1 to 10.6)	9.5 (7.6 to 11.2)	8.7 (7.5 to 10.1)	88.9 (78.6 to 109.8)	2.3 (1.0 to 3.0)	3.7 (3.2 to 4.2)	33.9 (28.7 to 43.8)
**20 to 24**	33.7 (30.2 to 37.6)	17.1 (12.8 to 21.1)	12.9 (11.5 to 14.5)	18.7 (16.8 to 20.7)	17.4 (14.5 to 20.0)	16.0 (13.8 to 18.0)	68.3 (61.1 to 81.2)	10.7 (5.1 to 13.5)	9.1 (8.3 to 10.0)	35.7 (31.4 to 45.4)
**25 to 29**	48.7 (44.1 to 54.5)	40.5 (32.4 to 47.7)	22.0 (19.6 to 24.6)	32.3 (28.5 to 36.2)	25.2 (21.3 to 28.7)	24.3 (21.4 to 27.0)	60.5 (54.4 to 71.7)	22.9 (12.8 to 27.6)	13.3 (12.2 to 14.6)	41.0 (36.1 to 52.8)
**30 to 34**	82.1 (74.6 to 90.9)	85.3 (71.0 to 99.2)	39.6 (35.7 to 44.6)	59.1 (52.4 to 66.7)	44.6 (38.0 to 49.8)	47.8 (41.7 to 53.3)	68.7 (61.4 to 79.5)	55.6 (36.0 to 65.9)	22.6 (20.7 to 24.6)	51.2 (45.0 to 66.0)
**35 to 39**	140.8 (127.7 to 155.7)	182.1 (154.2 to 213.1)	70.4 (63.9 to 78.3)	107.2 (96.1 to 119.1)	85.2 (73.7 to 95.4)	69.5 (61.9 to 76.5)	78.1 (68.8 to 91.9)	110.5 (84.9 to 130.5)	47.2 (43.5 to 50.8)	70.9 (62.8 to 91.8)
**40 to 44**	228.4 (210.5 to 247.2)	271.2 (232.9 to 317.5)	124.5 (114.0 to 138.0)	189.2 (170.4 to 207.5)	157.8 (137.3 to 175.9)	105.6 (95.0 to 115.4)	97.1 (86.9 to 113.8)	179.2 (153.2 to 215.4)	90.2 (84.0 to 97.4)	85.4 (75.7 to 111.2)
**45 to 49**	313.0 (288.8 to 340.2)	355.7 (304.0 to 419.2)	218.6 (201.1 to 238.0)	270.1 (246.2 to 293.9)	250.0 (219.6 to 277.3)	199.9 (181.4 to 216.8)	112.3 (100.6 to 132.3)	249.3 (217.3 to 317.1)	159.6 (148.9 to 170.1)	98.8 (86.8 to 125.0)
**50 to 54**	417.2 (384.5 to 452.3)	488.0 (425.6 to 563.4)	360.4 (329.5 to 397.0)	380.3 (344.0 to 414.6)	363.6 (321.3 to 401.8)	268.2 (244.3 to 291.5)	135.0 (116.7 to 155.2)	307.7 (266.9 to 406.9)	239.2 (223.4 to 255.2)	117.4 (104.1 to 150.7)
**55 to 59**	531.5 (491.0 to 575.1)	507.2 (433.6 to 588.8)	515.4 (477.0 to 561.2)	465.7 (422.8 to 506.0)	445.3 (392.6 to 490.9)	343.5 (312.8 to 373.4)	156.9 (136.7 to 181.4)	322.3 (278.5 to 458.8)	296.4 (279.6 to 315.4)	137.6 (121.7 to 176.5)
**60 to 64**	615.7 (570.4 to 664.8)	454.0 (384.7 to 527.8)	631.5 (575.3 to 689.2)	525.8 (475.7 to 570.7)	546.7 (470.6 to 605.5)	420.2 (378.6 to 456.6)	168.7 (147.0 to 194.4)	306.2 (263.6 to 432.2)	316.6 (294.1 to 339.6)	131.8 (115.9 to 165.3)
**65 to 69**	699.7 (639.7 to 757.5)	442.5 (369.9 to 517.5)	765.1 (696.4 to 836.8)	525.0 (478.0 to 567.9)	524.8 (454.8 to 586.0)	503.6 (451.0 to 550.7)	184.4 (160.5 to 210.0)	270.1 (230.3 to 372.7)	356.7 (328.9 to 383.3)	137.1 (119.9 to 172.7)
**70 to 74**	667.1 (600.5 to 725.1)	409.9 (336.4 to 471.8)	780.7 (707.1 to 848.3)	494.6 (443.8 to 538.7)	518.9 (447.9 to 577.7)	546.4 (485.1 to 597.4)	179.5 (156.6 to 205.7)	242.0 (200.8 to 344.5)	353.8 (324.8 to 379.4)	109.3 (93.8 to 131.2)
**75 to 79**	667.3 (598.2 to 731.1)	386.6 (313.3 to 447.0)	721.3 (651.8 to 782.6)	434.2 (387.7 to 473.0)	422.0 (361.3 to 473.3)	624.4 (557.9 to 681.7)	161.0 (137.8 to 181.6)	172.9 (143.4 to 235.9)	370.8 (338.5 to 398.3)	96.5 (78.5 to 114.0)
**80+**	680.1 (592.1 to 752.3)	361.5 (289.3 to 425.5)	504.2 (455.2 to 549.2)	427.3 (375.8 to 480.0)	406.4 (349.6 to 463.0)	525.2 (465.8 to 585.4)	123.9 (108.7 to 144.4)	133.4 (108.8 to 175.9)	309.3 (281.9 to 331.7)	63.1 (51.2 to 76.5)

DALY is disability-adjusted life-year, which is presented as rate per 100,000.

Both sexes combined

Age group (in years)	Liver cancer (95% uncertainty interval)	Non-Hodgkin lymphoma (95% uncertainty interval)	Gallbladder and biliary tract cancer (95% uncertainty interval)	Larynx cancer (95% uncertainty interval)	Pancreatic cancer (95% uncertainty interval)	Ovarian cancer (95% uncertainty interval)	Prostate cancer (95% uncertainty interval)	Bladder cancer (95% uncertainty interval)	Nasopharynx cancer (95% uncertainty interval)	Thyroid cancer (95% uncertainty interval)
**<1**	0.0 (0.0 to 0.0)	31.2 (26.1 to 39.8)	0.0 (0.0 to 0.0)	0.0 (0.0 to 0.0)	0.0 (0.0 to 0.0)	0.0 (0.0 to 0.0)	0.0 (0.0 to 0.0)	0.0 (0.0 to 0.0)	0.0 (0.0 to 0.0)	0.0 (0.0 to 0.0)
**1 to 4**	0.0 (0.0 to 0.0)	18.8 (13.8 to 25.5)	0.0 (0.0 to 0.0)	0.0 (0.0 to 0.0)	0.0 (0.0 to 0.0)	0.0 (0.0 to 0.0)	0.0 (0.0 to 0.0)	0.0 (0.0 to 0.0)	0.0 (0.0 to 0.0)	0.0 (0.0 to 0.0)
**5 to 9**	2.4 (1.9 to 3.0)	14.4 (10.8 to 18.5)	0.0 (0.0 to 0.0)	0.0 (0.0 to 0.0)	0.0 (0.0 to 0.0)	0.0 (0.0 to 0.0)	0.0 (0.0 to 0.0)	0.0 (0.0 to 0.0)	1.0 (0.8 to 1.2)	0.0 (0.0 to 0.0)
**10 to 14**	1.9 (1.6 to 2.3)	16.8 (13.7 to 19.9)	0.0 (0.0 to 0.0)	0.0 (0.0 to 0.0)	0.0 (0.0 to 0.0)	0.0 (0.0 to 0.0)	0.0 (0.0 to 0.0)	0.0 (0.0 to 0.0)	3.3 (2.7 to 4.1)	1.2 (0.9 to 1.5)
**15 to 19**	2.8 (2.4 to 3.1)	30.2 (25.2 to 34.4)	1.0 (0.9 to 1.2)	1.7 (1.5 to 1.9)	1.8 (1.6 to 2.0)	4.5 (3.9 to 5.3)	0.4 (0.4 to 0.7)	1.0 (0.9 to 1.1)	4.3 (3.8 to 4.9)	4.9 (4.2 to 6.0)
**20 to 24**	6.1 (5.5 to 6.7)	35.5 (30.1 to 39.7)	2.6 (2.2 to 3.0)	2.8 (2.6 to 3.2)	3.4 (3.1 to 3.7)	5.9 (5.2 to 6.7)	0.6 (0.5 to 0.9)	1.4 (1.3 to 1.6)	3.9 (3.4 to 4.4)	8.6 (7.7 to 10.2)
**25 to 29**	9.2 (8.3 to 10.2)	33.3 (27.7 to 37.1)	7.0 (5.9 to 7.9)	3.9 (3.5 to 4.4)	5.2 (4.8 to 5.6)	9.4 (8.3 to 10.5)	0.9 (0.7 to 1.4)	1.7 (1.6 to 1.9)	3.9 (3.5 to 4.4)	10.2 (9.2 to 11.7)
**30 to 34**	19.1 (17.3 to 21.1)	40.0 (32.4 to 44.3)	18.4 (15.4 to 20.5)	10.4 (9.3 to 11.8)	11.0 (10.2 to 11.9)	14.5 (12.8 to 16.0)	1.2 (0.9 to 1.9)	3.4 (3.1 to 3.7)	6.5 (5.8 to 7.2)	12.1 (10.9 to 14.1)
**35 to 39**	36.2 (33.0 to 39.7)	49.8 (39.8 to 55.1)	43.2 (35.9 to 48.6)	23.4 (21.3 to 26.0)	20.3 (19.0 to 21.8)	30.2 (26.4 to 33.9)	1.8 (1.5 to 2.9)	6.5 (6.0 to 7.0)	12.4 (11.1 to 13.8)	12.9 (11.5 to 14.9)
**40 to 44**	61.0 (55.6 to 66.9)	62.3 (49.1 to 68.8)	85.9 (72.1 to 96.6)	56.7 (51.6 to 62.0)	38.0 (35.6 to 40.3)	55.8 (48.7 to 62.9)	3.7 (3.1 to 5.5)	13.9 (12.8 to 15.1)	26.6 (24.0 to 29.3)	20.8 (18.6 to 23.7)
**45 to 49**	100.7 (91.6 to 110.4)	79.1 (61.4 to 87.6)	129.9 (108.6 to 145.1)	102.3 (93.6 to 110.8)	65.2 (61.6 to 69.2)	91.0 (79.1 to 101.5)	9.4 (7.9 to 12.9)	21.9 (20.3 to 23.5)	39.4 (35.3 to 43.3)	29.7 (26.7 to 33.2)
**50 to 54**	167.3 (151.0 to 184.0)	110.3 (85.8 to 121.8)	181.1 (151.9 to 202.4)	170.2 (156.3 to 185.3)	105.7 (99.2 to 113.2)	139.1 (119.3 to 158.4)	25.1 (19.9 to 32.9)	40.8 (38.3 to 43.5)	61.6 (55.1 to 67.4)	47.8 (42.7 to 53.7)
**55 to 59**	230.5 (212.1 to 252.3)	138.3 (105.8 to 153.1)	202.7 (171.3 to 226.3)	227.9 (208.8 to 248.2)	151.7 (143.2 to 160.1)	141.6 (122.1 to 157.1)	54.9 (41.5 to 70.8)	57.9 (54.5 to 61.4)	73.9 (67.3 to 80.9)	57.0 (51.7 to 64.7)
**60 to 64**	286.9 (261.2 to 311.5)	176.6 (142.1 to 193.2)	245.2 (204.7 to 276.4)	267.3 (244.1 to 290.7)	206.4 (193.9 to 219.8)	152.7 (132.4 to 170.9)	121.9 (89.6 to 152.6)	94.6 (87.7 to 101.2)	71.7 (63.8 to 80.1)	67.1 (60.7 to 76.5)
**65 to 69**	330.3 (298.8 to 361.6)	196.0 (158.6 to 215.1)	239.2 (201.6 to 272.9)	299.4 (272.5 to 328.4)	257.2 (239.5 to 274.9)	149.8 (132.6 to 167.3)	205.8 (154.5 to 253.9)	123.4 (113.4 to 133.1)	63.5 (57.1 to 70.8)	74.8 (67.2 to 85.6)
**70 to 74**	336.4 (303.7 to 365.6)	182.2 (148.1 to 199.7)	215.8 (186.5 to 246.0)	256.7 (234.2 to 280.5)	292.9 (271.2 to 314.3)	145.0 (124.4 to 162.8)	315.4 (238.8 to 399.2)	143.5 (131.7 to 155.1)	52.6 (47.5 to 58.4)	71.0 (63.7 to 81.0)
**75 to 79**	319.6 (289.1 to 347.2)	164.5 (136.8 to 180.4)	178.2 (152.0 to 205.1)	197.8 (180.9 to 213.8)	282.3 (259.5 to 303.7)	114.9 (98.9 to 128.9)	452.1 (332.2 to 556.6)	171.1 (157.3 to 184.4)	34.9 (31.3 to 38.7)	59.3 (52.5 to 67.0)
**80+**	244.5 (217.3 to 267.4)	142.0 (120.6 to 155.8)	150.7 (127.8 to 173.7)	167.1 (150.3 to 181.9)	236.1 (211.7 to 256.0)	79.9 (65.7 to 92.0)	431.0 (319.8 to 534.5)	187.5 (170.6 to 202.8)	24.8 (22.1 to 27.5)	49.4 (43.4 to 56.1)

DALY is disability-adjusted life-year, which is presented as rate per 100,000.

Both sexes combined

Age group (in years)	Multiple myeloma (95% uncertainty interval)	Hodgkin's lymphoma (95% uncertainty interval)	Uterine cancer (95% uncertainty interval)	Kidney cancer (95% uncertainty interval)	Mesothelioma (95% uncertainty interval)	Malignant skin melanoma (95% uncertainty interval)	Testicular cancer (95% uncertainty interval)	Non-melanoma skin cancer (95% uncertainty interval)
**<1**	0.0 (0.0 to 0.0)	1.9 (1.5 to 2.8)	0.0 (0.0 to 0.0)	5.9 (5.2 to 6.8)	0.0 (0.0 to 0.0)	0.0 (0.0 to 0.0)	0.0 (0.0 to 0.0)	0.0 (0.0 to 0.0)
**1 to 4**	0.0 (0.0 to 0.0)	3.1 (2.3 to 5.2)	0.0 (0.0 to 0.0)	5.4 (4.3 to 6.6)	0.0 (0.0 to 0.0)	0.0 (0.0 to 0.0)	0.0 (0.0 to 0.0)	0.0 (0.0 to 0.0)
**5 to 9**	0.0 (0.0 to 0.0)	13.4 (9.4 to 20.1)	0.0 (0.0 to 0.0)	2.1 (1.7 to 2.6)	0.0 (0.0 to 0.0)	0.0 (0.0 to 0.0)	0.0 (0.0 to 0.0)	0.0 (0.0 to 0.0)
**10 to 14**	0.0 (0.0 to 0.0)	13.6 (10.4 to 21.6)	0.0 (0.0 to 0.0)	1.0 (0.8 to 1.1)	0.0 (0.0 to 0.0)	0.0 (0.0 to 0.0)	0.0 (0.0 to 0.0)	0.0 (0.0 to 0.0)
**15 to 19**	2.4 (2.0 to 3.6)	17.0 (13.7 to 25.2)	0.4 (0.3 to 0.4)	1.0 (0.9 to 1.1)	1.0 (0.8 to 1.3)	1.4 (1.1 to 2.1)	2.9 (2.4 to 3.5)	0.7 (0.7 to 0.8)
**20 to 24**	3.5 (3.0 to 4.9)	15.0 (12.4 to 21.7)	0.7 (0.6 to 0.8)	1.5 (1.4 to 1.6)	1.3 (0.9 to 1.7)	2.1 (1.8 to 3.3)	5.9 (5.0 to 6.8)	0.7 (0.7 to 0.8)
**25 to 29**	3.4 (3.0 to 4.5)	14.2 (11.8 to 20.2)	1.1 (0.9 to 1.2)	2.3 (2.1 to 2.5)	2.0 (1.4 to 2.6)	2.9 (2.5 to 4.3)	7.4 (6.4 to 8.7)	0.8 (0.7 to 0.8)
**30 to 34**	6.4 (5.5 to 8.1)	15.1 (12.6 to 21.9)	2.4 (2.1 to 2.8)	3.7 (3.4 to 4.0)	2.5 (1.9 to 3.1)	3.0 (2.6 to 4.2)	8.4 (7.3 to 9.7)	1.2 (1.1 to 1.3)
**35 to 39**	11.2 (9.3 to 13.5)	16.3 (13.5 to 23.4)	6.4 (5.6 to 7.3)	7.0 (6.5 to 7.6)	4.4 (3.4 to 5.5)	5.6 (4.9 to 7.7)	8.2 (7.0 to 9.4)	1.9 (1.8 to 2.1)
**40 to 44**	19.7 (15.7 to 22.4)	18.9 (15.5 to 27.5)	13.7 (12.0 to 15.6)	10.8 (9.9 to 11.7)	6.5 (5.3 to 8.0)	7.0 (6.3 to 9.1)	6.2 (5.3 to 7.3)	3.0 (2.8 to 3.3)
**45 to 49**	29.8 (22.8 to 33.4)	22.8 (18.9 to 32.2)	25.0 (22.1 to 28.1)	19.2 (17.7 to 20.7)	9.6 (7.8 to 12.1)	8.0 (7.1 to 10.1)	5.7 (4.9 to 6.6)	4.7 (4.3 to 5.0)
**50 to 54**	50.4 (36.5 to 56.5)	25.1 (20.7 to 35.0)	45.3 (39.5 to 50.8)	30.2 (27.9 to 32.9)	14.4 (11.7 to 17.9)	11.0 (9.5 to 13.0)	5.4 (4.6 to 6.4)	7.1 (6.6 to 7.6)
**55 to 59**	66.5 (47.6 to 74.6)	29.7 (25.0 to 41.4)	58.6 (51.8 to 66.1)	46.3 (43.0 to 50.8)	19.5 (15.8 to 24.4)	14.4 (12.4 to 17.4)	4.3 (3.7 to 5.1)	10.5 (9.8 to 11.3)
**60 to 64**	79.2 (58.8 to 88.3)	29.4 (24.3 to 41.8)	78.0 (68.2 to 88.9)	52.1 (48.5 to 56.3)	19.3 (15.5 to 23.9)	13.8 (11.5 to 15.5)	4.7 (3.9 to 5.6)	16.9 (15.7 to 18.0)
**65 to 69**	88.5 (68.4 to 98.9)	27.0 (22.0 to 37.5)	86.7 (75.1 to 98.9)	58.4 (54.3 to 63.2)	20.7 (16.9 to 25.5)	16.0 (13.5 to 18.5)	4.9 (4.1 to 5.8)	22.7 (21.0 to 24.2)
**70 to 74**	81.3 (63.6 to 90.5)	22.1 (17.8 to 30.7)	75.4 (64.7 to 85.9)	51.8 (47.8 to 56.1)	22.3 (18.2 to 27.6)	16.0 (13.4 to 18.7)	5.6 (4.7 to 6.6)	25.1 (23.1 to 26.9)
**75 to 79**	78.9 (62.4 to 90.0)	20.7 (16.6 to 28.8)	65.8 (55.3 to 75.3)	51.4 (47.0 to 55.9)	21.1 (16.8 to 26.2)	16.1 (13.1 to 18.2)	3.4 (2.8 to 4.0)	30.7 (28.4 to 32.8)
**80+**	68.5 (55.8 to 83.5)	13.2 (10.8 to 18.4)	54.0 (45.5 to 61.7)	39.6 (35.8 to 43.7)	16.4 (12.4 to 20.1)	20.1 (15.7 to 22.7)	5.6 (4.7 to 6.4)	36.6 (33.2 to 39.7)

DALY is disability-adjusted life-year, which is presented as rate per 100,000.

Females

Age group (in years)	Breast cancer (95% uncertainty interval)	Cervical cancer (95% uncertainty interval)	Stomach cancer (95% uncertainty interval)	Colon and rectum cancer (95% uncertainty interval)	Lip and oral cavity cancer (95% uncertainty interval)	Ovarian cancer (95% uncertainty interval)	Tracheal, bronchus, and lung cancer (95% uncertainty interval)	Leukaemia (95% uncertainty interval)	Gallbladder and biliary tract cancer (95% uncertainty interval)	Pharynx cancer other than nasopharynx (95% uncertainty interval)	Oesophageal cancer (95% uncertainty interval)	Brain and nervous system cancer (95% uncertainty interval)	Non-Hodgkin lymphoma (95% uncertainty interval)
	Females												
**<1**	0.0 (0.0-0.0)	0.0 (0.0-0.0)	0.0 (0.0-0.0)	0.0 (0.0-0.0)	0.0 (0.0-0.0)	0.0 (0.0-0.0)	0.0 (0.0-0.0)	74.5 (59.7-94.9)	0.0 (0.0-0.0)	0.0 (0.0-0.0)	0.0 (0.0-0.0)	22.8 (18.3-30.9)	29.2 (24.2-38.4)
**1 to 4**	0.0 (0.0-0.0)	0.0 (0.0-0.0)	0.0 (0.0-0.0)	0.0 (0.0-0.0)	0.0 (0.0-0.0)	0.0 (0.0-0.0)	0.0 (0.0-0.0)	64.2 (47.4-98.9)	0.0 (0.0-0.0)	0.0 (0.0-0.0)	0.0 (0.0-0.0)	24.3 (18.2-35.5)	15.1 (11.3-22.1)
**5 to 9**	0.0 (0.0-0.0)	0.0 (0.0-0.0)	0.0 (0.0-0.0)	0.0 (0.0-0.0)	0.0 (0.0-0.0)	0.0 (0.0-0.0)	0.0 (0.0-0.0)	49.9 (36.0-76.2)	0.0 (0.0-0.0)	0.0 (0.0-0.0)	0.0 (0.0-0.0)	29.5 (21.4-44.3)	9.5 (6.9-13.0)
**10 to 14**	0.0 (0.0-0.0)	0.0 (0.0-0.0)	0.0 (0.0-0.0)	0.0 (0.0-0.0)	0.0 (0.0-0.0)	0.0 (0.0-0.0)	0.0 (0.0-0.0)	56.9 (43.6-80.5)	0.0 (0.0-0.0)	0.0 (0.0-0.0)	0.0 (0.0-0.0)	33.0 (25.5-46.5)	12.0 (9.6-14.9)
**15 to 19**	12.1 (8.3-14.7)	5.0 (2.2-6.3)	17.7 (14.9-20.8)	5.2 (4.0-6.3)	6.6 (5.6-7.7)	9.5 (8.3-11.1)	6.4 (5.2-7.7)	65.4 (52.8-87.4)	1.2 (0.9-1.4)	10.6 (8.3-13.2)	2.8 (2.4-3.2)	31.1 (25.2-43.8)	20.4 (17.0-23.8)
**20 to 24**	35.3 (26.1-43.7)	22.5 (10.7-28.5)	38.9 (33.7-45.6)	16.5 (13.5-19.8)	14.2 (12.3-16.5)	12.5 (11.0-14.1)	10.2 (8.5-12.0)	50.9 (40.7-64.6)	3.4 (2.8-3.9)	16.5 (13.4-19.8)	8.1 (7.2-9.1)	31.7 (26.2-43.3)	24.6 (20.9-28.5)
**25 to 29**	83.8 (66.9-98.8)	47.8 (26.8-57.6)	50.7 (43.5-59.1)	20.2 (16.8-23.7)	17.9 (15.5-20.7)	19.6 (17.4-21.9)	13.2 (11.3-15.7)	45.8 (37.1-58.5)	9.4 (7.7-10.7)	25.5 (21.8-30.0)	11.9 (10.4-13.5)	34.0 (28.2-45.8)	25.3 (20.7-28.9)
**30 to 34**	174.9 (145.5-203.6)	115.5 (74.7-136.9)	88.8 (77.3-104.0)	48.8 (40.6-57.0)	30.9 (26.8-35.6)	30.0 (26.7-33.2)	30.0 (26.2-35.6)	57.4 (45.1-72.8)	24.3 (20.1-27.6)	35.0 (29.9-40.8)	20.2 (18.0-22.5)	37.2 (31.0-50.9)	29.0 (23.6-32.6)
**35 to 39**	371.9 (314.1-436.5)	228.6 (175.7-270.1)	162.7 (140.7-188.6)	66.7 (57.0-76.9)	52.8 (45.9-60.9)	62.5 (54.7-70.0)	52.6 (46.2-62.9)	66.3 (49.0-81.4)	60.4 (48.9-70.3)	60.2 (52.3-69.5)	39.8 (35.9-44.0)	55.0 (45.2-76.4)	36.3 (28.3-40.6)
**40 to 44**	551.2 (472.7-648.3)	369.7 (316.0-444.3)	243.5 (215.3-275.3)	116.9 (100.3-132.6)	109.9 (96.8-125.0)	115.0 (100.5-129.7)	93.8 (83.4-117.0)	88.6 (69.8-107.6)	124.5 (99.2-143.5)	98.2 (85.3-111.9)	81.2 (72.0-90.3)	67.7 (55.7-93.4)	48.7 (35.6-54.6)
**45 to 49**	717.6 (610.1-845.2)	512.0 (446.4-651.4)	298.4 (265.0-336.9)	219.7 (190.7-245.6)	165.6 (147.3-185.1)	186.8 (162.5-208.5)	133.1 (120.2-167.4)	100.9 (77.3-118.9)	191.2 (161.5-217.3)	150.5 (132.3-168.8)	136.8 (124.2-150.1)	84.7 (68.9-115.0)	62.8 (45.7-70.1)
**50 to 54**	980.8 (852.9-1135.2)	629.4 (546.0-832.3)	404.0 (357.4-458.7)	308.2 (270.1-346.2)	217.0 (190.1-248.8)	284.5 (243.9-323.9)	213.2 (191.6-266.3)	126.6 (91.1-149.9)	252.3 (209.6-291.2)	219.3 (196.9-243.6)	203.0 (184.1-222.8)	98.9 (78.9-141.4)	93.9 (66.6-105.5)
**55 to 59**	1004.7 (854.9-1172.3)	655.3 (566.3-932.8)	474.0 (415.5-536.7)	353.7 (305.1-397.2)	268.9 (237.9-303.2)	287.9 (248.3-319.4)	255.4 (227.1-325.0)	135.8 (100.6-160.8)	261.5 (211.9-296.4)	244.0 (214.8-273.1)	215.5 (194.6-236.1)	108.1 (87.6-147.9)	112.6 (78.9-125.4)
**60 to 64**	886.4 (746.5-1033.0)	618.2 (532.3-872.6)	546.9 (476.3-618.1)	396.6 (340.8-451.0)	351.9 (307.3-398.9)	308.3 (267.3-345.0)	325.0 (285.5-425.3)	141.3 (106.3-167.7)	307.8 (252.7-349.4)	313.3 (273.7-350.8)	218.2 (195.8-239.1)	104.2 (84.5-143.4)	154.2 (119.2-171.9)
**65 to 69**	842.8 (700.2-990.8)	530.5 (452.4-732.1)	630.1 (541.3-717.5)	444.0 (378.1-506.2)	336.6 (292.6-382.3)	294.2 (260.4-328.7)	382.2 (335.1-480.3)	151.2 (112.2-180.9)	288.5 (240.7-328.8)	279.5 (239.4-316.5)	241.1 (215.4-266.8)	112.3 (90.4-147.5)	167.3 (133.9-186.3)
**70 to 74**	757.3 (617.3-875.3)	464.0 (385.0-660.5)	589.0 (502.2-671.4)	511.6 (430.7-581.9)	329.4 (277.6-375.5)	278.1 (238.5-312.2)	408.7 (350.3-507.6)	144.8 (103.2-173.0)	255.5 (208.0-295.6)	297.5 (247.4-341.0)	283.7 (245.0-316.6)	92.9 (71.3-120.6)	154.7 (126.2-173.3)
**75 to 79**	698.1 (561.0-810.4)	321.8 (266.8-438.9)	614.1 (514.3-707.2)	622.4 (517.9-710.4)	315.6 (266.9-363.5)	213.7 (183.9-239.9)	354.4 (303.1-422.6)	125.9 (90.5-148.4)	203.4 (164.0-234.5)	228.8 (183.4-267.4)	305.3 (261.6-342.6)	88.8 (64.7-112.9)	131.5 (102.8-147.7)
**80+**	627.5 (495.9-743.0)	241.9 (197.3-318.9)	628.7 (505.9-729.3)	519.5 (427.0-597.5)	348.8 (275.8-414.2)	144.8 (119.0-166.8)	282.4 (231.8-332.9)	93.2 (67.3-112.4)	173.5 (136.5-202.3)	231.4 (183.1-268.1)	250.6 (208.9-281.3)	55.3 (38.3-70.0)	106.9 (85.2-120.4)

DALY is disability-adjusted life-year, which is presented as rate per 100,000.

Females

Age group (in years)	Liver cancer (95% uncertainty interval)	Uterine cancer (95% uncertainty interval)	Pancreatic cancer (95% uncertainty interval)	Thyroid cancer (95% uncertainty interval)	Larynx cancer (95% uncertainty interval)	Multiple myeloma (95% uncertainty interval)	Nasopharynx cancer (95% uncertainty interval)	Hodgkin's lymphoma (95% uncertainty interval)	Bladder cancer (95% uncertainty interval)	Kidney cancer (95% uncertainty interval)	Malignant skin melanoma (95% uncertainty interval)	Mesothelioma (95% uncertainty interval)	Non-melanoma skin cancer (95% uncertainty interval)
**<1**	0.0 (0.0-0.0)	0.0 (0.0 to 0.0)	0.0 (0.0 to 0.0)	0.0 (0.0 to 0.0)	0.0 (0.0 to 0.0)	0.0 (0.0 to 0.0)	0.0 (0.0 to 0.0)	1.1 (0.9 to 1.4)	0.0 (0.0 to 0.0)	5.4 (4.5 to 6.3)	0.0 (0.0 to 0.0)	0.0 (0.0 to 0.0)	0.0 (0.0 to 0.0)
**1 to 4**	0.0 (0.0-0.0)	0.0 (0.0 to 0.0)	0.0 (0.0 to 0.0)	0.0 (0.0 to 0.0)	0.0 (0.0 to 0.0)	0.0 (0.0 to 0.0)	0.0 (0.0 to 0.0)	1.9 (1.1 to 3.4)	0.0 (0.0 to 0.0)	4.8 (3.8 to 5.8)	0.0 (0.0 to 0.0)	0.0 (0.0 to 0.0)	0.0 (0.0 to 0.0)
**5 to 9**	1.4 (1.0-1.9)	0.0 (0.0 to 0.0)	0.0 (0.0 to 0.0)	0.0 (0.0 to 0.0)	0.0 (0.0 to 0.0)	0.0 (0.0 to 0.0)	0.6 (0.5 to 0.8)	4.8 (2.9 to 8.1)	0.0 (0.0 to 0.0)	1.8 (1.4 to 2.3)	0.0 (0.0 to 0.0)	0.0 (0.0 to 0.0)	0.0 (0.0 to 0.0)
**10 to 14**	0.9 (0.7-1.2)	0.0 (0.0 to 0.0)	0.0 (0.0 to 0.0)	1.4 (1.0 to 1.8)	0.0 (0.0 to 0.0)	0.0 (0.0 to 0.0)	2.0 (1.6 to 2.5)	7.2 (4.9 to 11.7)	0.0 (0.0 to 0.0)	1.0 (0.8 to 1.3)	0.0 (0.0 to 0.0)	0.0 (0.0 to 0.0)	0.0 (0.0 to 0.0)
**15 to 19**	2.6 (2.1-3.2)	0.8 (0.7 to 0.9)	1.8 (1.5 to 2.0)	6.8 (5.5 to 8.4)	1.3 (1.1 to 1.6)	2.9 (2.3 to 4.8)	2.6 (2.2 to 3.1)	11.5 (8.4 to 18.1)	0.8 (0.7 to 0.9)	1.1 (0.9 to 1.2)	1.1 (0.9 to 1.6)	1.2 (0.7 to 1.8)	0.7 (0.6 to 0.8)
**20 to 24**	5.2 (4.4-6.2)	1.5 (1.3 to 1.8)	2.6 (2.2 to 3.0)	11.7 (10.0 to 13.8)	2.5 (2.0 to 3.1)	3.6 (3.0 to 5.3)	2.5 (2.1 to 3.0)	12.0 (8.7 to 19.0)	1.1 (0.9 to 1.2)	1.4 (1.2 to 1.6)	1.6 (1.3 to 2.3)	1.5 (0.7 to 2.2)	0.7 (0.6 to 0.8)
**25 to 29**	7.2 (5.9-8.5)	2.2 (1.9 to 2.6)	4.7 (4.2 to 5.3)	14.9 (12.9 to 17.1)	3.0 (2.3 to 4.0)	3.7 (3.1 to 5.2)	3.1 (2.6 to 3.6)	10.5 (7.6 to 16.4)	1.3 (1.1 to 1.4)	1.9 (1.6 to 2.1)	2.3 (2.0 to 3.2)	2.1 (1.1 to 3.0)	0.7 (0.6 to 0.7)
**30 to 34**	14.0 (11.5-16.6)	5.1 (4.4 to 5.8)	8.4 (7.4 to 9.5)	16.5 (14.3 to 19.1)	7.7 (6.1 to 10.1)	4.2 (3.5 to 5.4)	4.7 (3.8 to 5.6)	9.4 (6.9 to 14.0)	2.1 (1.9 to 2.4)	2.7 (2.4 to 3.1)	2.4 (2.0 to 3.3)	2.4 (1.6 to 3.4)	0.6 (0.6 to 0.7)
**35 to 39**	24.0 (20.0-28.1)	13.2 (11.7 to 15.1)	16.5 (14.9 to 18.3)	17.1 (14.6 to 20.0)	12.5 (10.9 to 14.5)	8.9 (7.1 to 10.7)	8.1 (6.7 to 9.7)	10.6 (7.5 to 16.3)	4.6 (4.0 to 5.2)	4.5 (4.0 to 5.1)	4.3 (3.6 to 5.7)	3.7 (2.6 to 5.3)	0.8 (0.7 to 0.9)
**40 to 44**	46.2 (37.5-54.1)	28.2 (24.7 to 32.2)	32.8 (29.9 to 36.0)	28.1 (24.0 to 32.8)	24.6 (21.6 to 28.3)	16.6 (12.7 to 19.7)	17.1 (14.2 to 20.3)	12.3 (8.7 to 18.4)	9.5 (8.5 to 10.9)	7.2 (6.5 to 8.2)	5.0 (4.3 to 6.5)	5.2 (3.9 to 7.1)	1.4 (1.2 to 1.6)
**45 to 49**	72.2 (59.3-83.7)	51.4 (45.4 to 57.7)	54.0 (49.0 to 59.5)	37.6 (32.5 to 43.3)	40.1 (35.8 to 45.1)	31.7 (23.6 to 37.2)	26.7 (22.5 to 31.1)	15.6 (11.0 to 22.6)	13.5 (12.1 to 15.3)	12.4 (11.1 to 13.9)	6.9 (5.3 to 8.1)	5.0 (3.8 to 6.8)	2.7 (2.3 to 3.0)
**50 to 54**	114.7 (90.4-135.7)	92.7 (80.8 to 103.9)	93.5 (82.4 to 104.9)	61.3 (52.2 to 72.0)	57.8 (51.0 to 65.1)	52.7 (37.7 to 61.8)	41.0 (33.7 to 48.2)	19.0 (12.6 to 28.6)	25.0 (22.2 to 28.0)	18.1 (15.9 to 20.7)	8.9 (6.0 to 10.4)	7.2 (5.4 to 10.0)	3.8 (3.3 to 4.3)
**55 to 59**	135.8 (109.0-160.4)	119.2 (105.3 to 134.4)	119.8 (108.4 to 130.7)	70.3 (61.1 to 81.4)	61.7 (54.5 to 69.5)	60.6 (43.8 to 69.6)	45.5 (38.4 to 52.7)	20.2 (14.6 to 30.6)	29.5 (26.6 to 32.7)	25.5 (23.1 to 28.4)	11.2 (8.0 to 13.1)	7.6 (5.6 to 10.3)	4.4 (3.9 to 4.9)
**60 to 64**	153.6 (127.6-182.2)	157.5 (137.7 to 179.5)	182.5 (163.1 to 200.3)	84.9 (74.2 to 98.2)	75.6 (66.8 to 85.6)	79.4 (59.4 to 95.1)	41.6 (34.6 to 49.1)	22.2 (15.2 to 34.3)	47.4 (42.3 to 52.7)	31.4 (27.9 to 35.4)	13.4 (9.6 to 15.6)	8.3 (5.9 to 11.4)	5.4 (4.8 to 6.2)
**65 to 69**	178.6 (146.3-210.4)	170.3 (147.5 to 194.4)	225.5 (200.2 to 249.1)	94.3 (80.9 to 109.3)	77.1 (65.6 to 88.8)	89.6 (70.3 to 104.5)	33.0 (27.1 to 38.8)	19.4 (13.6 to 30.5)	61.9 (54.6 to 68.9)	32.4 (28.9 to 35.9)	14.6 (10.8 to 17.3)	8.5 (5.8 to 11.8)	7.5 (6.5 to 8.5)
**70 to 74**	202.0 (163.0-235.1)	144.5 (124.0 to 164.7)	255.5 (222.7 to 284.7)	84.8 (73.0 to 98.3)	84.0 (70.3 to 96.2)	73.7 (57.4 to 91.6)	29.0 (23.8 to 34.4)	17.5 (12.1 to 26.2)	72.1 (62.3 to 81.1)	32.1 (28.1 to 36.1)	15.8 (11.5 to 18.6)	7.4 (5.2 to 10.4)	7.0 (6.0 to 7.9)
**75 to 79**	198.2 (156.3-232.0)	122.5 (103.0 to 140.1)	261.4 (225.9 to 291.7)	75.7 (63.9 to 87.2)	65.0 (55.1 to 73.2)	73.4 (58.2 to 93.3)	19.8 (16.4 to 23.6)	15.4 (10.7 to 22.8)	89.1 (77.0 to 100.2)	27.6 (24.2 to 30.9)	16.0 (11.6 to 18.6)	6.3 (4.7 to 8.8)	8.0 (6.8 to 9.1)
**80+**	168.8 (127.8-198.0)	97.8 (82.5 to 111.8)	222.5 (184.6 to 251.8)	58.5 (48.5 to 68.4)	59.7 (48.6 to 67.8)	67.6 (54.5 to 90.5)	13.9 (11.1 to 16.6)	9.7 (6.3 to 14.3)	99.3 (83.3 to 112.6)	23.3 (19.7 to 26.6)	19.2 (12.9 to 22.4)	4.1 (3.3 to 5.5)	17.1 (14.2 to 19.5)

DALY is disability-adjusted life-year, which is presented as rate per 100,000.

Males

Age group (in years)	Tracheal, bronchus, and lung cancer (95% uncertainty interval)	Lip and oral cavity cancer (95% uncertainty interval)	Pharynx cancer other than nasopharynx (95% uncertainty interval)	Stomach cancer (95% uncertainty interval)	Leukaemia (95% uncertainty interval)	Colon and rectum cancer (95% uncertainty interval)	Oesophageal cancer (95% uncertainty interval)	Larynx cancer (95% uncertainty interval)	Liver cancer (95% uncertainty interval)	Brain and nervous system cancer (95% uncertainty interval)	Non-Hodgkin lymphoma (95% uncertainty interval)	Prostate cancer (95% uncertainty interval)
						Males						
**<1**	0.0 (0.0 to 0.0)	0.0 (0.0 to 0.0)	0.0 (0.0 to 0.0)	0.0 (0.0 to 0.0)	80.2 (65.7 to 98.0)	0.0 (0.0 to 0.0)	0.0 (0.0 to 0.0)	0.0 (0.0 to 0.0)	0.0 (0.0 to 0.0)	26.4 (21.3 to 35.5)	33.1 (25.6 to 46.2)	0.0 (0.0 to 0.0)
**1 to 4**	0.0 (0.0 to 0.0)	0.0 (0.0 to 0.0)	0.0 (0.0 to 0.0)	0.0 (0.0 to 0.0)	88.7 (62.1 to 115.2)	0.0 (0.0 to 0.0)	0.0 (0.0 to 0.0)	0.0 (0.0 to 0.0)	0.0 (0.0 to 0.0)	32.7 (23.0 to 48.7)	22.2 (14.5 to 32.2)	0.0 (0.0 to 0.0)
**5 to 9**	0.0 (0.0 to 0.0)	0.0 (0.0 to 0.0)	0.0 (0.0 to 0.0)	0.0 (0.0 to 0.0)	88.0 (65.1 to 112.6)	0.0 (0.0 to 0.0)	0.0 (0.0 to 0.0)	0.0 (0.0 to 0.0)	3.3 (2.6 to 4.2)	40.8 (30.1 to 57.4)	18.8 (13.4 to 25.1)	0.0 (0.0 to 0.0)
**10 to 14**	0.0 (0.0 to 0.0)	0.0 (0.0 to 0.0)	0.0 (0.0 to 0.0)	0.0 (0.0 to 0.0)	90.8 (72.2 to 111.7)	0.0 (0.0 to 0.0)	0.0 (0.0 to 0.0)	0.0 (0.0 to 0.0)	2.8 (2.3 to 3.4)	42.8 (33.1 to 58.0)	21.1 (16.4 to 26.1)	0.0 (0.0 to 0.0)
**15 to 19**	10.9 (9.0 to 13.3)	11.7 (9.7 to 14.1)	8.5 (6.4 to 10.5)	15.1 (13.1 to 17.2)	110.0 (93.6 to 137.7)	11.9 (9.9 to 14.0)	4.5 (3.8 to 5.5)	2.0 (1.7 to 2.4)	2.9 (2.5 to 3.4)	36.4 (29.8 to 48.0)	38.9 (30.7 to 46.0)	0.9 (0.7 to 1.2)
**20 to 24**	15.3 (12.9 to 17.9)	22.7 (19.5 to 26.4)	18.2 (14.6 to 21.8)	29.0 (25.5 to 33.6)	84.1 (72.9 to 106.1)	15.5 (13.2 to 17.8)	10.0 (8.6 to 11.6)	3.2 (2.8 to 3.6)	6.8 (6.0 to 7.9)	39.2 (32.7 to 51.6)	45.4 (36.7 to 53.1)	1.1 (0.9 to 1.8)
**25 to 29**	30.0 (25.7 to 34.5)	45.6 (38.7 to 53.1)	24.8 (19.5 to 29.8)	47.0 (41.3 to 54.1)	73.9 (64.5 to 91.3)	28.0 (24.0 to 31.7)	14.6 (12.9 to 16.8)	4.8 (4.3 to 5.2)	11.1 (9.8 to 12.7)	47.4 (39.7 to 61.9)	40.7 (31.1 to 47.0)	1.7 (1.3 to 2.8)
**30 to 34**	48.6 (41.6 to 56.8)	85.2 (73.2 to 99.8)	53.4 (43.8 to 62.2)	75.9 (67.2 to 86.1)	79.2 (68.9 to 95.1)	46.9 (40.7 to 53.3)	24.9 (22.1 to 28.0)	12.9 (11.5 to 14.5)	23.9 (21.4 to 27.0)	64.2 (54.2 to 83.7)	50.2 (38.1 to 57.6)	2.3 (1.8 to 3.7)
**35 to 39**	87.1 (75.7 to 98.6)	158.1 (137.8 to 179.4)	108.5 (89.9 to 126.4)	120.4 (107.5 to 135.8)	89.2 (77.5 to 112.8)	72.2 (62.1 to 81.1)	54.1 (48.7 to 59.9)	33.6 (29.8 to 38.1)	47.6 (42.2 to 53.6)	85.8 (71.6 to 114.0)	62.4 (44.8 to 71.5)	3.5 (2.9 to 5.5)
**40 to 44**	153.4 (135.8 to 169.9)	263.7 (231.6 to 297.9)	213.9 (178.0 to 244.8)	214.2 (194.2 to 238.4)	105.2 (92.0 to 129.3)	95.0 (83.3 to 105.4)	98.6 (89.6 to 108.7)	86.9 (77.7 to 97.0)	74.9 (66.8 to 84.2)	102.0 (86.4 to 135.6)	75.2 (53.3 to 86.0)	7.2 (6.1 to 10.8)
**45 to 49**	299.8 (269.0 to 330.4)	369.2 (329.1 to 412.3)	344.4 (294.2 to 392.8)	326.8 (296.2 to 363.0)	123.2 (109.7 to 154.8)	181.0 (159.6 to 199.8)	181.2 (165.7 to 196.9)	161.3 (145.6 to 176.8)	127.7 (114.8 to 143.0)	112.3 (94.0 to 147.9)	94.6 (65.2 to 108.4)	18.2 (15.4 to 25.2)
**50 to 54**	501.1 (449.5 to 551.6)	536.6 (474.0 to 594.5)	501.6 (426.6 to 570.4)	429.7 (391.2 to 469.7)	143.0 (126.3 to 179.4)	229.9 (202.6 to 253.7)	273.8 (252.8 to 296.5)	277.6 (252.3 to 305.6)	217.6 (195.4 to 243.5)	135.1 (116.1 to 174.5)	126.1 (86.3 to 143.4)	49.0 (38.9 to 64.3)
**55 to 59**	767.0 (697.4 to 835.6)	656.2 (587.7 to 721.6)	640.1 (549.7 to 722.5)	587.0 (535.2 to 646.0)	177.3 (154.5 to 226.0)	333.6 (297.3 to 368.5)	374.7 (346.1 to 405.3)	388.7 (351.3 to 428.0)	322.2 (291.0 to 358.2)	166.2 (139.4 to 222.4)	163.0 (109.3 to 186.8)	107.9 (81.6 to 139.4)
**60 to 64**	932.3 (828.7 to 1024.1)	696.5 (615.5 to 772.6)	775.7 (651.6 to 883.3)	683.2 (617.8 to 756.0)	195.6 (169.6 to 236.4)	443.3 (392.6 to 487.3)	413.1 (375.3 to 449.2)	455.4 (410.3 to 500.8)	417.6 (373.8 to 461.5)	158.8 (135.4 to 201.6)	198.7 (146.0 to 225.7)	241.6 (177.5 to 302.4)
**65 to 69**	1162.2 (1029.4 to 1277.1)	720.3 (633.5 to 792.6)	779.2 (652.4 to 891.2)	771.9 (700.4 to 853.6)	218.8 (192.7 to 266.0)	565.4 (495.9 to 621.1)	476.5 (433.3 to 518.4)	529.9 (473.8 to 587.6)	487.6 (438.2 to 540.6)	162.9 (138.2 to 209.9)	225.7 (162.1 to 256.0)	419.2 (314.8 to 517.3)
**70 to 74**	1186.2 (1042.3 to 1296.9)	674.7 (590.7 to 751.9)	760.4 (636.1 to 868.8)	752.2 (678.4 to 831.1)	217.2 (185.6 to 273.7)	584.3 (513.2 to 648.6)	430.3 (387.1 to 471.3)	444.9 (401.7 to 491.4)	483.0 (431.3 to 535.7)	127.2 (104.8 to 158.5)	212.1 (152.3 to 240.4)	659.3 (499.1 to 834.4)
**75 to 79**	1147.6 (1013.2 to 1257.5)	572.0 (495.8 to 633.1)	646.4 (541.9 to 743.7)	729.0 (650.5 to 803.1)	201.8 (171.8 to 241.2)	626.7 (551.7 to 694.8)	446.8 (403.9 to 486.6)	352.2 (317.5 to 386.0)	460.7 (410.9 to 506.3)	105.4 (82.0 to 127.9)	202.9 (155.3 to 230.4)	977.3 (718.2 to 1203.2)
**80+**	777.1 (682.7 to 855.3)	524.0 (453.9 to 593.5)	621.8 (515.8 to 731.0)	743.4 (640.6 to 821.2)	161.6 (136.6 to 203.3)	532.3 (474.7 to 603.0)	381.7 (345.1 to 423.3)	299.4 (266.0 to 329.7)	337.8 (294.8 to 374.2)	72.8 (56.5 to 90.9)	185.2 (141.1 to 211.8)	961.5 (713.4 to 1192.5)

DALY is disability-adjusted life-year, which is presented as rate per 100,000.

Males

Age group (in years)	Pancreatic cancer (95% uncertainty interval)	Gallbladder and biliary tract cancer (95% uncertainty interval)	Bladder cancer (95% uncertainty interval)	Nasopharynx cancer (95% uncertainty interval)	Hodgkin's lymphoma (95% uncertainty interval)	Multiple myeloma (95% uncertainty interval)	Kidney cancer (95% uncertainty interval)	Thyroid cancer (95% uncertainty interval)	Testicular cancer (95% uncertainty interval)	Mesothelioma (95% uncertainty interval)	Breast cancer (95% uncertainty interval)	Non-melanoma skin cancer (95% uncertainty interval)	Malignant skin melanoma (95% uncertainty interval)
**<1**	0.0 (0.0 to 0.0)	0.0 (0.0 to 0.0)	0.0 (0.0 to 0.0)	0.0 (0.0 to 0.0)	2.6 (2.0 to 4.3)	0.0 (0.0 to 0.0)	6.4 (5.5 to 7.6)	0.0 (0.0 to 0.0)	0.0 (0.0 to 0.0)	0.0 (0.0 to 0.0)	0.0 (0.0 to 0.0)	0.0 (0.0 to 0.0)	0.0 (0.0 to 0.0)
**1 to 4**	0.0 (0.0 to 0.0)	0.0 (0.0 to 0.0)	0.0 (0.0 to 0.0)	0.0 (0.0 to 0.0)	4.2 (2.9 to 7.9)	0.0 (0.0 to 0.0)	5.9 (4.4 to 7.7)	0.0 (0.0 to 0.0)	0.0 (0.0 to 0.0)	0.0 (0.0 to 0.0)	0.0 (0.0 to 0.0)	0.0 (0.0 to 0.0)	0.0 (0.0 to 0.0)
**5 to 9**	0.0 (0.0 to 0.0)	0.0 (0.0 to 0.0)	0.0 (0.0 to 0.0)	1.3 (1.0 to 1.7)	21.1 (14.0 to 33.4)	0.0 (0.0 to 0.0)	2.3 (1.8 to 3.0)	0.0 (0.0 to 0.0)	0.0 (0.0 to 0.0)	0.0 (0.0 to 0.0)	0.0 (0.0 to 0.0)	0.0 (0.0 to 0.0)	0.0 (0.0 to 0.0)
**10 to 14**	0.0 (0.0 to 0.0)	0.0 (0.0 to 0.0)	0.0 (0.0 to 0.0)	4.5 (3.5 to 5.9)	19.2 (14.2 to 32.7)	0.0 (0.0 to 0.0)	0.9 (0.7 to 1.1)	1.0 (0.8 to 1.3)	0.0 (0.0 to 0.0)	0.0 (0.0 to 0.0)	0.0 (0.0 to 0.0)	0.0 (0.0 to 0.0)	0.0 (0.0 to 0.0)
**15 to 19**	1.9 (1.7 to 2.1)	0.9 (0.7 to 1.1)	1.1 (0.9 to 1.3)	5.8 (4.9 to 6.8)	22.0 (16.8 to 34.6)	2.0 (1.6 to 3.1)	1.0 (0.9 to 1.1)	3.3 (2.7 to 4.4)	5.4 (4.5 to 6.6)	0.8 (0.7 to 1.0)	0.7 (0.6 to 0.8)	0.7 (0.7 to 0.8)	1.6 (1.3 to 2.7)
**20 to 24**	4.2 (3.8 to 4.7)	1.9 (1.3 to 2.3)	1.8 (1.6 to 2.0)	5.1 (4.3 to 6.0)	17.7 (14.1 to 27.7)	3.4 (2.8 to 5.2)	1.6 (1.4 to 1.8)	5.8 (5.1 to 7.7)	11.2 (9.5 to 13.0)	1.1 (0.8 to 1.3)	0.7 (0.7 to 0.8)	0.8 (0.7 to 0.9)	2.7 (2.2 to 4.4)
**25 to 29**	5.7 (5.2 to 6.3)	4.9 (3.1 to 6.0)	2.2 (2.0 to 2.4)	4.7 (4.0 to 5.5)	17.6 (14.0 to 26.4)	3.0 (2.5 to 4.3)	2.7 (2.4 to 3.0)	5.9 (5.2 to 7.7)	14.3 (12.3 to 16.6)	1.9 (1.4 to 2.5)	0.8 (0.7 to 0.8)	0.9 (0.8 to 1.0)	3.4 (2.8 to 5.6)
**30 to 34**	13.5 (12.2 to 14.9)	13.0 (8.4 to 15.9)	4.5 (4.0 to 5.1)	8.1 (7.0 to 9.6)	20.5 (15.9 to 32.8)	8.5 (6.6 to 11.8)	4.6 (4.1 to 5.2)	8.0 (7.0 to 10.3)	16.2 (14.2 to 18.7)	2.5 (1.9 to 3.4)	2.2 (1.8 to 2.7)	1.7 (1.5 to 1.9)	3.5 (3.0 to 5.6)
**35 to 39**	23.9 (21.9 to 26.1)	27.1 (17.1 to 32.8)	8.3 (7.5 to 9.2)	16.4 (14.3 to 19.1)	21.6 (16.8 to 34.4)	13.3 (9.9 to 17.7)	9.3 (8.4 to 10.3)	8.9 (7.8 to 11.1)	15.8 (13.5 to 18.1)	4.9 (3.5 to 7.1)	4.6 (3.7 to 5.5)	3.0 (2.7 to 3.3)	6.7 (5.7 to 10.1)
**40 to 44**	42.8 (39.3 to 46.5)	49.5 (32.4 to 60.1)	18.1 (16.3 to 20.1)	35.5 (31.2 to 40.7)	25.1 (19.2 to 40.1)	22.7 (16.9 to 27.6)	14.1 (12.7 to 15.7)	13.9 (12.2 to 16.8)	12.1 (10.3 to 14.1)	7.7 (5.7 to 10.7)	7.6 (6.2 to 8.9)	4.5 (4.1 to 5.0)	8.9 (7.7 to 12.3)
**45 to 49**	75.8 (70.4 to 81.7)	71.7 (46.1 to 86.7)	29.8 (27.3 to 32.6)	51.5 (45.0 to 59.8)	29.5 (23.2 to 45.5)	28.0 (18.9 to 32.7)	25.6 (23.2 to 28.2)	22.1 (19.5 to 26.8)	11.2 (9.5 to 12.9)	13.9 (10.6 to 18.9)	12.4 (10.2 to 14.7)	6.5 (5.9 to 7.2)	9.2 (7.9 to 12.8)
**50 to 54**	117.4 (109.5 to 126.4)	112.9 (74.3 to 134.2)	56.0 (51.9 to 60.9)	81.3 (70.8 to 95.5)	30.8 (24.9 to 47.0)	48.2 (30.7 to 56.0)	41.9 (38.1 to 46.0)	34.8 (30.9 to 41.4)	10.6 (8.9 to 12.5)	21.2 (16.1 to 28.1)	16.5 (12.2 to 19.1)	10.3 (9.4 to 11.1)	13.1 (11.4 to 17.4)
**55 to 59**	182.5 (169.9 to 195.9)	145.8 (96.2 to 178.5)	85.3 (79.4 to 91.6)	101.5 (90.2 to 115.0)	38.9 (31.6 to 58.5)	72.2 (43.3 to 84.6)	66.4 (60.8 to 73.8)	44.2 (38.7 to 53.0)	8.5 (7.2 to 10.0)	31.0 (23.9 to 41.1)	25.8 (20.7 to 29.6)	16.5 (15.1 to 18.0)	17.4 (15.2 to 23.6)
**60 to 64**	229.9 (211.9 to 246.8)	183.7 (122.7 to 227.8)	140.9 (127.9 to 153.7)	101.2 (87.2 to 119.5)	36.4 (28.8 to 55.3)	79.0 (48.7 to 92.7)	72.4 (66.2 to 79.6)	49.6 (43.5 to 60.9)	9.2 (7.7 to 11.0)	30.2 (22.9 to 38.6)	29.6 (23.5 to 34.9)	28.1 (25.8 to 30.4)	14.1 (11.9 to 17.8)
**65 to 69**	290.1 (267.1 to 313.5)	188.0 (125.7 to 241.3)	187.1 (169.2 to 205.6)	95.1 (82.6 to 111.7)	35.0 (28.0 to 52.8)	87.4 (56.4 to 101.1)	85.3 (77.7 to 93.8)	54.6 (48.1 to 66.4)	10.0 (8.4 to 11.8)	33.4 (25.5 to 42.9)	27.4 (22.0 to 32.6)	38.5 (35.4 to 41.3)	17.5 (14.4 to 21.4)
**70 to 74**	333.6 (304.7 to 362.0)	172.4 (121.2 to 223.2)	221.3 (198.8 to 243.4)	78.3 (67.8 to 91.6)	27.0 (21.3 to 42.1)	89.6 (59.6 to 103.8)	73.4 (65.9 to 81.1)	56.0 (49.3 to 68.6)	11.6 (9.7 to 13.8)	38.6 (30.0 to 49.5)	31.1 (24.3 to 36.1)	44.9 (40.7 to 48.6)	16.3 (13.6 to 20.2)
**75 to 79**	306.6 (279.3 to 332.1)	148.9 (106.5 to 198.6)	266.3 (241.0 to 289.8)	52.5 (45.3 to 60.3)	27.0 (20.9 to 41.4)	85.3 (56.8 to 98.8)	79.2 (70.9 to 87.3)	40.3 (35.2 to 47.5)	7.3 (6.0 to 8.6)	38.4 (28.9 to 48.3)	24.8 (19.6 to 28.8)	57.1 (51.9 to 61.7)	16.1 (13.0 to 19.8)
**80+**	252.9 (223.7 to 275.2)	122.7 (86.9 to 157.3)	296.0 (263.0 to 324.5)	38.3 (32.8 to 43.3)	17.6 (13.7 to 26.8)	69.5 (45.9 to 83.4)	59.7 (52.5 to 66.9)	38.3 (32.9 to 45.3)	12.4 (10.5 to 14.2)	31.5 (22.6 to 39.3)	34.1 (28.2 to 40.7)	60.7 (53.7 to 67.3)	21.2 (15.5 to 26.0)

DALY is disability-adjusted life-year, which is presented as rate per 100,000.

8. Proportion of total DALYs for leading cancers in India that are attributable to GBD risk factors, 2016

Risk factors	All cancers	Stomach cancer	Breast cancer	Lung cancer	Lip and oral cavity cancer	Pharynx cancer other than nasopharynx	Colon and rectum cancer	Leukaemia	Cervical cancer	Oesophageal cancer	Brain and nervous system cancer	Prostate cancer	Larynx cancer	Liver cancer	Ovarian cancer	Gallbladder and biliary tract cancer	Thyroid cancer
**Air pollution**	3.3%	-	-	43.0%	-	-	-	-	-	-	-	-	-	-	-	-	-
**Alcohol use**	6.6%	-	1.9%	-	29.8%	30.1%	5.5%	-	-	15.6%	-	-	17.2%	11.7%	-	-	-
**Dietary risks**	6.0%	4.1%	0.0%	11.9%	7.0%	6.8%	43.2%	0.0%	-	21.5%	-	-	7.1%	0.0%	-	-	-
**High body-mass index**	1.9%	-	0.2%	-	-	-	3.6%	2.2%	-	11.4%	-	-	-	7.6%	1.4%	9.1%	5.1%
**High fasting plasma glucose**	1.7%	-	4.9%	6.6%	-	-	6.0%	-	-	-	-	-	-	1.8%	5.5%	-	-
**Low physical activity**	0.2%	-	1.0%	-	-	-	2.3%	-	-	-	-	-	-	-	-	-	-
**Occupational risks**	1.5%	-	0.6%	13.6%	-	-	-	1.2%	-	-	-	-	5.5%	-	0.7%	-	-
**Other environmental risks**	0.4%	-	-	5.8%	-	-	-	-	-	-	-	-	-	-	-	-	-
**Tobacco**	10.9%	3.5%	3.0%	43.2%	47.5%	-	1.4%	2.3%	1.4%	35.6%	-	1.1%	37.9%	5.9%	-	-	-
Smoking	7.9%	3.5%	0.6%	41.5%	20.9%	-	1.4%	2.3%	1.4%	17.4%	-	1.1%	37.9%	5.9%	-	-	-
Secondhand smoke	0.4%	-	2.4%	2.3%	-	-	-	-	-	-	-	-	-	-	-	-	-
Smokeless tobacco	3.4%	-	-	-	33.2%	-	-	-	-	22.6%	-	-	-	-	-	-	-
**Unsafe sex**	5.2%	-	-	-	-	-	-	-	100.0%	-	-	-	-	-	-	-	-

The percent values shown are based on the total DALYs for that cancer as denominator. The sum of the percent for individual risk factors may add up to more than the total for any given cancer due to overlap of their contribution, and also because the population attributable fractions of individual risk factors can add up to more than the total even if they are independent.
DALY is disability-adjusted life-year.

9. Risk factors contributing to cancer DALYs in India, 2016

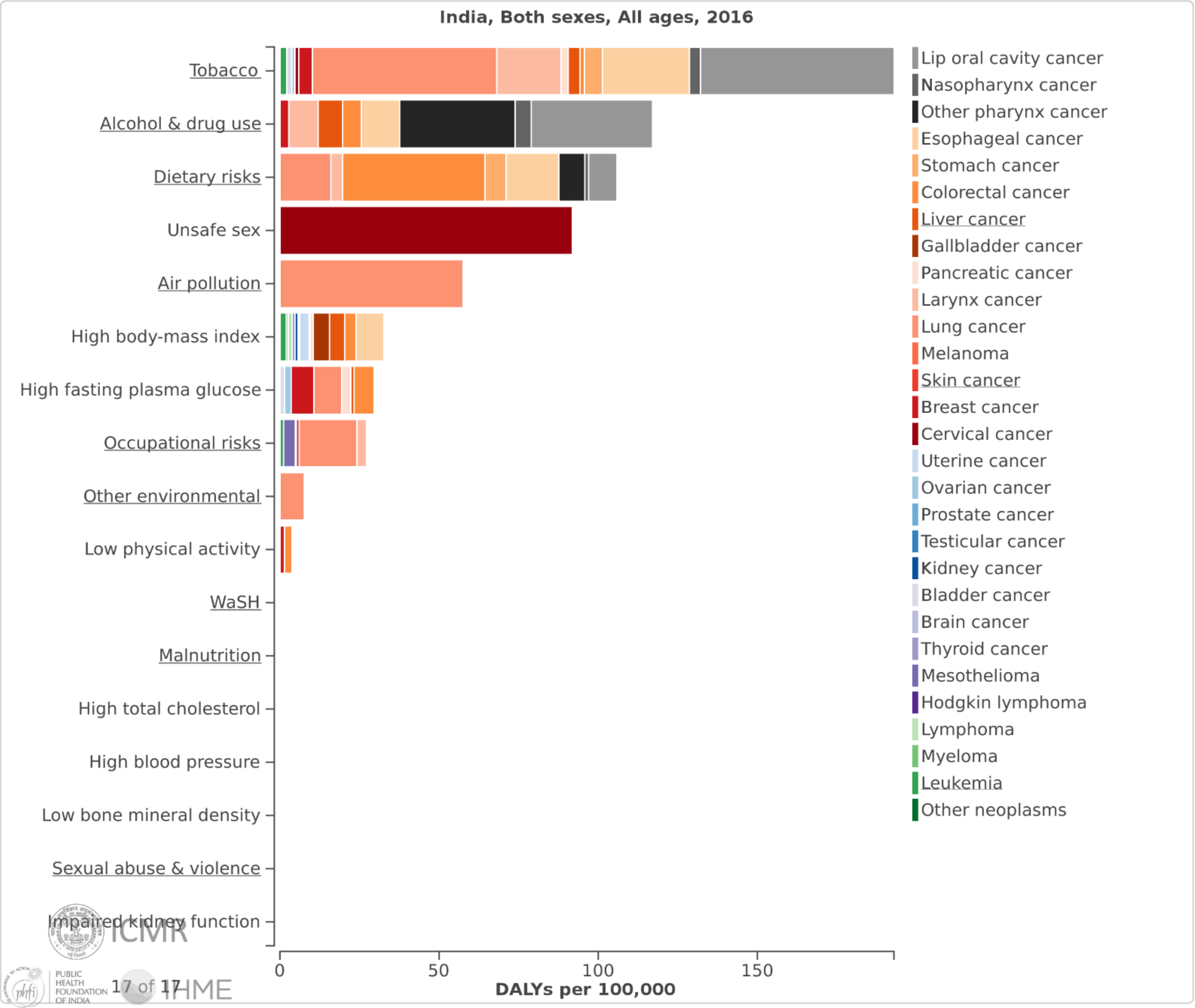

Source: Indian Council of Medical Research, Public Health Foundation of India, and Institute for Health Metrics and Evaluation. GBD India Compare. 2017. http://vizhub.healthdata.org/gbd-compare/india (accessed May 28, 2018).
DALY is disability-adjusted life-year. WaSH is water, sanitation, and hygiene.

10. Age-standardised incidence rate for different types of cancers by sex in the states of India, 2016

Both sexes combined

States of India	Both sexes combined													
	Lip and oral cavity cancer (95% uncertainty interval)	Breast cancer (95% uncertainty interval)	Stomach cancer (95% uncertainty interval)	Tracheal, bronchus, and lung cancer (95% uncertainty interval)	Colon and rectum cancer (95% uncertainty interval)	Cervical cancer (95% uncertainty interval)	Pharynx cancer other than nasopharynx (95% uncertainty interval)	Prostate cancer (95% uncertainty interval)	Oesophageal cancer (95% uncertainty interval)	Liver cancer (95% uncertainty interval)	Larynx cancer (95% uncertainty interval)	Leukaemia (95% uncertainty interval)	Gallbladder and biliary tract cancer (95% uncertainty interval)	Pancreatic cancer (95% uncertainty interval)
**India**	11.3 (10.6 to 11.8)	11.0 (10.0 to 12.1)	7.9 (7.6 to 8.1)	7.2 (6.7 to 7.6)	6.9 (6.4 to 7.2)	6.7 (6.0 to 8.5)	6.5 (5.9 to 7.1)	4.2 (3.3 to 5.1)	3.9 (3.8 to 4.0)	3.2 (3.0 to 3.4)	3.2 (3.1 to 3.3)	3.0 (2.7 to 3.4)	2.6 (2.3 to 2.9)	2.6 (2.5 to 2.6)
**Low ETL**	**12.2 (11.4 to 12.7)**	**9.5 (8.5 to 10.5)**	**9.5 (9.1 to 9.9)**	**7.0 (6.6 to 7.8)**	**7.2 (6.6 to 7.6)**	**6.8 (5.4 to 9.6)**	**7.8 (7.0 to 8.6)**	**3.9 (3.0 to 4.6)**	**4.1 (4.0 to 4.3)**	**3.5 (3.1 to 3.8)**	**3.5 (3.3 to 3.7)**	**2.9 (2.6 to 3.5)**	**3.2 (2.6 to 3.5)**	**2.5 (2.4 to 2.6)**
Bihar	5.0 (4.6 to 5.4)	7.7 (6.8 to 8.5)	4.3 (4.1 to 4.5)	7.2 (6.6 to 7.7)	6.1 (5.7 to 6.5)	6.6 (5.1 to 9.5)	2.0 (1.6 to 2.2)	3.7 (2.8 to 4.5)	5.3 (5.0 to 5.7)	3.5 (2.8 to 4.0)	2.8 (2.5 to 3.4)	2.3 (1.9 to 3.0)	2.8 (2.3 to 3.2)	2.2 (2.1 to 2.4)
Jharkhand	10.1 (9.1 to 11.0)	8.5 (7.2 to 11.4)	9.2 (8.7 to 9.8)	5.4 (4.8 to 6.3)	6.4 (5.8 to 7.0)	7.5 (5.6 to 9.8)	3.7 (3.1 to 4.2)	4.1 (3.3 to 5.1)	1.0 (0.9 to 1.1)	3.5 (3.2 to 4.0)	3.2 (2.9 to 3.6)	3.1 (2.6 to 3.5)	2.9 (2.4 to 3.3)	2.5 (2.3 to 2.7)
Uttar Pradesh	14.0 (12.8 to 15.2)	10.4 (8.7 to 12.2)	13.4 (12.6 to 14.2)	7.6 (7.0 to 8.8)	8.1 (7.4 to 9.0)	7.0 (5.3 to 10.3)	8.8 (7.5 to 10.2)	4.0 (3.1 to 4.8)	2.5 (2.3 to 2.6)	3.6 (3.2 to 4.0)	3.8 (3.4 to 4.2)	3.3 (2.9 to 3.8)	3.0 (2.6 to 3.6)	2.5 (2.3 to 2.7)
Rajasthan	9.1 (8.3 to 9.9)	9.2 (8.2 to 10.4)	10.1 (9.5 to 10.7)	6.4 (5.9 to 7.7)	6.3 (5.8 to 6.9)	6.5 (5.3 to 8.4)	12.1 (10.2 to 14.1)	3.4 (2.7 to 4.2)	4.1 (3.8 to 4.4)	3.4 (3.0 to 3.8)	3.3 (2.9 to 3.6)	2.1 (1.7 to 3.3)	2.8 (2.4 to 3.1)	2.5 (2.3 to 2.7)
Meghalaya	16.5 (15.4 to 17.7)	7.4 (6.0 to 11.6)	10.1 (9.5 to 10.7)	9.5 (8.8 to 10.1)	6.2 (5.7 to 6.7)	4.5 (3.9 to 7.6)	15.6 (13.9 to 17.3)	3.8 (3.2 to 4.8)	34.0 (31.9 to 36.2)	3.3 (3.0 to 3.6)	5.5 (5.1 to 5.9)	2.2 (1.9 to 3.3)	3.2 (2.4 to 3.7)	2.7 (2.5 to 2.8)
Assam	13.3 (12.2 to 14.6)	9.7 (8.1 to 13.2)	6.5 (6.1 to 6.9)	8.4 (7.8 to 9.1)	7.7 (7.0 to 8.4)	4.8 (4.0 to 10.0)	12.6 (11.0 to 13.9)	5.0 (3.5 to 5.7)	14.6 (13.6 to 15.7)	3.3 (2.9 to 3.6)	4.4 (4.1 to 4.8)	2.9 (2.6 to 4.1)	6.5 (2.8 to 7.8)	3.2 (3.0 to 3.5)
Chhattisgarh	11.2 (10.4 to 12.0)	9.5 (7.9 to 13.0)	7.6 (7.1 to 8.0)	6.0 (5.5 to 7.1)	7.0 (6.3 to 7.6)	8.9 (7.1 to 10.5)	8.4 (7.3 to 9.5)	3.9 (3.0 to 4.6)	3.4 (3.2 to 3.6)	3.5 (3.1 to 3.9)	3.8 (3.3 to 4.1)	3.2 (2.8 to 3.7)	3.0 (2.6 to 3.4)	3.3 (3.1 to 3.5)
Madhya Pradesh	19.7 (18.4 to 21.0)	10.5 (9.1 to 11.7)	7.0 (6.6 to 7.5)	7.3 (6.7 to 8.0)	6.6 (6.0 to 7.1)	7.8 (6.2 to 9.2)	9.1 (7.4 to 10.6)	3.9 (3.0 to 4.6)	4.5 (4.2 to 4.8)	3.0 (2.6 to 3.4)	3.6 (3.3 to 3.9)	3.2 (2.8 to 3.5)	3.2 (2.6 to 3.6)	2.4 (2.3 to 2.5)
Odisha	12.1 (11.2 to 12.9)	8.7 (7.1 to 12.2)	9.4 (8.9 to 9.9)	5.7 (5.1 to 6.4)	7.9 (7.0 to 8.6)	4.6 (3.7 to 9.9)	6.1 (5.2 to 6.9)	4.1 (3.2 to 4.9)	2.6 (2.4 to 2.7)	3.6 (3.2 to 4.0)	3.4 (3.1 to 3.8)	3.1 (2.7 to 3.5)	2.9 (2.5 to 3.4)	2.6 (2.4 to 2.8)
**Lower-middle ETL**	**14.2 (12.9 to 15.0)**	**10.8 (9.6 to 12.9)**	**5.0 (4.8 to 5.3)**	**7.9 (7.4 to 8.3)**	**6.5 (5.9 to 6.9)**	**5.6 (5.0 to 8.0)**	**5.8 (5.0 to 6.5)**	**4.0 (3.3 to 5.1)**	**4.9 (4.7 to 5.2)**	**2.5 (2.4 to 2.9)**	**2.8 (2.6 to 3.0)**	**3.2 (2.8 to 3.5)**	**1.8 (1.6 to 2.4)**	**2.8 (2.6 to 2.9)**
Arunachal Pradesh	7.4 (6.9 to 8.0)	7.5 (6.3 to 10.1)	27.3 (25.9 to 28.7)	9.2 (8.1 to 9.9)	6.0 (5.5 to 6.4)	6.7 (5.2 to 7.6)	6.4 (5.3 to 7.6)	3.8 (3.2 to 5.1)	8.7 (8.1 to 9.2)	15.7 (12.4 to 17.5)	3.6 (3.3 to 3.9)	3.1 (2.6 to 3.4)	3.3 (2.3 to 3.7)	2.8 (2.6 to 2.9)
Mizoram	6.4 (6.0 to 6.9)	10.7 (9.3 to 12.1)	32.1 (30.5 to 33.9)	31.8 (24.7 to 34.0)	9.8 (9.0 to 10.4)	7.2 (5.1 to 8.2)	7.9 (6.9 to 8.8)	4.0 (3.5 to 5.5)	15.7 (14.7 to 16.8)	7.3 (5.4 to 8.1)	4.2 (3.9 to 4.5)	3.4 (2.7 to 3.8)	3.1 (2.2 to 3.4)	2.8 (2.7 to 2.9)
Nagaland	6.8 (6.3 to 7.3)	7.4 (6.2 to 12.0)	18.9 (17.9 to 19.8)	6.6 (6.2 to 7.1)	7.6 (7.0 to 8.2)	5.1 (4.3 to 6.0)	8.3 (7.3 to 9.3)	4.7 (4.1 to 6.9)	10.5 (9.7 to 11.3)	4.9 (4.3 to 5.3)	3.7 (3.4 to 4.0)	2.3 (2.0 to 3.3)	2.1 (1.8 to 2.4)	2.3 (2.1 to 2.4)
Uttarakhand	10.5 (9.6 to 11.2)	10.1 (7.9 to 14.2)	11.6 (10.8 to 12.6)	9.4 (8.5 to 10.2)	7.4 (6.3 to 8.1)	6.6 (5.7 to 8.5)	9.5 (7.7 to 11.1)	4.6 (3.6 to 5.6)	3.0 (2.8 to 3.2)	3.4 (3.0 to 3.9)	3.6 (3.1 to 3.9)	3.0 (2.7 to 3.7)	2.7 (2.4 to 3.1)	2.8 (2.7 to 3.0)
Gujarat	16.1 (14.6 to 17.2)	11.5 (10.2 to 13.1)	2.8 (2.6 to 3.0)	6.8 (6.2 to 7.2)	6.3 (5.7 to 6.7)	5.6 (4.8 to 8.1)	5.2 (4.4 to 5.9)	3.9 (3.2 to 4.9)	4.9 (4.6 to 5.2)	2.0 (1.8 to 2.5)	2.6 (2.4 to 2.8)	3.3 (2.8 to 3.6)	1.4 (1.1 to 2.2)	2.8 (2.6 to 3.0)
Tripura	8.1 (7.6 to 8.6)	7.2 (6.1 to 11.1)	4.6 (4.4 to 4.9)	10.5 (9.7 to 11.3)	6.3 (5.9 to 6.8)	5.1 (4.5 to 7.8)	6.2 (5.6 to 6.8)	3.6 (3.1 to 4.7)	5.5 (5.2 to 5.8)	2.3 (2.2 to 2.6)	3.6 (3.4 to 3.9)	2.3 (2.1 to 3.3)	4.0 (2.4 to 4.5)	2.4 (2.2 to 2.5)
Sikkim	7.0 (6.3 to 7.7)	6.8 (5.4 to 10.4)	12.7 (12.0 to 13.8)	9.7 (8.6 to 10.5)	6.2 (5.5 to 6.7)	4.7 (4.0 to 5.8)	4.5 (3.6 to 5.1)	4.6 (3.8 to 6.1)	9.1 (8.5 to 9.8)	8.1 (6.0 to 9.1)	4.0 (3.7 to 4.4)	2.7 (2.4 to 3.0)	4.1 (2.0 to 4.9)	2.4 (2.2 to 2.5)
Manipur	4.0 (3.8 to 4.3)	7.4 (6.0 to 12.2)	5.5 (5.2 to 5.8)	15.9 (14.5 to 16.9)	6.2 (5.7 to 6.7)	4.4 (3.8 to 7.5)	1.9 (1.7 to 2.1)	4.1 (3.6 to 5.5)	3.2 (3.0 to 3.4)	2.7 (2.5 to 3.0)	1.9 (1.8 to 2.0)	2.6 (2.3 to 3.3)	3.2 (2.3 to 3.5)	2.5 (2.3 to 2.6)
**Higher-middle ETL**	**10.6 (9.9 to 11.1)**	**11.6 (10.3 to 13.3)**	**7.8 (7.5 to 8.1)**	**7.0 (6.6 to 7.4)**	**6.6 (6.0 to 6.9)**	**6.8 (5.6 to 8.0)**	**6.4 (5.7 to 7.0)**	**4.5 (3.5 to 5.4)**	**3.5 (3.3 to 3.6)**	**3.3 (3.1 to 3.4)**	**3.2 (3.0 to 3.3)**	**3.0 (2.6 to 3.2)**	**2.6 (2.2 to 2.9)**	**2.5 (2.5 to 2.6)**
Haryana	14.2 (13.1 to 15.4)	14.0 (12.1 to 15.9)	13.1 (12.4 to 13.8)	8.2 (7.6 to 9.2)	7.9 (7.2 to 8.6)	4.9 (4.1 to 8.2)	17.6 (14.9 to 20.5)	5.1 (4.0 to 6.3)	6.2 (5.7 to 6.7)	3.9 (3.5 to 4.7)	4.5 (3.6 to 5.1)	3.2 (2.8 to 4.0)	2.8 (2.4 to 3.2)	2.8 (2.6 to 3.0)
Delhi	13.1 (11.3 to 14.7)	22.2 (14.0 to 26.6)	3.7 (3.5 to 4.2)	12.1 (10.5 to 13.5)	7.5 (6.7 to 8.2)	7.1 (4.6 to 8.4)	5.2 (4.2 to 6.0)	8.2 (5.4 to 9.7)	4.7 (4.3 to 5.1)	2.8 (2.5 to 3.1)	6.2 (5.5 to 7.0)	4.7 (2.7 to 5.7)	6.2 (2.0 to 7.5)	2.5 (2.4 to 2.7)
Telangana	6.9 (6.4 to 7.4)	7.8 (6.2 to 12.3)	9.6 (9.1 to 10.2)	4.4 (4.0 to 5.3)	5.1 (4.5 to 5.6)	5.7 (4.9 to 7.8)	4.9 (4.2 to 5.7)	3.7 (3.1 to 4.9)	1.0 (0.9 to 1.0)	3.5 (3.0 to 3.9)	2.8 (2.5 to 3.6)	2.7 (2.3 to 3.0)	2.6 (2.2 to 3.0)	2.4 (2.3 to 2.5)
Andhra Pradesh	7.3 (6.9 to 7.9)	8.1 (6.7 to 12.4)	10.5 (9.9 to 11.1)	4.9 (4.5 to 6.1)	5.5 (5.1 to 5.9)	6.0 (5.3 to 8.3)	5.4 (4.7 to 6.1)	3.7 (3.0 to 4.6)	1.1 (1.1 to 1.2)	3.6 (3.2 to 4.0)	3.3 (3.0 to 3.6)	2.9 (2.5 to 3.2)	2.6 (2.3 to 3.0)	2.6 (2.5 to 2.7)
Jammu and Kashmir	3.9 (3.6 to 4.1)	7.6 (6.1 to 11.7)	20.3 (19.1 to 22.0)	15.5 (12.3 to 16.6)	5.9 (5.4 to 6.4)	3.6 (2.9 to 7.6)	6.4 (5.5 to 7.1)	4.5 (5.4 to 3.5)	4.3 (4.0 to 4.6)	3.7 (3.3 to 4.0)	3.3 (3.1 to 3.6)	2.9 (2.6 to 3.2)	2.6 (2.2 to 2.8)	2.6 (2.4 to 2.7)
Karnataka	14.8 (13.5 to 16.2)	12.0 (10.6 to 13.8)	10.5 (9.9 to 11.1)	6.4 (5.8 to 6.8)	6.4 (5.9 to 7.0)	10.5 (6.2 to 12.1)	12.9 (10.9 to 15.0)	4.1 (3.3 to 5.0)	4.1 (3.8 to 4.3)	3.6 (3.3 to 4.0)	3.2 (3.0 to 3.5)	3.2 (2.7 to 3.5)	2.5 (2.1 to 2.9)	2.5 (2.3 to 2.7)
West Bengal	10.7 (9.9 to 11.3)	10.5 (9.1 to 12.3)	7.4 (7.0 to 7.8)	9.9 (9.1 to 10.6)	7.1 (6.5 to 7.6)	5.6 (4.9 to 8.1)	4.9 (4.2 to 5.7)	4.4 (3.3 to 5.2)	2.8 (2.6 to 3.0)	3.1 (2.7 to 3.4)	3.4 (3.2 to 3.8)	2.9 (2.5 to 3.2)	3.4 (2.3 to 3.8)	2.5 (2.4 to 2.7)
Maharashtra	10.0 (9.2 to 10.7)	13.3 (11.5 to 15.1)	3.4 (3.2 to 3.7)	5.3 (4.9 to 5.8)	6.7 (6.1 to 7.2)	7.0 (5.2 to 7.9)	2.9 (2.5 to 3.2)	4.7 (3.7 to 5.8)	4.6 (4.3 to 4.9)	2.9 (2.6 to 3.1)	2.4 (2.2 to 2.6)	2.8 (2.6 to 3.3)	1.4 (1.2 to 2.1)	2.5 (2.4 to 2.7)
UTs* other than Delhi	17.9 (16.6 to 19.1)	8.9 (7.3 to 14.1)	8.7 (8.2 to 9.3)	6.5 (6.0 to 7.0)	6.7 (6.1 to 7.3)	5.7 (4.4 to 6.6)	6.8 (5.9 to 7.9)	4.8 (3.8 to 6.5)	2.5 (2.3 to 2.7)	3.5 (3.0 to 3.8)	3.0 (2.7 to 3.5)	2.7 (2.4 to 3.0)	2.3 (1.9 to 2.7)	3.4 (3.2 to 3.6)
**High ETL**	**9.4 (8.8 to 9.9)**	**14.2 (11.9 to 15.8)**	**4.9 (4.7 to 5.1)**	**7.5 (7.0 to 7.9)**	**7.0 (6.4 to 7.4)**	**6.7 (5.0 to 7.5)**	**3.6 (3.2 to 3.9)**	**4.7 (5.6 to 3.6)**	**3.8 (3.7 to 4.0)**	**2.7 (2.5 to 3.0)**	**2.5 (2.3 to 2.6)**	**3.0 (2.4 to 3.3)**	**1.6 (1.4 to 2.1)**	**2.6 (2.5 to 2.7)**
Himachal Pradesh	8.5 (7.7 to 9.2)	7.4 (5.7 to 13.7)	7.3 (6.8 to 7.9)	6.2 (5.6 to 7.1)	5.5 (4.9 to 6.0)	6.8 (3.9 to 8.2)	10.3 (8.4 to 12.1)	4.8 (3.7 to 5.9)	5.2 (4.9 to 5.6)	3.5 (2.9 to 4.0)	2.8 (2.4 to 3.5)	2.6 (2.2 to 3.1)	2.3 (1.7 to 2.9)	2.6 (2.4 to 2.9)
Punjab	4.5 (4.2 to 4.8)	15.1 (11.8 to 16.8)	2.8 (2.6 to 2.9)	6.7 (6.3 to 7.2)	6.8 (6.3 to 7.3)	6.5 (5.2 to 7.6)	1.8 (1.6 to 1.9)	4.9 (3.8 to 5.9)	6.2 (5.8 to 6.6)	2.5 (2.3 to 2.8)	1.9 (1.8 to 2.1)	3.9 (2.8 to 4.3)	3.1 (2.4 to 3.4)	2.4 (2.3 to 2.6)
Tamil Nadu	9.3 (8.6 to 9.9)	12.2 (10.4 to 14.3)	5.7 (5.4 to 6.0)	6.0 (5.5 to 6.4)	6.6 (6.0 to 7.0)	7.7 (5.3 to 8.9)	3.5 (3.0 to 3.9)	4.4 (3.7 to 5.6)	3.4 (3.2 to 3.7)	2.2 (2.0 to 2.7)	2.1 (2.0 to 2.3)	2.5 (2.3 to 3.2)	1.1 (0.8 to 2.0)	2.4 (2.3 to 2.6)
Goa	9.5 (8.9 to 10.3)	14.4 (11.1 to 16.7)	7.8 (7.4 to 8.3)	4.0 (3.7 to 4.4)	5.3 (4.9 to 5.7)	4.0 (3.4 to 5.5)	6.4 (5.5 to 7.3)	5.1 (4.1 to 6.7)	2.0 (1.8 to 2.1)	3.6 (3.1 to 4.1)	2.9 (2.6 to 3.4)	2.5 (2.1 to 3.0)	2.2 (1.7 to 2.6)	2.5 (2.4 to 2.7)
Kerala	12.9 (11.8 to 13.9)	18.4 (10.3 to 21.6)	4.3 (4.1 to 4.6)	11.0 (10.1 to 11.8)	8.1 (7.4 to 8.7)	5.3 (3.8 to 6.1)	3.7 (3.2 to 4.1)	5.1 (3.3 to 6.0)	2.8 (2.6 to 3.0)	3.5 (3.1 to 3.8)	3.3 (3.1 to 3.6)	3.4 (1.9 to 3.9)	1.3 (1.1 to 1.8)	2.8 (2.6 to 3.0)

Incidence rate per 100,000.

*Union territories. ETL is epidemiological transition level.

Both sexes combined

States of India	Non-Hodgkin lymphoma (95% uncertainty interval)	Ovarian cancer (95% uncertainty interval)	Bladder cancer (95% uncertainty interval)	Non-melanoma skin cancer (95% uncertainty interval)	Brain and nervous system cancer (95% uncertainty interval)	Thyroid cancer (95% uncertainty interval)	Uterine cancer (95% uncertainty interval)	Kidney cancer (95% uncertainty interval)	Multiple myeloma (95% uncertainty interval)	Nasopharynx cancer (95% uncertainty interval)	Hodgkin's lymphoma (95% uncertainty interval)	Malignant skin melanoma (95% uncertainty interval)	Mesothelioma (95% uncertainty interval)	Testicular cancer (95% uncertainty interval)
**India**	2.4 (2.0 to 2.5)	2.4 (2.2 to 2.5)	2.2 (2.1 to 2.2)	2.2 (1.5 to 3.3)	2.0 (1.9 to 2.5)	1.8 (1.7 to 2.0)	1.4 (1.3 to 1.5)	1.3 (1.2 to 1.3)	0.9 (0.8 to 1.0)	0.9 (0.8 to 0.9)	0.6 (0.5 to 0.8)	0.3 (0.3 to 0.4)	0.3 (0.2 to 0.3)	0.2 (0.2 to 0.2)
**Low ETL**	**2.3 (1.9 to 2.5)**	**2.2 (2.0 to 2.3)**	**2.2 (2.0 to 2.3)**	**2.6 (1.7 to 4.1)**	**1.9 (1.7 to 2.3)**	**1.5 (1.3 to 1.6)**	**1.2 (1.1 to 1.3)**	**1.1 (1.1 to 1.2)**	**0.8 (0.7 to 0.9)**	**1.1 (0.9 to 1.2)**	**0.6 (0.5 to 0.9)**	**0.3 (0.2 to 0.3)**	**0.3 (0.2 to 0.4)**	**0.2 (0.1 to 0.2)**
Bihar	2.1 (1.5 to 2.4)	1.8 (1.6 to 2.0)	1.8 (1.6 to 2.2)	2.6 (1.6 to 4.2)	1.7 (1.2 to 2.2)	1.2 (1.0 to 1.3)	1.0 (0.8 to 1.1)	0.9 (0.7 to 1.1)	0.7 (0.6 to 0.9)	1.0 (0.9 to 1.2)	0.5 (0.4 to 0.8)	0.2 (0.2 to 0.3)	0.3 (0.2 to 0.4)	0.0 (0.0 to 0.0)
Jharkhand	2.2 (1.9 to 2.4)	2.0 (1.8 to 2.4)	2.3 (2.1 to 2.4)	2.6 (1.6 to 4.4)	1.9 (1.6 to 2.3)	1.6 (1.4 to 1.9)	1.2 (1.0 to 1.4)	1.1 (1.0 to 1.2)	0.8 (0.7 to 0.9)	1.1 (0.9 to 1.2)	0.6 (0.5 to 0.9)	0.3 (0.2 to 0.3)	0.3 (0.2 to 0.4)	0.2 (0.1 to 0.2)
Uttar Pradesh	2.3 (1.9 to 2.5)	2.0 (1.8 to 2.5)	2.2 (2.0 to 2.5)	2.7 (1.7 to 4.3)	2.0 (1.7 to 2.3)	1.5 (1.2 to 1.7)	1.2 (1.0 to 1.4)	1.1 (1.0 to 1.2)	0.8 (0.7 to 0.9)	1.2 (1.0 to 1.3)	0.7 (0.5 to 1.0)	0.2 (0.2 to 0.3)	0.3 (0.2 to 0.4)	0.2 (0.2 to 0.2)
Rajasthan	2.9 (1.9 to 3.2)	2.0 (1.8 to 2.2)	1.9 (1.7 to 2.0)	2.6 (1.6 to 4.3)	1.9 (1.6 to 2.2)	1.5 (1.4 to 1.6)	1.1 (1.0 to 1.2)	1.1 (1.0 to 1.2)	0.8 (0.7 to 0.9)	1.1 (0.9 to 1.2)	0.5 (0.4 to 0.7)	0.3 (0.2 to 0.3)	0.3 (0.2 to 0.3)	0.0 (0.0 to 0.0)
Meghalaya	1.9 (1.7 to 2.2)	1.4 (1.3 to 2.3)	1.3 (1.2 to 1.4)	2.7 (1.7 to 4.2)	1.6 (1.2 to 2.5)	1.1 (1.0 to 1.9)	0.8 (0.7 to 0.9)	1.2 (1.1 to 1.3)	0.7 (0.6 to 1.0)	1.9 (1.7 to 2.0)	0.3 (0.1 to 0.7)	0.3 (0.3 to 0.4)	0.3 (0.2 to 0.3)	0.1 (0.1 to 0.1)
Assam	2.1 (1.9 to 2.3)	3.1 (2.1 to 3.3)	2.1 (1.9 to 2.3)	2.5 (1.7 to 3.9)	2.1 (1.8 to 2.8)	1.3 (1.2 to 1.9)	1.2 (1.0 to 1.3)	1.6 (1.4 to 1.7)	0.9 (0.7 to 1.1)	0.8 (0.7 to 0.9)	0.4 (0.2 to 1.0)	0.3 (0.3 to 0.4)	0.3 (0.3 to 0.4)	0.3 (0.2 to 0.3)
Chhattisgarh	2.3 (2.0 to 2.5)	2.3 (2.1 to 2.6)	4.3 (3.9 to 4.7)	2.6 (1.7 to 4.3)	2.1 (1.8 to 2.6)	1.9 (1.6 to 2.2)	1.4 (1.2 to 1.6)	1.2 (1.0 to 1.4)	1.0 (0.8 to 1.1)	1.2 (1.0 to 1.3)	0.6 (0.5 to 0.9)	0.3 (0.3 to 0.4)	0.3 (0.2 to 0.4)	0.2 (0.2 to 0.3)
Madhya Pradesh	2.4 (1.9 to 2.6)	2.7 (2.1 to 2.9)	1.9 (1.8 to 2.1)	2.3 (1.5 to 3.4)	2.0 (1.7 to 2.3)	1.4 (1.3 to 1.6)	1.3 (1.2 to 1.5)	1.1 (1.0 to 1.2)	0.9 (0.7 to 1.0)	0.9 (0.7 to 1.0)	0.6 (0.5 to 0.8)	0.3 (0.2 to 0.3)	0.3 (0.2 to 0.4)	0.3 (0.2 to 0.3)
Odisha	2.5 (1.9 to 2.7)	2.2 (1.9 to 2.6)	2.0 (1.8 to 2.2)	2.7 (1.7 to 4.1)	2.0 (1.8 to 2.4)	1.7 (1.4 to 2.0)	1.3 (1.0 to 1.5)	1.2 (1.1 to 1.3)	0.9 (0.7 to 1.0)	1.1 (0.9 to 1.2)	0.6 (0.5 to 0.9)	0.3 (0.2 to 0.3)	0.3 (0.2 to 0.4)	0.2 (0.2 to 0.2)
**Lower-middle ETL**	**2.4 (2.1 to 2.7)**	**2.1 (1.9 to 2.3)**	**2.1 (1.9 to 2.3)**	**1.6 (1.2 to 2.3)**	**1.9 (1.6 to 2.8)**	**1.5 (1.4 to 2.2)**	**1.1 (1.0 to 1.2)**	**1.3 (1.2 to 1.5)**	**0.8 (0.7 to 1.1)**	**1.0 (1.0 to 1.1)**	**0.5 (0.5 to 0.7)**	**0.3 (0.3 to 0.4)**	**0.3 (0.2 to 0.3)**	**0.3 (0.3 to 0.4)**
Arunachal Pradesh	1.8 (1.6 to 2.3)	4.6 (2.1 to 5.1)	1.4 (1.3 to 1.5)	2.8 (1.7 to 4.4)	1.6 (1.2 to 2.3)	4.5 (1.8 to 5.1)	0.6 (0.5 to 0.7)	1.1 (1.1 to 1.3)	0.7 (0.6 to 0.9)	3.5 (3.3 to 3.8)	0.5 (0.4 to 0.7)	0.3 (0.3 to 0.4)	0.3 (0.2 to 0.3)	0.2 (0.2 to 0.3)
Mizoram	2.3 (2.0 to 2.6)	1.8 (1.6 to 2.4)	2.1 (2.0 to 2.3)	2.0 (1.3 to 2.8)	2.0 (1.7 to 2.7)	3.4 (2.2 to 3.7)	1.1 (1.0 to 1.3)	1.1 (1.0 to 1.4)	1.1 (0.8 to 1.3)	4.4 (4.1 to 4.8)	0.4 (0.4 to 0.6)	0.6 (0.3 to 0.7)	0.2 (0.2 to 0.3)	0.2 (0.1 to 0.2)
Nagaland	2.6 (2.2 to 2.8)	1.7 (1.5 to 2.1)	1.3 (1.2 to 1.4)	2.8 (1.8 to 4.3)	1.6 (1.2 to 2.8)	4.7 (2.6 to 5.2)	0.7 (0.6 to 0.8)	1.2 (1.1 to 1.4)	0.7 (0.6 to 1.1)	9.1 (8.2 to 10.1)	0.4 (0.3 to 0.7)	0.4 (0.3 to 0.5)	0.3 (0.2 to 0.3)	0.3 (0.2 to 0.3)
Uttarakhand	2.4 (2.1 to 2.7)	2.3 (2.0 to 2.5)	2.6 (2.4 to 2.8)	2.7 (1.8 to 4.4)	2.2 (1.8 to 3.2)	2.5 (2.3 to 2.9)	1.5 (1.3 to 1.8)	1.4 (1.1 to 1.7)	1.0 (0.8 to 1.2)	1.1 (0.9 to 1.2)	0.5 (0.4 to 0.7)	0.4 (0.3 to 0.5)	0.3 (0.2 to 0.4)	0.3 (0.2 to 0.3)
Gujarat	2.5 (2.1 to 2.8)	2.1 (1.9 to 2.3)	1.6 (1.5 to 1.7)	1.3 (1.0 to 1.7)	1.9 (1.6 to 2.8)	1.1 (1.0 to 2.2)	1.1 (1.0 to 1.2)	1.3 (1.2 to 1.5)	0.8 (0.7 to 1.1)	0.6 (0.6 to 0.7)	0.5 (0.5 to 0.7)	0.3 (0.3 to 0.4)	0.3 (0.2 to 0.3)	0.4 (0.3 to 0.4)
Tripura	1.4 (1.2 to 2.1)	1.7 (1.5 to 2.2)	1.2 (1.1 to 1.3)	2.6 (1.7 to 4.3)	1.7 (1.3 to 2.4)	0.9 (0.8 to 1.6)	0.7 (0.6 to 0.8)	1.1 (1.1 to 1.3)	0.6 (0.5 to 1.0)	0.6 (0.5 to 0.6)	0.3 (0.2 to 0.7)	0.3 (0.3 to 0.4)	0.3 (0.2 to 0.3)	0.2 (0.2 to 0.2)
Sikkim	1.6 (1.4 to 2.4)	2.5 (2.0 to 2.7)	2.3 (2.0 to 2.5)	2.8 (1.8 to 4.4)	3.3 (1.9 to 4.2)	5.3 (2.2 to 6.3)	0.9 (0.8 to 1.1)	1.3 (1.1 to 1.6)	0.8 (0.7 to 1.0)	3.3 (2.9 to 3.8)	0.4 (0.3 to 0.6)	1.3 (0.3 to 1.7)	0.3 (0.2 to 0.3)	0.1 (0.1 to 0.1)
Manipur	2.7 (2.0 to 3.1)	1.8 (1.6 to 2.3)	2.2 (2.0 to 2.4)	2.1 (1.5 to 2.8)	1.6 (1.2 to 2.6)	3.3 (2.1 to 3.6)	0.7 (0.6 to 0.8)	1.1 (1.0 to 1.4)	0.7 (0.6 to 1.0)	3.2 (2.9 to 3.5)	0.3 (0.2 to 0.7)	0.3 (0.3 to 0.4)	0.3 (0.2 to 0.3)	0.1 (0.1 to 0.1)
**Higher-middle ETL**	**2.5 (2.1 to 2.7)**	**2.6 (2.4 to 2.7)**	**2.6 (2.4 to 2.7)**	**2.0 (1.4 to 2.9)**	**2.2 (1.9 to 2.8)**	**2.0 (1.9 to 2.3)**	**1.6 (1.5 to 1.7)**	**1.4 (1.3 to 1.5)**	**0.9 (0.8 to 1.1)**	**0.8 (0.7 to 0.9)**	**0.5 (0.4 to 0.7)**	**0.3 (0.3 to 0.4)**	**0.3 (0.2 to 0.3)**	**0.3 (0.3 to 0.3)**
Haryana	2.5 (2.2 to 3.1)	2.2 (2.0 to 2.7)	2.7 (2.4 to 3.1)	2.7 (1.8 to 4.5)	2.3 (1.9 to 3.6)	2.8 (2.5 to 3.3)	1.7 (1.5 to 1.9)	1.6 (1.3 to 2.1)	1.1 (0.9 to 1.4)	1.2 (1.0 to 1.5)	0.6 (0.5 to 0.9)	0.4 (0.3 to 0.6)	0.3 (0.2 to 0.4)	0.3 (0.3 to 0.4)
Delhi	4.5 (2.8 to 5.3)	3.9 (2.6 to 4.3)	5.4 (4.7 to 6.0)	1.0 (0.8 to 1.1)	3.3 (0.0 to 4.2)	3.6 (3.1 to 4.5)	3.4 (3.9 to 4.0)	2.4 (2.0 to 2.7)	2.1 (0.9 to 2.7)	0.5 (0.4 to 0.6)	1.2 (0.5 to 1.5)	0.6 (0.5 to 0.8)	0.3 (0.2 to 0.4)	0.5 (0.4 to 0.6)
Telangana	2.1 (1.9 to 2.3)	2.1 (1.9 to 2.4)	1.7 (1.5 to 1.9)	2.7 (1.7 to 4.3)	1.9 (1.6 to 2.3)	2.0 (1.7 to 2.4)	1.3 (1.1 to 1.5)	1.2 (1.1 to 1.3)	0.8 (0.7 to 0.9)	1.0 (0.8 to 1.3)	0.5 (0.4 to 0.8)	0.3 (0.3 to 0.4)	0.3 (0.2 to 0.3)	0.2 (0.1 to 0.3)
Andhra Pradesh	2.1 (1.8 to 2.4)	2.1 (1.9 to 2.3)	1.8 (1.7 to 2.0)	2.6 (1.6 to 4.2)	2.0 (1.8 to 2.3)	1.9 (1.7 to 2.1)	1.3 (1.1 to 1.4)	1.3 (1.2 to 1.4)	0.8 (0.7 to 1.0)	1.1 (0.9 to 1.2)	0.5 (0.4 to 0.7)	0.3 (0.3 to 0.4)	0.3 (0.2 to 0.3)	0.2 (0.2 to 0.2)
Jammu and Kashmir	2.3 (2.0 to 2.5)	2.2 (2.0 to 2.4)	2.2 (2.1 to 2.4)	2.6 (1.6 to 4.2)	2.1 (1.9 to 2.5)	1.9 (1.7 to 2.1)	1.2 (1.1 to 1.4)	1.3 (1.2 to 1.4)	0.9 (0.8 to 1.0)	1.0 (0.9 to 1.2)	0.5 (0.4 to 0.7)	0.3 (0.3 to 0.4)	0.3 (0.2 to 0.4)	0.2 (0.2 to 0.2)
Karnataka	2.7 (2.0 to 3.0)	2.6 (2.3 to 3.1)	2.4 (2.2 to 2.6)	2.0 (1.4 to 3.1)	2.2 (1.9 to 2.7)	1.9 (1.7 to 2.2)	1.7 (1.4 to 1.9)	1.3 (1.2 to 1.4)	0.9 (0.8 to 1.1)	1.1 (0.9 to 1.2)	0.5 (0.4 to 0.7)	0.3 (0.3 to 0.4)	0.3 (0.2 to 0.3)	0.2 (0.2 to 0.3)
West Bengal	2.4 (1.9 to 2.7)	2.8 (2.3 to 3.1)	2.4 (2.3 to 2.6)	2.0 (1.4 to 3.1)	2.0 (1.7 to 2.4)	1.5 (1.4 to 1.9)	1.5 (1.3 to 1.7)	1.3 (1.2 to 1.5)	1.0 (0.7 to 1.1)	0.8 (0.7 to 1.0)	0.5 (0.4 to 0.7)	0.3 (0.3 to 0.4)	0.3 (0.2 to 0.4)	0.2 (0.2 to 0.3)
Maharashtra	2.4 (2.1 to 2.7)	2.6 (2.4 to 2.8)	2.1 (1.9 to 2.2)	1.3 (1.1 to 1.5)	2.3 (1.9 to 3.2)	2.1 (1.9 to 2.7)	1.6 (1.4 to 1.8)	1.5 (1.3 to 1.7)	0.9 (0.8 to 1.2)	0.4 (0.3 to 0.4)	0.5 (0.4 to 0.6)	0.3 (0.3 to 0.5)	0.3 (0.2 to 0.3)	0.4 (0.3 to 0.4)
UTs* other than Delhi	4.6 (2.3 to 5.5)	1.8 (1.5 to 2.4)	2.6 (2.3 to 2.9)	2.7 (1.7 to 4.3)	2.2 (1.9 to 3.1)	2.9 (2.5 to 3.4)	1.6 (1.3 to 1.9)	1.4 (1.2 to 1.6)	1.9 (0.8 to 2.5)	0.9 (0.8 to 1.2)	0.4 (0.4 to 0.6)	0.5 (0.4 to 0.6)	0.3 (0.2 to 0.4)	0.3 (0.3 to 0.3)
**High ETL**	**2.5 (2.1 to 2.7)**	**2.7 (2.5 to 2.9)**	**2.7 (2.5 to 2.9)**	**1.7 (1.2 to 2.5)**	**2.1 (1.9 to 2.8)**	**2.6 (2.5 to 2.8)**	**1.9 (1.7 to 2.0)**	**1.3 (1.2 to 1.4)**	**1.3 (0.8 to 1.5)**	**0.6 (0.5 to 0.6)**	**0.5 (0.4 to 0.6)**	**0.5 (0.3 to 0.5)**	**0.3 (0.2 to 0.3)**	**0.2 (0.2 to 0.3)**
Himachal Pradesh	2.3 (1.9 to 2.6)	2.3 (1.7 to 2.6)	2.5 (2.0 to 3.0)	2.6 (1.6 to 4.2)	2.0 (1.8 to 2.6)	2.3 (1.9 to 3.1)	1.3 (1.0 to 1.7)	1.3 (1.2 to 1.5)	0.9 (0.8 to 1.1)	0.9 (0.7 to 1.1)	0.4 (0.3 to 0.6)	0.4 (0.3 to 0.5)	0.3 (0.2 to 0.3)	0.2 (0.2 to 0.3)
Punjab	2.2 (2.0 to 2.5)	3.0 (2.4 to 3.2)	2.0 (1.9 to 2.1)	2.5 (1.5 to 4.1)	2.1 (1.9 to 3.1)	1.3 (1.1 to 2.5)	1.7 (1.5 to 1.9)	1.4 (1.3 to 1.6)	1.1 (0.8 to 1.2)	0.7 (0.6 to 0.8)	0.6 (0.4 to 0.7)	0.3 (0.3 to 0.5)	0.3 (0.2 to 0.3)	0.3 (0.3 to 0.3)
Tamil Nadu	2.0 (1.8 to 2.4)	2.4 (2.2 to 2.5)	1.7 (1.5 to 1.8)	1.3 (1.0 to 1.7)	2.0 (1.6 to 2.9)	1.6 (1.4 to 2.4)	1.5 (1.3 to 1.7)	1.2 (1.1 to 1.4)	0.8 (0.7 to 1.1)	0.5 (0.5 to 0.6)	0.4 (0.4 to 0.6)	0.5 (0.3 to 0.6)	0.3 (0.2 to 0.3)	0.2 (0.2 to 0.2)
Goa	2.4 (2.2 to 2.9)	2.5 (2.3 to 2.8)	2.6 (2.2 to 2.8)	2.6 (1.6 to 4.3)	2.2 (1.9 to 3.2)	3.7 (2.9 to 4.8)	1.9 (1.5 to 2.5)	1.6 (1.4 to 1.8)	1.0 (0.9 to 1.3)	0.9 (0.7 to 1.1)	0.4 (0.3 to 0.5)	0.5 (0.4 to 0.7)	0.3 (0.2 to 0.3)	0.3 (0.3 to 0.4)
Kerala	3.5 (2.0 to 4.1)	3.3 (2.6 to 3.9)	3.1 (2.8 to 3.3)	1.8 (1.3 to 2.6)	2.3 (1.9 to 2.8)	5.5 (2.7 to 6.2)	2.9 (2.5 to 3.2)	1.4 (1.3 to 1.6)	2.3 (0.7 to 3.0)	0.4 (0.4 to 0.5)	0.6 (0.3 to 0.7)	0.4 (0.3 to 0.5)	0.2 (0.2 to 0.3)	0.3 (0.2 to 0.3)

Incidence rate per 100,000.
*Union territories. ETL is epidemiological transition level.

Females

States of India	Breast cancer (95% uncertainty interval)	Cervical cancer (95% uncertainty interval)	Lip and oral cavity cancer (95% uncertainty interval)	Stomach cancer (95% uncertainty interval)	Colon and rectum cancer (95% uncertainty interval)	Ovarian cancer (95% uncertainty interval)	Pharynx cancer other than nasopharynx (95% uncertainty interval)	Lung cancer (95% uncertainty interval)	Gallbladder and biliary tract cancer (95% uncertainty interval)	Thyroid cancer (95% uncertainty interval)	Oesophageal cancer (95% uncertainty interval)	Uterine cancer (95% uncertainty interval)	Leukaemia (95% uncertainty interval)
	Females												
**India**	21.6 (19.5 to 23.7)	13.5 (12.1 to 17.1)	9.2 (8.8 to 9.9)	7.3 (6.9 to 7.7)	6.7 (6.2 to 7.1)	4.8 (4.5 to 5.1)	3.8 (3.6 to 4.1)	3.8 (3.6 to 4.8)	3.3 (2.9 to 3.5)	2.7 (2.5 to 2.8)	3.0 (2.9 to 3.1)	2.8 (2.6 to 3.0)	2.4 (1.9 to 2.8)
**Low ETL**	**18.9 (16.9 to 21.0)**	**13.9 (11.1 to 19.7)**	**10.2 (9.5 to 11.0)**	**9.3 (8.8 to 9.9)**	**7.4 (6.8 to 8.0)**	**4.4 (4.0 to 4.8)**	**4.7 (4.4 to 5.1)**	**4.0 (3.7 to 5.6)**	**4.2 (3.3 to 4.7)**	**2.2 (1.9 to 2.5)**	**3.4 (3.3 to 3.6)**	**2.4 (2.1 to 2.6)**	**2.5 (2.1 to 3.2)**
Bihar	15.7 (13.9 to 17.4)	13.8 (10.5 to 19.9)	4.1 (3.8 to 4.6)	3.5 (3.3 to 3.8)	6.5 (5.8 to 7.1)	3.7 (3.4 to 4.1)	1.3 (1.1 to 1.5)	5.8 (5.3 to 6.5)	3.8 (3.1 to 4.6)	1.7 (1.4 to 2.0)	5.2 (4.7 to 5.7)	2.1 (1.7 to 2.4)	2.0 (1.4 to 3.3)
Jharkhand	17.4 (14.5 to 23.5)	15.6 (11.8 to 20.6)	5.2 (4.7 to 5.7)	9.1 (8.4 to 9.8)	6.9 (6.0 to 7.7)	4.2 (3.8 to 5.0)	3.5 (3.0 to 4.0)	3.4 (2.9 to 5.6)	4.0 (3.2 to 4.7)	2.5 (2.0 to 2.9)	0.8 (0.7 to 0.9)	2.6 (2.0 to 3.0)	2.8 (2.1 to 3.4)
Uttar Pradesh	21.1 (17.4 to 24.8)	14.6 (11.1 to 21.4)	11.5 (10.3 to 13.3)	14.7 (13.5 to 16.0)	8.8 (7.8 to 10.1)	4.2 (3.8 to 5.1)	5.7 (4.9 to 6.5)	3.9 (3.4 to 6.2)	4.1 (3.3 to 4.9)	2.3 (1.8 to 2.7)	2.2 (2.0 to 2.3)	2.5 (2.0 to 2.9)	3.0 (2.2 to 3.6)
Rajasthan	17.9 (15.8 to 20.0)	13.1 (10.7 to 16.7)	6.5 (5.9 to 7.3)	8.4 (7.6 to 9.1)	5.9 (5.3 to 6.5)	3.9 (3.5 to 4.3)	7.6 (6.6 to 8.7)	3.3 (2.9 to 5.4)	3.5 (3.0 to 3.9)	2.1 (1.9 to 2.3)	2.8 (2.6 to 3.0)	2.1 (1.9 to 2.4)	1.6 (1.2 to 2.8)
Meghalaya	14.2 (11.5 to 22.4)	9.0 (7.7 to 15.2)	12.4 (11.4 to 13.4)	6.3 (5.9 to 6.8)	5.5 (5.0 to 6.0)	2.7 (2.5 to 4.4)	3.5 (3.2 to 3.8)	4.3 (3.9 to 4.7)	4.6 (2.9 to 5.1)	1.2 (1.0 to 2.6)	20.2 (18.7 to 21.9)	1.5 (1.4 to 1.7)	1.7 (1.4 to 2.8)
Assam	19.4 (16.1 to 26.8)	10.1 (8.2 to 20.7)	8.4 (7.7 to 9.2)	4.3 (4.0 to 4.6)	7.4 (6.4 to 8.3)	6.3 (4.3 to 6.9)	4.2 (3.7 to 4.8)	4.6 (4.2 to 5.1)	9.4 (3.5 to 10.8)	2.0 (1.7 to 2.8)	10.0 (9.2 to 10.8)	2.4 (2.1 to 2.7)	2.4 (2.0 to 3.4)
Chhattisgarh	17.8 (14.7 to 24.8)	17.6 (13.8 to 20.7)	9.7 (8.8 to 10.6)	5.8 (5.3 to 6.2)	6.5 (5.7 to 7.2)	4.4 (4.0 to 5.0)	6.5 (5.7 to 7.3)	3.3 (2.9 to 5.3)	3.9 (3.2 to 4.4)	2.9 (2.4 to 3.4)	4.0 (3.7 to 4.3)	2.7 (2.2 to 3.1)	2.7 (2.1 to 3.2)
Madhya Pradesh	20.6 (17.8 to 23.2)	15.9 (12.6 to 18.8)	17.9 (16.4 to 19.6)	7.1 (6.5 to 7.7)	6.6 (5.8 to 7.3)	5.4 (4.1 to 5.9)	3.8 (3.3 to 4.5)	3.7 (3.3 to 4.5)	4.2 (3.1 to 4.6)	2.1 (1.9 to 2.3)	3.8 (3.5 to 4.1)	2.6 (2.3 to 2.9)	2.6 (2.0 to 3.0)
Odisha	17.2 (14.0 to 24.5)	9.3 (7.6 to 20.1)	13.2 (12.1 to 14.6)	9.3 (8.5 to 10.1)	8.5 (7.3 to 9.5)	4.4 (3.9 to 5.2)	4.6 (4.0 to 5.3)	3.5 (3.1 to 5.1)	3.9 (3.0 to 4.6)	2.6 (2.1 to 3.0)	2.2 (2.0 to 2.4)	2.6 (2.0 to 3.0)	2.6 (1.9 to 3.2)
**Lower-middle ETL**	**20.8 (18.4 to 25.0)**	**11.3 (10.1 to 16.0)**	**7.5 (7.0 to 8.2)**	**4.0 (3.8 to 4.2)**	**6.1 (5.4 to 6.6)**	**4.1 (3.8 to 4.5)**	**2.1 (1.9 to 2.3)**	**4.2 (3.9 to 4.6)**	**2.0 (1.9 to 2.7)**	**2.2 (2.0 to 3.2)**	**3.5 (3.2 to 3.7)**	**2.1 (1.9 to 2.4)**	**2.4 (1.8 to 2.7)**
Arunachal Pradesh	15.9 (13.1 to 21.6)	14.9 (11.5 to 16.8)	6.4 (5.8 to 6.9)	21.4 (19.9 to 23.0)	5.5 (4.9 to 6.2)	10.1 (4.5 to 11.2)	1.3 (1.1 to 1.5)	6.6 (4.5 to 7.2)	4.5 (3.1 to 5.0)	7.9 (2.7 to 8.9)	6.5 (6.0 to 7.1)	1.3 (1.1 to 1.4)	3.2 (1.8 to 3.7)
Mizoram	20.8 (17.8 to 23.7)	14.5 (10.3 to 16.4)	3.7 (3.4 to 4.0)	22.2 (20.8 to 23.7)	7.9 (7.1 to 8.5)	3.6 (3.3 to 4.8)	2.7 (2.5 to 2.9)	30.4 (16.9 to 33.7)	4.1 (2.6 to 4.5)	5.0 (3.3 to 5.6)	5.1 (4.8 to 5.5)	2.3 (2.0 to 2.6)	2.4 (1.7 to 2.7)
Nagaland	15.6 (13.0 to 25.9)	11.3 (9.4 to 13.3)	3.0 (2.8 to 3.3)	15.3 (14.3 to 16.3)	6.0 (5.3 to 6.7)	3.7 (3.4 to 4.6)	1.4 (1.3 to 1.6)	4.6 (4.2 to 5.0)	2.6 (2.3 to 2.9)	8.4 (3.8 to 9.5)	3.8 (3.5 to 4.1)	1.5 (1.4 to 1.7)	1.9 (1.5 to 3.0)
Uttarakhand	19.3 (14.9 to 27.4)	13.2 (11.2 to 16.8)	6.5 (6.0 to 7.0)	11.3 (10.3 to 12.6)	6.9 (5.7 to 8.1)	4.5 (4.0 to 5.0)	5.8 (5.0 to 7.1)	5.9 (4.4 to 6.6)	3.4 (2.9 to 3.8)	3.8 (3.4 to 4.3)	2.4 (2.2 to 2.7)	3.0 (2.6 to 3.4)	2.3 (1.8 to 2.7)
Gujarat	22.1 (19.6 to 25.3)	11.1 (9.7 to 16.2)	8.3 (7.5 to 9.2)	2.0 (1.9 to 2.2)	6.0 (5.4 to 6.6)	4.0 (3.7 to 4.6)	1.6 (1.4 to 1.7)	3.2 (2.9 to 3.7)	1.4 (1.2 to 2.7)	1.5 (1.3 to 3.3)	3.6 (3.3 to 3.9)	2.1 (1.9 to 2.4)	2.5 (1.8 to 2.9)
Tripura	14.0 (11.8 to 22.0)	10.5 (9.2 to 16.0)	5.1 (4.7 to 5.5)	3.3 (3.1 to 3.5)	5.6 (5.1 to 6.2)	3.4 (3.1 to 4.4)	1.6 (1.5 to 1.8)	3.5 (3.2 to 4.1)	5.6 (2.9 to 6.2)	1.2 (1.1 to 2.3)	3.6 (3.3 to 3.8)	1.4 (1.2 to 1.5)	1.9 (1.6 to 2.7)
Sikkim	14.7 (11.7 to 23.2)	10.9 (9.3 to 13.4)	5.2 (4.7 to 5.7)	11.1 (10.3 to 12.1)	5.2 (4.4 to 6.1)	5.7 (4.6 to 6.2)	2.9 (2.5 to 3.4)	10.0 (7.3 to 11.3)	6.4 (2.6 to 7.7)	9.1 (3.4 to 11.2)	8.1 (7.4 to 8.9)	2.1 (1.8 to 2.5)	2.0 (1.5 to 2.5)
Manipur	14.6 (11.8 to 24.1)	9.0 (7.6 to 15.2)	2.8 (2.5 to 3.1)	3.8 (3.6 to 4.1)	5.4 (4.8 to 6.0)	3.7 (3.3 to 4.6)	0.7 (0.6 to 0.7)	12.4 (9.9 to 13.7)	4.5 (2.7 to 4.9)	5.4 (3.1 to 6.1)	2.3 (2.1 to 2.5)	1.4 (1.3 to 1.6)	1.9 (1.7 to 2.8)
**Higher-middle ETL**	**22.4 (19.9 to 26.0)**	**13.6 (11.3 to 16.0)**	**9.2 (8.8 to 10.0)**	**7.4 (7.0 to 7.8)**	**6.2 (5.7 to 6.7)**	**5.1 (4.7 to 5.4)**	**4.1 (3.8 to 4.5)**	**3.8 (3.5 to 4.4)**	**3.2 (2.8 to 3.5)**	**3.0 (2.8 to 3.3)**	**2.7 (2.6 to 2.9)**	**3.1 (2.9 to 3.3)**	**2.3 (1.7 to 2.5)**
Haryana	27.5 (23.6 to 31.4)	10.0 (8.5 to 16.7)	11.0 (10.0 to 12.4)	14.2 (13.2 to 15.3)	7.0 (6.2 to 7.9)	4.5 (4.0 to 5.4)	11.8 (10.1 to 13.6)	3.9 (3.5 to 5.8)	3.5 (3.0 to 4.0)	4.3 (3.8 to 4.9)	3.7 (3.3 to 4.0)	3.3 (2.9 to 3.8)	2.5 (1.9 to 2.8)
Delhi	45.1 (28.6 to 54.0)	14.8 (9.8 to 17.6)	6.4 (5.7 to 7.1)	2.4 (2.2 to 2.7)	6.5 (5.5 to 7.4)	8.1 (5.4 to 9.0)	1.7 (1.5 to 1.9)	4.7 (4.0 to 5.3)	8.9 (2.5 to 10.4)	5.3 (4.4 to 7.0)	3.2 (2.9 to 3.5)	7.1 (6.0 to 8.3)	3.2 (1.5 to 4.2)
Telangana	14.7 (11.6 to 23.4)	11.2 (9.8 to 15.3)	8.7 (7.9 to 9.6)	11.2 (10.3 to 12.2)	5.0 (4.3 to 5.7)	4.0 (3.6 to 4.5)	4.5 (3.8 to 5.3)	2.5 (2.1 to 4.2)	3.2 (2.7 to 3.7)	2.9 (2.5 to 3.6)	0.8 (0.7 to 0.8)	2.4 (2.1 to 2.8)	2.2 (1.6 to 2.5)
Andhra Pradesh	15.1 (12.4 to 23.6)	11.9 (10.4 to 16.3)	8.8 (8.1 to 9.7)	12.0 (11.1 to 12.9)	5.2 (4.6 to 5.8)	4.1 (3.7 to 4.5)	4.7 (4.1 to 5.4)	2.6 (2.3 to 4.8)	3.2 (2.6 to 3.7)	2.7 (2.4 to 3.1)	0.8 (0.8 to 0.9)	2.4 (2.1 to 2.8)	2.2 (1.7 to 2.6)
Jammu and Kashmir	15.3 (12.3 to 23.9)	7.6 (6.1 to 16.1)	3.1 (2.8 to 3.3)	17.2 (15.9 to 18.7)	6.0 (5.3 to 6.7)	4.6 (4.2 to 5.0)	5.3 (4.6 to 6.1)	10.6 (4.5 to 11.9)	3.3 (2.8 to 3.6)	2.9 (2.5 to 3.2)	3.8 (3.5 to 4.2)	2.6 (2.2 to 2.9)	2.3 (1.8 to 2.6)
Karnataka	22.9 (20.2 to 26.3)	20.9 (12.1 to 24.0)	15.2 (13.7 to 17.8)	9.8 (9.0 to 10.7)	6.0 (5.4 to 6.8)	5.1 (4.5 to 5.9)	9.4 (7.9 to 11.0)	3.5 (3.1 to 4.0)	3.0 (2.4 to 3.7)	2.9 (2.5 to 3.3)	3.7 (3.4 to 4.0)	3.2 (2.7 to 3.7)	2.6 (1.8 to 3.0)
West Bengal	20.9 (18.1 to 24.6)	11.4 (10.0 to 16.6)	8.3 (7.7 to 9.0)	6.2 (5.7 to 6.7)	7.3 (6.5 to 8.1)	5.7 (4.6 to 6.2)	2.2 (1.9 to 2.5)	5.1 (4.7 to 5.6)	4.6 (2.8 to 5.1)	2.2 (2.0 to 2.7)	2.1 (1.9 to 2.2)	3.0 (2.7 to 3.4)	2.3 (1.8 to 2.7)
Maharashtra	25.8 (22.3 to 29.5)	14.0 (10.5 to 15.9)	7.2 (6.6 to 8.1)	2.4 (2.2 to 2.6)	6.0 (5.4 to 6.7)	5.2 (4.7 to 5.7)	1.2 (1.1 to 1.3)	3.0 (2.7 to 3.7)	1.4 (1.3 to 2.4)	3.1 (2.7 to 3.9)	3.8 (3.5 to 4.2)	3.2 (2.8 to 3.6)	2.1 (1.6 to 2.5)
UTs* other than Delhi	17.5 (14.3 to 28.4)	11.9 (9.2 to 13.8)	15.7 (14.5 to 17.2)	9.4 (8.7 to 10.3)	7.3 (6.3 to 8.3)	3.6 (3.0 to 4.9)	4.7 (4.0 to 5.6)	4.0 (3.6 to 4.5)	2.8 (2.3 to 3.4)	4.5 (3.6 to 5.5)	2.3 (2.1 to 2.5)	3.1 (2.6 to 3.8)	1.9 (1.4 to 2.4)
**High ETL**	**27.2 (22.4 to 30.1)**	**13.2 (9.7 to 14.7)**	**7.1 (6.6 to 7.7)**	**3.1 (2.9 to 3.3)**	**6.1 (5.6 to 6.6)**	**5.3 (4.8 to 5.7)**	**1.5 (1.4 to 1.6)**	**3.2 (3.0 to 3.6)**	**1.7 (1.5 to 2.4)**	**3.9 (3.5 to 4.2)**	**2.5 (2.4 to 2.7)**	**3.6 (3.3 to 4.0)**	**2.4 (1.5 to 2.8)**
Himachal Pradesh	14.0 (10.7 to 26.5)	13.5 (7.7 to 16.3)	5.0 (4.5 to 5.5)	6.8 (6.3 to 7.6)	4.1 (3.5 to 4.7)	4.5 (3.4 to 5.1)	6.8 (5.8 to 8.2)	2.4 (2.1 to 4.2)	2.7 (2.0 to 3.6)	3.4 (2.6 to 4.8)	4.1 (3.6 to 4.6)	2.4 (2.0 to 3.4)	1.9 (1.2 to 2.6)
Punjab	30.0 (23.2 to 33.5)	13.1 (10.5 to 15.4)	2.2 (2.0 to 2.4)	1.8 (1.7 to 1.9)	6.9 (6.3 to 7.7)	6.0 (4.9 to 6.5)	0.5 (0.4 to 0.5)	3.4 (3.1 to 4.2)	4.1 (2.8 to 4.5)	2.0 (1.7 to 3.7)	5.0 (4.6 to 5.5)	3.4 (3.1 to 3.8)	3.4 (1.9 to 3.9)
Tamil Nadu	23.5 (20.1 to 27.5)	15.1 (10.5 to 17.4)	7.1 (6.6 to 7.7)	3.6 (3.3 to 3.9)	5.9 (5.2 to 6.5)	4.6 (4.3 to 5)	1.5 (1.4 to 1.7)	2.8 (2.5 to 3.3)	0.9 (0.8 to 2.4)	2.3 (2.0 to 3.4)	2.2 (2.0 to 2.4)	2.8 (2.5 to 3.2)	1.9 (1.6 to 2.5)
Goa	26.9 (20.4 to 31.1)	7.9 (6.7 to 10.6)	10.1 (9.2 to 11.1)	7.8 (7.3 to 8.4)	5.0 (4.5 to 5.6)	4.8 (4.4 to 5.3)	5.2 (4.6 to 5.9)	2.6 (2.3 to 3.2)	2.6 (2.0 to 3.3)	5.5 (4.1 to 7.6)	1.7 (1.6 to 1.9)	3.5 (2.9 to 4.7)	1.8 (1.1 to 2.4)
Kerala	33.6 (18.5 to 39.5)	9.9 (7.1 to 11.3)	10.0 (9.0 to 11.6)	2.4 (2.2 to 2.6)	6.6 (5.9 to 7.3)	6.2 (4.7 to 7.3)	1.0 (0.9 to 1.1)	3.9 (3.4 to 4.5)	1.2 (1.1 to 1.9)	7.8 (3.6 to 9.0)	1.4 (1.3 to 1.5)	5.3 (4.6 to 6.0)	2.7 (1.1 to 3.2)

Incidence rate per 100,000.
*Union territories. ETL is epidemiological transition level.

Females

States of India	Non-Hodgkin lymphoma (95% uncertainty interval)	Pancreatic cancer (95% uncertainty interval)	Brain and nervous system cancer (95% uncertainty interval)	Liver cancer (95% uncertainty interval)	Non-melanoma skin cancer (95% uncertainty interval)	Larynx cancer (95% uncertainty interval)	Bladder cancer (95% uncertainty interval)	Kidney cancer (95% uncertainty interval)	Multiple myeloma (95% uncertainty interval)	Nasopharynx cancer (95% uncertainty interval)	Hodgkin's lymphoma (95% uncertainty interval)	Malignant skin melanoma (95% uncertainty interval)	Mesothelioma (95% uncertainty interval)
**India**	2.0 (1.6 to 2.0)	2.2 (2.1 to 2.4)	1.7 (1.4 to 2.2)	2.0 (1.7 to 2.2)	1.8 (1.1 to 2.8)	1.0 (1.0 to 1.1)	1.1 (1.1 to 1.2)	0.9 (0.8 to 0.9)	0.9 (0.7 to 1.0)	0.5 (0.4 to 0.5)	0.4 (0.3 to 0.5)	0.3 (0.2 to 0.3)	0.1 (0.1 to 0.2)
**Low ETL**	**1.9 (1.5 to 2.1)**	**2.3 (2.2 to 2.4)**	**1.6 (1.4 to 2.0)**	**2.2 (1.7 to 2.7)**	**2.2 (1.3 to 3.6)**	**1.3 (1.2 to 1.4)**	**1.2 (1.1 to 1.3)**	**0.8 (0.8 to 0.9)**	**0.8 (0.7 to 1.0)**	**0.6 (0.5 to 0.7)**	**0.4 (0.3 to 0.6)**	**0.2 (0.2 to 0.3)**	**0.2 (0.1 to 0.2)**
Bihar	1.7 (1.4 to 2.0)	2.0 (1.8 to 2.2)	1.5 (1.1 to 1.9)	2.3 (1.7 to 2.9)	2.2 (1.3 to 3.7)	1.3 (1.1 to 1.4)	1.1 (0.9 to 1.3)	0.7 (0.6 to 0.8)	0.8 (0.6 to 0.9)	0.6 (0.5 to 0.8)	0.4 (0.3 to 0.6)	0.2 (0.2 to 0.3)	0.2 (0.1 to 0.3)
Jharkhand	1.9 (1.5 to 2.1)	2.3 (2.0 to 2.6)	1.7 (1.4 to 2.1)	2.3 (1.7 to 3.0)	2.2 (1.3 to 3.7)	1.3 (1.2 to 1.5)	1.5 (1.3 to 1.7)	0.8 (0.7 to 1.0)	0.9 (0.7 to 1.1)	0.6 (0.5 to 0.8)	0.4 (0.4 to 0.7)	0.2 (0.2 to 0.3)	0.2 (0.1 to 0.2)
Uttar Pradesh	1.8 (1.5 to 2.1)	2.2 (2.0 to 2.5)	1.6 (1.4 to 2.0)	2.3 (1.7 to 3.1)	2.2 (1.3 to 3.8)	1.4 (1.3 to 1.6)	1.2 (1.0 to 1.4)	0.8 (0.7 to 1.0)	0.8 (0.7 to 1.0)	0.7 (0.5 to 0.8)	0.5 (0.4 to 0.7)	0.2 (0.2 to 0.3)	0.2 (0.1 to 0.3)
Rajasthan	2.5 (1.5 to 2.7)	2.2 (2.0 to 2.5)	1.5 (1.2 to 1.9)	2.1 (1.6 to 2.5)	2.2 (1.3 to 3.9)	1.1 (1.0 to 1.2)	1.0 (0.9 to 1.1)	0.8 (0.7 to 0.9)	0.8 (0.6 to 0.9)	0.5 (0.4 to 0.6)	0.3 (0.2 to 0.5)	0.2 (0.2 to 0.3)	0.1 (0.1 to 0.2)
Meghalaya	1.6 (1.4 to 1.7)	2.5 (2.3 to 2.8)	1.4 (1.0 to 2.1)	1.6 (1.4 to 1.9)	2.3 (1.4 to 3.8)	1.4 (1.3 to 1.5)	0.7 (0.6 to 0.7)	0.8 (0.7 to 0.9)	0.7 (0.6 to 1.0)	0.8 (0.7 to 0.8)	0.2 (0.1 to 0.5)	0.3 (0.2 to 0.4)	0.1 (0.1 to 0.2)
Assam	1.6 (1.4 to 1.8)	3.0 (2.8 to 3.4)	1.8 (1.4 to 2.5)	2.2 (1.7 to 2.5)	2.1 (1.3 to 3.3)	1.2 (1.1 to 1.3)	0.9 (0.8 to 1.0)	1.2 (1.1 to 1.4)	0.8 (0.7 to 1.1)	0.3 (0.3 to 0.3)	0.3 (0.1 to 0.7)	0.3 (0.2 to 0.3)	0.2 (0.1 to 0.3)
Chhattisgarh	1.8 (1.6 to 2.0)	2.9 (2.7 to 3.1)	1.7 (1.4 to 2.2)	2.3 (1.7 to 2.8)	2.2 (1.3 to 3.7)	1.3 (1.2 to 1.4)	3.3 (2.8 to 3.9)	0.9 (0.7 to 1.0)	1.0 (0.7 to 1.1)	0.6 (0.5 to 0.7)	0.4 (0.3 to 0.7)	0.3 (0.2 to 0.3)	0.1 (0.1 to 0.2)
Madhya Pradesh	1.9 (1.5 to 2.1)	2.1 (2.0 to 2.3)	1.6 (1.4 to 2.0)	1.9 (1.5 to 2.3)	2.0 (1.3 to 3.0)	1.1 (1.0 to 1.2)	1.0 (0.9 to 1.1)	0.8 (0.7 to 0.9)	0.9 (0.7 to 1.0)	0.4 (0.4 to 0.5)	0.4 (0.3 to 0.6)	0.3 (0.2 to 0.3)	0.1 (0.1 to 0.2)
Odisha	2.0 (1.5 to 2.2)	2.4 (2.1 to 2.7)	1.7 (1.4 to 2.2)	2.3 (1.7 to 3.0)	2.2 (1.3 to 3.6)	1.3 (1.2 to 1.4)	1.2 (1.0 to 1.4)	0.9 (0.7 to 1.0)	0.9 (0.7 to 1.1)	0.6 (0.4 to 0.8)	0.4 (0.3 to 0.7)	0.2 (0.2 to 0.3)	0.2 (0.1 to 0.2)
**Lower-middle ETL**	**1.9 (1.6 to 2.1)**	**2.4 (2.2 to 2.6)**	**1.6 (1.3 to 2.4)**	**1.5 (1.3 to 2.1)**	**1.2 (0.8 to 1.7)**	**0.6 (0.5 to 0.6)**	**0.8 (0.8 to 0.9)**	**0.9 (0.8 to 1.0)**	**0.8 (0.7 to 1.1)**	**0.5 (0.4 to 0.5)**	**0.3 (0.3 to 0.5)**	**0.3 (0.2 to 0.4)**	**0.1 (0.1 to 0.2)**
Arunachal Pradesh	1.9 (1.6 to 2.2)	2.7 (2.5 to 3.0)	1.3 (1.1 to 1.9)	7.3 (1.6 to 8.9)	2.2 (1.3 to 3.6)	1.2 (1.1 to 1.3)	0.7 (0.6 to 0.7)	0.8 (0.7 to 0.9)	0.5 (0.4 to 1.0)	2.5 (2.3 to 2.8)	0.3 (0.2 to 0.4)	0.3 (0.2 to 0.4)	0.1 (0.1 to 0.2)
Mizoram	1.8 (1.6 to 2.0)	2.5 (2.3 to 2.7)	1.9 (1.4 to 2.3)	5.4 (1.7 to 6.5)	1.6 (1.0 to 2.5)	2.0 (1.9 to 2.2)	1.5 (1.4 to 1.7)	0.8 (0.7 to 0.9)	1.0 (0.7 to 1.2)	2.6 (2.4 to 2.9)	0.2 (0.1 to 0.4)	0.5 (0.2 to 0.6)	0.1 (0.1 to 0.1)
Nagaland	2.1 (1.6 to 2.3)	2.1 (1.9 to 2.3)	1.4 (1.0 to 2.2)	2.6 (1.5 to 3.0)	2.2 (1.3 to 3.6)	0.8 (0.7 to 0.9)	0.6 (0.6 to 0.7)	0.7 (0.6 to 0.8)	0.5 (0.4 to 1.0)	2.1 (1.8 to 2.4)	0.2 (0.1 to 0.5)	0.3 (0.3 to 0.4)	0.1 (0.1 to 0.1)
Uttarakhand	1.9 (1.6 to 2.0)	2.5 (2.3 to 2.7)	1.8 (1.4 to 2.6)	2.1 (1.6 to 2.5)	2.2 (1.3 to 3.7)	1.0 (0.9 to 1.1)	1.5 (1.3 to 1.7)	1.0 (0.8 to 1.1)	1.0 (0.7 to 1.2)	0.5 (0.4 to 0.6)	0.3 (0.3 to 0.5)	0.3 (0.3 to 0.4)	0.1 (0.1 to 0.2)
Gujarat	1.9 (1.6 to 2.2)	2.4 (2.2 to 2.7)	1.6 (1.2 to 2.4)	1.2 (1.0 to 2.1)	0.9 (0.6 to 1.2)	0.4 (0.4 to 0.5)	0.7 (0.6 to 0.8)	0.9 (0.8 to 1.1)	0.8 (0.7 to 1.1)	0.3 (0.3 to 0.3)	0.4 (0.3 to 0.5)	0.3 (0.2 to 0.4)	0.1 (0.1 to 0.2)
Tripura	1.1 (0.9 to 1.7)	2.1 (2.0 to 2.3)	1.4 (1.1 to 2.0)	1.6 (1.4 to 1.9)	2.2 (1.3 to 3.6)	0.9 (0.9 to 1.0)	0.7 (0.6 to 0.8)	0.8 (0.7 to 0.9)	0.6 (0.5 to 1.0)	0.3 (0.3 to 0.3)	0.3 (0.2 to 0.5)	0.2 (0.2 to 0.3)	0.1 (0.1 to 0.2)
Sikkim	1.5 (1.3 to 1.9)	2.0 (1.8 to 2.2)	3.7 (1.4 to 5.3)	6.2 (1.7 to 7.7)	2.3 (1.4 to 3.7)	2.2 (2.0 to 2.4)	0.8 (0.7 to 0.9)	1.0 (0.9 to 1.1)	0.9 (0.7 to 1.1)	2.0 (1.8 to 2.3)	0.1 (0.1 to 0.4)	1.3 (0.2 to 1.7)	0.1 (0.1 to 0.1)
Manipur	2.4 (1.6 to 2.7)	2.2 (2.0 to 2.5)	1.4 (1.0 to 2.2)	1.7 (1.5 to 2.2)	1.8 (1.3 to 2.6)	0.9 (0.8 to 1.0)	1.1 (1.0 to 1.2)	0.8 (0.7 to 0.9)	0.7 (0.6 to 1.0)	1.5 (1.4 to 1.7)	0.2 (0.1 to 0.5)	0.2 (0.2 to 0.4)	0.1 (0.1 to 0.2)
**Higher-middle ETL**	**2.0 (1.6 to 2.2)**	**2.2 (2.1 to 2.4)**	**1.8 (1.4 to 2.4)**	**2.0 (1.6 to 2.2)**	**1.6 (1.1 to 2.5)**	**1.0 (1.0 to 1.1)**	**1.2 (1.1 to 1.2)**	**1.0 (0.9 to 1.0)**	**0.9 (0.8 to 1.1)**	**0.4 (0.3 to 0.4)**	**0.4 (0.3 to 0.5)**	**0.3 (0.3 to 0.4)**	**0.1 (0.1 to 0.2)**
Haryana	1.9 (1.7 to 2.2)	2.5 (2.2 to 2.9)	1.9 (1.4 to 2.8)	2.3 (1.8 to 2.7)	2.2 (1.3 to 3.9)	1.2 (1.1 to 1.3)	1.4 (1.3 to 1.6)	1.0 (0.9 to 1.3)	1.1 (0.8 to 1.3)	0.6 (0.5 to 0.7)	0.4 (0.3 to 0.6)	0.3 (0.3 to 0.4)	0.1 (0.1 to 0.2)
Delhi	3.7 (2.2 to 4.2)	2.1 (1.9 to 2.3)	2.6 (1.5 to 3.6)	1.7 (1.4 to 2.1)	0.7 (0.6 to 0.9)	1.3 (1.1 to 1.4)	2.3 (2.0 to 2.6)	1.3 (1.2 to 1.6)	1.9 (0.9 to 2.3)	0.2 (0.2 to 0.2)	0.7 (0.3 to 1.0)	0.6 (0.4 to 0.7)	0.1 (0.1 to 0.2)
Telangana	1.9 (1.5 to 2.1)	2.1 (1.9 to 2.3)	1.6 (1.4 to 2.0)	2.1 (1.6 to 2.4)	2.3 (1.3 to 3.8)	0.9 (0.8 to 1.0)	1.0 (0.9 to 1.1)	0.8 (0.8 to 0.9)	0.8 (0.7 to 1.0)	0.5 (0.4 to 0.6)	0.3 (0.2 to 0.5)	0.3 (0.2 to 0.4)	0.1 (0.1 to 0.2)
Andhra Pradesh	1.9 (1.5 to 2.1)	2.2 (2.1 to 2.4)	1.6 (1.4 to 2.0)	2.2 (1.7 to 2.6)	2.2 (1.3 to 3.6)	1.1 (1.0 to 1.2)	1.1 (0.9 to 1.2)	0.9 (0.8 to 1.0)	0.8 (0.7 to 1.0)	0.5 (0.4 to 0.6)	0.3 (0.3 to 0.5)	0.3 (0.2 to 0.3)	0.1 (0.1 to 0.2)
Jammu and Kashmir	1.9 (1.6 to 2.1)	2.3 (2.1 to 2.5)	1.7 (1.4 to 2.1)	2.2 (1.7 to 2.5)	2.2 (1.3 to 3.7)	1.1 (1.0 to 1.2)	1.2 (1.0 to 1.3)	0.9 (0.8 to 1.0)	0.9 (0.7 to 1.0)	0.5 (0.4 to 0.6)	0.3 (0.3 to 0.5)	0.3 (0.2 to 0.4)	0.2 (0.1 to 0.2)
Karnataka	2.2 (1.6 to 2.4)	2.3 (2.0 to 2.6)	1.9 (1.4 to 2.3)	2.3 (1.7 to 2.7)	1.7 (1.1 to 2.8)	1.2 (1.1 to 1.3)	1.3 (1.1 to 1.5)	0.9 (0.8 to 1.1)	0.9 (0.7 to 1.1)	0.6 (0.4 to 0.7)	0.3 (0.3 to 0.5)	0.2 (0.2 to 0.3)	0.1 (0.1 to 0.2)
West Bengal	2.0 (1.5 to 2.2)	2.2 (2.1 to 2.4)	1.6 (1.4 to 2.2)	2.0 (1.6 to 2.3)	1.7 (1.1 to 2.7)	1.2 (1.1 to 1.2)	1.1 (1.1 to 1.2)	0.9 (0.9 to 1.0)	0.9 (0.7 to 1.0)	0.4 (0.3 to 0.5)	0.4 (0.3 to 0.5)	0.3 (0.2 to 0.3)	0.2 (0.1 to 0.2)
Maharashtra	1.9 (1.6 to 2.2)	2.2 (2.0 to 2.5)	1.8 (1.4 to 2.6)	1.7 (1.5 to 2.0)	1.0 (0.8 to 1.2)	0.8 (0.7 to 0.8)	1.0 (0.9 to 1.2)	1.0 (0.9 to 1.2)	0.8 (0.7 to 1.1)	0.2 (0.2 to 0.2)	0.4 (0.2 to 0.5)	0.3 (0.2 to 0.5)	0.1 (0.1 to 0.2)
UTs* other than Delhi	3.7 (1.9 to 4.2)	2.6 (2.3 to 2.8)	1.8 (1.5 to 2.5)	2.0 (1.5 to 2.4)	2.3 (1.3 to 3.8)	0.9 (0.8 to 1.0)	1.5 (1.2 to 1.8)	1.0 (0.9 to 1.1)	1.2 (0.8 to 1.4)	0.5 (0.4 to 0.5)	0.3 (0.2 to 0.4)	0.4 (0.3 to 0.5)	0.1 (0.1 to 0.1)
**High ETL**	**1.9 (1.6 to 2.1)**	**2.1 (2.0 to 2.3)**	**1.7 (1.4 to 2.3)**	**1.4 (1.2 to 2.0)**	**1.4 (0.9 to 2.1)**	**0.6 (0.6 to 0.7)**	**0.9 (0.9 to 1.0)**	**0.8 (0.8 to 0.9)**	**1.2 (0.8 to 1.4)**	**0.4 (0.3 to 0.4)**	**0.3 (0.2 to 0.4)**	**0.5 (0.2 to 0.5)**	**0.1 (0.1 to 0.1)**
Himachal Pradesh	1.9 (1.3 to 2.2)	2.1 (1.8 to 2.5)	1.6 (1.4 to 2.0)	1.9 (1.3 to 2.4)	2.2 (1.3 to 3.7)	0.7 (0.7 to 0.8)	1.3 (1.0 to 1.6)	0.8 (0.7 to 1.0)	0.8 (0.5 to 1.0)	0.4 (0.3 to 0.5)	0.3 (0.2 to 0.4)	0.3 (0.2 to 0.5)	0.1 (0.1 to 0.1)
Punjab	1.7 (1.5 to 1.8)	2.2 (2.0 to 2.3)	1.7 (1.4 to 2.6)	1.7 (1.5 to 2.3)	2.0 (1.2 to 3.5)	0.5 (0.5 to 0.6)	1.2 (1.1 to 1.4)	0.9 (0.8 to 1.0)	1.1 (0.8 to 1.2)	0.2 (0.2 to 0.3)	0.4 (0.2 to 0.5)	0.3 (0.3 to 0.4)	0.1 (0.1 to 0.2)
Tamil Nadu	1.5 (1.4 to 1.8)	2.1 (2.0 to 2.3)	1.6 (1.3 to 2.4)	1.3 (1.1 to 2.0)	1.0 (0.7 to 1.4)	0.6 (0.6 to 0.7)	0.9 (0.8 to 1.0)	0.9 (0.8 to 0.9)	0.8 (0.6 to 1.1)	0.5 (0.5 to 0.6)	0.3 (0.2 to 0.4)	0.6 (0.2 to 0.7)	0.1 (0.1 to 0.2)
Goa	2.1 (1.8 to 2.3)	2.2 (2.1 to 2.4)	1.8 (1.5 to 2.5)	2.0 (1.3 to 2.4)	2.2 (1.3 to 3.8)	0.8 (0.7 to 0.9)	1.3 (1.1 to 1.6)	1.0 (0.9 to 1.2)	1.1 (0.8 to 1.3)	0.4 (0.4 to 0.5)	0.2 (0.2 to 0.4)	0.4 (0.3 to 0.6)	0.1 (0.1 to 0.1)
Kerala	2.6 (1.6 to 3.2)	2.1 (1.9 to 2.5)	1.8 (1.4 to 2.3)	1.4 (1.1 to 2.0)	1.5 (1.0 to 2.2)	0.6 (0.6 to 0.7)	0.8 (0.7 to 0.9)	0.6 (0.5 to 0.7)	1.9 (0.7 to 2.6)	0.2 (0.2 to 0.2)	0.4 (0.1 to 0.5)	0.4 (0.2 to 0.4)	0.1 (0.1 to 0.1)

Incidence rate per 100,000.
*Union territories. ETL is epidemiological transition level.

Males

Males

States of India	Lip and oral cavity cancer (95% uncertainty interval)	Lung cancer (95% uncertainty interval)	Pharynx cancer other than nasopharynx (95% uncertainty interval)	Stomach cancer (95% uncertainty interval)	Prostate cancer (95% uncertainty interval)	Colon and rectum cancer (95% uncertainty interval)	Larynx cancer (95% uncertainty interval)	Oesophageal cancer (95% uncertainty interval)	Liver cancer (95% uncertainty interval)	Leukaemia (95% uncertainty interval)	Non-Hodgkin lymphoma (95% uncertainty interval)	Bladder cancer (95% uncertainty interval)
**India**	13.5 (12.3 to 14.2)	10.7 (9.9 to 11.1)	9.4 (8.2 to 10.3)	8.5 (8.2 to 9.0)	9.0 (7.1 to 10.8)	7.1 (6.4 to 7.5)	5.4 (5.2 to 5.7)	4.8 (4.6 to 5.0)	4.5 (4.3 to 4.7)	3.5 (3.2 to 4.2)	2.9 (2.2 to 3.1)	3.3 (3.2 to 3.4)
**Low ETL**	**14.1 (12.8 to 14.9)**	**10.0 (9.2 to 10.5)**	**10.9 (9.3 to 12.3)**	**9.6 (9.1 to 10.2)**	**7.9 (6.2 to 9.4)**	**6.9 (6.3 to 7.4)**	**5.8 (5.3 to 6.2)**	**4.8 (4.6 to 5.0)**	**4.7 (4.3 to 5.1)**	**3.3 (3.1 to 4.1)**	**2.8 (2.2 to 3.1)**	**3.0 (2.9 to 3.3)**
Bihar	5.8 (5.0 to 6.6)	8.4 (7.6 to 9.3)	2.5 (1.9 to 3.1)	5.0 (4.6 to 5.4)	7.0 (5.4 to 8.4)	5.7 (5.2 to 6.2)	4.2 (3.6 to 5.3)	5.5 (4.9 to 6.1)	4.6 (3.6 to 5.4)	2.6 (2.2 to 3.2)	2.4 (1.6 to 3.0)	2.5 (2.0 to 3.0)
Jharkhand	14.6 (12.7 to 16.3)	7.2 (6.3 to 8.1)	3.9 (3.1 to 4.7)	9.4 (8.7 to 10.2)	7.8 (6.3 to 9.6)	6.0 (5.2 to 6.6)	5.0 (4.4 to 5.7)	1.2 (1.1 to 1.4)	4.7 (4.1 to 5.3)	3.4 (2.8 to 3.9)	2.5 (2.0 to 2.9)	3.0 (2.8 to 3.3)
Uttar Pradesh	16.4 (14.6 to 17.9)	11.1 (10.1 to 12)	11.8 (9.5 to 14.3)	12.2 (11.3 to 13.2)	7.8 (6.1 to 9.5)	7.5 (6.6 to 8.4)	6.1 (5.4 to 6.8)	2.8 (2.5 to 3.0)	4.8 (4.3 to 5.4)	3.7 (3.2 to 4.2)	2.7 (2.2 to 3.1)	3.1 (2.8 to 3.6)
Rajasthan	12.0 (10.6 to 13.4)	10.0 (9.1 to 11.1)	17.1 (13.7 to 21.1)	12.1 (11.2 to 13.0)	7.8 (6.1 to 9.6)	6.9 (6.1 to 7.7)	5.8 (5.0 to 6.4)	5.5 (5.0 to 6.1)	4.9 (4.3 to 5.5)	2.7 (2.2 to 4.2)	3.3 (2.2 to 3.9)	2.9 (2.6 to 3.2)
Meghalaya	20.8 (18.9 to 22.9)	15.3 (14.0 to 16.6)	28.2 (24.9 to 31.5)	14.2 (13.1 to 15.3)	8.4 (7.0 to 10.7)	7.0 (6.2 to 7.7)	9.8 (9.1 to 10.7)	48.3 (44.6 to 52.4)	5.1 (4.6 to 5.7)	2.7 (2.3 to 4.4)	2.2 (1.9 to 2.8)	2.1 (1.9 to 2.2)
Assam	18.0 (16.0 to 20.2)	12.2 (10.9 to 13.4)	20.6 (17.7 to 23.1)	8.7 (8.0 to 9.4)	9.9 (6.9 to 11.3)	8.1 (7.2 to 8.8)	7.6 (6.9 to 8.3)	19.1 (17.4 to 21.0)	4.3 (3.8 to 4.9)	3.4 (3.0 to 5.0)	2.6 (2.3 to 3.0)	3.3 (3.0 to 3.6)
Chhattisgarh	12.9 (11.6 to 14.1)	9.2 (8.3 to 10.1)	10.6 (8.6 to 12.8)	9.7 (9.0 to 10.5)	8.8 (6.8 to 10.4)	7.5 (6.6 to 8.4)	6.6 (5.6 to 7.3)	2.8 (2.5 to 3.0)	4.9 (4.3 to 5.7)	3.7 (3.3 to 4.7)	2.8 (2.3 to 3.1)	5.5 (5.0 to 6.0)
Madhya Pradesh	21.6 (19.2 to 23.5)	11.2 (10.0 to 12.1)	14.5 (11.3 to 17.7)	7.0 (6.5 to 7.6)	8.3 (6.4 to 9.8)	6.6 (5.8 to 7.3)	6.3 (5.6 to 6.9)	5.3 (4.8 to 5.7)	4.1 (3.4 to 4.9)	3.8 (3.2 to 4.3)	2.9 (2.2 to 3.3)	3.0 (2.8 to 3.3)
Odisha	11.0 (9.6 to 12.1)	7.9 (6.8 to 8.8)	7.6 (6.0 to 9.1)	9.5 (8.8 to 10.2)	8.2 (6.4 to 9.8)	7.4 (6.4 to 8.2)	5.5 (5.0 to 6.2)	2.9 (2.7 to 3.2)	4.8 (4.3 to 5.5)	3.5 (3.1 to 4.2)	3.0 (2.2 to 3.5)	2.8 (2.5 to 3.1)
**Lower-middle ETL**	**21.2 (18.9 to 23.0)**	**12.0 (10.9 to 12.8)**	**9.8 (8.3 to 11.1)**	**6.2 (5.9 to 6.9)**	**9.0 (7.4 to 11.3)**	**6.9 (6.2 to 7.4)**	**5.2 (4.8 to 5.6)**	**6.6 (6.2 to 7.0)**	**3.7 (3.4 to 4.0)**	**3.9 (3.4 to 4.5)**	**3.0 (2.3 to 3.5)**	**2.8 (2.6 to 3.0)**
Arunachal Pradesh	8.4 (7.4 to 9.2)	11.6 (10.3 to 12.7)	10.8 (8.8 to 13.1)	32.5 (30.3 to 34.8)	7.6 (6.4 to 10.0)	6.4 (5.7 to 7.0)	5.6 (5.2 to 6.1)	10.5 (9.6 to 11.4)	23.1 (20.7 to 25.9)	3.1 (2.8 to 4.0)	1.7 (1.4 to 2.7)	2.1 (1.9 to 2.2)
Mizoram	9.2 (8.3 to 10.1)	33.3 (30.3 to 36.0)	13.1 (11.3 to 14.9)	42.3 (39.6 to 45.3)	8.5 (7.3 to 11.5)	11.8 (10.6 to 12.8)	6.4 (5.9 to 6.9)	26.6 (24.6 to 28.6)	9.3 (8.3 to 10.3)	4.5 (3.3 to 5.2)	2.8 (2.2 to 3.3)	2.8 (2.5 to 3.1)
Nagaland	9.9 (8.9 to 10.9)	8.3 (7.6 to 9.1)	14.0 (12.2 to 15.8)	21.8 (20.2 to 23.3)	8.8 (7.6 to 12.8)	8.9 (8.1 to 9.8)	6.0 (5.5 to 6.6)	16.0 (14.6 to 17.6)	6.8 (6.2 to 7.5)	2.7 (2.3 to 4.2)	3.0 (2.4 to 3.4)	1.8 (1.6 to 2.0)
Uttarakhand	14.9 (13.0 to 16.3)	13.3 (11.6 to 14.6)	13.5 (10.5 to 16.6)	11.8 (10.8 to 13.7)	9.9 (7.8 to 12.0)	7.9 (6.6 to 8.8)	6.3 (5.3 to 7.0)	3.5 (3.2 to 3.8)	4.9 (4.3 to 6.2)	3.7 (3.1 to 5.1)	2.9 (2.3 to 3.6)	3.8 (3.5 to 4.3)
Gujarat	24.6 (21.9 to 26.9)	10.8 (9.6 to 11.6)	9.3 (7.7 to 10.7)	3.8 (3.5 to 4.3)	8.9 (7.4 to 11.3)	6.6 (5.9 to 7.2)	5.0 (4.6 to 5.4)	6.5 (5.9 to 7.0)	2.9 (2.6 to 3.3)	4.1 (3.4 to 4.6)	3.1 (2.3 to 3.6)	2.7 (2.4 to 2.9)
Tripura	11.2 (10.2 to 12.1)	17.9 (16.4 to 19.4)	10.8 (9.6 to 12.0)	6.1 (5.7 to 6.5)	7.9 (6.7 to 10.3)	7.1 (6.4 to 7.7)	6.5 (6.0 to 7.0)	7.5 (6.9 to 8.1)	3.2 (2.9 to 3.5)	2.8 (2.4 to 4.2)	1.8 (1.5 to 2.7)	1.8 (1.6 to 1.9)
Sikkim	8.5 (7.2 to 9.5)	9.5 (8.1 to 10.5)	5.7 (4.5 to 6.7)	13.9 (12.8 to 15.7)	8.2 (6.9 to 10.9)	7.0 (6.1 to 7.6)	5.4 (4.9 to 6.1)	9.9 (8.9 to 10.9)	9.6 (8.5 to 10.9)	3.2 (2.9 to 3.9)	1.8 (1.4 to 2.8)	3.5 (3.0 to 3.8)
Manipur	5.3 (4.8 to 5.7)	19.4 (17.7 to 20.9)	3.1 (2.7 to 3.4)	7.2 (6.7 to 7.7)	8.5 (7.4 to 11.4)	7.0 (6.3 to 7.7)	2.9 (2.7 to 3.2)	4.1 (3.8 to 4.4)	3.7 (3.4 to 4.1)	3.3 (2.9 to 4.1)	3.1 (2.3 to 3.7)	3.4 (3.1 to 3.7)
**Higher-middle ETL**	**12.0 (10.8 to 12.7)**	**10.6 (9.7 to 11.2)**	**8.8 (7.5 to 10.0)**	**8.3 (7.9 to 8.8)**	**9.8 (7.6 to 11.7)**	**7.0 (6.3 to 7.4)**	**5.5 (5.2 to 5.8)**	**4.3 (4.1 to 4.4)**	**4.6 (4.4 to 4.9)**	**3.7 (3.2 to 4.3)**	**3.0 (2.3 to 3.3)**	**3.6 (3.4 to 3.7)**
Haryana	17.4 (15.5 to 19.2)	12.7 (11.5 to 13.8)	23.5 (18.8 to 28.7)	11.9 (11.1 to 12.9)	10.7 (8.4 to 13.2)	8.8 (7.9 to 9.8)	8.0 (6.1 to 9.1)	8.8 (7.9 to 9.7)	5.5 (4.8 to 7.4)	3.9 (3.2 to 5.6)	3.1 (2.4 to 4.2)	4.2 (3.6 to 4.9)
Delhi	19.4 (16.1 to 22.5)	19.3 (16.3 to 22.1)	8.6 (6.7 to 10.2)	5.1 (4.5 to 6.0)	16.7 (11 to 19.7)	8.5 (7.3 to 9.4)	10.9 (9.6 to 12.5)	6.1 (5.4 to 6.8)	3.8 (3.3 to 4.3)	6.1 (3.5 to 7.4)	5.3 (2.7 to 6.6)	8.6 (7.4 to 9.6)
Telangana	4.9 (4.3 to 5.4)	6.7 (5.8 to 7.5)	5.4 (4.2 to 6.7)	7.9 (7.3 to 8.7)	8.3 (6.9 to 11.0)	5.2 (4.5 to 5.8)	5.0 (4.3 to 6.6)	1.2 (1.1 to 1.3)	5.1 (4.4 to 5.7)	3.3 (2.7 to 3.8)	2.4 (2.1 to 2.8)	2.6 (2.2 to 3.0)
Andhra Pradesh	5.7 (5.1 to 6.2)	7.6 (6.8 to 8.3)	6.2 (5.0 to 7.5)	8.9 (8.3 to 9.6)	8.4 (6.8 to 10.4)	5.9 (5.3 to 6.4)	5.7 (5.2 to 6.3)	1.5 (1.4 to 1.6)	5.2 (4.5 to 5.9)	3.5 (3.1 to 4.1)	2.5 (2.1 to 2.8)	2.8 (2.5 to 3.1)
Jammu and Kashmir	4.7 (4.2 to 5.1)	20.1 (18.1 to 21.7)	7.4 (5.9 to 8.9)	23.3 (21.4 to 26.3)	9.1 (7.1 to 10.9)	5.9 (5.3 to 6.4)	5.5 (5.0 to 6.0)	4.8 (4.3 to 5.2)	5.1 (4.5 to 5.6)	3.5 (3.0 to 4.0)	2.8 (2.2 to 3.1)	3.3 (3.0 to 3.6)
Karnataka	14.2 (12.7 to 15.7)	9.7 (8.7 to 10.5)	16.9 (13.4 to 20.7)	11.3 (10.5 to 12.2)	9.4 (7.3 to 11.2)	6.9 (6.1 to 7.4)	5.5 (4.9 to 6.2)	4.6 (4.2 to 4.9)	5.2 (4.7 to 5.9)	3.8 (3.3 to 4.4)	3.2 (2.2 to 3.8)	3.6 (3.3 to 4.0)
West Bengal	13.0 (11.7 to 14.1)	14.8 (13.3 to 16.2)	7.7 (6.3 to 9.2)	8.7 (8.1 to 9.4)	9.4 (7.0 to 10.8)	7.0 (6.2 to 7.6)	5.7 (5.3 to 6.4)	3.5 (3.2 to 3.8)	4.3 (3.7 to 4.8)	3.4 (2.9 to 4.0)	2.9 (2.2 to 3.3)	3.8 (3.5 to 4.1)
Maharashtra	12.9 (11.5 to 14.1)	7.9 (7.0 to 8.6)	4.7 (3.9 to 5.3)	4.6 (4.2 to 5.2)	10.3 (8.2 to 12.5)	7.5 (6.6 to 8.1)	4.1 (3.7 to 4.5)	5.4 (5.0 to 5.9)	4.1 (3.7 to 4.5)	3.5 (3.2 to 4.5)	2.8 (2.3 to 3.4)	3.3 (3.0 to 3.6)
UTs* other than Delhi	20.2 (18.0 to 22.3)	9.2 (8.2 to 10.2)	9.0 (7.3 to 10.9)	8.0 (7.3 to 8.9)	10.3 (8.3 to 14.0)	6.3 (5.6 to 7.0)	5.2 (4.5 to 6.2)	2.7 (2.4 to 2.9)	5.0 (4.3 to 5.6)	3.4 (3.0 to 4.1)	5.5 (2.4 to 7.1)	3.8 (3.3 to 4.3)
**High ETL**	**11.9 (10.7 to 12.7)**	**12.3 (11.3 to 13.0)**	**5.9 (5.1 to 6.5)**	**6.8 (6.5 to 7.2)**	**10.4 (8.0 to 12.3)**	**7.9 (7.2 to 8.3)**	**4.5 (4.2 to 4.8)**	**5.3 (5.0 to 5.6)**	**4.1 (3.9 to 4.4)**	**3.7 (3.1 to 4.1)**	**3.1 (2.3 to 3.5)**	**3.7 (3.3 to 3.9)**
Himachal Pradesh	12.3 (10.7 to 13.7)	10.5 (9.3 to 11.5)	14.2 (10.7 to 17.8)	7.8 (7.0 to 9.0)	10.6 (8.1 to 13.0)	7.1 (6.2 to 7.8)	5.1 (4.3 to 6.4)	6.5 (5.9 to 7.2)	5.2 (4.5 to 6.0)	3.3 (2.8 to 3.8)	2.8 (2.4 to 3.2)	4.0 (3.1 to 4.7)
Punjab	6.8 (6.3 to 7.4)	10.0 (9.3 to 10.7)	3.2 (2.7 to 3.4)	3.8 (3.5 to 4.0)	10.2 (7.9 to 12.2)	6.8 (6.2 to 7.4)	3.3 (3.1 to 3.6)	7.3 (6.7 to 8.0)	3.2 (2.9 to 3.6)	4.5 (3.4 to 4.9)	2.7 (2.3 to 3.3)	2.8 (2.6 to 3.0)
Tamil Nadu	11.6 (10.2 to 12.6)	9.4 (8.4 to 10.1)	5.5 (4.6 to 6.3)	7.9 (7.4 to 8.4)	9.4 (7.8 to 12.0)	7.3 (6.5 to 7.9)	3.7 (3.4 to 4.1)	4.8 (4.4 to 5.2)	3.3 (3.0 to 3.6)	3.2 (2.8 to 4.3)	2.5 (2.2 to 3.1)	2.6 (2.3 to 2.8)
Goa	8.8 (7.8 to 9.7)	5.8 (5.2 to 6.3)	7.8 (6.2 to 9.3)	7.8 (7.2 to 8.7)	12.1 (9.7 to 15.8)	5.7 (5.2 to 6.3)	5.5 (4.7 to 6.5)	2.2 (2.1 to 2.4)	5.6 (4.7 to 6.5)	3.3 (2.9 to 4.0)	2.9 (2.5 to 3.6)	4.2 (3.6 to 4.6)
Kerala	16.4 (14.5 to 17.9)	19.9 (17.9 to 21.5)	7.0 (5.9 to 7.9)	6.8 (6.3 to 7.3)	12.5 (8.0 to 14.5)	10.1 (9.0 to 10.9)	6.7 (6.1 to 7.2)	4.6 (4.3 to 5.0)	6.1 (5.5 to 6.8)	4.2 (2.4 to 4.9)	4.5 (2.2 to 5.6)	6.3 (5.6 to 6.8)

Incidence rate per 100,000.
*Union territories. ETL is epidemiological transition level.

Males

States of India	Brain and nervous system cancer (95% uncertainty interval)	Pancreatic cancer (95% uncertainty interval)	Non-melanoma skin cancer (95% uncertainty interval)	Gallbladder and biliary tract cancer (95% uncertainty interval)	Kidney cancer (95% uncertainty interval)	Nasopharynx cancer (95% uncertainty interval)	Thyroid cancer (95% uncertainty interval)	Multiple myeloma (95% uncertainty interval)	Hodgkin's lymphoma (95% uncertainty interval)	Testicular cancer (95% uncertainty interval)	Breast cancer (95% uncertainty interval)	Mesothelioma (95% uncertainty interval)	Malignant skin melanoma (95% uncertainty interval)
**India**	2.4 (2.1 to 3.0)	2.9 (2.8 to 2.9)	2.6 (1.8 to 3.8)	1.9 (1.3 to 2.4)	1.7 (1.6 to 1.8)	1.3 (1.2 to 1.4)	1.0 (0.9 to 1.2)	1.0 (0.7 to 1.1)	0.7 (0.6 to 1.1)	0.5 (0.4 to 0.5)	0.5 (0.4 to 0.5)	0.4 (0.4 to 0.6)	0.3 (0.3 to 0.4)
**Low ETL**	**2.2 (1.9 to 2.8)**	**2.8 (2.7 to 2.9)**	**3.0 (2.0 to 4.6)**	**2.2 (1.4 to 2.5)**	**1.4 (1.3 to 1.6)**	**1.5 (1.3 to 1.7)**	**0.8 (0.7 to 0.9)**	**0.8 (0.6 to 1.0)**	**0.8 (0.6 to 1.2)**	**0.3 (0.3 to 0.4)**	**0.4 (0.3 to 0.5)**	**0.4 (0.3 to 0.6)**	**0.3 (0.2 to 0.3)**
Bihar	2.0 (1.2 to 2.8)	2.5 (2.3 to 2.7)	2.9 (1.9 to 4.6)	1.9 (1.3 to 2.5)	1.1 (0.8 to 1.4)	1.4 (1.0 to 1.8)	0.7 (0.5 to 0.8)	0.7 (0.4 to 0.9)	0.6 (0.5 to 1.0)	0.1 (0.1 to 0.1)	0.3 (0.2 to 0.4)	0.4 (0.2 to 0.6)	0.2 (0.1 to 0.3)
Jharkhand	2.2 (1.7 to 2.8)	2.6 (2.4 to 2.8)	3.0 (1.9 to 4.9)	1.9 (1.3 to 2.4)	1.4 (1.2 to 1.6)	1.5 (1.2 to 1.8)	0.8 (0.7 to 1.0)	0.7 (0.6 to 0.9)	0.8 (0.6 to 1.2)	0.3 (0.3 to 0.4)	0.4 (0.3 to 0.5)	0.4 (0.3 to 0.5)	0.3 (0.2 to 0.3)
Uttar Pradesh	2.3 (1.9 to 2.8)	2.8 (2.6 to 3.0)	3.1 (2.1 to 4.8)	2.1 (1.5 to 2.6)	1.4 (1.3 to 1.7)	1.6 (1.4 to 1.9)	0.8 (0.7 to 1.0)	0.8 (0.7 to 1.0)	0.8 (0.7 to 1.3)	0.4 (0.3 to 0.4)	0.5 (0.3 to 0.5)	0.5 (0.3 to 0.6)	0.3 (0.2 to 0.4)
Rajasthan	2.2 (1.9 to 2.8)	2.8 (2.6 to 3.0)	3.1 (2.0 to 4.7)	2.1 (1.4 to 2.6)	1.5 (1.4 to 1.7)	1.6 (1.3 to 1.9)	0.9 (0.8 to 1.0)	0.8 (0.7 to 1.0)	0.7 (0.6 to 1.1)	0.1 (0.0 to 0.1)	0.2 (0.1 to 0.5)	0.4 (0.3 to 0.6)	0.3 (0.2 to 0.4)
Meghalaya	1.9 (1.4 to 3.1)	2.8 (2.6 to 3.0)	3.1 (2.1 to 4.9)	1.7 (1.4 to 2.6)	1.5 (1.4 to 1.8)	3.0 (2.6 to 3.3)	0.9 (0.8 to 1.1)	0.7 (0.6 to 1.1)	0.3 (0.2 to 1.1)	0.2 (0.2 to 0.3)	0.4 (0.4 to 0.5)	0.4 (0.4 to 0.6)	0.3 (0.3 to 0.4)
Assam	2.3 (1.9 to 3.2)	3.4 (3.2 to 3.7)	3.0 (2.1 to 4.4)	3.9 (1.4 to 5.3)	1.9 (1.6 to 2.1)	1.2 (1.0 to 1.4)	0.7 (0.6 to 1.0)	0.9 (0.7 to 1.2)	0.5 (0.3 to 1.4)	0.5 (0.5 to 0.6)	0.6 (0.4 to 0.7)	0.5 (0.3 to 0.7)	0.3 (0.3 to 0.4)
Chhattisgarh	2.4 (2.0 to 3.1)	3.7 (3.5 to 4.0)	3.2 (2.1 to 5.0)	2.1 (1.4 to 2.7)	1.6 (1.4 to 1.9)	1.8 (1.5 to 2.0)	1.0 (0.8 to 1.2)	1.0 (0.7 to 1.1)	0.8 (0.7 to 1.3)	0.5 (0.4 to 0.5)	0.5 (0.4 to 0.6)	0.4 (0.3 to 0.6)	0.3 (0.3 to 0.4)
Madhya Pradesh	2.3 (2.0 to 2.8)	2.7 (2.5 to 2.9)	2.7 (1.9 to 3.9)	2.3 (1.4 to 2.7)	1.4 (1.2 to 1.6)	1.3 (1.1 to 1.6)	0.8 (0.7 to 1.0)	0.9 (0.6 to 1.1)	0.8 (0.6 to 1.2)	0.5 (0.5 to 0.6)	0.5 (0.3 to 0.6)	0.4 (0.3 to 0.6)	0.3 (0.3 to 0.4)
Odisha	2.3 (2.0 to 2.8)	2.8 (2.6 to 3.1)	3.1 (2.1 to 4.7)	2.0 (1.4 to 2.5)	1.5 (1.3 to 1.6)	1.6 (1.4 to 1.9)	0.9 (0.8 to 1.0)	0.9 (0.7 to 1.0)	0.8 (0.6 to 1.2)	0.4 (0.3 to 0.5)	0.4 (0.3 to 0.5)	0.4 (0.3 to 0.6)	0.3 (0.2 to 0.4)
**Lower-middle ETL**	**2.2 (1.8 to 3.3)**	**3.2 (3.0 to 3.3)**	**2.2 (1.7 to 2.9)**	**1.5 (1.3 to 2.4)**	**1.7 (1.5 to 2.0)**	**1.6 (1.5 to 1.7)**	**0.8 (0.7 to 1.2)**	**0.9 (0.7 to 1.2)**	**0.7 (0.6 to 1.0)**	**0.6 (0.6 to 0.7)**	**0.6 (0.4 to 0.6)**	**0.4 (0.4 to 0.5)**	**0.4 (0.3 to 0.5)**
Arunachal Pradesh	1.8 (1.3 to 2.9)	2.8 (2.6 to 3.0)	3.3 (2.1 to 5.1)	2.2 (1.3 to 2.7)	1.5 (1.3 to 1.7)	4.4 (4.0 to 5.0)	1.7 (0.9 to 1.9)	0.8 (0.6 to 1.0)	0.6 (0.5 to 0.9)	0.4 (0.4 to 0.5)	0.5 (0.4 to 0.6)	0.4 (0.3 to 0.5)	0.3 (0.3 to 0.4)
Mizoram	2.0 (1.6 to 3.3)	3.1 (2.9 to 3.3)	2.3 (1.7 to 3.3)	2.1 (1.3 to 2.6)	1.4 (1.2 to 1.9)	6.2 (5.5 to 6.9)	1.8 (1.1 to 2.1)	1.2 (0.6 to 1.4)	0.7 (0.5 to 0.9)	0.3 (0.3 to 0.3)	0.8 (0.4 to 0.9)	0.4 (0.3 to 0.5)	0.7 (0.3 to 0.9)
Nagaland	1.7 (1.2 to 3.4)	2.4 (2.2 to 2.6)	3.2 (2.2 to 4.9)	1.8 (1.2 to 2.3)	1.6 (1.4 to 1.9)	14.9 (13.2 to 16.9)	1.5 (1.3 to 1.6)	0.8 (0.6 to 1.2)	0.6 (0.5 to 1.0)	0.5 (0.4 to 0.6)	0.6 (0.4 to 0.7)	0.4 (0.3 to 0.5)	0.5 (0.3 to 0.6)
Uttarakhand	2.6 (2.1 to 4.0)	3.2 (3.0 to 3.5)	3.4 (2.3 to 5.3)	2.1 (1.4 to 2.5)	1.8 (1.4 to 2.4)	1.6 (1.4 to 1.9)	1.2 (1.1 to 1.5)	1.0 (0.7 to 1.3)	0.7 (0.6 to 1.1)	0.5 (0.5 to 0.6)	0.6 (0.4 to 0.8)	0.5 (0.4 to 0.6)	0.4 (0.3 to 0.6)
Gujarat	2.2 (1.8 to 3.3)	3.3 (3.1 to 3.5)	1.9 (1.5 to 2.3)	1.3 (1.0 to 2.4)	1.7 (1.5 to 2.0)	1.0 (0.8 to 1.1)	0.7 (0.6 to 1.2)	0.9 (0.7 to 1.2)	0.7 (0.6 to 1.0)	0.7 (0.6 to 0.8)	0.6 (0.4 to 0.7)	0.4 (0.4 to 0.5)	0.3 (0.3 to 0.5)
Tripura	1.9 (1.4 to 2.9)	2.6 (2.4 to 2.8)	3.1 (2.0 to 5.0)	2.4 (1.4 to 2.9)	1.5 (1.4 to 1.7)	0.9 (0.8 to 1.0)	0.6 (0.5 to 1.0)	0.6 (0.5 to 1.0)	0.3 (0.1 to 1.0)	0.4 (0.3 to 0.4)	0.4 (0.4 to 0.5)	0.4 (0.3 to 0.6)	0.3 (0.3 to 0.4)
Sikkim	3.0 (2.1 to 3.7)	2.6 (2.4 to 2.8)	3.2 (2.1 to 5.0)	2.3 (1.2 to 3.0)	1.5 (1.3 to 2.0)	4.4 (3.6 to 5.1)	2.2 (1.2 to 2.6)	0.7 (0.5 to 1.1)	0.5 (0.4 to 0.8)	0.2 (0.2 to 0.2)	0.6 (0.4 to 0.7)	0.4 (0.4 to 0.5)	1.3 (0.3 to 1.8)
Manipur	1.8 (1.3 to 3.1)	2.7 (2.5 to 2.9)	2.3 (1.7 to 3.1)	1.9 (1.3 to 2.4)	1.5 (1.3 to 1.9)	4.9 (4.4 to 5.4)	1.2 (1.0 to 1.3)	0.7 (0.6 to 1.1)	0.5 (0.3 to 0.9)	0.2 (0.1 to 0.2)	0.3 (0.3 to 0.6)	0.4 (0.3 to 0.5)	0.3 (0.3 to 0.4)
**Higher-middle ETL**	**2.6 (2.1 to 3.3)**	**2.9 (2.8 to 3.0)**	**2.4 (1.7 to 3.3)**	**1.9 (1.3 to 2.4)**	**1.9 (1.8 to 2.0)**	**1.2 (1.1 to 1.4)**	**1.1 (1.0 to 1.3)**	**1.0 (0.7 to 1.2)**	**0.7 (0.6 to 1.0)**	**0.6 (0.5 to 0.6)**	**0.5 (0.4 to 0.6)**	**0.5 (0.4 to 0.5)**	**0.4 (0.3 to 0.5)**
Haryana	2.7 (2.1 to 4.6)	3.1 (2.9 to 3.3)	3.3 (2.2 to 5.2)	2.1 (1.4 to 2.7)	2.2 (1.7 to 3.0)	1.8 (1.5 to 2.4)	1.5 (1.2 to 1.9)	1.2 (0.8 to 1.7)	0.8 (0.7 to 1.3)	0.7 (0.5 to 0.8)	0.7 (0.5 to 0.8)	0.5 (0.4 to 0.7)	0.5 (0.4 to 0.7)
Delhi	4.0 (2.2 to 5.5)	2.9 (2.7 to 3.2)	1.2 (1.1 to 1.4)	3.8 (1.1 to 5.5)	3.5 (2.6 to 3.9)	0.8 (0.6 to 0.9)	2.1 (1.7 to 2.6)	2.4 (0.7 to 3.2)	1.6 (0.6 to 2.1)	0.9 (0.8 to 1.1)	1.2 (0.5 to 1.6)	0.5 (0.3 to 0.7)	0.7 (0.5 to 1.0)
Telangana	2.3 (1.8 to 2.8)	2.7 (2.5 to 2.9)	3.2 (2.1 to 5.0)	1.9 (1.2 to 2.4)	1.6 (1.4 to 1.9)	1.5 (1.1 to 2.1)	1.1 (0.8 to 1.3)	0.7 (0.6 to 1.0)	0.6 (0.5 to 1.1)	0.4 (0.3 to 0.5)	0.4 (0.3 to 0.5)	0.4 (0.3 to 0.5)	0.3 (0.3 to 0.4)
Andhra Pradesh	2.3 (2.0 to 2.9)	3.0 (2.8 to 3.2)	3.1 (2.0 to 5.0)	2.0 (1.4 to 2.5)	1.7 (1.6 to 1.9)	1.6 (1.4 to 2.0)	1.0 (0.9 to 1.2)	0.8 (0.7 to 1.1)	0.7 (0.5 to 1.0)	0.4 (0.4 to 0.5)	0.5 (0.4 to 0.7)	0.4 (0.3 to 0.5)	0.4 (0.3 to 0.4)
Jammu and Kashmir	2.4 (2.1 to 3.1)	2.9 (2.7 to 3.1)	3.1 (2.0 to 4.8)	1.9 (1.3 to 2.3)	1.7 (1.5 to 1.8)	1.5 (1.2 to 1.8)	1.0 (0.9 to 1.2)	0.9 (0.6 to 1.1)	0.6 (0.5 to 1.0)	0.4 (0.4 to 0.5)	0.5 (0.4 to 0.6)	0.5 (0.4 to 0.6)	0.3 (0.3 to 0.4)
Karnataka	2.6 (2.1 to 3.2)	2.7 (2.6 to 2.9)	2.4 (1.7 to 3.5)	1.8 (1.3 to 2.4)	1.7 (1.6 to 1.9)	1.6 (1.4 to 1.8)	1.0 (0.9 to 1.2)	0.9 (0.7 to 1.2)	0.7 (0.5 to 1.1)	0.5 (0.4 to 0.5)	0.5 (0.4 to 0.6)	0.4 (0.3 to 0.6)	0.3 (0.3 to 0.5)
West Bengal	2.3 (1.9 to 2.9)	2.9 (2.7 to 3.1)	2.4 (1.7 to 3.5)	2.3 (1.3 to 2.7)	1.8 (1.5 to 1.9)	1.2 (1.0 to 1.6)	0.9 (0.8 to 1.1)	1.0 (0.6 to 1.2)	0.7 (0.5 to 0.9)	0.5 (0.4 to 0.5)	0.5 (0.3 to 0.6)	0.5 (0.3 to 0.6)	0.4 (0.3 to 0.4)
Maharashtra	2.7 (2.1 to 3.9)	2.9 (2.7 to 3.1)	1.6 (1.4 to 1.9)	1.4 (1.2 to 2.2)	2.0 (1.7 to 2.3)	0.6 (0.5 to 0.6)	1.1 (1.0 to 1.5)	0.9 (0.7 to 1.3)	0.6 (0.5 to 0.9)	0.7 (0.6 to 0.8)	0.5 (0.4 to 0.6)	0.5 (0.4 to 0.5)	0.4 (0.3 to 0.6)
UTs* other than Delhi	2.6 (2.1 to 3.8)	4.2 (3.9 to 4.6)	3.3 (2.1 to 5.0)	1.8 (1.2 to 2.3)	1.9 (1.6 to 2.3)	1.4 (1.1 to 1.8)	1.5 (1.3 to 1.8)	2.6 (0.6 to 3.7)	0.6 (0.5 to 0.9)	0.6 (0.5 to 0.7)	0.5 (0.4 to 0.6)	0.4 (0.3 to 0.6)	0.6 (0.4 to 0.8)
**High ETL**	**2.5 (2.1 to 3.4)**	**3.0 (2.9 to 3.1)**	**2.1 (1.6 to 3.0)**	**1.4 (1.2 to 2.3)**	**1.9 (1.7 to 2.1)**	**0.8 (0.7 to 0.8)**	**1.4 (1.3 to 1.5)**	**1.4 (0.7 to 1.7)**	**0.7 (0.5 to 0.8)**	**0.5 (0.5 to 0.5)**	**0.4 (0.4 to 0.6)**	**0.4 (0.4 to 0.5)**	**0.5 (0.3 to 0.5)**
Himachal Pradesh	2.5 (2.1 to 3.3)	3.1 (2.9 to 3.4)	3.1 (2.0 to 4.8)	1.8 (1.1 to 2.4)	1.9 (1.6 to 2.2)	1.3 (1.1 to 1.7)	1.3 (1.0 to 1.5)	0.9 (0.6 to 1.1)	0.6 (0.5 to 0.9)	0.5 (0.4 to 0.6)	0.5 (0.4 to 0.6)	0.4 (0.4 to 0.5)	0.4 (0.3 to 0.6)
Punjab	2.5 (2.1 to 3.7)	2.7 (2.6 to 2.9)	2.9 (1.8 to 4.7)	2.0 (1.3 to 2.4)	1.9 (1.7 to 2.3)	1.2 (1.0 to 1.4)	0.6 (0.5 to 1.5)	1.2 (0.7 to 1.4)	0.7 (0.5 to 0.9)	0.6 (0.5 to 0.6)	0.5 (0.4 to 0.7)	0.4 (0.4 to 0.6)	0.4 (0.3 to 0.6)
Tamil Nadu	2.3 (1.9 to 3.6)	2.8 (2.6 to 2.9)	1.6 (1.3 to 2.1)	1.2 (0.9 to 2.3)	1.7 (1.5 to 1.9)	0.6 (0.5 to 0.6)	0.9 (0.8 to 1.4)	0.8 (0.7 to 1.3)	0.6 (0.5 to 0.9)	0.4 (0.4 to 0.5)	0.4 (0.3 to 0.6)	0.5 (0.4 to 0.5)	0.5 (0.3 to 0.6)
Goa	2.6 (2.1 to 4.0)	2.9 (2.7 to 3.1)	3.2 (2.1 to 4.9)	1.7 (1.0 to 2.2)	2.3 (1.8 to 2.7)	1.4 (1.0 to 1.8)	2.0 (1.6 to 2.4)	0.9 (0.6 to 1.5)	0.6 (0.4 to 0.8)	0.6 (0.5 to 0.7)	0.6 (0.4 to 0.7)	0.4 (0.3 to 0.5)	0.6 (0.4 to 0.9)
Kerala	2.9 (2.1 to 3.6)	3.7 (3.4 to 3.9)	2.3 (1.7 to 3.1)	1.4 (1.0 to 2.2)	2.4 (2.1 to 2.7)	0.7 (0.6 to 0.7)	2.8 (1.5 to 3.2)	2.7 (0.6 to 3.8)	0.8 (0.3 to 1.0)	0.6 (0.5 to 0.7)	0.5 (0.4 to 0.6)	0.4 (0.3 to 0.5)	0.5 (0.3 to 0.6)

Incidence rate per 100,000.
*Union territories. ETL is epidemiological transition level.

11. Crude incidence rate of the ten leading incident cancers in the states of India by sex, 2016

Females

Females										
	Breast cancer (95% uncertainty interval)	Cervical cancer (95% uncertainty interval)	Lip and oral cavity cancer (95% uncertainty interval)	Stomach cancer (95% uncertainty interval)	Colon and rectum cancer (95% uncertainty interval)	Ovarian cancer (95% uncertainty interval)	Pharynx cancer other than nasopharynx (95% uncertainty interval)	Lung cancer (95% uncertainty interval)	Gallbladder and biliary tract cancer (95% uncertainty interval)	Thyroid cancer (95% uncertainty interval)
**India**	18.3 (16.5 to 20.1)	12.2 (10.8 to 15.1)	7.2 (6.9 to 7.7)	5.6 (5.3 to 5.9)	5.0 (4.6 to 5.3)	4.0 (3.7 to 4.3)	3.1 (2.9 to 3.2)	2.9 (2.7 to 3.6)	2.6 (2.3 to 2.8)	2.5 (2.3 to 2.6)
**Low ETL**	14.1 (12.6 to 15.7)	11.3 (9.1 to 15.6)	7.0 (6.6 to 7.6)	6.3 (5.9 to 6.7)	4.8 (4.4 to 5.2)	3.3 (3.0 to 3.6)	3.3 (3.1 to 3.6)	2.7 (2.4 to 3.7)	3.0 (2.3 to 3.3)	1.7 (1.5 to 2.0)
Bihar	10.8 (9.6 to 12.2)	10.7 (8.1 to 14.6)	2.6 (2.4 to 2.9)	2.1 (2.0 to 2.3)	3.7 (3.3 to 4.0)	2.6 (2.3 to 2.9)	0.9 (0.7 to 1.0)	3.5 (3.2 to 3.9)	2.5 (2.0 to 3.0)	1.2 (1.0 to 1.5)
Jharkhand	12.5 (10.3 to 17.0)	12.1 (9.1 to 15.7)	3.4 (3.1 to 3.7)	5.8 (5.3 to 6.3)	4.1 (3.5 to 4.6)	3.0 (2.7 to 3.6)	2.3 (2.0 to 2.6)	2.0 (1.8 to 3.5)	2.7 (2.1 to 3.2)	2.0 (1.6 to 2.3)
Uttar Pradesh	15.2 (12.5 to 17.8)	11.6 (8.7 to 16.4)	7.8 (7.0 to 9.1)	9.8 (9.0 to 10.6)	5.7 (5.0 to 6.5)	3.1 (2.7 to 3.7)	3.9 (3.4 to 4.4)	2.5 (2.2 to 4.1)	2.8 (2.2 to 3.3)	1.7 (1.3 to 2.1)
Rajasthan	13.7 (12.2 to 15.4)	10.8 (8.8 to 13.4)	4.6 (4.2 to 5.2)	5.9 (5.4 to 6.4)	4.0 (3.6 to 4.4)	3.0 (2.6 to 3.3)	5.5 (4.8 to 6.2)	2.2 (2.0 to 3.8)	2.5 (2.1 to 2.8)	1.7 (1.6 to 1.9)
Meghalaya	8.4 (6.7 to 13.7)	5.6 (4.8 to 9.9)	6.6 (6.1 to 7.2)	3.5 (3.2 to 3.7)	2.6 (2.3 to 2.8)	1.6 (1.5 to 2.7)	1.9 (1.8 to 2.2)	2.2 (2.0 to 2.4)	2.5 (1.5 to 2.8)	0.9 (0.7 to 1.9)
Assam	14.4 (11.9 to 20.2)	8.0 (6.5 to 16.6)	5.8 (5.3 to 6.3)	2.8 (2.6 to 3.0)	4.4 (3.8 to 5.1)	4.8 (3.2 to 5.3)	2.9 (2.6 to 3.4)	2.9 (2.6 to 3.2)	6.5 (2.4 to 7.6)	1.7 (1.4 to 2.3)
Chhattisgarh	14.6 (12.0 to 20.5)	15.8 (12.0 to 18.7)	7.3 (6.6 to 8.1)	4.3 (4.0 to 4.6)	4.5 (3.9 to 5.0)	3.7 (3.3 to 4.1)	5.0 (4.4 to 5.6)	2.3 (2.0 to 3.9)	3.0 (2.5 to 3.4)	2.6 (2.1 to 3.0)
Madhya Pradesh	16.3 (14.0 to 18.3)	13.3 (10.3 to 15.7)	13.1 (12.0 to 14.3)	5.1 (4.7 to 5.5)	4.6 (4.0 to 5.1)	4.3 (3.2 to 4.7)	2.8 (2.4 to 3.3)	2.6 (2.4 to 3.2)	3.1 (2.3 to 3.4)	1.7 (1.6 to 2.0)
Odisha	15.1 (12.3 to 21.7)	8.9 (7.3 to 18.6)	10.8 (9.8 to 11.9)	7.5 (6.9 to 8.2)	6.7 (5.7 to 7.6)	3.9 (3.4 to 4.6)	3.8 (3.3 to 4.3)	2.7 (2.4 to 4.1)	3.2 (2.5 to 3.9)	2.4 (1.9 to 2.8)
**Lower-middle ETL**	17.9 (15.7 to 21.4)	10.2 (9.1 to 14.3)	5.9 (5.5 to 6.5)	3.0 (2.8 to 3.2)	4.4 (4.0 to 4.8)	3.5 (3.3 to 3.9)	1.7 (1.5 to 1.8)	3.2 (2.9 to 3.4)	1.6 (1.5 to 2.1)	2.1 (1.9 to 3.0)
Arunachal Pradesh	9.3 (7.6 to 12.9)	9.2 (7.4 to 10.5)	3.2 (2.9 to 3.5)	11.0 (10.2 to 11.7)	2.4 (2.1 to 2.7)	6.1 (2.7 to 6.8)	0.6 (0.6 to 0.7)	3.1 (2.0 to 3.4)	2.3 (1.5 to 2.6)	5.8 (1.9 to 6.6)
Mizoram	15.6 (13.4 to 17.9)	11.2 (8.1 to 12.8)	2.5 (2.2 to 2.7)	14.5 (13.6 to 15.5)	5.1 (4.6 to 5.6)	2.7 (2.5 to 3.6)	1.9 (1.7 to 2.0)	18.9 (10.7 to 20.8)	2.7 (1.7 to 3.0)	4.3 (2.9 to 4.9)
Nagaland	9.2 (7.6 to 15.9)	7.0 (5.9 to 8.7)	1.6 (1.5 to 1.7)	7.7 (7.3 to 8.3)	2.8 (2.5 to 3.1)	2.3 (2.1 to 2.8)	0.8 (0.7 to 0.9)	2.2 (2.0 to 2.4)	1.3 (1.2 to 1.5)	6.7 (3.0 to 7.7)
Uttarakhand	16.6 (12.7 to 23.5)	12.0 (10.1 to 15.2)	5.2 (4.8 to 5.7)	8.9 (8.2 to 10.0)	5.3 (4.4 to 6.3)	3.8 (3.4 to 4.3)	4.8 (4.0 to 5.8)	4.6 (3.5 to 5.1)	2.7 (2.3 to 3.1)	3.6 (3.2 to 4.0)
Gujarat	19.6 (17.3 to 22.5)	10.3 (8.9 to 14.8)	6.7 (6.1 to 7.5)	1.4 (1.3 to 1.6)	4.5 (4.0 to 4.9)	3.6 (3.3 to 4.0)	1.3 (1.2 to 1.4)	2.5 (2.2 to 2.9)	1.1 (1.0 to 2.2)	1.4 (1.2 to 3.1)
Tripura	11.1 (9.3 to 17.9)	8.8 (7.6 to 13.6)	3.9 (3.5 to 4.2)	2.5 (2.3 to 2.6)	4.0 (3.6 to 4.4)	2.8 (2.5 to 3.6)	1.3 (1.2 to 1.4)	2.6 (2.4 to 3.0)	4.3 (2.2 to 4.8)	1.1 (1.0 to 2.1)
Sikkim	9.2 (7.3 to 14.8)	7.1 (6.1 to 9.0)	2.9 (2.6 to 3.2)	6.2 (5.7 to 6.7)	2.6 (2.2 to 3.1)	3.8 (3.0 to 4.1)	1.6 (1.4 to 2.0)	5.3 (3.9 to 6.0)	3.6 (1.5 to 4.3)	7.3 (2.6 to 9.2)
Manipur	11.2 (9.0 to 18.9)	7.2 (6.1 to 12.6)	2.0 (1.8 to 2.2)	2.7 (2.5 to 2.8)	3.4 (3.0 to 3.8)	2.9 (2.6 to 3.6)	0.5 (0.4 to 0.5)	8.3 (6.5 to 9.2)	3.2 (1.9 to 3.5)	4.8 (2.7 to 5.4)
**Higher-middle ETL**	20.5 (18.2 to 23.8)	13.1 (10.8 to 15.2)	7.7 (7.4 to 8.3)	6.1 (5.8 to 6.5)	4.9 (4.5 to 5.3)	4.7 (4.3 to 4.9)	3.5 (3.3 to 3.8)	3.1 (2.8 to 3.6)	2.7 (2.4 to 3.0)	2.9 (2.7 to 3.2)
Haryana	23.5 (20.1 to 26.7)	9.2 (7.9 to 14.9)	8.6 (7.8 to 9.7)	10.9 (10.1 to 11.7)	5.3 (4.7 to 5.9)	3.8 (3.4 to 4.6)	9.3 (8.0 to 10.7)	3.0 (2.6 to 4.4)	2.8 (2.4 to 3.2)	4.1 (3.6 to 4.7)
Delhi	36.0 (22.9 to 43.3)	12.1 (8.4 to 14.4)	4.5 (4.0 to 5.1)	1.5 (1.4 to 1.7)	4.2 (3.5 to 4.7)	6.5 (4.3 to 7.4)	1.2 (1.1 to 1.4)	3.1 (2.6 to 3.5)	6.4 (1.8 to 7.6)	5.3 (4.3 to 7.0)
Telangana	13.5 (10.7 to 21.8)	11.1 (9.7 to 14.9)	7.4 (6.7 to 8.2)	9.3 (8.6 to 10.2)	3.8 (3.3 to 4.4)	3.7 (3.3 to 4.2)	3.9 (3.3 to 4.5)	2.0 (1.7 to 3.5)	2.8 (2.4 to 3.3)	2.9 (2.5 to 3.6)
Andhra Pradesh	13.9 (11.5 to 22.1)	11.9 (10.4 to 15.9)	7.6 (6.9 to 8.3)	10.0 (9.3 to 10.9)	4.0 (3.5 to 4.5)	3.8 (3.4 to 4.2)	4.0 (3.6 to 4.7)	2.1 (1.8 to 4.1)	2.9 (2.3 to 3.3)	2.7 (2.3 to 3.1)
Jammu and Kashmir	11.8 (9.5 to 18.7)	6.5 (5.3 to 13.1)	2.2 (2.0 to 2.4)	12.0 (11.1 to 13.1)	4.0 (3.5 to 4.5)	3.6 (3.3 to 3.9)	3.8 (3.3 to 4.4)	7.3 (3.1 to 8.2)	2.4 (2.0 to 2.6)	2.5 (2.2 to 2.8)
Karnataka	21.0 (18.5 to 24.3)	20.5 (11.7 to 23.8)	13.0 (11.6 to 15.2)	8.3 (7.6 to 9.0)	4.8 (4.3 to 5.4)	4.7 (4.2 to 5.5)	8.2 (6.9 to 9.6)	2.9 (2.6 to 3.3)	2.7 (2.1 to 3.2)	2.8 (2.4 to 3.2)
West Bengal	18.8 (16.3 to 22.0)	10.9 (9.6 to 15.5)	6.8 (6.2 to 7.4)	4.9 (4.6 to 5.4)	5.7 (5.1 to 6.4)	5.2 (4.1 to 5.6)	1.8 (1.6 to 2.1)	4.1 (3.7 to 4.4)	3.8 (2.3 to 4.2)	2.1 (1.9 to 2.6)
Maharashtra	24.6 (21.2 to 28.0)	13.6 (10.3 to 15.5)	6.5 (5.9 to 7.3)	2.0 (1.9 to 2.2)	5.1 (4.6 to 5.7)	5.0 (4.5 to 5.4)	1.1 (1.0 to 1.2)	2.7 (2.4 to 3.2)	1.3 (1.1 to 2.2)	3.1 (2.8 to 4.0)
UTs* other than Delhi	14.6 (11.9 to 23.8)	11.2 (8.1 to 13.2)	11.5 (10.5 to 12.5)	6.6 (6.1 to 7.2)	5.2 (4.4 to 6.0)	3.0 (2.4 to 4.1)	3.5 (3.0 to 4.1)	2.8 (2.5 to 3.1)	2.1 (1.7 to 2.6)	4.4 (3.6 to 5.5)
**High ETL**	28.7 (23.6 to 31.9)	14.2 (10.6 to 15.9)	7.0 (6.5 to 7.6)	3.0 (2.8 to 3.2)	5.9 (5.4 to 6.3)	5.6 (5.0 to 6.0)	1.5 (1.4 to 1.6)	3.1 (2.9 to 3.5)	1.7 (1.5 to 2.4)	4.2 (3.9 to 4.6)
Himachal Pradesh	13.7 (10.6 to 26.0)	14.0 (7.8 to 17.0)	4.6 (4.2 to 5.1)	6.2 (5.8 to 6.9)	3.7 (3.1 to 4.2)	4.4 (3.3 to 5.0)	6.4 (5.4 to 7.6)	2.2 (1.9 to 3.9)	2.5 (1.8 to 3.4)	3.5 (2.7 to 5.0)
Punjab	29.3 (22.7 to 32.8)	13.2 (10.6 to 15.5)	2.0 (1.8 to 2.2)	1.5 (1.4 to 1.6)	6.1 (5.6 to 6.8)	6.0 (4.7 to 6.5)	0.4 (0.4 to 0.5)	3.0 (2.8 to 3.8)	3.9 (2.6 to 4.2)	2.1 (1.8 to 3.8)
Tamil Nadu	24.3 (20.6 to 28.3)	16.0 (11.2 to 18.5)	6.8 (6.3 to 7.4)	3.3 (3.1 to 3.6)	5.2 (4.6 to 5.7)	4.8 (4.4 to 5.2)	1.5 (1.4 to 1.7)	2.6 (2.3 to 3.0)	0.9 (0.7 to 2.3)	2.5 (2.2 to 3.7)
Goa	28.8 (22.2 to 33.8)	8.9 (7.5 to 11.8)	9.9 (9.1 to 11.0)	7.6 (7.1 to 8.2)	4.9 (4.4 to 5.5)	5.1 (4.6 to 5.7)	5.3 (4.6 to 6.0)	2.5 (2.2 to 3.2)	2.7 (2.0 to 3.4)	6.2 (4.7 to 8.6)
Kerala	39.8 (21.8 to 46.8)	11.7 (8.4 to 13.4)	11.2 (10.1 to 13.0)	2.7 (2.5 to 2.9)	7.4 (6.6 to 8.3)	7.2 (5.5 to 8.5)	1.1 (1.0 to 1.3)	4.4 (3.9 to 5.0)	1.4 (1.2 to 2.2)	9.1 (4.2 to 10.5)

Ratio of the state incidence to median incidence of all states

>2.74 2.25-2.74 1.75-2.24 1.25-1.74 1.00-1.24 0.75-0.99 <0.75

Incidence rate per 100,000.

*Union territories. ETL is epidemiological transition level.

Males

Males	Lip and oral cavity cancer (95% uncertainty interval)	Lung cancer (95% uncertainty interval)	Pharynx cancer other than nasopharynx (95% uncertainty interval)	Stomach cancer (95% uncertainty interval)	Prostate cancer (95% uncertainty interval)	Colon and rectum cancer (95% uncertainty interval)	Larynx cancer (95% uncertainty interval)	Oesophageal cancer (95% uncertainty interval)	Liver cancer (95% uncertainty interval)	Leukaemia (95% uncertainty interval)
**India**	9.9 (9.0 to 10.4)	7.1 (6.6 to 7.5)	6.7 (5.8 to 7.3)	5.8 (5.6 to 6.1)	4.8 (3.8 to 5.8)	4.6 (4.2 to 4.9)	3.8 (3.7 to 4.0)	3.3 (3.2 to 3.4)	3.1 (2.9 to 3.2)	2.9 (2.6 to 3.5)
**Low ETL**	9.3 (8.4 to 9.9)	6.0 (5.5 to 6.3)	7.0 (6.0 to 7.8)	6.0 (5.7 to 6.4)	3.7 (3.0 to 4.5)	4.1 (3.7 to 4.4)	3.7 (3.4 to 3.9)	3.0 92.9 to 3.2)	2.9 (2.6 to 3.1)	2.7 (2.4 to 3.3)
Bihar	3.6 (3.1 to 4.1)	4.8 (4.3 to 5.3)	1.5 (1.2 to 1.9)	2.9 (2.7 to 3.1)	3.1 (2.4 to 3.7)	3.2 (2.9 to 3.5)	2.6 (2.2 to 3.2)	3.2 (2.9 to 3.6)	2.8 (2.1 to 3.2)	2.0 (1.7 to 2.5)
Jharkhand	9.4 (8.1 to 10.6)	4.1 (3.6 to 4.7)	2.4 (1.9 to 3.0)	5.7 (5.3 to 6.3)	3.7 (3.0 to 4.5)	3.5 (3.0 to 3.8)	3.1 (2.8 to 3.6)	0.7 (0.6 to 0.8)	2.9 (2.5 to 3.3)	2.8 (2.3 to 3.2)
Uttar Pradesh	10.6 (9.4 to 11.6)	6.6 (6.0 to 7.2)	7.4 (5.9 to 8.9)	7.6 (7.0 to 8.2)	3.7 (2.9 to 4.5)	4.4 (3.8 to 4.9)	3.8 (3.3 to 4.3)	1.7 (1.5 to 1.8)	2.9 (2.6 to 3.3)	2.9 (2.5 to 3.4)
Rajasthan	7.6 (6.7 to 8.5)	5.8 (5.3 to 6.4)	10.4 (8.3 to 12.8)	7.2 (6.7 to 7.8)	3.4 (2.7 to 4.2)	3.9 (3.4 to 4.3)	3.5 (3.0 to 3.8)	3.3 (2.9 to 3.6)	2.8 (2.5 to 3.2)	2.0 (1.6 to 3.3)
Meghalaya	11.0 (10.0 to 12.2)	6.7 (6.1 to 7.3)	14.4 (12.7 to 16.2)	6.6 (6.2 to 7.2)	2.6 (2.2 to 3.5)	3.1 (2.7 to 3.3)	4.7 (4.3 to 5.1)	24.9 (22.9 to 27.0)	2.3 (2.1 to 2.6)	1.8 (1.5 to 3.2)
Assam	12.3 (10.9 to 13.9)	7.2 (6.5 to 7.9)	13.6 (11.6 to 15.2)	5.4 (5.0 to 5.8)	4.5 (3.2 to 5.1)	4.9 (4.3 to 5.3)	4.8 (4.4 to 5.3)	12.0 (10.9 to 13.3)	2.6 (2.3 to 3.0)	2.6 (2.3 to 4.0)
Chhattisgarh	8.8 (7.9 to 9.7)	5.5 (5.0 to 6.1)	7.1 (5.8 to 8.5)	6.2 (5.8 to 6.7)	4.1 (3.2 to 4.9)	4.5 (4.0 to 5.1)	4.4 (3.6 to 4.8)	1.8 (1.6 to 1.9)	3.0 (2.7 to 3.6)	3.0 (2.6 to 3.8)
Madhya Pradesh	14.6 (13.1 to 16.0)	6.9 (6.2 to 7.5)	9.4 (7.4 to 11.5)	4.4 (4.1 to 4.8)	4.0 (3.1 to 4.7)	3.9 (3.5 to 4.4)	4.0 (3.6 to 4.4)	3.3 (3.1 to 3.6)	2.5 (2.1 to 3.0)	3.1 (2.6 to 3.5)
Odisha	8.7 (7.6 to 9.7)	5.6 (4.9 to 6.3)	6.0 (4.7 to 7.2)	7.4 (6.8 to 8.0)	4.9 (3.9 to 5.8)	5.4 (4.7 to 6.1)	4.3 (3.9 to 4.9)	2.2 (2.0 to 2.5)	3.7 (3.2 to 4.2)	3.1 (2.7 to 3.6)
**Lower-middle ETL**	15.3 (13.6 to 16.7)	7.6 (6.9 to 8.1)	6.8 (5.7 to 7.7)	3.8 (3.6 to 4.1)	4.3 (3.7 to 5.6)	4.3 (3.9 to 4.6)	3.5 (3.2 to 3.7)	4.3 (4.0 to 4.6)	2.3 (2.1 to 2.5)	3.3 (2.7 to 3.6)
Arunachal Pradesh	4.6 (4.0 to 5.0)	5.4 (4.8 to 5.9)	5.5 (4.5 to 6.6)	16.7 (15.5 to 17.9)	2.4 (2.0 to 3.2)	2.9 (2.6 to 3.2)	2.8 (2.6 to 3.0)	5.3 (4.8 to 5.8)	11.4 (10.2 to 12.8)	2.4 (2.1 to 2.9)
Mizoram	6.1 (5.5 to 6.6)	18.9 (17.2 to 20.5)	8.3 (7.1 to 9.4)	25.1 (23.4 to 26.9)	3.7 (3.2 to 5.1)	7.0 (6.3 to 7.6)	3.8 (3.5 to 4.2)	16.3 (15.0 to 17.7)	5.5 (4.9 to 6.0)	3.7 (2.5 to 4.2)
Nagaland	5.6 (5.0 to 6.1)	3.9 (3.6 to 4.2)	7.4 (6.5 to 8.4)	11.1 (10.3 to 11.9)	3.3 (2.8 to 4.9)	4.7 (4.3 to 5.1)	3.1 (2.8 to 3.3)	8.1 (7.4 to 8.9)	3.5 (3.2 to 3.9)	2.0 (1.7 to 3.1)
Uttarakhand	10.8 (9.4 to 11.8)	9.2 (8.0 to 10.1)	9.7 (7.4 to 11.9)	8.3 (7.6 to 9.6)	5.7 (4.5 to 6.9)	5.3 (4.4 to 5.9)	4.5 (3.8 to 5.0)	2.4 (2.2 to 2.7)	3.4 (2.9 to 4.2)	3.1 (2.6 to 4.3)
Gujarat	18.0 (15.8 to 19.7)	6.9 (6.2 to 7.5)	6.5 (5.4 to 7.5)	2.0 (1.8 to 2.3)	4.3 (3.6 to 5.5)	4.1 (3.7 to 4.5)	3.4 (3.1 to 3.7)	4.2 (3.9 to 4.6)	1.8 (1.6 to 2.1)	3.4 (2.7 to 3.9)
Tripura	8.1 (7.4 to 8.8)	11.6 (10.6 to 12.5)	7.6 (6.7 to 8.3)	4.0 (3.8 to 4.3)	4.0 (3.5 to 5.4)	4.5 (4.1 to 4.9)	4.4 (4.0 to 4.8)	5.1 (4.6 to 5.5)	2.0 (1.8 to 2.3)	2.2 (1.9 to 3.5)
Sikkim	5.4 (4.5 to 6.0)	5.2 (4.5 to 5.8)	3.5 (2.7 to 4.0)	8.0 (7.4 to 9.0)	3.5 (2.9 to 4.8)	3.9 (3.4 to 4.3)	3.1 (2.8 to 3.5)	5.6 (5.0 to 6.2)	5.5 (4.8 to 6.2)	2.5 (2.2 to 3.0)
Manipur	3.7 (3.4 to 4.0)	12.1 (11.0 to 13.0)	2.1 (1.8 to 2.3)	4.6 (4.3 to 5.0)	4.2 (3.6 to 5.8)	4.4 (3.9 to 4.8)	1.9 (1.8 to 2.1)	2.6 (2.4 to 2.8)	2.4 (2.2 to 2.6)	2.7 (2.3 to 3.3)
**Higher-middle ETL**	9.3 (8.4 to 9.8)	7.5 (6.8 to 8.0)	6.6 (5.7 to 7.5)	5.9 (5.7 to 6.3)	5.5 (4.4 to 6.7)	4.8 (4.4 to 5.1)	4.1 (3.9 to 4.3)	3.1 (2.9 to 3.2)	3.3 (3.2 to 3.5)	3.1 (2.7 to 3.6)
Haryana	12.0 (10.7 to 13.3)	8.1 (7.4 to 8.9)	16.0 (12.7 to 19.5)	7.8 (7.3 to 8.5)	5.5 (4.4 to 6.8)	5.5 (4.9 to 6.1)	5.3 (4.0 to 6.1)	5.7 (5.1 to 6.3)	3.5 (3.0 to 4.8)	3.2 (2.6 to 4.6)
Delhi	13.0 (10.6 to 15.3)	10.7 (8.9 to 12.3)	5.4 (4.1 to 6.3)	2.8 (2.5 to 3.3)	7.2 (4.8 to 8.6)	5.0 (4.2 to 5.5)	6.6 (5.8 to 7.5)	3.6 (3.1 to 4.0)	2.2 (1.9 to 2.5)	4.9 (2.7 to 6.0)
Telangana	3.7 (3.2 to 4.2)	4.6 (4.0 to 5.2)	4.1 (3.1 to 5.0)	5.7 (5.2 to 6.3)	4.6 (3.9 to 6.2)	3.5 (3.0 to 3.9)	3.8 (3.3 to 5.0)	0.9 (0.8 to 0.9)	3.7 (3.1 to 4.2)	2.8 (2.2 to 3.3)
Andhra Pradesh	4.4 (3.9 to 4.8)	5.3 (4.8 to 5.8)	4.7 (3.8 to 5.7)	6.5 (6.1 to 7.0)	4.7 (3.8 to 5.8)	4.0 (3.6 to 4.3)	4.4 (4.0 to 4.9)	1.1 (1.0 to 1.1)	3.8 (3.3 to 4.4)	3.0 (2.6 to 3.5)
Jammu and Kashmir	3.2 (2.8 to 3.4)	12.8 (11.6 to 13.9)	4.9 (3.9 to 5.8)	15.1 (13.9 to 17.1)	4.6 (3.6 to 5.5)	3.5 (3.2 to 3.8)	3.5 (3.2 to 3.9)	3.0 (2.7 to 3.3)	3.2 (2.9 to 3.6)	2.8 (2.4 to 3.2)
Karnataka	10.9 (9.7 to 12.0)	6.9 (6.1 to 7.5)	12.6 (9.9 to 15.4)	8.3 (7.7 to 9.0)	5.3 (4.2 to 6.4)	4.8 (4.2 to 5.2)	4.2 (3.7 to 4.7)	3.3 (3.1 to 3.6)	3.7 (3.3 to 4.2)	3.2 (2.8 to 3.7)
West Bengal	10.2 (9.2 to 11.2)	10.8 (9.7 to 11.9)	6.0 (4.9 to 7.1)	6.5 (6.0 to 7.0)	5.3 (4.0 to 6.1)	5.0 (4.4 to 5.5)	4.4 (4.0 to 4.9)	2.6 (2.4 to 2.9)	3.1 (2.7 to 3.6)	3.0 (2.5 to 3.4)
Maharashtra	10.5 (9.4 to 11.5)	5.7 (5.1 to 6.2)	3.8 (3.1 to 4.3)	3.2 (2.9 to 3.6)	6.4 (5.1 to 7.8)	5.5 (4.9 to 6.0)	3.2 (2.9 to 3.5)	4.1 (3.8 to 4.4)	3.1 (2.8 to 3.4)	3.1 (2.8 to 3.9)
UTs* other than Delhi	13.0 (11.6 to 14.5)	5.2 (4.6 to 5.7)	5.6 (4.5 to 6.8)	4.7 (4.4 to 5.3)	4.5 (3.5 to 6.2)	3.5 (3.1 to 4.0)	3.1 (2.7 to 3.8)	1.6 (1.4 to 1.7)	2.9 (2.5 to 3.3)	2.6 (2.3 to 3.2)
**High ETL**	11.0 (9.9 to 11.8)	10.6 (9.7 to 11.2)	5.4 (4.7 to 6.0)	5.9 (5.6 to 6.2)	7.4 (5.8 to 8.9)	6.7 (6.0 to 7.0)	4.1 (3.8 to 4.3)	4.6 (4.4 to 4.9)	3.5 (3.3 to 3.8)	3.4 (2.8 to 3.8)
Himachal Pradesh	10.2 (8.9 to 11.4)	8.4 (7.4 to 9.3)	11.6 (8.8 to 14.5)	6.2 (5.6 to 7.2)	7.3 (5.6 to 9.0)	5.5 (4.8 to 6.1)	4.2 (3.5 to 5.3)	5.2 (4.7 to 5.8)	4.2 (3.5 to 4.9)	3.0 (2.5 to 3.4)
Punjab	5.8 (5.3 to 6.3)	7.7 (7.2 to 8.3)	2.6 (2.3 to 2.8)	2.7 (2.6 to 2.9)	6.8 (5.3 to 8.1)	5.1 (4.7 to 5.6)	2.7 (2.5 to 2.9)	5.8 (5.3 to 6.3)	2.5 (2.2 to 2.7)	4.0 (3.0 to 4.4)
Tamil Nadu	10.7 (9.4 to 11.8)	7.9 (7.1 to 8.5)	5.1 (4.2 to 5.8)	6.8 (6.4 to 7.3)	6.4 (5.4 to 8.3)	6.0 (5.4 to 6.5)	3.3 (3.0 to 3.7)	4.2 (3.8 to 4.5)	2.7 (2.4 to 3.0)	2.9 (2.6 to 4.0)
Goa	7.5 (6.6 to 8.3)	4.4 (3.9 to 4.8)	6.6 (5.3 to 7.9)	6.3 (5.8 to 7.0)	8.2 (6.4 to 10.7)	4.4 (4.0 to 4.8)	4.7 (4.0 to 5.7)	1.8 (1.6 to 1.9)	4.6 (3.8 to 5.4)	2.9 (2.6 to 3.5)
Kerala	16.7 (14.7 to 18.4)	19.5 (17.5 to 21.3)	7.3 (6.1 to 8.3)	6.6 (6.1 to 7.1)	10.3 (6.7 to 12.0)	9.8 (8.7 to 10.6)	6.7 (6.2 to 7.4)	4.5 (4.2 to 4.9)	6.0 (5.4 to 6.7)	4.2 (2.4 to 4.8)

Ratio of the state incidence to median incidence of all states

Colour key	
Red	>2.74
Light red	2.25-2.74
Orange	1.75-2.24
Dark yellow	1.25-1.74
Light yellow	1.00-1.24
Light blue	0.75-0.99
Blue	<0.75

Incidence rate per 100,000.

*Union territories. ETL is epidemiological transition level.

12. Percent change of age-standardised incidence for different types of cancers in the states of India by sex, 1990 to 2016

Females

Females

States of India	Breast cancer	Cervical cancer	Lip and oral cavity cancer	Stomach cancer	Colon and rectum cancer	Ovarian cancer	Pharynx cancer other than nasopharynx	Lung cancer	Gallbladder and biliary tract cancer	Thyroid cancer	Oesophageal cancer	Uterine cancer	Leukaemia
**India**	**39.1 (5.1 to 85.5)**	**-40.2 (-57.6 to -27.1)**	**-11.8 (-16.9 to -5.9)**	**-42.7 (-48.2 to -32.7)**	**1.1 (-19.2 to 16.0)**	**27.2 (18.0 to 40.2)**	**-23.5 (-34.2 to -7.6)**	**14.3 (1.0 to 24.1)**	**18.5 (-19.0 to 34.8)**	**29.8 (13.7 to 60.2)**	**-38.8 (-41.4 to -35.9)**	**2.7 (-7.5 to 17.8)**	**-28.4 (-44.2 to -6.7)**
**Low ETL**	**32.0 (-1.4 to 62.4)**	**-37.5 (-69.7 to -19.9)**	**-12.5 (-19.3 to -4.6)**	**-39.4 (-45.3 to -28.9)**	**-4.7 (-24.2 to 10.8)**	**23.1 (14.8 to 32.6)**	**-18.9 (-30.4 to -3.1)**	**22.2 (7.7 to 35.8)**	**33.5 (-16.7 to 58.1)**	**0.5 (-11.8 to 23.0)**	**-24.1 (-29.2 to -18.6)**	**-22.4 (-32.0 to -7.8)**	**-31.7 (-49.5 to -1.2)**
Bihar	35.2 (5.1 to 61.9)	-32.8 (-72.3 to -13.3)	-14.1 (-26.2 to -0.7)	-26.5 (-36.1 to -15.2)	8.8 (-6.7 to 28.5)	11.0 (-1.7 to 25.0)	-16.3 (-30.1 to 0.0)	67.6 (17.3 to 101.9)	52.6 (-12.2 to 84.2)	-17.1 (-28.0 to -0.2)	**	-29.2 (-41.6 to -12.2)	-28.1 (-54.5 to -1.9)
Jharkhand	43.4 (-3.6 to 91.0)	-54.9 (-79.5 to -23.0)	-5.6 (-17.9 to 12.3)	-33.6 (-42.5 to -21.9)	1.9 (-23.0 to 27.2)	27.7 (9.2 to 48.9)	-15.6 (-32.7 to 6.6)	18.3 (-1.1 to 41.2)	44.3 (-18.0 to 85.6)	10.5 (-7.2 to 38.1)	-38.5 (-45.8 to -30.6)	-22.1 (-36.2 to -3.5)	-28.6 (-45.0 to 3.2)
Uttar Pradesh	26.6 (-5.9 to 62.0)	-36.3 (-70.5 to -16.4)	-11.0 (-22.6 to 2.3)	-39.1 (-47.0 to -28.2)	-8.0 (-29.6 to 13.2)	20.0 (6.8 to 35.2)	-16.7 (-32.1 to 4.6)	18.1 (-0.2 to 38.4)	46.7 (-15.1 to 84.5)	-3.1 (-18.7 to 21.8)	-36.9 (-44.2 to -29.3)	-25.4 (-37.7 to -4.9)	-31.3 (-53.4 to 6.7)
Rajasthan	30.9 (-2.9 to 64.6)	-36.6 (-75.1 to -16.5)	-10.0 (-22.1 to 4.6)	-36.8 (-45.0 to -24.9)	0.7 (-22.3 to 21.4)	21.9 (4.9 to 45.3)	-14.5 (-30.6 to 3.6)	10.1 (-10.2 to 29.5)	45.8 (-15.8 to 72.8)	10.8 (-5.3 to 35.2)	-39.8 (-46.6 to -31.5)	-20.0 (-32.8 to -2.2)	-33.3 (-54.3 to -8.7)
Meghalaya	44.7 (-4.9 to 99.4)	-32.5 (-55.8 to -3.2)	3.0 (-9.1 to 17.0)	-43.4 (-51.5 to -29.8)	2.7 (-26.9 to 30.6)	29.3 (9.2 to 47.9)	-18.7 (-35.4 to 8.8)	43.7 (18.5 to 67.9)	-15.7 (-36.5 to 8.0)	54.5 (18.6 to 107.1)	-32.7 (-39.9 to -24.2)	5.7 (-13.7 to 37.0)	-30.0 (-44.3 to -0.3)
Assam	22.0 (-17.9 to 78.4)	-39.9 (-66.3 to -14.7)	-7.5 (-20.0 to 6.6)	-43.9 (-50.8 to -33.3)	-5.2 (-29.4 to 19.9)	18.9 (3.6 to 37.2)	-31.0 (-43.8 to -9.1)	6.3 (-10.0 to 22.2)	-18.4 (-36.6 to 5.0)	23.0 (-3.9 to 62.5)	-42.5 (-49.6 to -34.8)	3.5 (-15.0 to 30.6)	-34.6 (-49.3 to -7.6)
Chhattisgarh	53.6 (1.4 to 113.3)	-32.5 (-47.4 to -11.7)	-3.3 (-16.0 to 13.7)	-33.6 (-43.3 to -20.0)	11.4 (-16.8 to 39.3)	35.6 (14.0 to 60.1)	-11.6 (-29.2 to 13.3)	17.9 (-2.9 to 46.5)	46.6 (-14.8 to 88.6)	27.1 (4.6 to 64.4)	-38.5 (-45.3 to -30.2)	-15.2 (-29.4 to 7.2)	-23.9 (-38.8 to 4.7)
Madhya Pradesh	42.1 (3.6 to 74.3)	-35.7 (-62.7 to -15.8)	-17.2 (-26.8 to -5.8)	-43.7 (-50.7 to -33.4)	-7.6 (-29.0 to 11.6)	36.9 (21.6 to 53.9)	-33.6 (-48.9 to -11.1)	6.2 (-9.3 to 20.7)	31.6 (-20.7 to 61.2)	2.5 (-16.1 to 38.7)	-37.9 (-44.6 to -29.5)	-11.7 (-27.8 to 11.1)	-33.4 (-47.8 to 1.9)
Odisha	27.7 (-15.2 to 76.8)	-40.3 (-64.5 to -18.5)	-11.0 (-22.3 to 1.6)	-42.5 (-50.4 to -31.5)	-10.5 (-36.4 to 13.2)	24.6 (9.1 to 43.5)	-24.4 (-41.0 to -0.8)	7.6 (-12.8 to 25.2)	31.5 (-21.2 to 73.8)	3.4 (-15.0 to 33.7)	-45.8 (-52.4 to -38.8)	-27.4 (-41.6 to -6.3)	-36.0 (-49.0 to -6.5)
**Lower-middle ETL**	**31.4 (2.8 to 98.7)**	**-38.4 (-46.9 to -26.3)**	**6.9 (-3.6 to 18.8)**	**-45.5 (-52.0 to -35.7)**	**28.0 (3.9 to 49.0)**	**23.9 (11.4 to 43.8)**	**-32.3 (-41.8 to -18.3)**	**13.4 (-0.3 to 24.9)**	**-6.1 (-19.4 to 7.4)**	**54.0 (30.9 to 88.7)**	**-39.4 (-44.8 to -32.9)**	**28.8 (13.1 to 51.5)**	**-20.0 (-43.1 to -0.6)**
Arunachal Pradesh	41.0 (-8.4 to 95.4)	-34.1 (-58.3 to 3.7)	-35.7 (-44.3 to -26.3)	-42.9 (-51.6 to -29.6)	5.2 (-22.4 to 31.0)	43.8 (18.9 to 68.8)	-39.6 (-53.3 to -17.4)	3.9 (-17.9 to 23.7)	-19.5 (-41.0 to 12.6)	59.2 (21.0 to 121.4)	-36.8 (-45.6 to -26.5)	4.6 (-15.0 to 38.4)	-41.2 (-57.4 to -2.0)
Mizoram	31.8 (-9.7 to 90.3)	-30.7 (-41.6 to -9.2)	-4.9 (-15.8 to 7.2)	-43.1 (-49.3 to -32.5)	-4.5 (-28.6 to 19.1)	34.0 (16.8 to 52.6)	-30.0 (-42.7 to -11.8)	14.5 (-4.0 to 30.5)	-16.3 (-32.2 to 0.4)	82.7 (47.2 to 132.2)	-37.1 (-44.8 to -28.7)	24.4 (2.0 to 57.6)	-32.3 (-46.9 to -10.8)
Nagaland	69.6 (3.4 to 136.9)	-31.6 (-65.6 to 2.1)	1.6 (-10.8 to 15.6)	-47.3 (-54.5 to -35.6)	29.5 (-9.3 to 65.6)	22.5 (-0.9 to 54.1)	-19.9 (-37.4 to 8.8)	14.9 (-8.3 to 36.3)	-14.0 (-37.2 to 16.3)	168.1 (103.5 to 257.7)	-34.7 (-42.6 to -24.9)	26.6 (3.7 to 75.8)	-35.6 (-50.4 to -0.9)
Uttarakhand	52.0 (-15.0 to 148.2)	-50.9 (-63.2 to -22.3)	-2.7 (-13.3 to 9.0)	-54.8 (-62.6 to -40.9)	-14.9 (-44.3 to 20.1)	36.0 (14.7 to 64.8)	-29.7 (-47.2 to 3.5)	-4.1 (-22.9 to 14.8)	15.9 (-34.9 to 70.3)	57.1 (19.9 to 135.3)	-56.6 (-63.3 to -45.9)	-16.8 (-34.2 to 14.9)	-38.2 (-53.0 to -8.3)
Gujarat	28.1 (4.2 to 92.2)	-35.9 (-44.0 to -22.8)	10.3 (-2.6 to 25.4)	-36.9 (-43.1 to -29.3)	47.0 (25.1 to 69.4)	20.7 (6.5 to 41.6)	-35.6 (-43.5 to -27.5)	19.1 (4.9 to 33.6)	-5.8 (-17.1 to 8.8)	36.9 (15.9 to 88.3)	-36.8 (-43.7 to -29.0)	50.7 (29.8 to 75.8)	-13.4 (-40.9 to 4.0)
Tripura	30.0 (-11.2 to 77.8)	-32.7 (-49.0 to -11.3)	-4.1 (-16.1 to 8.9)	-41.9 (-49.7 to -29.1)	-3.7 (-28.5 to 20.0)	32.9 (13.6 to 54.5)	-18.2 (-35.0 to 7.5)	3.1 (-14.7 to 19.5)	-17.1 (-35.9 to 3.4)	45.9 (15.6 to 87.6)	-31.9 (-40.0 to -22.8)	4.7 (-14.7 to 30.7)	-30.1 (-44.0 to -4.6)
Sikkim	44.0 (-11.6 to 117.4)	-41.2 (-53.8 to -14.4)	-9.5 (-22.9 to 4.5)	-51.8 (-59.3 to -39.3)	-2.0 (-33.8 to 35.3)	57.1 (33.1 to 81.3)	-49.7 (-63.4 to -24.5)	-3.6 (-23.5 to 13.4)	-38.0 (-56.1 to -15.1)	90.4 (41.6 to 171.4)	-52.7 (-61.1 to -41.0)	-2.8 (-23.7 to 33.0)	-42.5 (-58.7 to -14.7)
Manipur	53.4 (-2.0 to 117.1)	-38.1 (-56.1 to -20.0)	-4.8 (-17.2 to 9.5)	-43.4 (-50.5 to -33.0)	9.2 (-19.3 to 35.6)	24.7 (4.5 to 50.4)	-22.8 (-35.9 to -4.2)	10.9 (-5.7 to 30.1)	-18.9 (-33.5 to -1.9)	64.8 (32.5 to 115.9)	-41.0 (-48.7 to -33.1)	16.6 (-2.5 to 58.1)	-31.5 (-43.7 to -7.8)
**Higher-middle ETL**	**39.9 (2.9 to 96.0)**	**-41.7 (-48.2 to -28.5)**	**-12.6 (-18.0 to -6.6)**	**-47.0 (-52.8 to -37.4)**	**4.3 (-18.0 to 21.3)**	**25.9 (15.7 to 46.8)**	**-27.0 (-39.8 to -8.8)**	**7.6 (-6.8 to 17.2)**	**12.3 (-23.2 to 30.6)**	**35.0 (15.0 to 75.2)**	**-48.1 (-51.2 to -44.5)**	**9.6 (-3.5 to 28.8)**	**-28.3 (-46.9 to -10.4)**
Haryana	61.7 (10.3 to 120.8)	-33.4 (-47.8 to -17.6)	-9.4 (-22.3 to 7.6)	-39.5 (-47.7 to -28.0)	8.3 (-19.9 to 37.0)	32.1 (11.7 to 57.9)	-16.6 (-35.9 to 10.1)	6.3 (-13.4 to 30.4)	46.9 (-12.3 to 78.8)	101.7 (65.7 to 154.3)	-44.8 (-52.2 to -36.4)	12.2 (-6.5 to 40.2)	-25.2 (-41.1 to -0.1)
Delhi	57.1 (12.8 to 132.5)	-41.4 (-50.3 to -26.4)	-0.4 (-14.1 to 16.4)	-51.0 (-56.9 to -41.7)	7.1 (-19.1 to 31.7)	14.6 (-0.7 to 50.4)	-37.5 (-50.5 to -17.8)	9.3 (-8.0 to 28.1)	-10.4 (-27.6 to 8.4)	135.7 (81.9 to 229.7)	-45.1 (-52.3 to -36.1)	85.8 (47.6 to 138.6)	-11.5 (-44.5 to 16.5)
Telangana	41.1 (-13.2 to 116.5)	-42.8 (-54.6 to -16.9)	-10.9 (-22.4 to 2.6)	-49.6 (-57.2 to -38.8)	2.8 (-29.9 to 35.1)	28.1 (7.9 to 52.8)	-27.4 (-47.4 to 4.5)	-2.4 (-23.1 to 15.0)	25.7 (-24.2 to 65.7)	41.7 (5.4 to 107.0)	-52.7 (-58.2 to -46.8)	-18.7 (-35.9 to 11.3)	-34.9 (-50.8 to -11.3)
Andhra Pradesh	34.5 (-12.6 to 87.3)	-41.7 (-52.5 to -16.0)	-11.3 (-22.1 to 0.9)	-47.1 (-54.3 to -36.1)	2.2 (-26.9 to 28.4)	21.6 (6.2 to 44.9)	-26.5 (-43.4 to 3.6)	9.8 (-12.0 to 28.0)	26.4 (-24.4 to 61.4)	23.6 (-4.6 to 67.7)	-49.6 (-55.7 to -42.4)	-21.2 (-35.1 to 2.3)	-34.2 (-48.8 to -14.1)
Jammu and Kashmir	33.6 (-15.4 to 106.0)	-43.1 (-54.5 to -20.0)	-11.8 (-22.1 to 0.4)	-51.0 (-58.2 to -39.6)	0.3 (-29.7 to 31.0)	31.2 (13.9 to 51.4)	-18.5 (-36.8 to 11.2)	-2.1 (-19.7 to 13.5)	25.0 (-28.1 to 65.8)	27.4 (-1.6 to 80.8)	-50.7 (-58.7 to -39.1)	-22.0 (-37.0 to 4.8)	-33.8 (-49.7 to -9.2)
Karnataka	45.5 (8.6 to 106.2)	-37.6 (-47.7 to -18.5)	-11.4 (-23.3 to 6.3)	-41.1 (-48.9 to -30.2)	10.3 (-13.7 to 33.4)	31.0 (13.1 to 57.4)	-21.1 (-40.0 to 4.1)	22.3 (2.9 to 45.4)	34.6 (-22.6 to 62.4)	17.6 (0.7 to 86.6)	-46.1 (-51.6 to -39.6)	6.3 (-9.0 to 29.3)	-19.2 (-38.6 to -2.0)
West Bengal	25.7 (-15.4 to 82.7)	-45.6 (-56.7 to -21.3)	-15.2 (-25.3 to -3.6)	-45.6 (-52.1 to -35.9)	-14.5 (-37.7 to 9.7)	33.1 (14.4 to 51.1)	-34.2 (-49.8 to -7.2)	13.7 (-4.0 to 29.8)	7.3 (-26.9 to 33.4)	7.8 (-15.0 to 52.9)	-49.8 (-55.2 to -43.6)	-11.1 (-27.1 to 16.7)	-37.6 (-50.8 to -13.3)
Maharashtra	34.2 (8.0 to 104.2)	-42.5 (-51.3 to -31.9)	-12.6 (-22.3 to -1.8)	-46.6 (-51.6 to -40.3)	21.3 (4.2 to 38.3)	15.4 (4.1 to 49.0)	-40.8 (-47.6 to -33.7)	-6.4 (-17.7 to 10.1)	-22.3 (-31.2 to -10.5)	39.6 (19.0 to 104.4)	-51.0 (-56.6 to -43.8)	56.5 (34.2 to 83.1)	-21.4 (-45.9 to -7.0)
UTs* other than Delhi	57.2 (5.6 to 117.5)	-38.3 (-49.6 to -16.8)	-7.5 (-18.9 to 4.8)	-37.3 (-45.9 to -21.7)	10.6 (-19.2 to 41.1)	14.7 (1.5 to 41.5)	-8.4 (-28.8 to 24.2)	1.5 (-15.9 to 18.7)	30.6 (-24.5 to 65.3)	97.7 (55.3 to 163.9)	-33.0 (-43.0 to -19.5)	11.1 (-10.0 to 43.4)	-27.5 (-40.6 to -13.2)
**High ETL**	**56.0 (16.2 to 121.0)**	**-45.0 (-51.8 to -27.9)**	**-14.1 (-21.4 to -4.8)**	**-38.9 (-44.9 to -29.8)**	**7.5 (-14.3 to 25.2)**	**42.3 (27.2 to 56.3)**	**-29.9 (-41.0 to -14.8)**	**8.4 (-6.7 to 23.3)**	**-12.3 (-24.9 to -0.1)**	**110.3 (83.3 to 145.9)**	**-50.9 (-54.6 to -46.8)**	**75.5 (57.3 to 107.7)**	**-20.4 (-43.9 to -1.3)**
Himachal Pradesh	37.9 (-22.3 to 139.8)	-52.4 (-63.6 to -21.8)	-11.3 (-27.3 to 6.6)	-58.5 (-65.4 to -44.1)	-7.0 (-41.1 to 38.3)	32.3 (2.4 to 59.1)	-32.7 (-53.7 to 9.7)	-5.3 (-23.8 to 12.1)	17.4 (-38.0 to 74.7)	72.0 (19.5 to 173.2)	-59.6 (-67.0 to -49.4)	-12.3 (-34.2 to 36.2)	-36.5 (-57.3 to -5.3)
Punjab	50.9 (3.3 to 121.4)	-40.8 (-50.4 to -21.4)	5.7 (-8.1 to 22.2)	-31.3 (-41.4 to -15.2)	-11.9 (-32.2 to 9.5)	48.4 (24.1 to 70.9)	-0.9 (-17.5 to 28.6)	-11.0 (-25.4 to 4.3)	-17.9 (-31.3 to -4.3)	119.9 (78.3 to 183.1)	-50.2 (-57.7 to -39.6)	57.7 (32.7 to 105.0)	-28.2 (-42.1 to -6.5)
Tamil Nadu	48.5 (6.3 to 110.8)	-47.8 (-56.0 to -32.8)	-15.0 (-23.6 to -4.6)	-34.2 (-41.5 to -24.1)	11.8 (-16.2 to 35.8)	45.1 (28.0 to 61.3)	-29.7 (-41.2 to -15.6)	6.4 (-11.2 to 22.9)	-19.6 (-31.5 to -3.8)	111.0 (72.9 to 157.4)	-46.3 (-51.8 to -40.1)	53.5 (29.9 to 88.4)	-24.1 (-48.7 to 1.3)
Goa	80.8 (17.2 to 155.7)	-40.3 (-52.2 to -4.7)	-24.4 (-32.8 to -14.6)	-49.3 (-56.3 to -36.5)	13.8 (-19.2 to 44.8)	21.7 (8.5 to 52.7)	-15.0 (-35.5 to 16.7)	-7.7 (-27.7 to 7.8)	26.0 (-26.2 to 61.4)	158.7 (95.4 to 265.0)	-54.6 (-62.1 to -40.9)	32.0 (6.0 to 69.4)	-29.0 (-48.1 to -1.0)
Kerala	69.1 (38.5 to 132.5)	-35.0 (-45.8 to -10.4)	-13.8 (-25.2 to 3.2)	-36.0 (-42.6 to -28.3)	16.0 (0.1 to 34.7)	36.1 (17.3 to 65.8)	-30.9 (-40.6 to -20.9)	25.1 (4.6 to 48.0)	-10.4 (-22.5 to 7.6)	122.5 (90.8 to 184.0)	-58.3 (-63.0 to -52.9)	131.5 (91.8 to 176.3)	-6.9 (-28.2 to 7.6)

Range of percent change

>50.0 | 25.1 to 50.0 | 0.1 to 25.0 | -25.1 to 0.0 | -50.1 to -25.0 | < -50.1

*Union Territories. **These estimates are not reported because of issues with the data. ETL is epidemiological transition level.

Females

States of India	Non-Hodgkin lymphoma	Pancreatic cancer	Brain and nervous system cancer	Liver cancer	Non-melanoma skin cancer	Larynx cancer	Bladder cancer	Kidney cancer	Multiple myeloma	Nasopharynx cancer	Hodgkin's lymphoma	Malignant skin melanoma	Mesothelioma
**India**	**33.3 (5.1 to 47.1)**	**-0.9 (-7.3 to 6.9)**	**8.2 (-8.5 to 74.6)**	**23.4 (-10.5 to 44.9)**	**-6.4 (-14.4 to 2.0)**	**-53.7 (-57.0 to -50.9)**	**0.2 (-16.0 to 13.6)**	**23.3 (8.7 to 32.2)**	**5.7 (-21.9 to 41.6)**	**-56.7 (-73.2 to -38.0)**	**-54.6 (-60.2 to -45.5)**	**26.3 (-7.1 to 69.3)**	**-25.9 (-50.0 to -5.3)**
**Low ETL**	**34.3 (1.9 to 49.9)**	**1.3 (-6.5 to 10.6)**	**0.8 (-13.3 to 55.9)**	**32.1 (-10.3 to 58.9)**	**7.3 (0.8 to 14.8)**	**-53.8 (-57.4 to -49.9)**	**-5.0 (-19.6 to 9.0)**	**15.5 (-3.0 to 26.5)**	**1.7 (-23.8 to 34.2)**	**-60.1 (-80.1 to -27.6)**	**-58.3 (-67.4 to -43.6)**	**12.2 (-13.7 to 39.7)**	**-28.1 (-49.5 to -8.1)**
Bihar	29.3 (4.0 to 48.9)	-7.2 (-18.2 to 5.7)	-6.4 (-25.1 to 28.4)	41.7 (-9.8 to 77.3)	9.4 (0.0 to 17.1)	-54.3 (-60.7 to -47.2)	-13.6 (-26.2 to 2.2)	2.8 (-16.6 to 22.7)	-4.7 (-19.7 to 22.3)	-62.7 (-84.2 to -13.2)	-60.1 (-72.8 to -41.4)	1.4 (-12.2 to 20.4)	-26.6 (-47.8 to -5.7)
Jharkhand	31.1 (3.8 to 53.7)	5.1 (-11.9 to 29.6)	5.8 (-20.5 to 84.1)	37.2 (-12.1 to 79.6)	7.1 (-1.5 to 17.5)	-54.0 (-60.3 to -46.1)	1.1 (-15.7 to 24.4)	17.8 (-8.0 to 44.4)	3.2 (-25.3 to 48.1)	-54.1 (-74.0 to -25.8)	-55.6 (-68.5 to -35.2)	17.9 (-10.4 to 45.6)	-32.0 (-53.4 to -5.7)
Uttar Pradesh	33.0 (3.6 to 53.2)	-1.1 (-13.4 to 13.4)	-1.4 (-18.4 to 50.0)	39.1 (-8.4 to 77.4)	9.9 (2.7 to 19.0)	-50.8 (-57.2 to -43.6)	-9.5 (-24.2 to 5.3)	13.0 (-8.2 to 32.0)	-1.0 (-23.8 to 33.7)	-59.6 (-81.2 to -21.3)	-56.8 (-69.2 to -38.1)	8.9 (-13.8 to 35.4)	-25.8 (-47.8 to -3.2)
Rajasthan	37.3 (1.4 to 65.4)	5.9 (-8.4 to 25.3)	1.3 (-18.8 to 67.8)	39.0 (-11.2 to 74.2)	10.5 (3.3 to 20.5)	-53.3 (-59.4 to -46.0)	-6.8 (-21.4 to 12.5)	19.0 (5.6 to 34.7)	3.0 (-24.1 to 41.8)	-63.7 (-83.9 to -22.4)	-60.8 (-71.4 to -47.3)	15.2 (-10.3 to 44.4)	-27.4 (-49.0 to -5.3)
Meghalaya	25.1 (4.9 to 48.7)	12.6 (-2.5 to 31.0)	20.7 (-10.0 to 91.1)	-11.8 (-37.3 to 13.4)	11.8 (2.7 to 21.5)	-2.6 (-17.2 to 12.9)	9.7 (-17.6 to 40.7)	44.9 (13.3 to 82.0)	2.8 (-31.0 to 45.2)	-44.8 (-60.3 to -25.7)	-40.9 (-51.5 to -23.4)	38.4 (-7.3 to 69.9)	-27.2 (-52.1 to -2.2)
Assam	6.4 (-8.5 to 20.3)	0.9 (-11.4 to 15.7)	9.7 (-12.8 to 45.5)	-15.4 (-38.8 to 7.1)	2.8 (-8.3 to 16.4)	-50.6 (-57.5 to -42.8)	0.4 (-24.1 to 25.4)	25.8 (-1.3 to 51.3)	-5.2 (-40.3 to 19.2)	-53.1 (-70.1 to -29.1)	-43.4 (-51.5 to -27.5)	19.6 (-21.5 to 49.7)	-29.1 (-51.7 to 0.6)
Chhattisgarh	33.6 (4.3 to 64.4)	21.1 (2.8 to 48.0)	10.4 (-18.4 to 105.5)	37.5 (-13.2 to 80.3)	10.3 (4.0 to 19.2)	-51.3 (-58.2 to -44.4)	41.0 (6.2 to 75.1)	28.3 (3.6 to 59.8)	12.8 (-24.6 to 61.8)	-45.3 (-60.8 to -25.6)	-52.2 (-65.8 to -28.9)	31.5 (-5.6 to 61.2)	-20.1 (-46.3 to 12.3)
Madhya Pradesh	43.4 (2.4 to 66.1)	1.5 (-10.0 to 15.1)	2.6 (-15.5 to 59.6)	23.4 (-11.6 to 60.4)	-4.4 (-18.2 to 13.1)	-56.0 (-61.7 to -48.8)	-13.4 (-30.2 to 4.5)	16.5 (-12.8 to 36.2)	14.0 (-17.3 to 38.0)	-64.2 (-80.7 to -37.2)	-57.8 (-67.0 to -46.7)	13.4 (-18.0 to 45.4)	-30.6 (-52.2 to -7.4)
Odisha	42.4 (2.0 to 64.2)	2.1 (-10.2 to 15.6)	3.4 (-18.2 to 71.5)	30.4 (-13.1 to 76.3)	8.7 (-0.3 to 22.1)	-61.7 (-67.4 to -55.4)	-11.0 (-30.2 to 12.0)	20.4 (2.1 to 40.9)	3.8 (-32.7 to 35.9)	-55.8 (-75.3 to -32.2)	-64.8 (-74.7 to -50.8)	16.4 (-20.5 to 50.4)	-34.9 (-58.0 to -11.3)
**Lower-middle ETL**	**29.7 (6.4 to 48.3)**	**-1.5 (-11.7 to 10.6)**	**17.8 (-5.1 to 87.0)**	**8.2 (-12.6 to 29.3)**	**-45.8 (-58.0 to -28.8)**	**-51.4 (-55.9 to -46.9)**	**3.4 (-14.1 to 19.0)**	**23.2 (7.5 to 42.5)**	**13.6 (-18.0 to 47.5)**	**-57.8 (-64.5 to -50.9)**	**-45.5 (-52.2 to -38.8)**	**10.2 (-18.5 to 74.6)**	**-20.3 (-47.7 to 2.6)**
Arunachal Pradesh	39.0 (4.9 to 61.4)	18.7 (1.7 to 41.3)	16.5 (-11.7 to 73.0)	-9.5 (-38.6 to 22.2)	11.6 (1.9 to 26.4)	-28.0 (-39.9 to -15.2)	4.6 (-21.4 to 36.5)	43.8 (10.6 to 71.9)	6.4 (-36.2 to 39.9)	-47.1 (-63.5 to -23.6)	-65.1 (-74.1 to -51.6)	38.4 (-14.8 to 74.6)	-40.2 (-61.1 to -17.2)
Mizoram	24.8 (5.3 to 42.5)	16.9 (6.1 to 29.3)	12.4 (-14.3 to 74.8)	-16.9 (-39.5 to 4.8)	-31.5 (-50.5 to -4.7)	-41.3 (-49.3 to -32.1)	7.7 (-15.0 to 30.5)	40.5 (18.6 to 65.3)	-6.3 (-40.9 to 35.4)	-32.8 (-44.7 to -16.2)	-41.6 (-53.4 to -30.3)	51.5 (-1.7 to 81.4)	-22.6 (-47.3 to -0.6)
Nagaland	-6.2 (-21.9 to 19.3)	-1.7 (-15.2 to 14.9)	19.4 (-16.1 to 87.3)	14.9 (-19.3 to 51.6)	8.5 (1.5 to 20.6)	6.2 (-10.4 to 24.0)	17.0 (-11.9 to 50.1)	43.8 (13.7 to 81.1)	13.3 (-34.7 to 48.2)	-55.3 (-74.3 to -29.6)	-35.9 (-46.3 to -15.4)	61.0 (-1.2 to 103.1)	-41.0 (-62.7 to -19.5)
Uttarakhand	33.8 (7.8 to 54.2)	8.6 (-3.2 to 22.3)	14.7 (-18.7 to 122.7)	21.0 (-20.6 to 72.4)	9.1 (0.2 to 18.0)	-64.7 (-70.2 to -59.0)	7.9 (-29.1 to 49.1)	32.7 (0.8 to 59.8)	9.8 (-42.6 to 44.9)	-57.1 (-69.1 to -38.7)	-60.0 (-72.4 to -32.7)	49.0 (-21.0 to 103.8)	-34.5 (-63.4 to -1.5)
Gujarat	32.2 (8.2 to 55.5)	-4.7 (-16.2 to 9.4)	19.9 (-2.6 to 83.1)	13.3 (-9.0 to 39.6)	-60.9 (-72.3 to -41.1)	-50.8 (-56.5 to -44.0)	-0.5 (-13.5 to 15.2)	19.7 (1.1 to 41.9)	15.5 (-9.1 to 49.4)	-64.4 (-69.6 to -58.7)	-40.9 (-48.5 to -30.8)	1.2 (-20.3 to 71.1)	-15.7 (-43.3 to 8.7)
Tripura	13.8 (-2.7 to 32.4)	14.5 (-0.2 to 32.6)	14.7 (-9.1 to 63.7)	-11.2 (-35.4 to 12.5)	8.0 (-0.6 to 22.0)	-4.6 (-18.5 to 11.4)	9.3 (-13.3 to 34.8)	40.0 (16.4 to 66.6)	7.9 (-32.8 to 33.5)	-39.2 (-54.3 to -21.3)	-58.8 (-70.4 to -41.6)	21.0 (-13.5 to 53.4)	-24.9 (-50.4 to -2.7)
Sikkim	16.7 (-0.7 to 37.6)	7.2 (-5.1 to 21.2)	-18.5 (-39.2 to 83.3)	-19.2 (-50.0 to 9.6)	9.8 (-2.0 to 27.9)	-63.3 (-69.5 to -56.5)	-9.9 (-37.6 to 24.2)	26.2 (2.8 to 47.7)	-12.9 (-53.1 to 36.4)	-48.2 (-55.9 to -36.4)	-58.5 (-72.5 to -44.5)	34.7 (-39.6 to 92.9)	-39.6 (-65.1 to -9.3)
Manipur	12.6 (-3.0 to 27.9)	9.1 (-6.5 to 28.5)	22.9 (-10.1 to 99.0)	-12.5 (-34.8 to 11.3)	-13.6 (-25.9 to 11.0)	-41.6 (-49.9 to -32.5)	19.6 (-3.0 to 44.9)	37.8 (9.7 to 74.0)	2.7 (-33.6 to 48.5)	-45.9 (-59.3 to -30.1)	-41.3 (-51.3 to -32.2)	39.3 (-3.7 to 72.1)	-24.1 (-49.7 to 4.5)
**Higher-middle ETL**	**37.0 (8.0 to 49.1)**	**-6.2 (-13.1 to 1.0)**	**10.4 (-9.2 to 81.9)**	**21.0 (-11.8 to 44.2)**	**-7.2 (-15.8 to 4.7)**	**-55.1 (-59.2 to -52.0)**	**0.8 (-16.2 to 16.7)**	**25.4 (12.6 to 36.9)**	**5.9 (-24.4 to 41.5)**	**-50.9 (-59.8 to -41.5)**	**-54.9 (-59.6 to -48.9)**	**34.9 (-5.0 to 86.4)**	**-27.6 (-53.3 to -5.7)**
Haryana	50.4 (10.6 to 89.0)	11.7 (-6.3 to 35.8)	15.5 (-16.5 to 115.3)	40.5 (-10.1 to 81.2)	13.2 (2.8 to 31.1)	-48.9 (-56.3 to -40.7)	18.6 (-3.0 to 44.0)	36.1 (15.0 to 66.7)	20.5 (-14.1 to 68.1)	-41.8 (-58.4 to -20.2)	-49.2 (-63.0 to -30.1)	47.9 (11.6 to 96.9)	-16.9 (-47.6 to 13.4)
Delhi	62.2 (35.3 to 86.2)	-14.3 (-24.5 to -2.9)	10.0 (-15.8 to 73.1)	1.3 (-28.4 to 28.7)	-17.0 (-27.5 to -4.0)	-42.3 (-52.2 to -32.1)	-7.2 (-24.4 to 11.3)	34.3 (8.1 to 64.7)	32.4 (-3.5 to 56.0)	-59.3 (-65.1 to -51.8)	2.0 (-16.3 to 24.1)	78.9 (28.2 to 150.3)	-30.7 (-55.5 to -6.8)
Telangana	45.0 (6.6 to 70.9)	0.7 (-11.4 to 15.4)	7.9 (-18.0 to 105.2)	27.0 (-15.9 to 73.7)	13.3 (3.4 to 26.3)	-63.9 (-68.8 to -58.3)	-6.9 (-30.4 to 18.3)	29.6 (9.1 to 50.5)	3.4 (-37.7 to 44.7)	-49.2 (-59.4 to -33.9)	-60.8 (-72.9 to -40.6)	37.3 (-16.5 to 85.1)	-27.6 (-55.9 to 2.6)
Andhra Pradesh	40.3 (4.0 to 64.1)	0.3 (-10.1 to 12.4)	4.0 (-18.4 to 77.0)	34.0 (-8.6 to 80.0)	8.4 (0.2 to 16.7)	-60.6 (-66.3 to -54.3)	-7.3 (-28.4 to 17.9)	27.6 (8.2 to 45.6)	3.6 (-27.2 to 37.0)	-44.7 (-53.6 to -30.6)	-65.8 (-74.3 to -53.7)	30.8 (-17.0 to 67.2)	-28.5 (-54.9 to -1.9)
Jammu and Kashmir	37.2 (4.4 to 57.7)	-0.7 (-11.1 to 10.1)	3.8 (-19.0 to 74.7)	26.3 (-14.2 to 71.7)	8.5 (1.7 to 17.4)	-62.9 (-68.1 to -57.0)	-11.4 (-33.5 to 15.0)	22.2 (-5.5 to 42.5)	-0.8 (-38.5 to 31.6)	-47.3 (-55.0 to -34.0)	-60.9 (-72.9 to -39.7)	28.8 (-18.7 to 70.8)	-34.7 (-59.1 to -7.3)
Karnataka	62.5 (14.4 to 104.8)	4.4 (-12.2 to 25.5)	15.8 (-10.1 to 116.3)	35.3 (-10.7 to 69.2)	0.0 (-7.4 to 10.8)	-44.6 (-51.8 to -36.8)	7.8 (-8.2 to 30.6)	32.8 (14.1 to 59.1)	13.5 (-17.6 to 64.6)	-36.9 (-50.2 to -22.2)	-57.9 (-68.8 to -40.7)	30.9 (3.3 to 77.2)	-13.2 (-43.9 to 20.2)
West Bengal	25.8 (3.4 to 44.4)	-2.1 (-12.3 to 8.9)	1.1 (-15.7 to 45.0)	12.9 (-19.8 to 49.6)	-16.2 (-24.7 to -3.1)	-58.4 (-64.0 to -52.3)	-13.2 (-32.1 to 10.4)	21.0 (-2.3 to 41.6)	-1.0 (-36.8 to 22.6)	-56.1 (-69.8 to -37.9)	-62.3 (-68.8 to -55.7)	37.6 (-14.3 to 67.2)	-34.2 (-57.7 to -10.8)
Maharashtra	18.8 (4.1 to 37.0)	-20.2 (-29.9 to -9.3)	15.6 (-8.4 to 83.7)	9.7 (-10.0 to 31.2)	-25.2 (-49.0 to 5.5)	-51.2 (-57.1 to -45.3)	11.1 (-8.5 to 32.2)	18.2 (0.4 to 38.0)	-1.5 (-23.5 to 41.9)	-64.9 (-68.8 to -60.3)	-44.1 (-56.7 to -31.5)	21.0 (-1.4 to 100.8)	-29.2 (-55.2 to -7.0)
UTs* other than Delhi	86.8 (24.3 to 115.4)	6.3 (-4.2 to 17.5)	7.3 (-16.1 to 73.9)	36.3 (-8.2 to 77.5)	9.4 (1.3 to 17.6)	-54.8 (-60.7 to -48.0)	25.1 (-0.3 to 48.6)	25.8 (6.8 to 43.3)	20.0 (-12.4 to 39.4)	-43.5 (-54.3 to -29.6)	-43.7 (-59.9 to -18.6)	81.3 (19.3 to 111.1)	-24.9 (-53.1 to -2.8)
**High ETL**	**23.7 (9.1 to 46.5)**	**6.2 (-4.8 to 21.1)**	**22.8 (-1.0 to 103.6)**	**8.2 (-14.2 to 32.1)**	**-24.9 (-32.5 to -13.1)**	**-46.7 (-51.7 to -41.6)**	**16.2 (-3.1 to 36.1)**	**40.8 (25.2 to 63.1)**	**11.8 (-17.5 to 56.9)**	**-50.5 (-56.6 to -42.4)**	**-37.0 (-48.0 to -26.8)**	**45.9 (4.2 to 101.0)**	**-16.0 (-47.4 to 9.1)**
Himachal Pradesh	43.8 (11.4 to 70.8)	-2.7 (-18.3 to 13.5)	4.0 (-17.9 to 76.0)	21.2 (-28.0 to 79.1)	11.5 (-2.3 to 22.3)	-66.1 (-71.5 to -60.1)	-1.1 (-30.5 to 32.3)	23.4 (-7.3 to 48.1)	-0.9 (-47.7 to 36.6)	-54.4 (-61.8 to -39.6)	-59.2 (-74.8 to -34.1)	55.4 (-19.5 to 115.4)	-39.9 (-66.1 to -6.3)
Punjab	32.1 (8.3 to 64.8)	18.5 (-0.3 to 45.3)	23.9 (-8.3 to 91.8)	-6.9 (-30.3 to 22.1)	-7.0 (-14.4 to 4.7)	-14.8 (-28.4 to -0.8)	12.8 (-10.0 to 38.9)	53.6 (24.0 to 96.7)	23.7 (-17.6 to 58.5)	-26.3 (-36.4 to -13.6)	-38.2 (-46.5 to -28.5)	41.5 (7.4 to 88.1)	-20.8 (-46.2 to 4.5)
Tamil Nadu	33.2 (7.6 to 56.8)	-0.7 (-12.7 to 13.9)	24.7 (-1.4 to 94.9)	0.9 (-24.4 to 27.6)	-46.1 (-59.9 to -24.9)	-48.6 (-55.3 to -41.5)	12.2 (-10.3 to 36.3)	33.7 (14.7 to 56.6)	16.7 (-20.4 to 49.3)	-51.1 (-59.2 to -39.9)	-41.2 (-55.0 to -24.0)	51.0 (-3.5 to 96.3)	-18.8 (-50.5 to 10.1)
Goa	65.9 (30.6 to 95.1)	-1.9 (-11.7 to 9.6)	11.9 (-12.3 to 86.7)	35.0 (-7.7 to 73.1)	10.0 (2.5 to 18.8)	-54.5 (-61.2 to -47.2)	19.7 (-2.6 to 40.0)	35.2 (19.9 to 53.1)	20.1 (-14.3 to 48.7)	-34.7 (-48.4 to -16.9)	-44.3 (-58.7 to -22.9)	83.6 (21.6 to 158.6)	-15.7 (-53.6 to 11.5)
Kerala	11.0 (-7.1 to 41.6)	11.8 (-4.6 to 35.6)	22.5 (-4.4 to 146.0)	29.5 (-8.2 to 64.3)	-7.6 (-21.5 to 9.0)	-46.9 (-53.2 to -39.4)	31.1 (15.0 to 55.6)	52.7 (24.9 to 89.8)	7.0 (-13.5 to 87.3)	-54.9 (-61.4 to -49.0)	-23.1 (-36.5 to -8.8)	34.7 (10.9 to 110.3)	2.7 (-32.8 to 41.0)

Range of percent change

>50.0 | 25.1 to 50.0 | 0.1 to 25.0 | -25.1 to 0.0 | -50.1 to -25.0 | < -50.1

*Union Territories. ETL is epidemiological transition level.

Males

States of India	Lip and oral cavity cancer	Lung cancer	Pharynx cancer other than nasopharynx	Stomach cancer	Prostate cancer	Colon and rectum cancer	Larynx cancer	Oesophageal cancer	Liver cancer	Leukaemia	Non-Hodgkin lymphoma	Bladder cancer	Brain and nervous system cancer
**India**	-1.8 (-20.9 to 8.2)	0.2 (-15.9 to 8.9)	10.9 (-17.7 to 34.1)	-36.7 (-41.5 to -27.9)	34.3 (12.5 to 51.6)	21.4 (-5.1 to 36.8)	-23.2 (-28.8 to -17.9)	-24.6 (-30.6 to -18.9)	38.4 (25.3 to 50.2)	-4.7 (-13.0 to 2.5)	24.3 (-3.2 to 45.7)	21.8 (7.4 to 29.5)	0.6 (-15.6 to 110.3)
**Low ETL**	**-1.3 (-21.5 to 11.6)**	**13.6 (-5.6 to 24.3)**	18.5 (-12.9 to 46.1)	**-31.1 (-37.5 to -20.7)**	**22.2 (4.6 to 33.6)**	**11.9 (-14.8 to 29.2)**	**-23.9 (-30.0 to -17.6)**	**-9.9 (-18.6 to -0.5)**	**41.3 (26.0 to 57.2)**	**-8.8 (-22.3 to -0.2)**	**25.9 (-3.0 to 41.8)**	**18.9 (6.6 to 29.5)**	**-7.0 (-22.7 to 85.6)**
Bihar	-18.3 (-34.5 to -1.4)	10.9 (-6.1 to 28.1)	-0.1 (-28.9 to 30.2)	-31.9 (-39.6 to -21.6)	9.8 (-7.8 to 23.7)	8.4 (-12.8 to 26.2)	-36.8 (-46.8 to -25.5)	**	40.7 (15.1 to 66.6)	-12.0 (-25.7 to 3.6)	16.2 (-7.4 to 42.1)	3.7 (-15.1 to 21.2)	-14.8 (-33.2 to 31.1)
Jharkhand	-12.9 (-33.1 to 4.1)	-7.0 (-29.6 to 14.6)	9.7 (-28.0 to 52.4)	-36.8 (-45.4 to -23.7)	24.4 (-2.0 to 44.8)	10.2 (-21.7 to 36.4)	-34.2 (-44.0 to -23.6)	-24.6 (-37.1 to -7.1)	42.1 (16.6 to 67.1)	-10.9 (-24.4 to 2.3)	23.6 (-2.4 to 46.5)	16.5 (-3.9 to 33.6)	-8.2 (-27.6 to 94.7)
Uttar Pradesh	0.0 (-22.2 to 19.2)	24.6 (0.3 to 42.3)	19.9 (-19.0 to 60.9)	-25.7 (-34.8 to -12.8)	20.7 (5.5 to 35.8)	10.1 (-18.0 to 33.3)	-19.4 (-30.5 to -8.0)	-25.6 (-35.4 to -14.1)	47.0 (25.6 to 72.0)	-8.5 (-25.5 to 5.2)	26.4 (-2.1 to 47.7)	20.2 (4.0 to 37.8)	-8.6 (-27.2 to 83.3)
Rajasthan	5.6 (-20.4 to 24.2)	9.7 (-11.4 to 30.7)	34.0 (-11.4 to 85.2)	-29.0 (-37.4 to -15.6)	23.5 (7.4 to 38.7)	14.9 (-14.2 to 39.8)	-22.0 (-33.1 to -10.9)	-21.5 (-32.6 to -9.1)	47.8 (27.2 to 75.9)	-4.6 (-23.4 to 14.6)	31.4 (-4.6 to 64.5)	17.8 (1.3 to 36.3)	-6.6 (-26.3 to 107.7)
Meghalaya	17.2 (-13.3 to 38.7)	34.5 (4.7 to 60.3)	29.2 (-13.4 to 78.4)	-33.3 (-42.7 to -17.0)	38.1 (15.6 to 67.3)	10.5 (-20.7 to 37.5)	12.1 (-5.6 to 32.7)	-4.9 (-19.1 to 10.5)	3.3 (-15.3 to 32.2)	-0.2 (-19.6 to 26.0)	22.6 (0.4 to 66.6)	39.2 (7.1 to 69.7)	19.5 (-13.8 to 150.1)
Assam	2.4 (-23.0 to 24.2)	7.1 (-14.0 to 25.0)	9.9 (-20.6 to 37.3)	-38.0 (-45.7 to -26.8)	32.4 (7.1 to 52.8)	19.1 (-13.0 to 43.8)	-12.3 (-24.5 to 2.5)	-21.6 (-32.5 to -7.9)	6.0 (-15.9 to 28.3)	-7.5 (-21.6 to 5.9)	2.1 (-14.4 to 32.1)	15.9 (-5.6 to 34.5)	13.0 (-13.5 to 86.3)
Chhattisgarh	5.7 (-16.3 to 23.3)	11.0 (-11.9 to 31.9)	34.3 (-6.1 to 85.8)	-27.9 (-37.4 to -12.8)	37.3 (14.2 to 64.1)	21.2 (-14.9 to 54.2)	-19.0 (-31.1 to -6.2)	-22.8 (-32.8 to -12.0)	45.5 (20.5 to 78.3)	-4.8 (-19.6 to 12.8)	30.7 (-0.3 to 66.1)	57.9 (30.2 to 83.0)	-1.8 (-27.2 to 144.7)
Madhya Pradesh	7.4 (-13.5 to 22.7)	18.5 (-3.2 to 34.1)	9.4 (-23.2 to 46.4)	-30.9 (-39.6 to -18.1)	30.1 (10.9 to 47.8)	27.9 (-2.0 to 56.0)	-16.2 (-27.4 to -4.3)	-19.4 (-29.5 to -8.3)	36.0 (14.6 to 62.4)	-6.4 (-18.7 to 4.8)	38.1 (-2.8 to 68.7)	23.8 (5.5 to 43.1)	-1.7 (-21.6 to 101.4)
Odisha	-8.2 (-33.3 to 8.8)	-11.0 (-33.0 to 7.1)	13.3 (-28.9 to 60.3)	-44.4 (-51.7 to -32.9)	24.0 (-3.4 to 43.9)	2.1 (-29.8 to 25.3)	-36.3 (-45.1 to -24.6)	-33.8 (-46.3 to -21.2)	45.1 (17.0 to 71.6)	-12.6 (-24.8 to -1.4)	29.7 (-5.4 to 52.0)	12.4 (-11.0 to 32.1)	-7.4 (-26.3 to 96.7)
**Lower-middle ETL**	**6.8 (-10.1 to 19.6)**	**-10.3 (-24.8 to -0.5)**	**-12.1 (-32.9 to 6.9)**	**-41.2 (-47.0 to -29.8)**	**41.1 (19.0 to 65.1)**	**33.9 (7.1 to 51.9)**	**-25.8 (-34.2 to -18.1)**	**-27.2 (-34.7 to -19.3)**	**35.8 (18.8 to 54.8)**	**6.9 (-4.7 to 18.6)**	**27.0 (-2.0 to 57.4)**	**16.5 (0.4 to 27.9)**	**14.7 (-8.2 to 126.7)**
Arunachal Pradesh	-10.4 (-30.9 to 5.8)	-10.2 (-28.1 to 5.7)	-5.4 (-37.6 to 38.9)	-32.8 (-42.1 to -17.8)	33.5 (7.9 to 55.6)	56.8 (14.1 to 96.6)	1.7 (-16.9 to 22.3)	-9.2 (-22.8 to 6.5)	12.3 (-8.3 to 40.0)	-7.2 (-18.9 to 5.1)	16.0 (-5.6 to 58.6)	34.9 (4.7 to 65.5)	17.1 (-9.8 to 125.7)
Mizoram	14.9 (-11.1 to 34.0)	12.5 (-9.8 to 29.3)	14.9 (-20.3 to 50.9)	-33.1 (-40.1 to -20.6)	43.1 (23.1 to 68.6)	44.4 (8.6 to 71.6)	-0.1 (-13.7 to 15.2)	0.1 (-13.2 to 13.4)	8.6 (-8.1 to 31.7)	1.0 (-16.6 to 20.8)	21.4 (-3.2 to 59.0)	36.6 (12.8 to 55.9)	16.7 (-13.3 to 125.9)
Nagaland	8.3 (-19.1 to 31.0)	19.4 (-6.7 to 46.3)	23.6 (-17.8 to 74.7)	-30.8 (-40.2 to -15.1)	52.7 (25.2 to 92.8)	59.4 (12.7 to 105.2)	20.1 (-1.1 to 44.0)	-17.0 (-30.4 to -1.2)	46.7 (21.5 to 84.2)	-4.7 (-26.6 to 24.6)	2.8 (-21.4 to 71.3)	47.1 (17.9 to 86.0)	17.7 (-15.9 to 155.0)
Uttarakhand	-0.5 (-28.1 to 17.6)	-10.4 (-34.1 to 8.3)	16.9 (-31.6 to 73.3)	-43.9 (-53.3 to -20.9)	54.3 (9.5 to 98.6)	26.4 (-20.0 to 62.4)	-27.5 (-38.4 to -10.9)	-30.9 (-43.6 to -14.9)	46.9 (15.8 to 78.0)	-5.4 (-22.8 to 19.4)	35.2 (-1.2 to 76.1)	45.8 (11.7 to 74.0)	4.8 (-26.6 to 192.6)
Gujarat	7.4 (-8.0 to 21.3)	-15.1 (-27.5 to -4.4)	-20.7 (-38.4 to -4.1)	-44.4 (-50.6 to -34.6)	39.1 (20.1 to 60.2)	35.6 (15.3 to 52.7)	-29.5 (-38.1 to -20.9)	-30.7 (-38.6 to -21.7)	45.8 (24.6 to 69.3)	10.7 (-2.2 to 25.9)	28.5 (-2.4 to 55.1)	8.4 (-5.6 to 21.8)	16.7 (-6.4 to 115.5)
Tripura	13.8 (-15.8 to 36.8)	16.3 (-8.1 to 37.6)	20.0 (-19.4 to 65.0)	-29.7 (-38.8 to -15.2)	30.5 (11.7 to 50.2)	23.5 (-6.5 to 49.2)	9.3 (-8.4 to 27.8)	-10.9 (-23.8 to 5.7)	7.1 (-10.2 to 32.4)	-1.2 (-19.5 to 15.5)	14.5 (-4.4 to 54.5)	34.2 (9.0 to 58.6)	16.6 (-12.9 to 115.8)
Sikkim	4.2 (-26.1 to 25.4)	-10.3 (-33.4 to 7.5)	14.8 (-31.3 to 60.3)	-46.9 (-54.7 to -26.6)	42.8 (11.6 to 78.5)	24.8 (-11.8 to 55.4)	-16.0 (-30.7 to 0.6)	-23.2 (-35.9 to -10.1)	-1.2 (-20.9 to 21.9)	-10.0 (-23.2 to 9.9)	17.7 (-7.4 to 70.4)	19.8 (-11.1 to 41.5)	10.5 (-16.6 to 161.1)
Manipur	7.0 (-16.1 to 25.2)	1.6 (-17.3 to 19.1)	13.8 (-19.0 to 48.8)	-37.9 (-45.2 to -25.8)	44.2 (23.1 to 70.6)	33.7 (-2.1 to 67.5)	-3.4 (-17.8 to 11.3)	-20.1 (-30.2 to -9.3)	2.3 (-13.4 to 25.7)	-0.5 (-18.3 to 20.6)	6.5 (-13.7 to 63.6)	33.8 (12.5 to 57.2)	20.7 (-11.2 to 154.0)
**Higher-middle ETL**	**-5.8 (-25.1 to 3.9)**	**-5.7 (-22.6 to 5.1)**	**5.3 (-25.7 to 34.4)**	**-41.4 (-46.2 to -32.2)**	**39.3 (15.8 to 60.7)**	**27.4 (-0.2 to 43.7)**	**-25.6 (-32.7 to -18.4)**	**-33.9 (-39.8 to -28.2)**	**38.5 (25.7 to 53.1)**	**-4.0 (-10.8 to 4.2)**	**24.2 (-3.7 to 52.6)**	**21.0 (5.0 to 29.0)**	**3.7 (-15.1 to 122.6)**
Haryana	13.5 (-14.2 to 40.4)	21.2 (-0.3 to 49.5)	44.0 (-6.1 to 119.5)	-24.0 (-33.4 to -7.6)	59.0 (41.0 to 88.5)	44.0 (7.0 to 87.4)	1.4 (-21.9 to 18.6)	-15.2 (-30.1 to 2.3)	55.0 (30.2 to 97.5)	3.0 (-17.6 to 37.0)	47.4 (3.9 to 105.7)	56.0 (33.1 to 88.5)	8.4 (-25.9 to 200.0)
Delhi	33.3 (1.2 to 64.4)	11.4 (-12.0 to 33.0)	24.1 (-15.7 to 58.7)	-35.8 (-43.5 to -24.7)	79.3 (44.9 to 110.7)	33.3 (5.1 to 56.2)	1.5 (-14.3 to 20.8)	-15.6 (-29.4 to -0.6)	26.3 (4.0 to 51.2)	20.6 (2.3 to 50.5)	48.5 (13.7 to 75.4)	26.2 (4.2 to 46.0)	9.7 (-20.2 to 116.1)
Telangana	-0.5 (-30.4 to 17.9)	-7.9 (-30.8 to 10.4)	31.6 (-24.1 to 96.0)	-43.5 (-51.6 to -30.1)	37.7 (7.4 to 73.9)	40.1 (-6.7 to 79.0)	-29.9 (-41.1 to -15.8)	-22.4 (-35.7 to -8.8)	46.0 (18.0 to 76.8)	-8.4 (-20.7 to 10.2)	28.3 (-4.0 to 66.3)	24.0 (-2.1 to 45.7)	-2.7 (-26.0 to 165.3)
Andhra Pradesh	5.7 (-21.8 to 23.7)	6.5 (-17.9 to 26.7)	36.2 (-15.1 to 99.3)	-37.4 (-44.8 to -25.5)	32.7 (11.6 to 50.1)	45.7 (8.3 to 78.3)	-21.3 (-32.8 to -9.7)	-12.6 (-23.7 to 0.6)	55.9 (27.5 to 92.6)	-5.9 (-16.6 to 7.8)	23.8 (-8.3 to 62.0)	25.4 (2.0 to 45.7)	-2.9 (-27.5 to 144.3)
Jammu and Kashmir	-5.5 (-31.0 to 12.4)	-12.6 (-32.9 to 2.1)	28.3 (-22.2 to 78.7)	-45.1 (-53.7 to -25.1)	38.8 (6.2 to 65.3)	28.4 (-8.6 to 55.0)	-31.0 (-40.3 to -18.7)	-35.5 (-46.4 to -21.2)	50.8 (23.7 to 76.7)	-7.7 (-18.8 to 5.2)	30.2 (-4.7 to 54.6)	23.6 (1.4 to 43.2)	-3.8 (-27.1 to 125.3)
Karnataka	-6.9 (-30.3 to 8.3)	8.3 (-14.1 to 25.0)	0.1 (-37.2 to 47.2)	-39.4 (-46.8 to -28.8)	41.4 (15.4 to 63.0)	26.7 (-3.2 to 49.5)	-26.2 (-36.4 to -14.5)	-33.5 (-42.3 to -24.2)	49.4 (29.1 to 77.0)	2.1 (-10.3 to 13.2)	45.0 (-6.3 to 80.7)	30.8 (11.4 to 47.6)	2.1 (-18.8 to 138.2)
West Bengal	-10.1 (-34.0 to 6.8)	-4.4 (-26.0 to 15.7)	3.5 (-35.1 to 44.0)	-37.7 (-45.2 to -26.7)	35.7 (5.0 to 55.7)	6.7 (-23.0 to 25.7)	-30.3 (-39.8 to -17.6)	-33.4 (-43.6 to -19.5)	30.1 (5.2 to 51.2)	-11.9 (-22.9 to -1.2)	12.8 (-6.5 to 28.5)	19.2 (-2.2 to 35.7)	-4.1 (-22.6 to 76.8)
Maharashtra	-17.4 (-29.1 to -7.3)	-28.9 (-39.7 to -20.0)	-22.3 (-39.2 to -8.3)	-50.3 (-55.6 to -41.7)	30.4 (9.2 to 69.9)	30.7 (12.2 to 46.6)	-34.0 (-41.3 to -26.1)	-44.9 (-51.0 to -38.1)	27.1 (10.6 to 44.0)	-6.1 (-15.0 to 1.9)	8.3 (-6.3 to 46.1)	1.1 (-11.2 to 11.7)	11.2 (-11.0 to 113.1)
UTs* other than Delhi	7.9 (-15.7 to 27.0)	11.6 (-6.5 to 35.8)	39.0 (-10.3 to 99.0)	-30.4 (-41.3 to -7.5)	63.3 (36.8 to 89.8)	30.9 (-5.3 to 60.2)	-14.2 (-28.4 to -1.0)	-19.6 (-30.8 to -6.9)	49.2 (28.3 to 75.7)	-2.5 (-16.6 to 21.4)	70.1 (0.3 to 122.9)	46.7 (27.4 to 69.4)	1.1 (-24.9 to 136.6)
**High ETL**	**-1.8 (-21.6 to 8.6)**	**-9.5 (-23.8 to -0.1)**	**10.5 (-20.0 to 35.3)**	**-41.4 (-46.4 to -33.5)**	**52.2 (23.2 to 76.9)**	**34.6 (6.9 to 52.8)**	**-11.3 (-20.7 to -2.3)**	**-35.2 (-40.7 to -29.9)**	**31.0 (16.6 to 46.9)**	**2.8 (-6.7 to 12.8)**	**19.3 (-2.9 to 61.8)**	**33.0 (12.5 to 46.3)**	**12.2 (-9.6 to 138.2)**
Himachal Pradesh	2.7 (-26.8 to 24.4)	-1.4 (-25.9 to 19.1)	42.7 (-19.6 to 127.2)	-37.2 (-47.8 to -9.9)	63.5 (26.3 to 100.0)	29.0 (-12.2 to 68.4)	-15.0 (-28.7 to 0.3)	-27.9 (-39.9 to -14.6)	52.3 (24.0 to 88.5)	-5.1 (-21.3 to 21.2)	41.4 (2.5 to 89.7)	48.1 (18.9 to 71.9)	2.4 (-26.7 to 187.6)
Punjab	17.2 (-11.1 to 37.8)	-5.7 (-24.9 to 9.5)	43.1 (-2.6 to 91.7)	-24.8 (-36.8 to 8.1)	58.1 (28.6 to 82.8)	38.8 (5.8 to 70.7)	14.4 (-3.5 to 32.0)	-25.9 (-35.4 to -15.1)	14.0 (-5.2 to 39.5)	0.6 (-13.9 to 18.7)	39.0 (-0.7 to 98.1)	51.5 (22.4 to 80.4)	20.3 (-9.0 to 142.3)
Tamil Nadu	-2.9 (-25.5 to 11.5)	-3.3 (-21.9 to 11.8)	9.2 (-24.7 to 38.6)	-43.1 (-49.0 to -34.7)	46.5 (16.1 to 70.1)	39.0 (5.3 to 63.3)	-11.2 (-23.6 to 2.5)	-29.8 (-38.7 to -20.4)	32.8 (9.7 to 55.6)	0.4 (-10.9 to 12.4)	24.5 (-4.4 to 58.8)	37.2 (11.5 to 55.7)	21.0 (-3.4 to 137.0)
Goa	-0.8 (-28.3 to 18.8)	-14.1 (-35.1 to 2.2)	27.0 (-25.2 to 90.7)	-22.6 (-34.2 to 10.8)	75.7 (45.9 to 112.5)	65.3 (24.5 to 98.9)	-8.8 (-22.5 to 5.1)	-37.5 (-47.4 to -26.9)	56.5 (29.4 to 88.0)	-3.2 (-16.4 to 14.9)	46.9 (9.2 to 96.8)	60.5 (39.4 to 81.8)	3.8 (-23.3 to 152.0)
Kerala	-5.8 (-21.1 to 7.4)	-16.9 (-29.5 to -5.9)	-1.5 (-25.0 to 18.4)	-42.6 (-47.9 to -35.2)	55.5 (25.7 to 89.5)	27.5 (7.4 to 45.7)	-18.0 (-28.2 to -6.9)	-50.2 (-56.2 to -43.4)	32.7 (11.7 to 54.1)	8.8 (-6.0 to 25.1)	5.8 (-9.1 to 62.3)	23.9 (5.6 to 39.8)	-1.8 (-24.8 to 127.1)

Range of percent change

>50.0 | 25.1 to 50.0 | 0.1 to 25.0 | -25.1 to 0.0 | -50.1 to -25.0 | < -50.1

*Union Territories. **These estimates are not reported because of issues with the data. ETL is epidemiological transition level.

	Males											
States of India	Pancreatic cancer	Non-melanoma skin cancer	Gallbladder and biliary tract cancer	Kidney cancer	Nasopharynx cancer	Thyroid cancer	Multiple myeloma	Hodgkin's lymphoma	Testicular cancer	Breast cancer	Mesothelioma	Malignant skin melanoma
**India**	-12.8 (-16.4 to -9.7)	-10.3 (-15.5 to -3.3)	2.0 (-23.4 to 19.6)	41.6 (22.9 to 68.4)	-24.2 (-32.7 to -13.9)	16.2 (5.1 to 50.3)	31.3 (-8.0 to 76.7)	-54.4 (-61.9 to -39.1)	-35.1 (-44.0 to -29.2)	41.4 (-2.8 to 76.3)	10.7 (-23.2 to 46.6)	43.0 (-1.4 to 121.6)
**Low ETL**	**-12.2 (-17.0 to -7.4)**	**0.8 (-6.1 to 8.4)**	**6.7 (-20.3 to 27.4)**	**25.4 (7.8 to 56.5)**	**-15.6 (-27.0 to 1.7)**	**-8.4 (-18.8 to 16.7)**	**26.4 (-12.0 to 62.3)**	**-59.0 (-67.6 to -40.1)**	**-49.8 (-57.6 to -43.4)**	**26.5 (-4.7 to 57.3)**	**10.1 (-20.0 to 42.4)**	**23.0 (-7.5 to 81.9)**
Bihar	-19.4 (-28.2 to -8.7)	1.0 (-8.1 to 9.5)	8.8 (-24.2 to 43.9)	4.3 (-13.0 to 32.7)	-20.8 (-34.5 to 0.3)	-23.6 (-37.2 to 4.0)	8.6 (-20.1 to 33.5)	-63.4 (-73.7 to -44.7)	-55.7 (-64.3 to -46.0)	12.4 (-14.7 to 39.3)	5.7 (-25.2 to 33.3)	-3.9 (-26.8 to 46.9)
Jharkhand	-12.3 (-22.0 to -1.2)	-0.5 (-11.6 to 11.1)	4.7 (-27.1 to 37.1)	20.4 (-2.7 to 54.3)	-21.4 (-35.1 to -1.1)	-6.5 (-25.9 to 25.5)	19.1 (-22.7 to 62.6)	-59.8 (-71.8 to -38.6)	-57.1 (-65.2 to -46.1)	21.5 (-11.6 to 58.0)	7.1 (-28.8 to 47.0)	18.3 (-16.2 to 100.6)
Uttar Pradesh	-13.7 (-22.3 to -4.3)	4.0 (-5.8 to 15.9)	9.9 (-19.9 to 43.1)	26.0 (6.4 to 61.7)	-12.1 (-30.5 to 10.4)	-9.8 (-25.5 to 15.8)	26.5 (-10.3 to 64.0)	-57.2 (-69.0 to -36.9)	-55.4 (-63.9 to -46.8)	32.7 (-0.9 to 59.7)	13.7 (-17.7 to 51.8)	16.8 (-7.0 to 76.2)
Rajasthan	-11.3 (-20.0 to -2.0)	3.8 (-4.3 to 13.5)	7.3 (-22.0 to 38.3)	30.9 (4.4 to 65.0)	-11.2 (-27.7 to 10.9)	-4.5 (-23.1 to 21.7)	27.7 (-11.6 to 77.8)	-59.8 (-71.1 to -38.6)	-9.2 (-33.5 to 11.5)	23.8 (-0.5 to 68.9)	9.2 (-19.2 to 58.7)	34.8 (-6.9 to 87.4)
Meghalaya	3.7 (-7.6 to 15.2)	4.8 (-4.2 to 16.9)	-7.3 (-24.3 to 13.1)	64.2 (20.7 to 146.5)	-7.6 (-29.2 to 20.7)	19.4 (-2.8 to 48.6)	29.3 (-17.2 to 103.0)	-31.4 (-40.2 to -19.6)	-16.8 (-40.8 to 7.2)	8.7 (-15.2 to 91.1)	13.6 (-19.7 to 67.5)	49.0 (2.2 to 129.5)
Assam	-1.5 (-13.4 to 11.5)	-2.7 (-10.5 to 8.8)	-8.4 (-23.5 to 6.1)	49.2 (18.0 to 89.7)	-11.6 (-31.2 to 16.3)	29.5 (9.6 to 51.6)	19.4 (-24.0 to 61.6)	-37.2 (-46.2 to -27.8)	-16.5 (-38.1 to 4.7)	15.1 (-6.9 to 58.5)	9.1 (-25.8 to 44.9)	47.3 (-3.8 to 117.1)
Chhattisgarh	5.4 (-5.6 to 15.7)	4.2 (-6.1 to 15.4)	9.7 (-25.8 to 42.7)	38.5 (7.5 to 90.0)	-11.9 (-28.0 to 6.4)	6.1 (-13.0 to 39.0)	41.5 (-8.6 to 107.9)	-54.6 (-68.0 to -32.7)	-44.7 (-55.8 to -32.8)	43.7 (5.9 to 96.8)	18.8 (-18.0 to 78.9)	48.0 (1.4 to 142.0)
Madhya Pradesh	-11.4 (-19.9 to -1.8)	-9.5 (-18.8 to 2.4)	9.4 (-21.1 to 34.7)	31.9 (5.7 to 66.1)	-19.1 (-33.3 to 0.7)	-4.3 (-20.2 to 22.9)	53.6 (-7.6 to 116.8)	-57.9 (-68.6 to -39.6)	-35.7 (-49.1 to -21.5)	40.2 (1.3 to 73.8)	10.5 (-17.8 to 57.4)	41.3 (-0.5 to 120.9)
Odisha	-12.2 (-21.7 to -2.0)	0.0 (-10.7 to 10.9)	3.1 (-31.1 to 36.0)	25.5 (-2.1 to 61.2)	-20.6 (-38.2 to 1.7)	-10.4 (-25.8 to 21.3)	22.3 (-23.3 to 65.2)	-65.4 (-74.8 to -49.8)	-60.3 (-67.0 to -52.0)	20.3 (-14.6 to 55.9)	5.2 (-31.8 to 48.5)	28.0 (-19.9 to 101.1)
**Lower-middle ETL**	**-10.8 (-17.5 to -3.9)**	**-35.3 (-48.3 to -19.6)**	**-4.9 (-24.1 to 14.9)**	**63.1 (34.2 to 99.1)**	**-40.4 (-48.9 to -30.2)**	**41.4 (22.1 to 60.1)**	**35.1 (-7.2 to 87.9)**	**-39.7 (-47.2 to -32.8)**	**-35.2 (-46.5 to -24.1)**	**72.4 (2.3 to 105.3)**	**12.8 (-23.0 to 55.7)**	**37.4 (-8.0 to 128.8)**
Arunachal Pradesh	14.1 (0.9 to 28.4)	6.4 (-3.4 to 18.2)	-14.0 (-25.5 to 2.6)	65.0 (20.4 to 139.1)	-1.0 (-24.5 to 29.6)	34.4 (12.3 to 60.8)	32.0 (-20.0 to 95.3)	-62.8 (-73.5 to -43.0)	-1.9 (-27.5 to 24.4)	18.5 (-9.6 to 87.7)	9.2 (-23.1 to 66.1)	47.9 (-6.6 to 123.2)
Mizoram	-1.2 (-9.9 to 8.9)	-30.4 (-47.6 to -12.5)	-6.1 (-22.0 to 10.9)	64.9 (24.4 to 123.7)	-9.5 (-26.5 to 14.2)	52.0 (30.0 to 80.9)	15.0 (-23.5 to 84.7)	-34.3 (-44.7 to -23.2)	1.1 (-23.7 to 25.1)	44.8 (8.8 to 84.4)	13.8 (-19.7 to 61.0)	109.2 (35.0 to 186.1)
Nagaland	5.5 (-7.2 to 18.9)	3.6 (-8.4 to 18.3)	-8.4 (-25.3 to 9.6)	61.5 (15.1 to 153.5)	-20.9 (-38.1 to 7.1)	85.1 (55.2 to 117.4)	37.6 (-14.7 to 110.3)	-34.5 (-48.4 to -24.4)	50.0 (8.4 to 92.0)	29.1 (1.0 to 108.9)	14.6 (-22.6 to 69.7)	78.5 (5.1 to 217.9)
Uttarakhand	-0.6 (-11.1 to 11.2)	3.6 (-6.4 to 13.9)	-0.5 (-32.9 to 35.6)	52.1 (13.7 to 112.6)	-21.2 (-36.7 to 1.1)	29.8 (2.6 to 82.7)	37.4 (-26.6 to 112.0)	-56.7 (-68.2 to -35.2)	-40.8 (-51.8 to -18.1)	36.3 (-6.1 to 99.4)	15.7 (-34.9 to 72.6)	72.2 (-10.6 to 203.0)
Gujarat	-14.5 (-22.4 to -6.0)	-46.1 (-60.6 to -26.7)	-5.3 (-23.0 to 16.8)	65.4 (38.5 to 100.1)	-57.1 (-64.5 to -48.4)	46.0 (25.2 to 67.1)	35.9 (-4.5 to 82.7)	-34.9 (-43.4 to -23.3)	-36.0 (-48.0 to -23.6)	92.5 (4.0 to 133.1)	12.1 (-22.1 to 56.4)	26.7 (-12.1 to 119.6)
Tripura	9.5 (-2.3 to 23.6)	4.3 (-5.4 to 16.1)	-2.6 (-21.8 to 16.9)	61.2 (22.3 to 124.3)	-3.8 (-27.0 to 23.6)	16.7 (-4.3 to 38.4)	24.6 (-17.0 to 83.6)	-35.2 (-42.3 to -20.8)	-22.0 (-42.7 to -0.9)	9.1 (-14.4 to 75.5)	13.4 (-18.3 to 62.1)	45.0 (2.1 to 97.1)
Sikkim	2.8 (-9.0 to 14.9)	2.4 (-8.5 to 12.8)	-16.2 (-29.9 to 0.8)	73.1 (24.5 to 144.5)	-15.9 (-38.8 to 10.0)	45.4 (15.1 to 82.4)	23.8 (-32.9 to 108.4)	-31.6 (-48.0 to -11.6)	-9.3 (-32.3 to 14.1)	26.9 (-5.3 to 100.9)	9.6 (-31.0 to 64.6)	80.8 (-19.2 to 198.3)
Manipur	-2.7 (-12.2 to 7.6)	-24.2 (-36.5 to -9.1)	-9.9 (-24.4 to 8.5)	66.6 (24.4 to 141.4)	-12.3 (-29.2 to 13.7)	39.9 (18.9 to 61.3)	29.9 (-15.0 to 104.2)	-34.5 (-44.3 to -25.5)	-5.1 (-31.1 to 21.2)	31.4 (7.1 to 101.3)	18.1 (-15.7 to 74.2)	51.8 (11.7 to 138.8)
**Higher-middle ETL**	**-17.5 (-21.6 to -13.5)**	**-13.1 (-20.5 to -4.1)**	**0.7 (-26.8 to 20.6)**	**46.9 (23.4 to 76.6)**	**-28.8 (-39.0 to -19.8)**	**26.4 (11.7 to 73.2)**	**33.5 (-7.6 to 82.1)**	**-53.5 (-62.0 to -38.9)**	**-28.8 (-38.6 to -20.7)**	**44.7 (-2.6 to 83.8)**	**9.8 (-26.8 to 49.6)**	**61.8 (2.7 to 147.7)**
Haryana	-8.2 (-17.7 to 1.2)	5.3 (-7.3 to 20.1)	14.6 (-25.4 to 55.8)	66.3 (23.4 to 133.5)	-1.2 (-25.1 to 30.7)	57.0 (24.5 to 101.6)	66.8 (5.8 to 142.7)	-45.0 (-61.4 to -13.2)	-15.8 (-34.4 to 3.9)	82.4 (27.6 to 145.2)	24.6 (-14.1 to 91.3)	87.5 (29.3 to 169.9)
Delhi	-17.1 (-26.2 to -6.1)	-27.4 (-38.5 to -15.6)	2.0 (-27.5 to 25.7)	56.3 (27.3 to 92.6)	-42.3 (-55.7 to -26.7)	116.6 (66.5 to 196.4)	44.1 (-2.2 to 82.8)	2.8 (-18.3 to 26.7)	40.8 (7.5 to 79.3)	76.5 (12.4 to 119.7)	5.3 (-30.5 to 46.6)	92.2 (33.3 to 205.9)
Telangana	-12.5 (-22.4 to -2.2)	4.0 (-5.3 to 14.0)	4.6 (-29.3 to 35.7)	43.2 (10.2 to 103.6)	-14.8 (-35.4 to 6.1)	18.1 (-9.0 to 61.0)	27.5 (-29.8 to 105.6)	-60.4 (-71.5 to -42.2)	-48.3 (-58.3 to -34.1)	34.8 (-6.8 to 88.7)	14.2 (-30.6 to 70.8)	60.3 (-8.7 to 164.3)
Andhra Pradesh	-6.3 (-15.4 to 4.0)	2.9 (-5.9 to 11.4)	7.6 (-25.2 to 34.4)	52.7 (19.9 to 100.5)	-10.6 (-28.3 to 11.9)	14.0 (-6.8 to 48.3)	36.2 (-14.6 to 101.7)	-63.0 (-74.0 to -42.5)	-47.1 (-58.6 to -36.2)	53.5 (0.8 to 104.9)	13.7 (-23.1 to 72.0)	57.3 (-0.4 to 128.9)
Jammu and Kashmir	-12.3 (-20.8 to -3.0)	0.3 (-8.9 to 9.4)	1.2 (-29.9 to 31.2)	41.8 (13.1 to 92.8)	-20.8 (-37.4 to 1.7)	11.1 (-9.7 to 50.3)	29.6 (-18.2 to 78.0)	-59.4 (-69.6 to -40.4)	-48.3 (-57.5 to -35.5)	35.5 (-7.7 to 77.1)	9.5 (-31.3 to 49.2)	43.1 (-8.4 to 135.0)
Karnataka	-14.7 (-23.2 to -6.0)	-7.5 (-16.1 to 2.1)	5.0 (-24.9 to 32.5)	34.2 (9.8 to 75.5)	-18.1 (-32.9 to -1.3)	-0.4 (-17.4 to 61.5)	35.2 (-11.5 to 93.0)	-61.8 (-72.7 to -36.6)	-36.9 (-47.2 to -24.8)	19.4 (-0.4 to 86.4)	14.3 (-24.5 to 65.5)	46.8 (-2.4 to 134.5)
West Bengal	-10.1 (-18.4 to -1.6)	-23.3 (-31.0 to -12.7)	2.7 (-29.9 to 29.2)	33.4 (10.5 to 61.4)	-20.8 (-39.4 to 2.7)	7.5 (-14.6 to 39.0)	22.3 (-19.8 to 57.3)	-59.3 (-68.9 to -46.5)	-38.5 (-50.7 to -26.4)	19.9 (-9.5 to 51.6)	4.2 (-30.1 to 38.8)	57.5 (-1.0 to 119.1)
Maharashtra	-31.2 (-37.3 to -24.3)	-23.6 (-44.8 to -1.9)	-15.7 (-31.7 to -0.5)	52.1 (30.4 to 79.3)	-61.9 (-67.3 to -55.5)	45.5 (21.6 to 98.4)	21.8 (-10.9 to 72.0)	-42.5 (-54.8 to -31.8)	-17.3 (-31.6 to -2.5)	64.8 (-5.4 to 110.3)	5.9 (-32.9 to 46.5)	55.6 (-2.0 to 165.5)
UTs* other than Delhi	3.7 (-6.0 to 14.8)	-3.9 (-13.3 to 4.8)	4.0 (-23.1 to 35.6)	42.0 (12.1 to 85.4)	-10.9 (-24.7 to 6.0)	68.9 (42.2 to 118.9)	80.2 (3.8 to 132.9)	-40.3 (-55.4 to -8.5)	-10.8 (-27.1 to 8.8)	65.0 (17.2 to 106.6)	16.5 (-20.9 to 58.3)	96.7 (34.8 to 160.7)
**High ETL**	**-4.1 (-9.8 to 2.1)**	**-25.0 (-31.0 to -17.7)**	**-9.3 (-26.6 to 8.8)**	**65.1 (33.3 to 100.7)**	**-35.3 (-43.7 to -26.9)**	**65.8 (45.3 to 93.3)**	**37.0 (-4.6 to 93.7)**	**-37.9 (-46.8 to -30.2)**	**-5.2 (-21.6 to 7.3)**	**63.7 (1.3 to 102.0)**	**12.9 (-27.6 to 57.7)**	**55.3 (3.8 to 164.1)**
Himachal Pradesh	-7.2 (-17.5 to 4.8)	3.0 (-7.2 to 13.9)	5.9 (-27.9 to 36.2)	56.1 (17.2 to 124.2)	-14.4 (-32.0 to 12.2)	52.1 (22.7 to 102.1)	46.2 (-16.4 to 123.4)	-52.5 (-65.0 to -27.9)	-25.2 (-39.6 to -5.6)	60.6 (8.0 to 122.2)	14.6 (-26.8 to 75.8)	88.1 (13.5 to 197.0)
Punjab	10.2 (-0.5 to 21.7)	-12.1 (-21.3 to -2.9)	-7.6 (-23.8 to 13.7)	82.1 (32.6 to 154.5)	-18.7 (-33.3 to -0.9)	77.4 (49.8 to 100.7)	60.6 (-0.7 to 145.8)	-28.1 (-42.4 to -11.0)	17.5 (-18.4 to 50.5)	48.6 (9.2 to 109.3)	14.3 (-20.0 to 67.0)	61.5 (13.1 to 153.0)
Tamil Nadu	-9.6 (-17.9 to -0.6)	-39.3 (-51.0 to -24.2)	-11.6 (-29.1 to 12.2)	66.2 (30.6 to 110.0)	-35.6 (-48.5 to -22.0)	74.8 (45.7 to 103.5)	38.7 (-20.6 to 93.6)	-40.3 (-53.4 to -27.7)	-3.7 (-22.0 to 14.8)	55.2 (-2.1 to 98.8)	11.1 (-30.6 to 61.2)	80.1 (11.3 to 164.7)
Goa	-10.9 (-19.4 to -2.0)	0.6 (-8.8 to 10.1)	10.4 (-25.1 to 42.6)	57.8 (20.5 to 109.8)	-9.8 (-28.6 to 12.8)	117.4 (80.5 to 183.6)	47.3 (-12.8 to 116.1)	-40.7 (-57.7 to -7.9)	7.6 (-12.1 to 32.4)	84.2 (17.5 to 133.8)	24.4 (-26.3 to 75.0)	126.1 (41.8 to 235.4)
Kerala	-2.7 (-11.0 to 6.0)	-17.1 (-27.7 to -6.6)	-12.6 (-27.3 to 1.6)	55.7 (28.6 to 90.8)	-54.4 (-62.4 to -46.2)	62.3 (38.9 to 113.8)	28.2 (-8.4 to 88.1)	-36.6 (-44.5 to -28.8)	-14.6 (-29.1 to 2.0)	92.1 (3.8 to 133.3)	15.4 (-28.1 to 59.1)	16.7 (-20.9 to 165.5)

Range of percent change

>50.0 | 25.1 to 50.0 | 0.1 to 25.0 | -25.1 to 0.0 | -50.1 to -25.0 | < -50.1

*Union Territories. ETL is epidemiological transition level.
